# A comparative metabolomics investigation of flavonoid variation in faba bean flowers

**DOI:** 10.1007/s11306-023-02014-w

**Published:** 2023-05-30

**Authors:** Fatma M. Elessawy, Jessa Hughes, Hamid Khazaei, Albert Vandenberg, Anas El-Aneed, Randy W. Purves

**Affiliations:** 1 College of Pharmacy and Nutrition, University of Saskatchewan, Saskatoon, SK Canada; 2 Department of Plant Sciences, University of Saskatchewan, Saskatoon, SK Canada; 3 Centre for Veterinary Drug Residues, Canadian Food Inspection Agency, Saskatoon, SK Canada; 4 Present Address: Natural Resources Institute Finland (Luke), Helsinki, Finland

**Keywords:** *Vicia faba* L., Untargeted metabolomics, Flowers, Standard petal, Wing petal, Polyphenols, Kaempferol, Anthocyanins

## Abstract

**Introduction:**

Faba bean (*Vicia faba* L.) flowers are edible and used as garnishes because of their aroma, sweet flavor and attractive colors. Anthocyanins are the common plant pigments that give flowers their vivid colors, whereas non-anthocyanin flavonoids can serve as co-pigments that can modify the color intensity of flowers.

**Objectives:**

To explore the polyphenol diversity and differences in standard and wing petals of faba bean flowers; and identify glycosylated flavonoids that contribute to flower color.

**Methods:**

Flower standard and wing petals from 30 faba bean genotypes (eight color groups with a total of 60 samples) were used for polyphenol extraction. Samples were analyzed using a targeted method and a semi-untargeted analysis using liquid chromatography–high resolution mass spectrometry (LC–HRMS) combined with photodiode array (PDA) detection. Compound Discoverer software was used for polyphenol identification and multivariate analysis.

**Results:**

The semi-untargeted analysis guided by the PDA detected 90 flavonoid metabolites present in faba bean flower petals. Ten anthocyanins largely influenced the flower colors, but other flavonoids (63 flavonols and 12 flavones) found with variable levels in different flower color groups appeared to also influence color, especially in mixed colors.

**Conclusion:**

Analysis of the different colored faba bean flowers confirmed that the color variation between the flowers was mainly controlled by anthocyanins in brown, red and purple-red flowers. Of the other flavonoids, multiglycosylated kaempferols were abundant in white and brown flowers, monoglycosylated kaempferols were common in red and purple-red flowers, and quercetin and apigenin glycosides were abundant co-pigments in purple-red flowers.

**Supplementary Information:**

The online version contains supplementary material available at 10.1007/s11306-023-02014-w.

## Introduction

Faba bean (*Vicia faba* L.) is a cool-season grain legume crop (pulse) that is globally grown for human food and animal feed (Duc, 1997). It has gained importance for its seeds high protein content, nutritional value and its environmental benefits (Kopke & Nemecek, 2010). Faba bean has an efficient nitrogen fixation ability through symbiosis in its root nodules, higher than any other pulse crop (Hardarson et al., 1991; Klippenstein et al., 2022). Its seeds have a high protein content (29% of dry matter), as well as carbohydrates, dietary fiber, vitamins, and micronutrients (Khazaei & Vandenberg, 2020). Besides the nutritional value of the seeds, faba bean flowers are edible and can be used to garnish salads, soups and desserts because of their aroma, sweet flavor and attractive colors (Sutton et al., 1992). Additionally, the consumption of edible flowers provides health benefits, since they are rich in phytochemicals, such as polyphenols (Fernandes et al., 2017). Due to their free radical scavenging capability, polyphenols have been reported to reduce the risk of developing a number of chronic degenerative diseases in humans such as diabetes (Kunyanga et al., 2012), cardiovascular disease (Agouni et al., 2009) and different types of cancer (Martínez et al., 2003).

In addition to their health benefits, polyphenols as secondary plant metabolites, help plants defend themselves from pathogens and environmental stresses (Beart et al., 1985). As a class of polyphenols, flavonoids, usually glycosylated, are responsible for the vivid colors of flowers that attract insects for pollination and seed dispersal, and protect plants against UV and visible light (Grotewold, 2006; Xu et al., 2018). Among flavonoids, anthocyanins are the common plant pigments conferring orange, pink, purple and blue colors along with patterns in flowers (Diretto et al., 2019; Du et al., 2018; Iwashina & Kokubugata, 2014). Anthocyanidins are rarely found in nature in their aglycone form, instead they are typically glycosylated (anthocyanins). In fact, almost 94% of the newly characterized anthocyanins are derivatives of the six common anthocyanidins: cyanidin, delphinidin, pelargonidin, peonidin, petunidin and malvidin (He & Giusti, 2010; Wallace & Giusti, 2019). The expression levels of the anthocyanin biosynthetic pathway genes were found to be consistent with the color change in cotton (*Gossypium herbaceum*) flowers (Tan et al., 2013). Besides anthocyanins, in yellow flowers, chalcones, aurones and flavonols are found to be the main pigments (Iwashina, 2015). For example, quercetin glycosides accumulate in the vacuoles of the yellow flowers of *Clematis* cultivars (Hashimoto et al., 2008), whereas kaempferol glycosides contribute to the creamy tone of “Honey Moon” flowers (Iwashina et al., 2010). Furthermore, flavonols, flavones, flavan-3-ols and dihydroflavonols were reported to efficiently serve as co-pigments where they stabilize and modify the color intensity of flowers (Trouillas et al., 2016). Apart from pigmentation, flavonoids are abundant in edible flowers and have diverse health promoting effects (Benvenuti & Mazzoncini, 2021). In our previous study, anthocyanins showed higher antioxidant activity than glycosylated flavonols, mainly kaempferol and quercetin (Elessawy et al., 2021).

Faba bean has specialized zygomorphic flowers with papilionaceous features. The flowers consist typically of five petals comprised of a fused standard, two wings, a fused keel and one pistil with ten fused stamens (Duc, 1997). The wild-type flower color of faba bean is white with a dark spot on the wing petals and brown or violet stripes running vertically along the standard petals (Hughes et al., 2020). The color of faba bean flowers can be pure or mixed combinations of white, purple, brown, pink, red and yellow with black, brown or yellow spots on the wing petals (Alghamdi et al., 2020). Such a large range of faba bean flower colors and patterns promotes these flowers as potential candidates for the ornamental and floriculture industries (Hughes et al., 2020). Previous studies examined the inheritance of white flower color (Crofts et al., 1980; Zanotto et al., 2020), red flower color, and yellow-spotted flowers in this species (Cabrera, 1988; Sjödin, 1971). The flower color is under control of four independent recessive genes (Crofts et al., 1980; Hughes et al., 2020). Thus, no high genotype by environment interactions would be expected, although flower color may be influenced by intense environmental stresses, light intensity, and soil properties, such as soil pH that can affect anthocyanin types and levels, e.g., in *Hydrangea macrophylla* (Schreiber et al., 2011). Faba bean is adapted in soils with pH above 6.5, but less adapted to lower pH (~ 5 or less), which will restrict its growth (French and White, 2005). In general, the non-genetic factors that may influence faba bean flower color are yet to be fully understood.

There has been limited research to explain the correlation between the different colors of flower petals (mainly standard and wing petals) exerted by the controlling genes, and the flux in the biosynthetic pathway of polyphenols. Liquid chromatography–mass spectrometry (LC–MS) based metabolomics has been widely used as a powerful tool for differentiating among plant phenotypes (Patel et al., 2021; Song et al., 2021). Previous studies reported the use of LC–MS based targeted and untargeted metabolomics in determining phenotypic variation among colored flowers of *Rhododendron schlippenbachii* Maxim. (Park et al., 2018), Gladiolus (*Gladiolus grandiflora* Hort.) (Kim et al., 2016) and buckwheat (*Fagopyrum esculentum*) (Li et al., 2020). The main goal of this study was to explore the polyphenol diversity, particularly among anthocyanins, in wing and standard petals of faba bean flowers to identify key polyphenols involved in flower color using targeted and semi-untargeted LC–MS metabolomics. In addition to anthocyanins, glycosylated flavonoids, as co-pigments, can also contribute to the color intensity of the flowers, and therefore, a second objective was to explore variations in the glycosylated flavonoids among the colored flower petals.

## Materials and methods

### Plant material and growing conditions

**Fig. 1:**
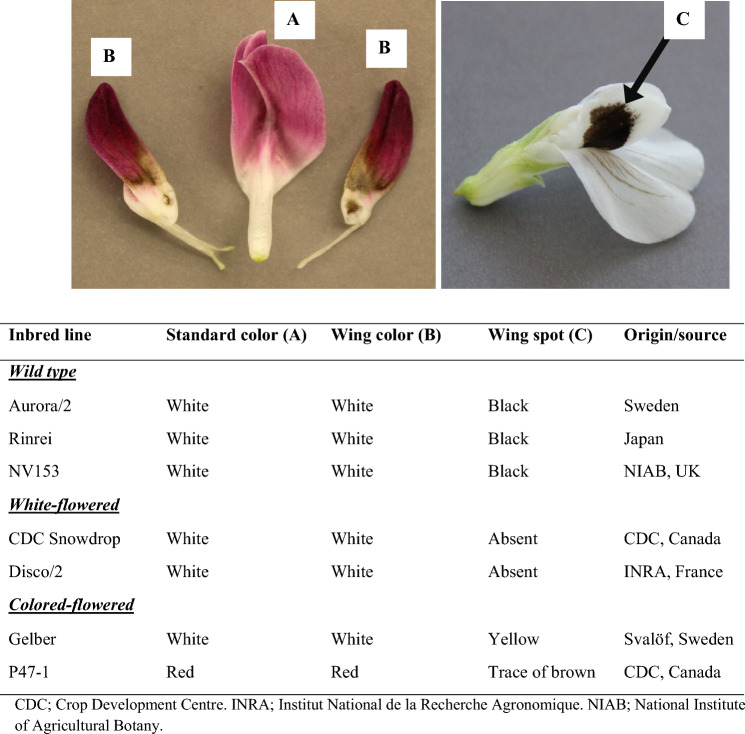
Main parts of a faba bean flower: standard (A) and wing (B) petals with/without a wing spot (C), followed by an overview of the color characteristics of floral tissues and origin of faba bean parental lines used in this study

Seven faba bean inbred lines were used to create new flower color combinations (Fig. 1). Samples of 30 progenies of nine single crosses made with seven faba bean inbred lines (Table 1) were used in this study. More details about pedigree, flower color and floral tissue used for analysis are given in Table 1. The petals of each flower were separated into standard and wing petals (Fig. 1); then each petal group was divided into nine phenotypic sub-groups based on petal color combinations as follows: brown (B), brown/red (B/R), brown/pink (B/P), red (R), purple-red (PurR), white (W), white/pink (W/P), white/yellow (W/Y) and white/brown (W/B). Note that red (R) is only present in the standards as there were no red (R) wings. The color of individual petals could be classified as uniform (brown, red, white, or purple-red) or as a mix of more than one color (brown/pink, brown/red, white/pink, white/yellow and white/brown). Note that although purple-red was grouped as a single-color, the shade of purple was visually observed to vary slightly among the group. Photos of representative petal color groups are shown in Fig. S1. Each sample had three biological replicates. The flower petal colors in this study were evaluated according to the horticultural color chart (Wilson, 1938).

**Table 1:**
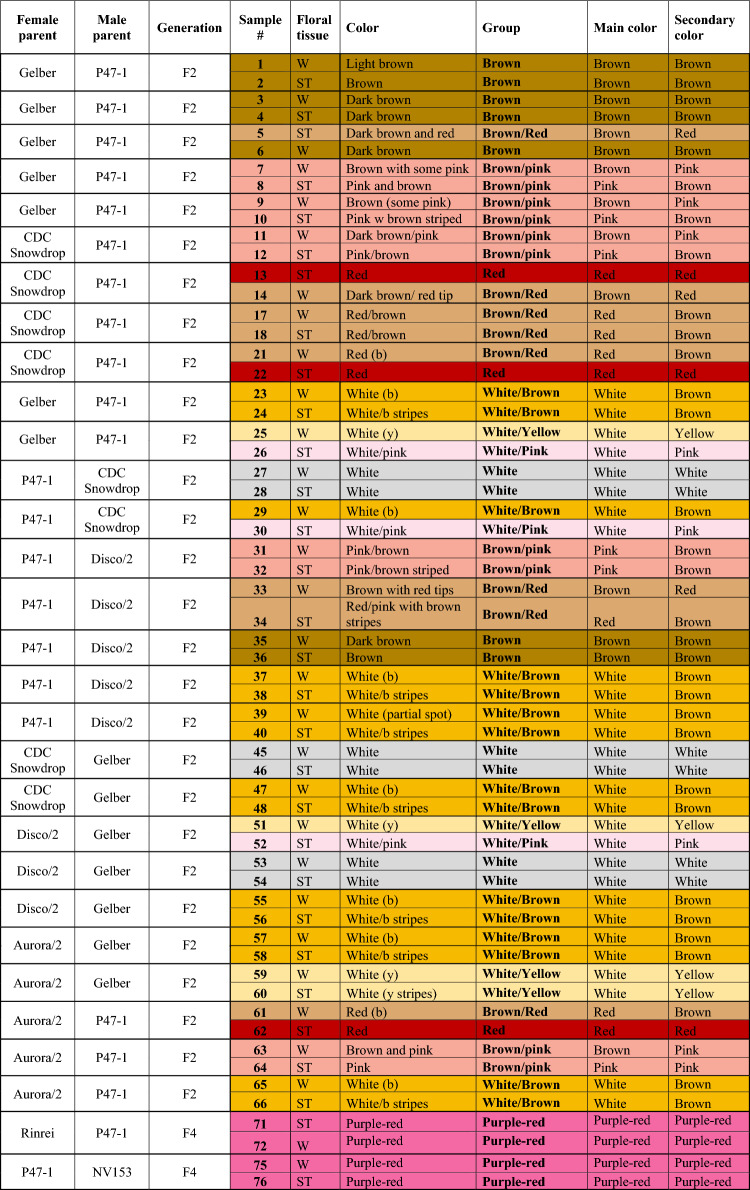
Detailed description of color phenotypes of faba bean progeny petal tissues used in this study (flower color codes are *b* brown, *db* dark brown, *p* pink, *y* yellow; floral petal codes are *ST* standard and *W* wing)

Flower samples from standard and wing petals of 30 faba bean pre-breeding genotypes with different colors (see Table 1) were harvested (60 samples in total) from plants grown in the College of Agriculture and Bioresources controlled environment facility at the University of Saskatchewan, Saskatoon, Canada. The light conditions in the phytotron chamber were 16 h days at a photon flux of 300 μmol m^−2^ s^−1^ followed by 8 h of dark. Seeds were inoculated with *Rhizobium leguminosarum* bv. *viciae* (faba bean strain) and grown in 4-L pots filled with Sun-Gro potting mix No. 3 (Sun-Gro Horticulture, Agawam, MA, USA). Beneficial mites (*Amblyseius cucumeris*) and nematodes (*Steinernema feltiae*) were used to suppress the thrips population. Flowers were harvested at 50% of the flowering stage for all genotypes.

### Sample extraction

Fresh flowers were harvested from plants in full bloom. Flower extracts were prepared using a procedure described previously (Elessawy et al., 2020), which was derived from (Mirali et al., 2014). The same extracts were used for both targeted LC–MS and untargeted LC–HRMS analysis. The only modification in this study, compared with previous work (Elessawy et al., 2020), was that ~ 15 mg of freeze-dried powdered flower tissue was used (instead of 50 mg) since less flower tissue was available.

### Targeted LC–MS analysis of the flower extracts

A targeted LC–MS method developed in our lab (Elessawy et al., 2020) was used to identify flavonoids present in the flower extracts. A modification in this study was that the method was transferred to a newer platform, namely a Vanquish ultra-high performance liquid chromatography (UHPLC) system coupled to a TSQ Altis triple quadrupole mass spectrometer (Thermo Fisher, San Jose, CA). The same chromatographic conditions, column and mobile phase (containing 1% formic acid) were used (Elessawy et al., 2020). Note that the formic acid concentration in the mobile phase is a critical factor for anthocyanin peak shape; 1% formic acid in the mobile phase gave the best trade-off between anthocyanin peak widths and the intensity of the other flavonoids.

As targeted methods are limited in their scope, a photodiode array detector (PDA) was added in-line with the mass spectrometer to ensure all flavonoids present in the flower extracts were detected (i.e., LC–PDA–MS). Thus, in addition to the MS data, UV–vis spectra were acquired simultaneously using the wavelength range of 200–680 nm. The wavelength region of 380–680 nm was used in the analysis to identify compounds that could affect the flower color.

### Untargeted LC–HRMS analysis of the flower extracts

To identify all major flavonoids in the faba bean flower extracts, the extracts were also analyzed using LC–HRMS on a Quadrupole-Orbitrap (Thermo Fisher Q-Exactive) mass spectrometer with a HESI (heated ESI) source. The Q-Exactive acquired full scan data using a mass resolution (full width at half maximum, FWHM, @ *m/z* 200) of 140,000 in negative mode with a mass range of 140–1800 *m/z*. A QC (quality control) sample, which contained equal amounts of all 180 samples (60 complete flowers; 30 standard petals and 30 wing petals with three replicates each), was injected every 10–12 runs to account for any change in retention time or signal intensity and used for relative quantification (Sangster et al., 2006). In addition, ID (identification) samples, which contained an aliquot from all the samples within a color group (one for brown (B), brown/red (B/R), etc.), were prepared for obtaining fragmentation data. The scan function “Full scan/ddMS2” was used to acquire fragmentation data (i.e., ddMS2) on the most abundant ions detected in full-scan mode. Mass resolution of the full scan analysis was 70,000 (FWHM @ *m/z* 200) and MS/MS was carried out on the 7 most abundant peaks at a resolution of 17,500 (FWHM @ *m/z* 200) from each scan using a stepped collision energy fragmentation. ID samples were injected three times using collision energies of 10/15, 30/40, and 55/75 eV. The MS/MS acquisitions used an exclusion list (*m/z* values) of the most intense ions detected from the blank sample.

### Data analysis using the Compound Discoverer software

A customized untargeted workflow was developed by adapting an existing workflow in Compound Discoverer 3.2 software (Thermo Fisher), which is used to process LC–HRMS raw data. The workflow is similar to one reported previously (Elessawy et al., 2021). The Compound Discoverer parameters used in generating the analysis are incorporated in a “Summaries” window by the software. Tabs within this summary include “Workflow”, “Study”, “Grouping and Ratio”, and “Filters”. These tabs are in text format and the outputs for this study are given in the Supplementary materials (Summary S1). In the workflow, the full-scan accurate mass data is used to determine the possible molecular formula for each *m/z* value and MS2 spectra from ID samples to help identify compounds. In addition to using Thermo’s mzCloud library, which contains fragmentation data of over 19,000 compounds analyzed with Thermo Orbitrap instrumentation (http://www.mzcloud.org), the MS2 spectra were also compared using the mzVault node to an in-house library at the Core Mass Spectrometry Facility (University of Saskatchewan, Saskatoon, Canada). Fragmentation spectra from several other libraries were also used offline, including libraries available in public databases, such as the human metabolome database (hmdb.ca). A post-processing node “Differential Analysis” is used to find significant statistical differences between sample groups using interactive visualizations, such as volcano plots and heat maps. Principal component analysis (PCA) and hierarchical cluster analysis (HCA) were the primary differential analysis tools used in this study. The CD software uses PCA to visualize the correlation between multivariate data. The software generates a scores plot, loadings plot and a variances plot. The scores plot interprets relationships among sample groups, whereas the loadings plot interprets relationships among the variables. Typically, a high-quality PCA plot will show the QC data points as a tight cluster, which indicates reliable differences among samples. Figure S7 shows QC clusters in PCA plots (Figs. 3, 4; Figs. S3 and S4) used in this study. HCA in the Compound Discoverer software uses an agglomerative (bottom-up) approach to find the similarities between the samples and compounds. We used normalized areas and "scale before clustering", which applies a z-score transformation before performing the hierarchical clustering. With this scaling, the heat map legend displays the range of the scaled values, and the dendrogram nodes display the scaled distance values. For the remaining user defined variables in the HCA analysis, “Distance Function” and “Linkage method”, the defaults “Euclidean” and “Complete” were used, respectively.

The resulting data was filtered to focus primarily on flavonoids present in the extracts. Thus, MS peaks generated by the software needed to have a corresponding UV peak (380–680 nm) to be included, and a retention time window between 5 and 18 min was used since this retention time window covers the majority of flavonoids (Elessawy et al., 2021).

## Results and discussion

### LC–PDA–MS analysis of the flower extracts

**Fig. 2:**
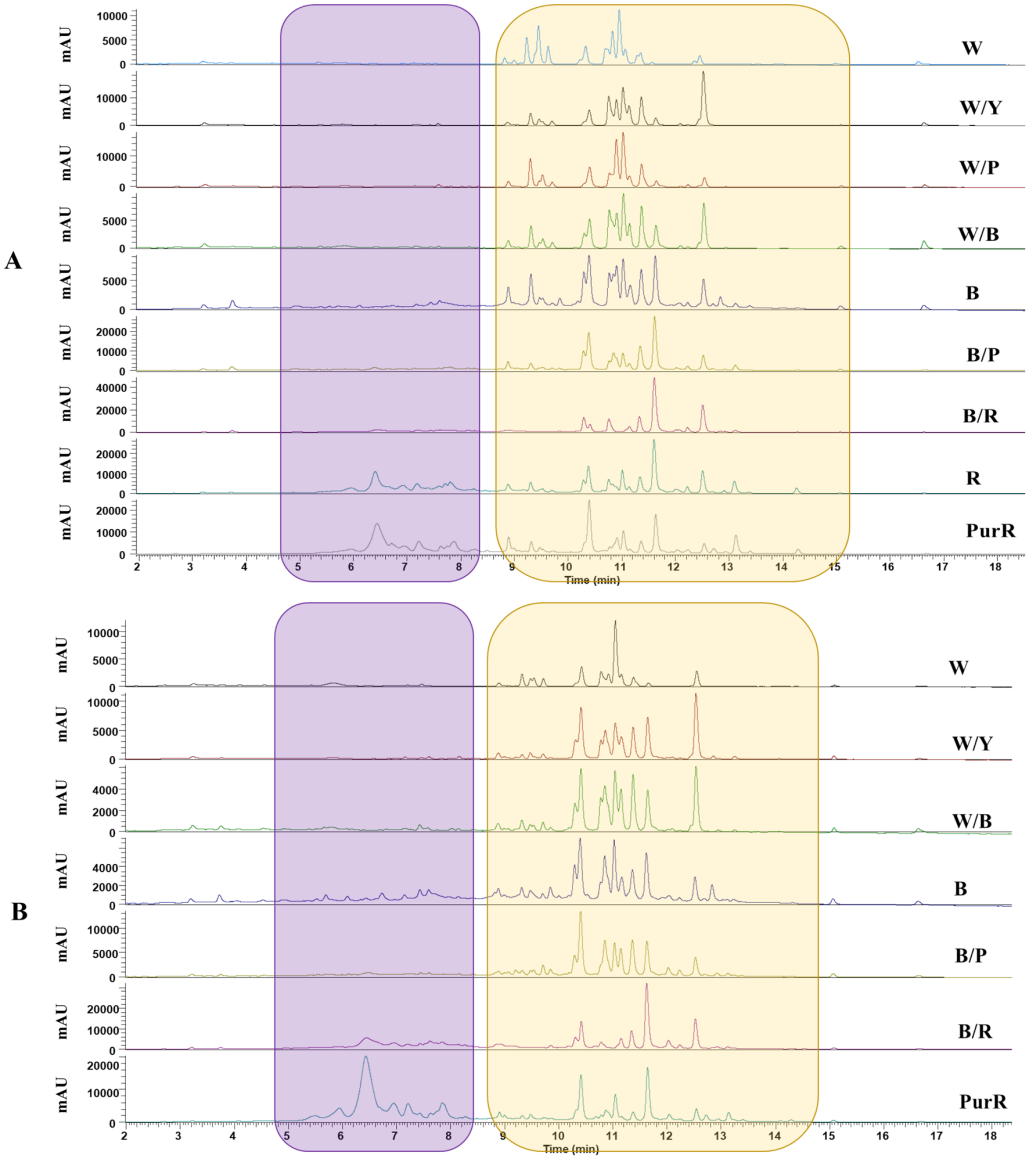
LC–UV–visible spectra (380–680 nm) examples of faba bean flower standard (**A**) and wing (**B**) petals. *W* white, *W/P* white/pink, *W/Y* white/yellow, *W/B* white/brown, *B* brown, *B/P* brown/pink, *B/R* brown/red, *R* red, *PurR* purple-red and *mAU* milli-absorbance units. Highlighted regions are underlined and italicized in Sect. 3.1

Absorbance spectra from the LC–PDA–MS analyses of flower standard and wing petal extracts in the UV–visible range of 380–680 nm given in Fig. 2A, B, respectively, show two regions of interest. The highlighted region in the yellow box in Fig. 2 shows several peaks with retention times of 9–14 min; previous work with our targeted LC–MS method (Elessawy et al., 2020), which contains 98 polyphenols, suggests that peaks in this region correspond mostly to flavonols and flavones. However, many of these UV–vis peaks do not correspond to compounds in the targeted method, which suggests that many compounds in the extract are not analytes in our targeted method. A previous study using white (low tannin) faba bean flowers revealed that flower tissue contains a large amount of highly glycosylated flavonols (Zanotto et al., 2020), many of which do not have authentic standards. Thus, as will be described further in Sect.  3.2 (semi-untargeted analysis), many of the observed peaks do in fact correspond to highly glycosylated flavonols that are not included as analytes in our targeted method.

The region highlighted by the purple box in Fig. 2 shows peaks eluting between 5–8 min, which are most noticeable in the purple-red petals (PurR) spectrum. Many of these wider peaks observed in this retention time window were suspected to be anthocyanins since the wavelength range (490–550 nm) of the UV–vis spectra is indicative of the absorbance of anthocyanins (Giusti & Wrolstad, 2001), as is shown in Fig. S2 for the flowers. The more intense UV–vis traces for standard (A) and wing (B) petals in Fig. S2 are essentially identical to their traces in Fig. 2 (i.e., PurR) indicating that the reduced wavelength range is representative of the major species in this region. Besides PurR, in Fig. S2A, B, the brown (B), brown/pink (B/P), brown/red (B/R) petals, and red (R) standards (recall no red wings) show absorbance bands in the visible region indicative of anthocyanins. Conversely, the much lower intensity absorbance bands that are present in all spectra, but especially visible in the white flower spectra (e.g., at 5.8 min), correspond to background noise. The anthocyanins have slightly wider peaks in the LC–UV–vis spectra as a result of the LC gradient pH value, which was a compromise made in optimizing the detection of *all* polyphenols in our targeted method (Elessawy et al., 2020). Identities of many UV–vis peaks in the purple region in Fig. 2 were confirmed with the targeted method but some unknown peaks were also present. To help identify all of the UV–vis peaks in Fig. 2 that could not be identified by the targeted method, a semi-untargeted analysis was carried out.

### Semi-untargeted metabolomics profiling of flavonoids in faba bean flowers

An untargeted analysis can readily yield thousands of compounds. To focus the analysis only on flavonoids in the flowers, the UV–vis spectra were used to guide the Compound Discoverer software analysis (Sect. 2.5), thereby making this a semi-untargeted approach. Occasionally, the software separately identified adducts and isotopic features instead of grouping these as one metabolite. Therefore, these additional features were manually removed prior to differential analysis as was done in our previous work (Elessawy et al., 2021). There were 90 suspected flavonoid metabolites detected by MS that had corresponding peaks in the UV–visible region (380–680 nm) with retention times between 5 and 18 min (LC–HRMS).

Our identification of these 90 metabolites initially focused on anthocyanins, whose MS peaks also showed corresponding peaks in the narrowed UV–vis range of 490–550 nm (Fig. S2), since anthocyanins will have the most influence on flower color. Ten suspected anthocyanin metabolites were discovered; our targeted LC–MS method was used to confirm the identities of seven of them, and the three remaining unknown metabolites were investigated further. Interestingly, full scan mass spectra for anthocyanins analyzed in negative ion mode typically produce unique doublet ions, a water adduct [M-2H + H_2_O]^−^ in addition to the [M-2H]^−^ ions (Sun et al., 2012), which helped to confirm the remaining metabolites in this sub-class were anthocyanins. Identities of all anthocyanins detected in this study are shown in Table 2**,** and consistent with Sumner and colleagues (Sumner et al., 2007), identification level 1 corresponds to metabolites confirmed with an authentic standard (used in our targeted method), whereas identification level 2 is putative. Lower collision energies were used to identify the masses of the glycans, whereas higher collision energies were used to putatively identify the aglycones by comparing fragmentation spectra to authentic standards and confirming the presence of key fragment ions. Although the location of the glycosylation could not be determined from our experiments, the three remaining anthocyanin peaks were putatively identified as 3-*O*-glycosides because glycosylation at 3-OH (hydroxyl group at C3) is the most common among the naturally occurring anthocyanins based on previous studies (Griesser et al., 2008; Montefiori et al., 2011).

**Table 2:** Anthocyanins detected and identified in faba bean flowers. Identification levels are: confirmed (1) and putative (2)

RT (min)	Formula	Calc. molecular weight	Precursor ion/water adduct ion (*m/z*)	Mass error (ppm)	Compound ID	ID level
6.10	C_21_ H_20_ O_12_	464.0964	463.0892/481.0996	2.07	Delphinidin-3-*O*-glucoside	1
6.56	C_27_ H_30_ O_16_	610.1542	609.1475/627.1584	1.36	Delphinidin-3-*O*-rutinoside	1
7.11	C_27_ H_30_ O_15_	594.1588	593.1515/611.1619	0.51	Cyanidin-3-*O*-rutinoside	1
7.13	C_21_ H_20_ O_11_	448.1008	447.0949/465.1035	0.54	Delphinidin-3-*O*-rhamnoside	1
7.29	C_22_ H_22_ O_12_	478.1116	477.1040/495.1148	0.93	Petunidin-3-*O*-glucoside	2
7.66	C_28_ H_32_ O_16_	624.1699	623.1624/641.1728	1.42	Petunidin-3-*O*-rutinoside	2
7.91	C_21_ H_20_ O_10_	432.1063	431.0985/449.1092	1.48	Cyanidin-3-*O*-rhamnoside	1
8.13	C_28_ H_32_ O_15_	608.1755	607.1680/625.1788	2.20	Peonidin-3-*O*-rutinoside	1
8.36	C_23_ H_24_ O_12_	492.1276	491.1213/509.1317	1.63	Malvidin-3-*O*-glucoside	1
8.62	C_29_ H_34_ O_16_	638.1861	637.1778/655.1887	2.20	Malvidin-3-*O*-rutinoside	2

**Fig. 3:**
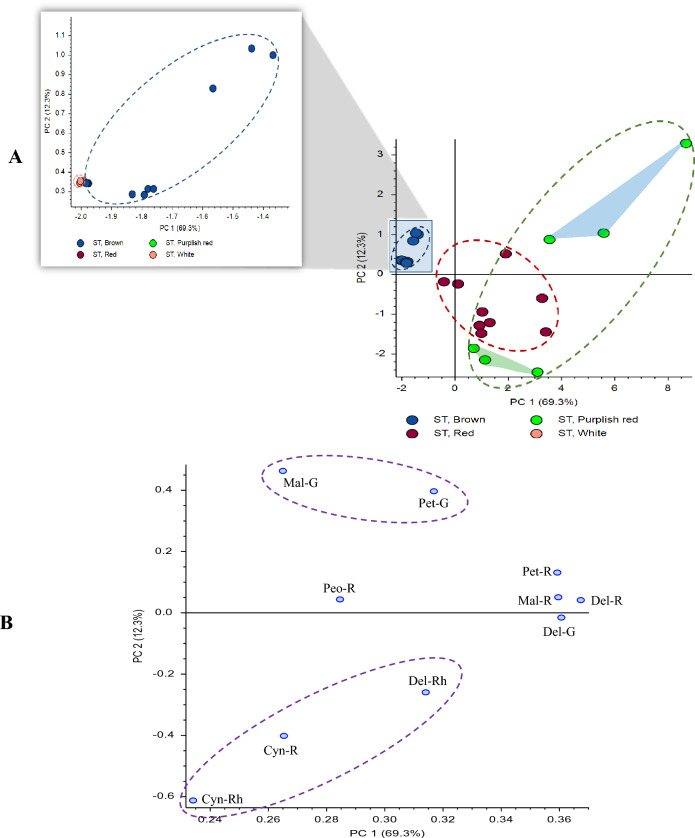
Principal component analysis. **A** scores plot, **B** loadings plot of single-colored standards based upon their main color and including identified anthocyanins only. Green highlighted triangle refers to PurR1 group, whereas blue highlighted triangle refers to PurR2 group. *Mal* malvidin, *Pet* petunidin, *Cyan* cyanidin, *Peo* peonidin, *Del* delphinidin, *G* glucoside, *R* rutinoside and *Rh* rhamnoside

**Fig. 4:**
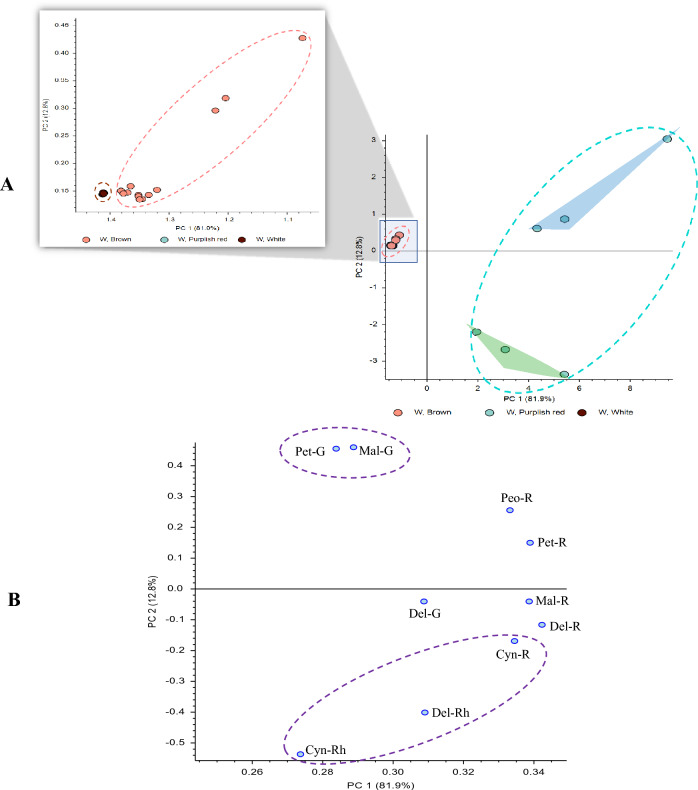
Principal component analysis. **A** scores plot, **B** loadings plot of single-colored wings based upon their main color and including identified anthocyanins only. Green highlighted triangle refers to PurR1 group, whereas blue highlighted triangle refers to PurR2 group. *Mal* malvidin, *Pet* petunidin, *Cyan* cyanidin, *Peo* peonidin, *Del* delphinidin, *G* glucoside, *R* rutinoside and *Rh* rhamnoside

Using the anthocyanins identified in Table 2**,** differences among the color groups were investigated further. Since some of the flower phenotypes with petals exhibit stripes or tips with two distinct colors (i.e., entries having a “/” within the column “group” in Table 1; sample pictures are shown in Fig. S1) that added complexity to the interpretation, for simplicity, initially only anthocyanins in one-colored flowers were examined. The anthocyanins identified in Table 2 were used to generate a principal component analysis (PCA) scores plot (PC1 vs PC2) of the single-colored standard petals (white, brown, red and purple-red) shown in Fig. 3A, and the single-colored wings (white, brown and purple-red) shown in Fig. 4A. Recall that although purple-red is considered a single-color, visually the shade of purple varied among the flower phenotypes. Both PCA scores plots show that brown and white colors cluster close together in the plot, whereas red and purple-red standard petals (Fig. 3A) or purple-red wing petals (Fig. 4A) are distinct from the white and brown clusters. An expansion of the region with brown/white in both figures shows that these groups do in fact separate. Since anthocyanins are essentially completely absent in the white (standard and wing) petals, and only present in significantly lower amounts in brown compared with red or purple-red, white and brown are found very close together on the plot. However, within the purple-red groups in both Figs. 3A and 4A, there is a large change in PC2 within the group that is not observed for the other colors. That is, one group of purple-red (PurR1 in the green highlighted triangle) flowers shows up in the region with the red genotypes, but another group of purple-red (PurR2 in the blue highlighted triangle) shows up in a separate region of the scores plot. PCA loadings plots, which indicate how strongly each metabolite influences a principal component in a PCA plot, are shown in Figs. 3B and 4B. Figure 3B shows that in the standards the two cyanidin glycosides (and to a lesser extent delphinidin 3-*O*-rhamnoside) have the largest influence in the region where the red genotypes and PurR1 are observed, presumably influencing the red color in flower petals. This correlation is supported by Vergara et al*.* who found that cyanidin glycosides are principally responsible for the bright red color of the Chilean national flower (Vergara et al., 2009). The loadings plot is not as definitive for the PurR2 group, although the petunidin and malvidin glycosides appear to be most important, especially petunidin-3-*O*-glucoside and malvidin-3-*O-*glucoside. Figure 4B shows that the loadings plot for the wings is similar to the standards, although there are some small differences that are observed. For the PurR1 group, the three most influential metabolites are the same, however, cyanidin-3-*O*-rutinoside appears less influential than delphinidin-3-*O*-rhamnoside. Although there are also some changes in the region for PurR2, petunidin-3-*O*-glucoside and malvidin-3-*O*-glucoside again appear to be the most important, suggesting that these compounds are important components in influencing the resulting purple shade in the purple-red flower petals.

**Fig. 5:**
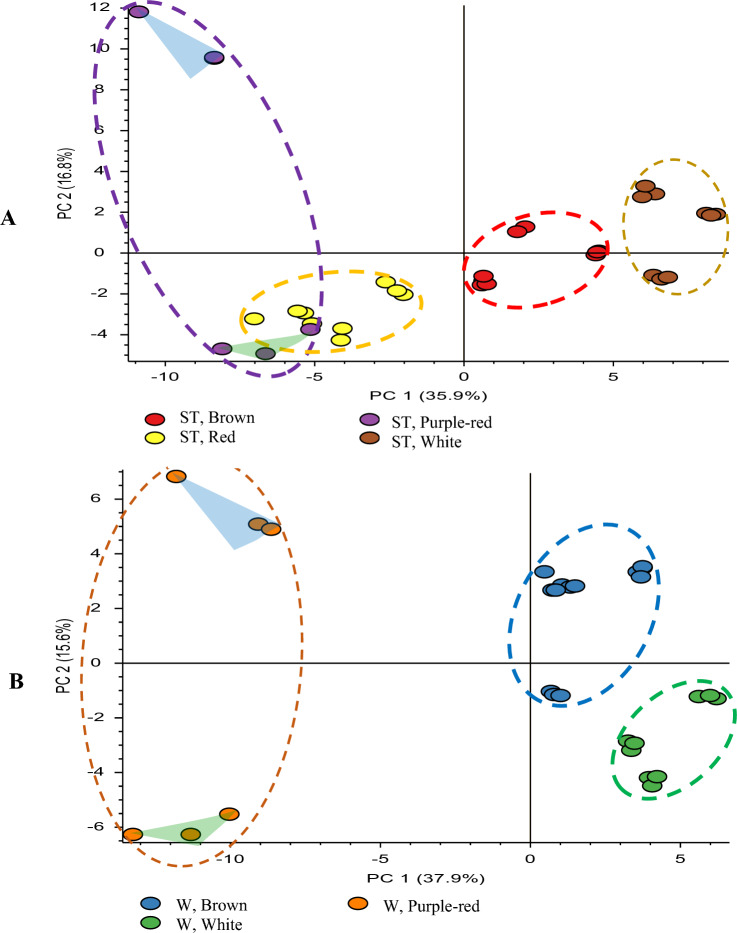
Principal component analysis of **A** single-colored standard petals, **B** single-colored wing petals based upon their main color using 88 flavonoids identified in faba bean flower petals. Green highlighted triangle refers to PurR1 group, whereas blue highlighted triangle refers to PurR2 group

Although the major differences between the colors in both standard and wing petals can be largely explained by anthocyanin composition, anthocyanins are only a subclass of the flavonoids. Therefore, differences in the other flavonoids were also investigated among different flower colors, especially since some of these compounds could be co-pigments that can influence the color intensity of the flower petals (Asen et al., 1972; Goto & Kondo, 1991; Thompson et al., 1972). In addition to the 10 anthocyanins discussed above, these 90 suspected flavonoid metabolites were found to include 63 flavonols, 12 flavones and 3 dihydroflavonols; the majority of the flavonoids were glycosylated. Table S1 shows identities of the remaining 80 flavonoids (anthocyanins are shown in Table 2). These flavonoids were identified in a similar way as was described for the anthocyanins. That is, low collision energies were used to identify the masses of the glycans, whereas high energy fragmentation spectra were compared with fragmentation spectra of authentic standards to confirm key fragment ions of the aglycones. Although the aglycone can be putatively identified based on characteristic fragment ions, the exact type of glycone (e.g., glucose or galactose) and the location of attachment are not known. Thus, in Table S1, the type of glycone (e.g., hexose) is indicated and the identification level is 2/3. This level, which lies between levels 2 and 3 used in Sumner’s work (Sumner et al., 2007), was used in our previous work to indicate that the aglycone is putatively identified, but only the type of glycone can be determined (Elessawy et al., 2021). Using the 88 flavonoids identified at level 2/3 or higher, PCA plots were generated for standard and wing petals separately and are shown in Fig. 5A, B, respectively. For standard petal groups, the clusters for white and brown flowers are separate, whereas red and purple-red clusters remain partially overlapped. For the wing petal groups, there are three separate clusters for the white, brown and purple-red (also as was the case in Fig. 4A). In both Fig. 5A, B, there is still a lot of variability in PC2 within the purple-red group (PurR1 and PurR2). Loadings plots were used again to investigate key flavonoids responsible for the separation of purple-red petals. Thus, the corresponding loadings plots for the PCA plots shown in Fig. 5A, B are given in Figs. S3 and S4, respectively. Besides the malvidin and petunidin glucosides, three additional anthocyanins, five multi-glycosylated quercetins, and five other glycosylated flavonols were found in the same region as the PurR2 group in the PCA plot of standard petals in Fig. S3. Conversely, four mono- and three di-glycosylated kaempferol and two C-glycosylated apigenin derivatives appear in the region with the red and PurR1 groups along with cyanidin 3-*O*-rhamnoside in standard petals. The loadings plot for the wings in Fig. S4 shows the same blue dots identified as influential in the standard petals. The figure shows that many of the same metabolites that are important flavonoids identified in standards are also important in the wings. Essentially all of the additional flavonoids identified as potentially important in the wings in Fig. S4 were quercetin glycosides.

**Fig. 6:**
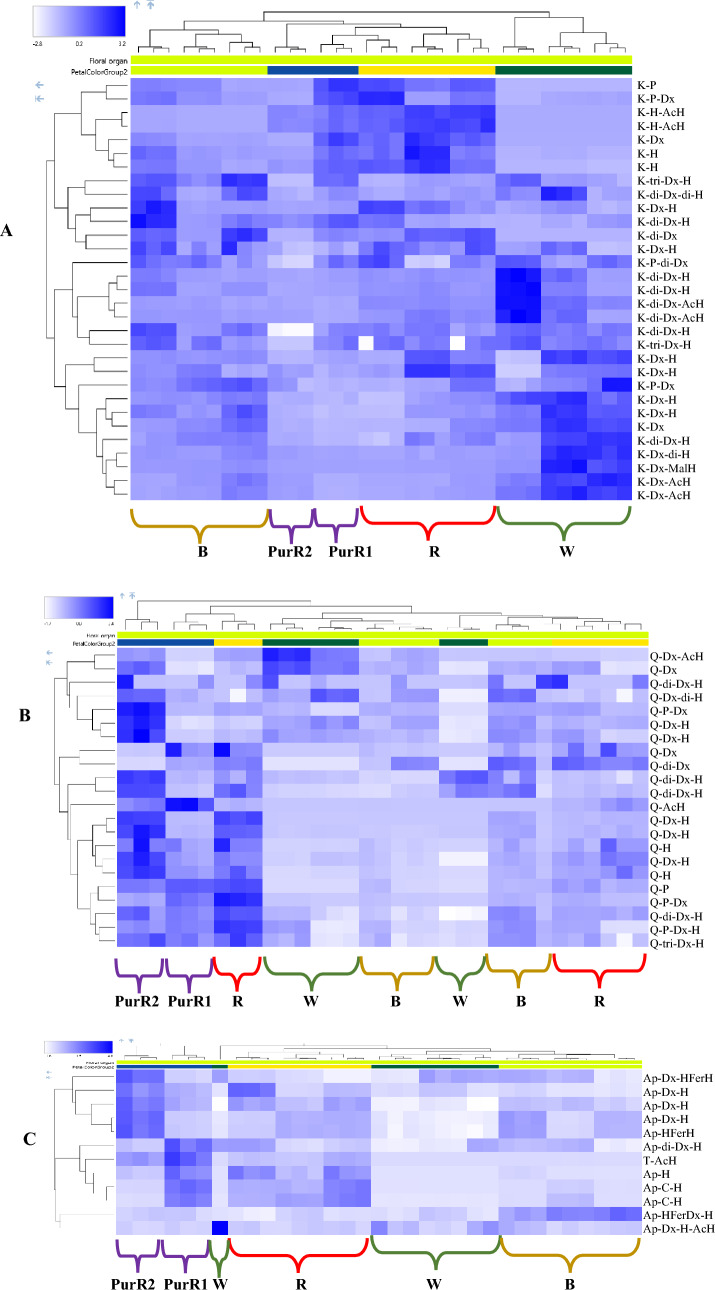
Hierarchal clustering analysis (HCA) plot of single-colored faba bean standard flower petal groups (*B* brown, *PurR* purple-red, *R* red and *W* white) using identified glycosylated **A** kaempferol, **B** quercetin and **C** flavone derivatives. Each rectangle represents a metabolite, and its color intensity refers to the relative amount of that specific metabolite in a specific sample. The areas were scaled before clustering by applying a z-score transformation and the heat map legend displays the range of the z-score values. *K* kaempferol, *Q* quercetin, *Ap* apigenin, *T* tricetin, *Dx* deoxyhexoside, *H* hexoside, *P* pentoside, *AcH* acetyl hexoside, *MalH* malonyl hexoside, *HFerH* hydroxyferuloyl hexoside

Hierarchical clustering plots (HCA) were also generated for the major flavonols and flavones to help illustrate differences observed in the PCA plots. HCA plots for standard petals are shown in Fig. 6. Figure 6A shows the distribution of glycosylated kaempferol derivatives in single colored flower standard petals. Mono-glycosylated kaempferol derivatives are typically found in higher amounts in red and PurR1 petals, whereas multi-glycosylated kaempferol derivatives (especially the tri- and tetra- glycosylated) have the highest levels in white petals. The purple-red genotypes (PurR2) in the top left quadrant of the plots in Fig. 5 show low amounts of kaempferol glycosides, whereas the other group (PurR1) shows a similar distribution to the red petals. Figure 6B shows the distribution of glycosylated quercetin derivatives in single colored flower standard petals. Interestingly, the purple-red petal (PurR2) group shows many intense quercetin glycosides (Fig. 6B), whereas other color groups (including PurR1) have lower amounts of quercetin glycosides. The observations in Fig. 6A, B support the findings from the loadings plots. Figure 6C shows that 10 of 12 flavone metabolites are present in much lower amounts in white and brown petals compared with the red and purple-red petal groups. The purple-red groups show two very different distributions of glycosylated apigenin derivatives. The presence of high levels of glycosylated quercetin and apigenin derivatives in purple-red (PurR2) compared with red petal groups might explain the intense red color (in purple-red) since they were reported to be strong co-pigments (Li et al., 2018; Trouillas et al., 2016). The HCA plots highlight large differences in the polyphenol profiles within the purple-red group despite the fact that only small visible changes were observed. HCA plots of single-colored wing petals are shown in Fig. S5. Similar to the standard petals, the differences that were observed in the PCA scores and loadings plots (Fig. 4) for the wings, are also reflected in the HCA plots for the wings (Fig. S5).

PCA plots were also generated using the identified flavonoids in mixed colored petals and these are shown in Fig. S6. The PCA plots for the mixed colors showed a lot of scatter and no distinct pattern, therefore they will not be discussed further.

## Conclusions

In this study, a semi-untargeted analysis revealed 90 flavonoid polyphenols in different colored faba bean flowers. The data analysis confirmed that the color variation between the flowers was primarily controlled by ten anthocyanins (Table 2) in brown, red and purple-red flowers. Cyanidin glycosides were associated with the bright red color of standard petals, whereas malvidin and petunidin glucosides were more important to purple-red standard petals. Although some small differences were observed, the wing petals showed a similar pattern. Of the remaining flavonoids, the majority were glycosylated flavonols, most of which contained kaempferol or quercetin. Multi-glycosylated kaempferols were found to be the most abundant in white flowers, whereas red petals contained the highest amounts of the mono-glycosylated kaempferols. Significant differences in the polyphenol profile among purple-red flowers were observed with small changes in the shade of purple and therefore these flowers were treated as two groups, PurR1 and PurR2. PurR1 standards were more similar to red standards having more cyanidin and kaempferol glycosides, whereas PurR2 standards contained more petunidin, malvidin, quercetin and apigenin glycosides. Also, results suggested that apigenin glycosides and multi-glycosylated quercetins might be related to the purple shade in PurR2 petals as these flavonoids were most abundant in this color group. When the mixed petal colors were compared with the pure colors, no distinguishable clusters were observed suggesting complex relationships among the flavonoids in these flowers. This study has revealed some interesting patterns among polyphenolic profiles and the diverse faba bean flower petal colors. However, because of the complexity of these relationships, as illustrated by the absence of distinguishable clusters in the mixed petals, further studies will be needed before this information can help guide faba bean breeders. For example, implementing a much larger study involving more controlled crosses between multiple genotypically distinct parent lines with the same flower color by a common parent line of a different flower color. A common parent would reduce variability and help to better understand the polyphenolic contributions of specific genotypes. Also, studies exploring the flux in the biosynthetic pathway for these polyphenols during flower development could be valuable as the current study only examined polyphenol composition at one time point.

## Supplementary Information

Below is the link to the electronic supplementary material.

Supplementary file1 (DOCX 2624 KB)

## Data Availability

The data from this article are available from the corresponding author on reasonable request.
