# Supplementary material for: A comparative metabolomics investigation of flavonoid variation in faba bean flowers
Source: Metabolomics. 2023 May 30;19(6):52. doi: 10.1007/s11306-023-02014-w (PMC10229742; doi:10.1007/s11306-023-02014-w)
Supplement: Supplementary file 1 — Supplementary file1 (DOCX 2624 KB) [file 11306_2023_2014_MOESM1_ESM.docx]

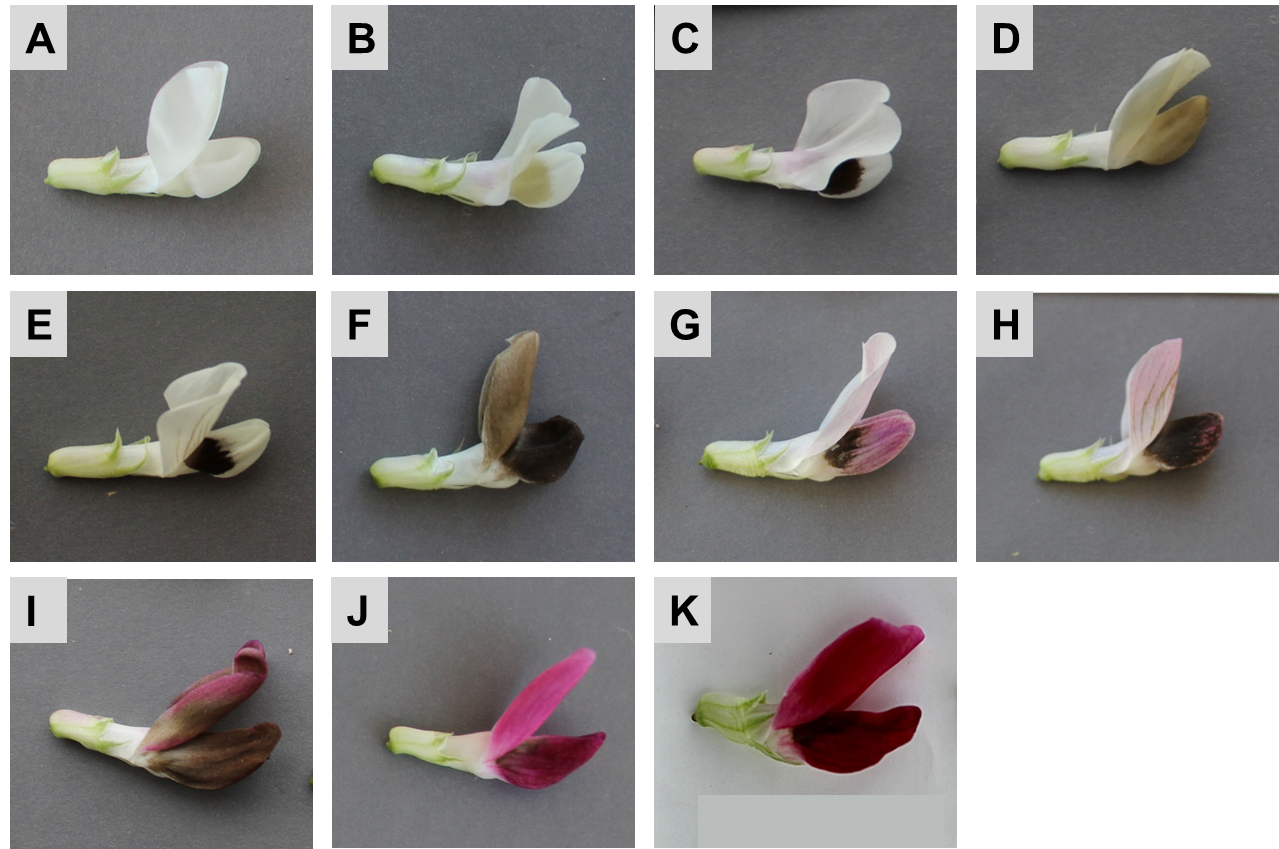


**Figure S1**. Pictures for faba bean flowers with various colored petals: A) white ST & white W, B) white/pink ST & white/yellow wing, C) white/pink ST & white/yellow W, D) white yellow ST & white yellow W, E) white/brown ST & white/brown W, F) brown ST & brown W, G) pink ST & pink/brown W, H) pink/brown ST & pink/brown W, I) red/brown ST & red/brown W, J) red ST & red/brown W, and K) purplish red ST & purplish red W. ST: standard petal, and W: wing petal.


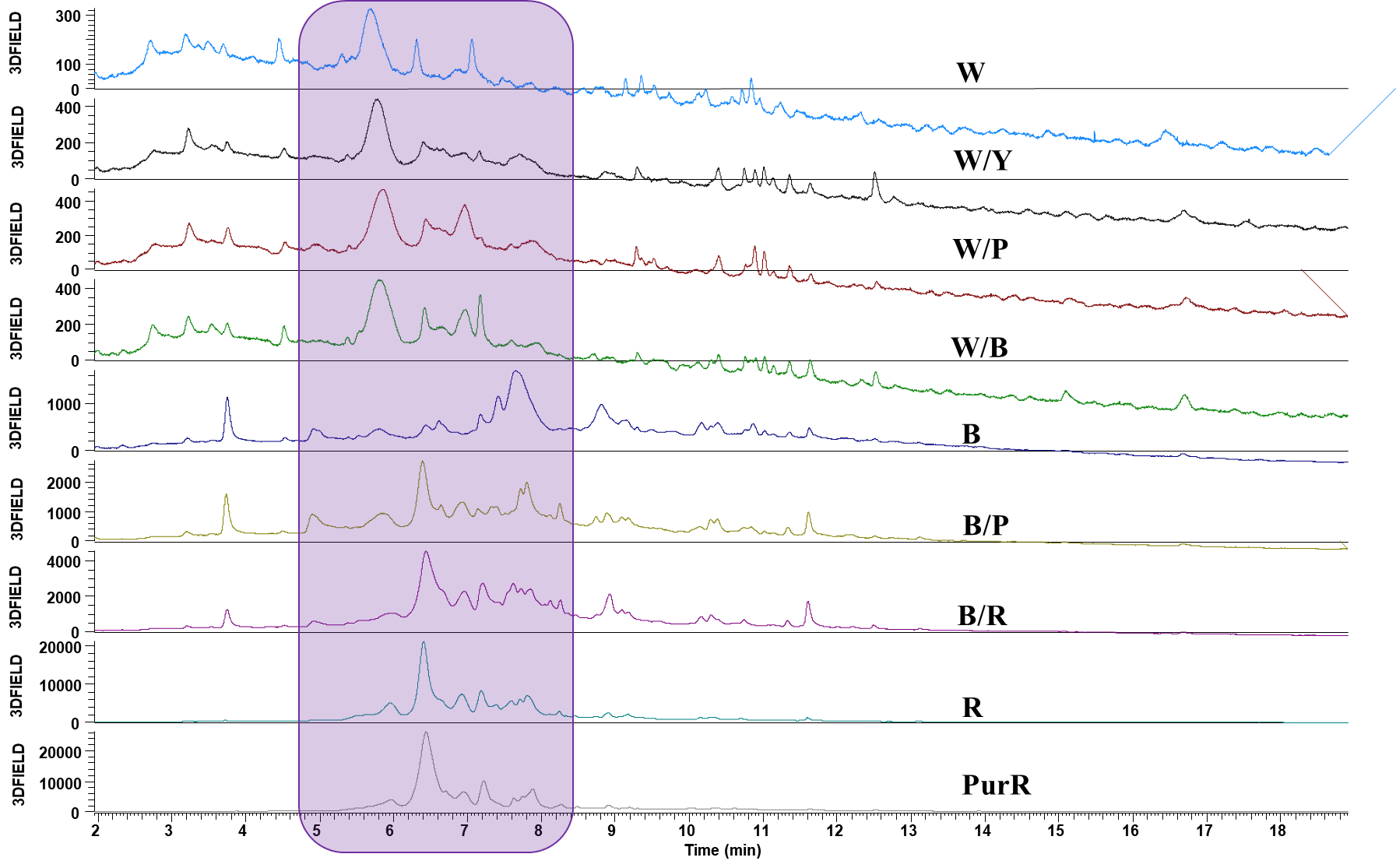


**mAU mAU mAU mAU mAU mAU mAU mAU mAU**

**A**


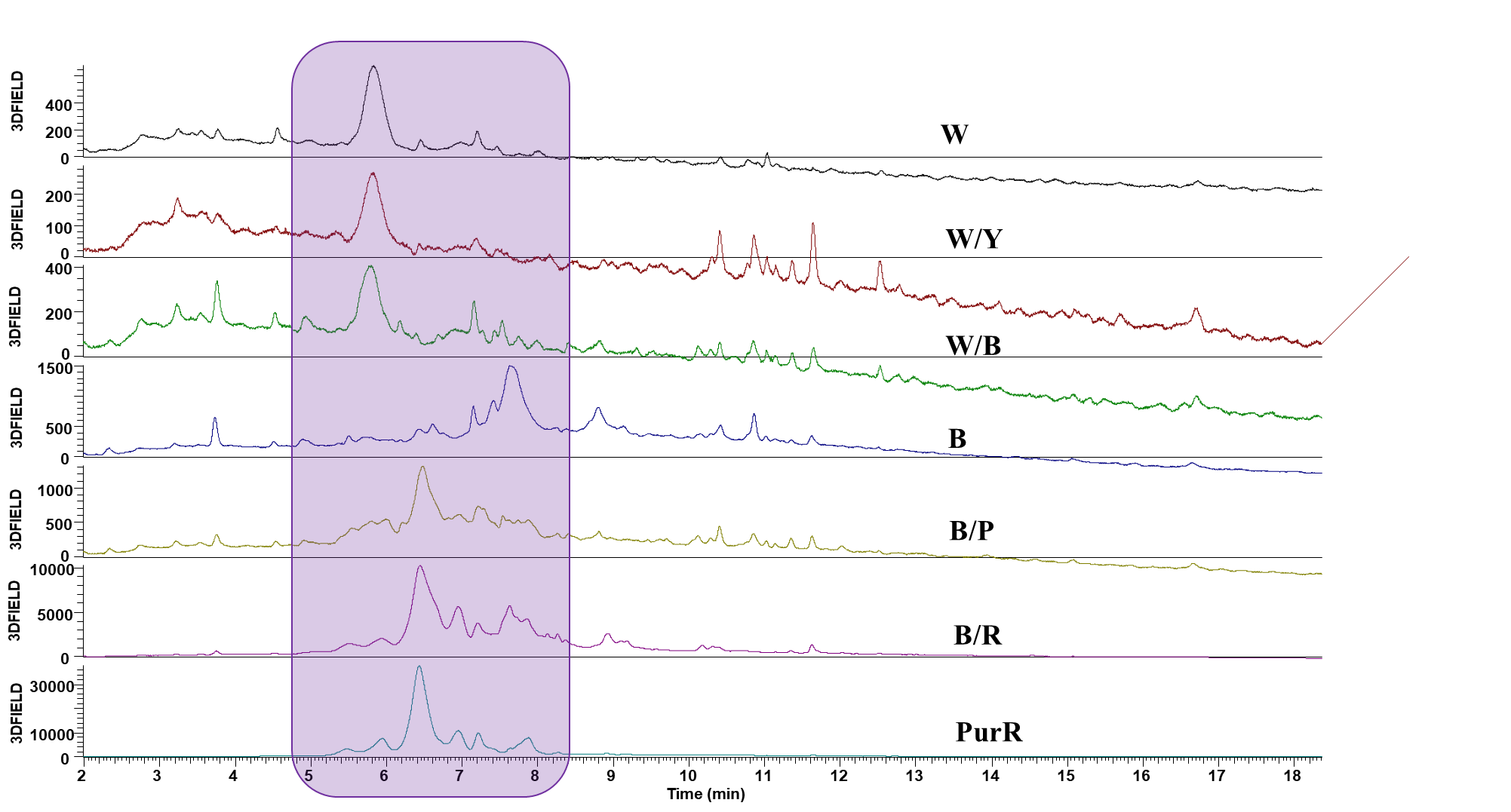


**mAU mAU mAU mAU mAU mAU mAU**

**B**

**Figure S2.** LC-UV-visible spectra (490-550 nm) of faba bean flower standard (A) and wing (B) petals (an example of each color category). W: white, W/P: white/pink, W/Y: white/yellow, W/B: white/brown, B: brown, B/P: brown/pink, B/R: brown/red, R: red, PurR: purple-red and mAU: milli-absorbance units.


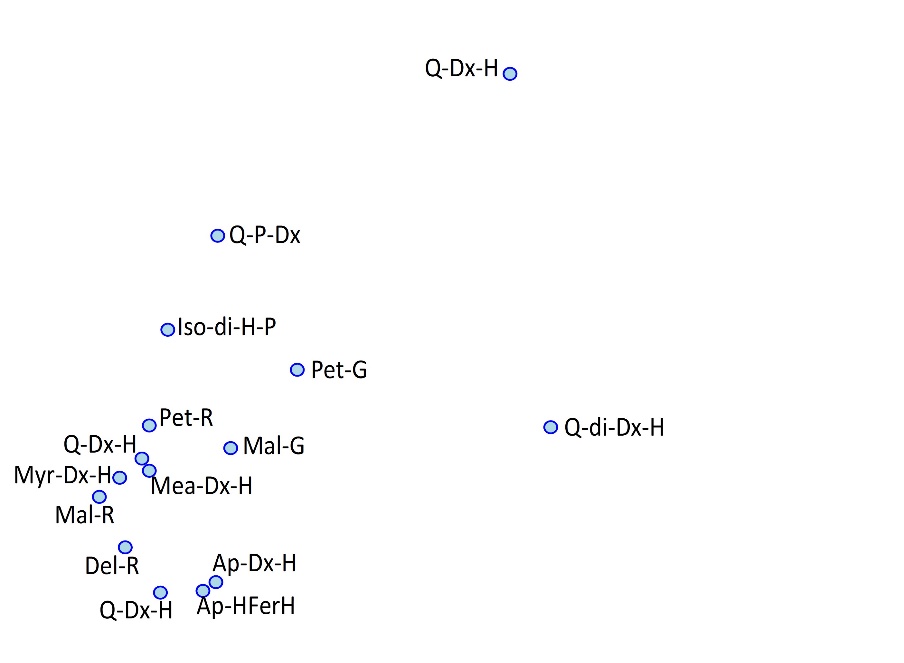


**B**

**A**


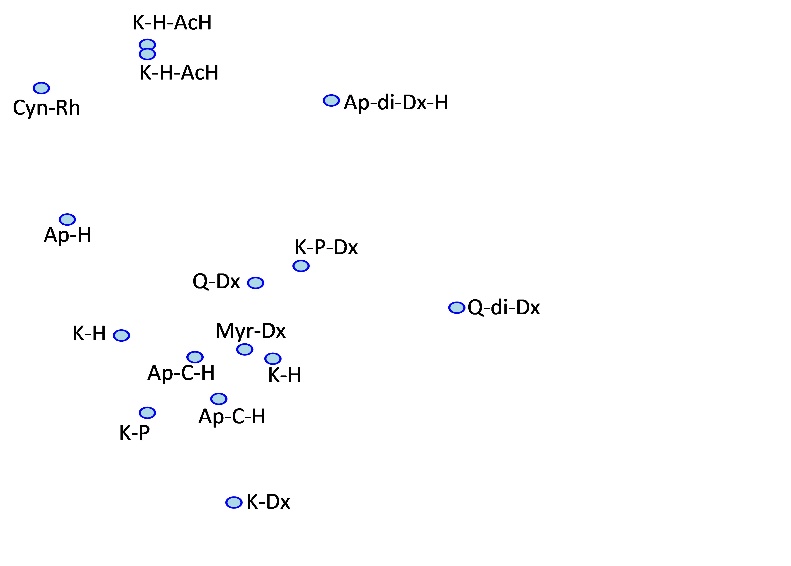


**Figure S3.** Principal component analysis (A) scores plots and (B) loadings plots of single-colored standard petals based upon their main color using identified flavonoids. Blue-colored dots refer to the flavonoids that are expected to be contributing the most to the different red shades of purple-red flowers. These flavonoids are written in bold in **Tables 2 and S1**. Cyn: cyanidin, Mal: malvidin, Pet: petunidin, K: kaempferol, Q: quercetin, Ap: apigenin, Iso: isorhamnetin, Myr: myricetin, Mea: mearnsetin, R: rutinoside, Dx: deoxyhexoside, H: hexoside, P: pentoside, Rh: rhamnoside, AcH: acetyl hexoside, HFerH: hydroxyferuloyl hexoside, C-H: C-hexoside, G: glucoside.


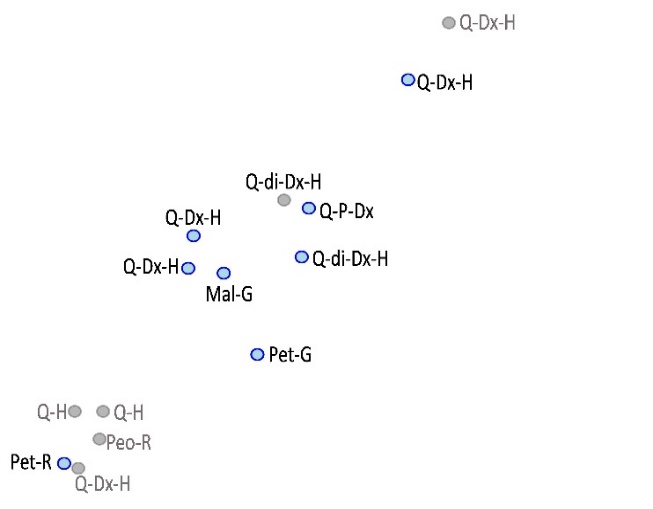


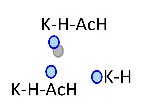

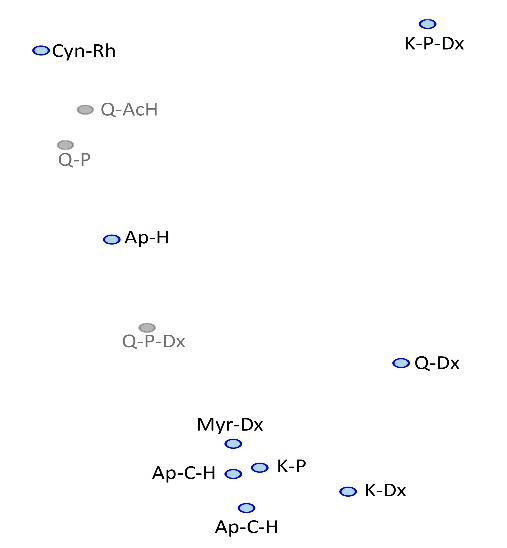


K-P-Dx

Del-R

Mal-R

Mea-Dx-H

Ap-Dx-H

Ap-HFerH

Cyn-Rh

K-H

Iso-P-Di-Dx

Myr-Dx-H

Ap-Di-Dx-H

Q-Di-Dx

**B**

**A**

**Figure S4.** Principal component analysis (A) scores plots and (B) loadings plots of single-colored wing petals based upon their main color using identified flavonoids. Blue-colored dots refer to the flavonoids identified in **Figure S3**. Flavonoids that are expected to be contributing the most to different shades of purplish red standard flower petals are indicated in the dashed boxes. These flavonoids are written in bold in **Tables 2 and S1**. Cyn: cyanidin, Mal: malvidin, Pet: petunidin, K: kaempferol, Q: quercetin, Ap: apigenin, Iso: isorhamnetin, Myr: myricetin, Mea: mearnsetin, R: rutinoside, Dx: deoxyhexoside, H: hexoside, P: pentoside, Rh: rhamnoside, AcH: acetyl hexoside, HFerH: hydroxyferuloyl hexoside, C-H: C-hexoside, G: glucoside.


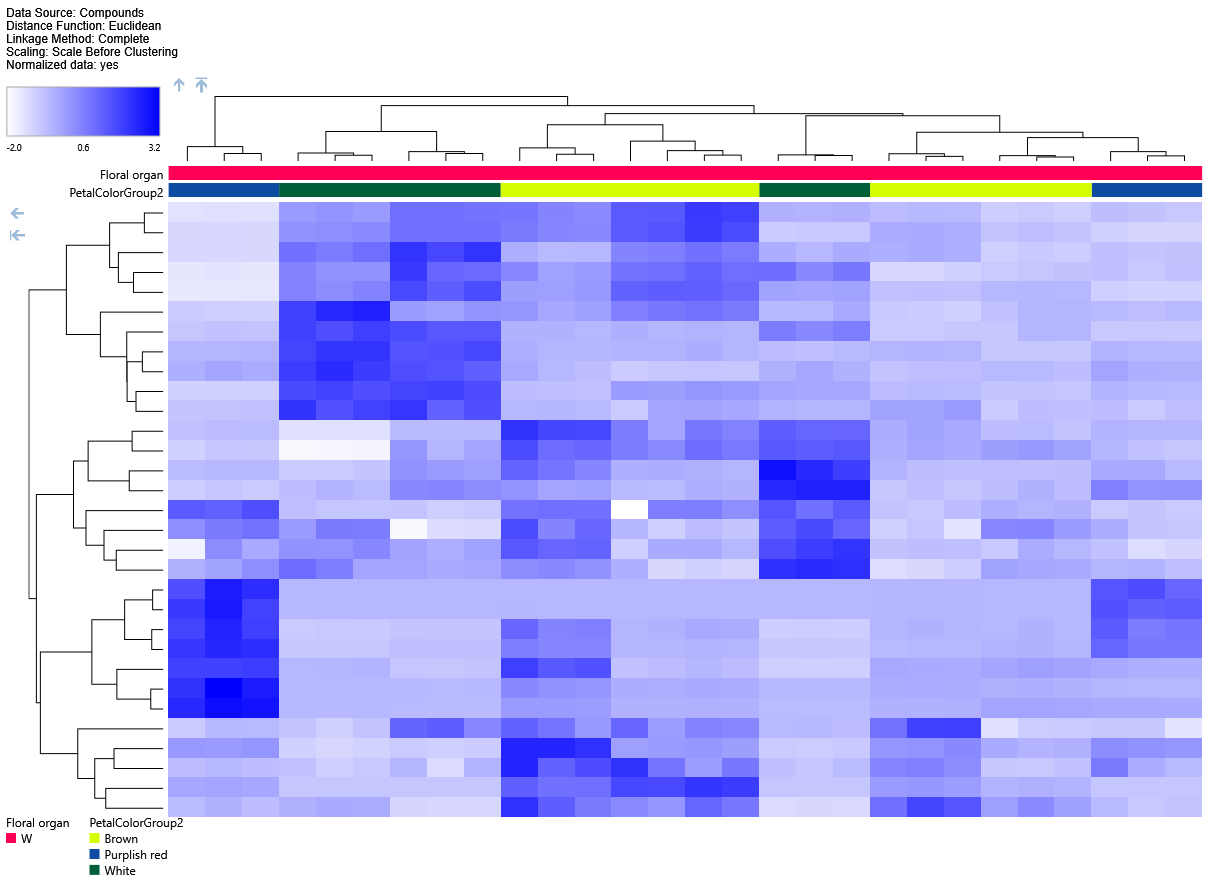


K-Dx-H

K-Dx-MalH

K-Dx-AcH

K-Dx-H

K-Dx

K-P-Dx

K-di-Dx-H

K-Dx-H

K-Dx-H

K-Dx-AcH

K-Dx-di-H

K-di-Dx-H

K-di-Dx-AcH

K-di-Dx-H

K-di-Dx-AcH

K-tri-Dx-H

K-P-di-Dx

K-di-Dx-H

K-tri-Dx-H

K-H-AcH

K-H-AcH

K-H

K-H

K-P-Dx

K-Dx

K-P

K-di-Dx-di-H

K-Dx-H

K-Dx-H

K-di-Dx

K-di-Dx-H

**A**

**PurR1 W B W B PurR2**


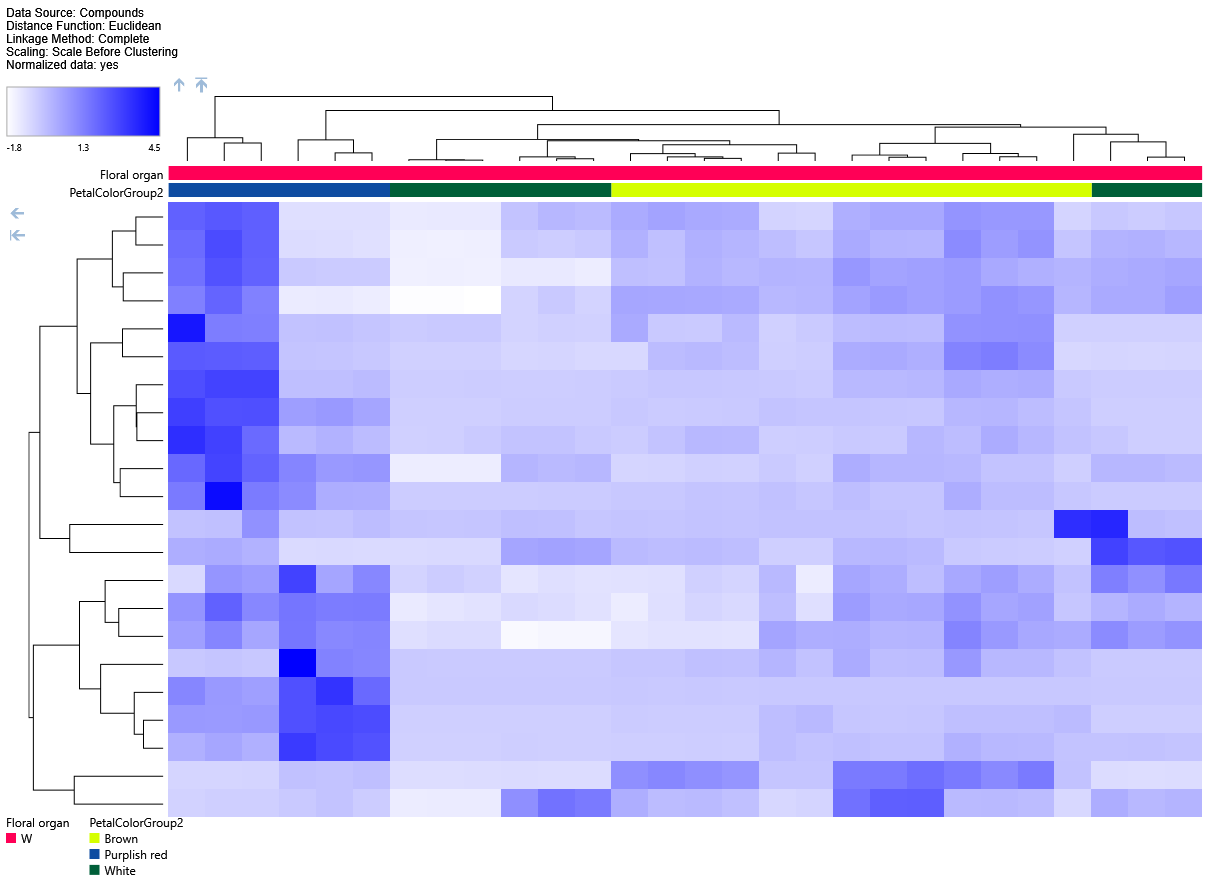


**PurR2 PurR1 W B W**

Q-Dx-H

Q-Dx-H

Q-P-Dx

Q-Dx

Q-di-Dx-H

Q-di-Dx-H

Q-Dx-H

Q-H

Q-Dx-H

Q-Dx-H

Q-H

Q-di-Dx-H

Q-Dx-AcH

Q-tri-Dx-H

Q-di-Dx-H

Q-P-Dx-H

Q-Dx

Q-AcH

Q-P

Q-P-Dx

Q-di-Dx

Q-Dx-di-H

**B**


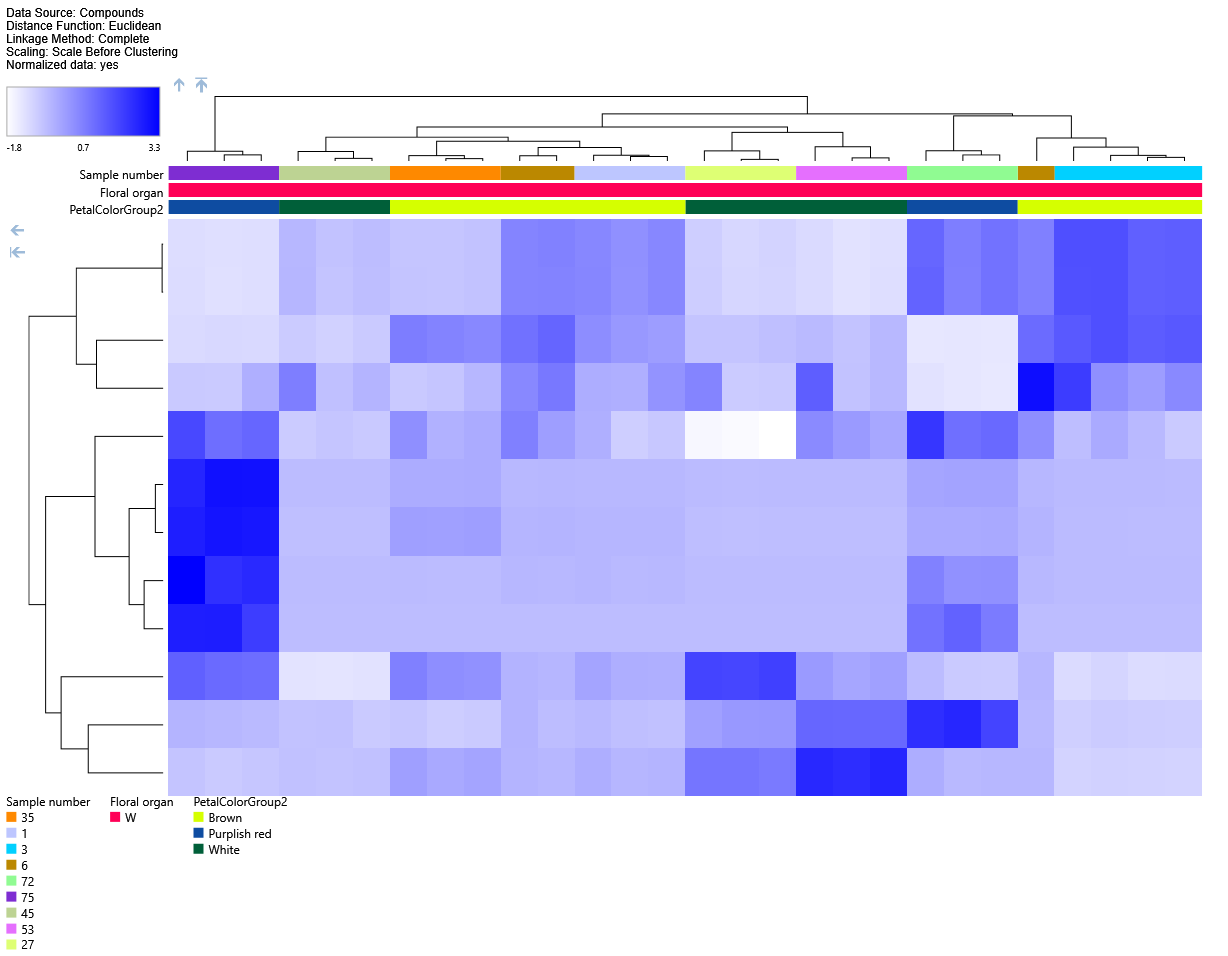


Ap-Dx-H

Ap-HFerH

Ap-HFerDx-H

Ap-Dx-H-AcH

Ap-Dx-H

Ap-C-H

Ap-C-H

Ap -H

T-AcH

Ap-di-Dx-H

Ap-Dx-H

Ap-Dx-HFerH

**C**

**PurR1 W B W PurR2 B**

**Figure S5.** Hierarchal clustering analysis (HCA) plot of single-colored faba bean wing flower petal groups (B: brown, PurR: purplish red, R: red and W: white) using identified glycosylated kaempferol (A), quercetin (B) and flavone (C) derivatives. Each rectangle represents a metabolite, and its color intensity refers to the relative amount of that specific metabolite in a specific sample. The areas were scaled before clustering by applying a z-score transformation and the heat map legend displays the range of the z-score values. K: kaempferol, Q: quercetin, Ap: apigenin, T: tricetin, Dx: deoxyhexoside, H: hexoside, P: pentoside, AcH: acetyl hexoside, MalH: malonyl hexoside, HFerH: hydroxyferuloyl hexoside.

A-Dx-H-AcH

A-HFerDx-H

A-di-Dx-H

T-AcH

A-H

A-C-H

A-C-H

A-Dx-HFerH

A-Dx-H

A-Dx-H

A-Dx-H

A-HFerH

**A**

**B**

Figure S6. Principal component analysis of flavonoids in (A) pure white petals and mixed color petals containing white, and (B) pure color petals and non-white mixed color petals. Dashed oval shapes refer to clusters of pure colored petals. ST: standard, W: wing petals.

**A**

**B**

**Figure S7.** Principal componenet analysis scores plot showing the observed variation in OC data (tight clusters) compared to flower samples (A) using anthocyanins and (B) flavonoids identified.

**Table S1.** Identification of non-anthocyanin flavonoid metabolites absorbing light in the visible region (380-680 nm) detected in faba bean flowers. An identification level of isomeric (2/3) was used to illustrate the glycosylation. Non-anthocyanin flavonoids written in bold italic contribute to the different red shades of purple-red standard flower petals (**Figure S3**).

| **Name** | **Formula** | **Calc. Molecular weight** | **RT [min]** | **Mass error [ppm]** |
| --- | --- | --- | --- | --- |
| **Dihydroflavonols** | | | | |
| Dihydromyricetin deoxyhexoside | C_21_ H_22_ O_12_ | 466.1112 | 8.57 | 0.09 |
| Dihydrokaempferol hexoside | C_21_ H_22_ O_11_ | 450.1168 | 8.90 | 1.25 |
| Dihydroquercetin hexoside | C_21_ H_22_ O_12_ | 466.1112 | 11.00 | 0.25 |
| Unidentified (ID level 4) |  | 466.2786 | 12.77 |  |
| Unidentified (ID level 4) |  | 552.2788 | 13.85 |  |
| **Flavones** |  |  |  |  |
| Tricetin acetyl hexoside | C24 H26 O12 | 506.1425 | 10.25 | 0.20 |
| Apigenin hexoside deoxyhexoside acetyl hexoside | C34 H42 O21 | 786.2222 | 10.32 | 0.45 |
| Apigenin hydroxyferuloyl hexoside deoxyhexoside | C40 H52 O24 | 916.2863 | 10.97 | 1.55 |
| ***Apigenin C-hexoside*** | C21 H20 O10 | 432.1056 | 12.05 | -0.07 |
| ***Apigenin C-hexoside*** | C21 H20 O10 | 432.1056 | 12.28 | -0.11 |
| Apigenin hydroxyferuloyl deoxyhexoside hexoside | C34 H42 O20 | 770.2273 | 12.63 | 0.49 |
| ***Apigenin di-deoxyhexoside hexoside*** | C33 H40 O18 | 724.2228 | 12.83 | 1.82 |
| ***Apigenin deoxyhexoside hexoside*** | C27 H30 O14 | 578.164 | 12.99 | 0.7 |
| ***Apigenin hydroxyferuloyl hexoside*** | C28 H32 O16 | 624.1695 | 13.01 | 0.78 |
| Apigenin deoxyhexoside hexoside | C27 H30 O14 | 578.1636 | 14.05 | 0.12 |
| Apigenin deoxyhexoside hexoside | C27 H30 O14 | 578.1637 | 14.29 | 0.16 |
| ***Apigenin hexoside*** | C21 H20 O10 | 432.1056 | 15.19 | -0.23 |
| **Flavonols (kaempferol derivatives)** |  |  |  |  |
| Kaempferol di-hexoside di-deoxyhexoside | C39 H50 O24 | 902.2694 | 8.36 | 0.25 |
| Kaempferol di-hexoside deoxyhexoside | C33 H40 O20 | 756.2113 | 9.68 | -0.03 |
| ***Kaempferol hexoside acetyl hexoside*** | C30 H36 O16 | 652.2002 | 9.82 | -0.14 |
| Kaempferol hexoside tri-deoxyhexoside | C39 H50 O23 | 886.2739 | 10.16 | -0.42 |
| Kaempferol hexoside tri-deoxyhexoside | C39 H50 O23 | 886.2741 | 10.32 | -0.18 |
| Kaempferol hexoside di-deoxyhexoside | C33 H40 O19 | 740.2163 | 10.38 | -0.08 |
| ***Kaempferol hexoside acetyl hexoside*** | C30 H36 O16 | 652.2004 | 10.39 | 0.11 |
| Kaempferol hexoside di-deoxyhexoside | C33 H40 O19 | 740.2163 | 10.57 | -0.09 |
| Kaempferol hexoside di-deoxyhexoside | C33 H40 O19 | 740.2178 | 10.65 | 1.92 |
| Kaempferol di-deoxyhexoside hexoside | C33 H40 O19 | 740.2162 | 11.69 | -0.26 |
| Kaempferol deoxyhexoside hexoside | C27 H30 O15 | 594.1582 | 11.74 | -0.46 |
| Kaempferol pentoside di-deoxyhexoside | C32 H38 O18 | 710.2058 | 11.84 | -0.03 |
| Kaempferol hexoside di-deoxyhexoside | C33 H40 O19 | 740.2161 | 11.96 | -0.35 |
| Kaempferol deoxyhexoside hexoside | C27 H30 O15 | 594.1582 | 12.09 | -0.48 |
| Kaempferol acetyl hexoside di-deoxyhexoside | C35 H42 O20 | 782.2276 | 12.23 | 0.86 |
| Kaempferol pentoside deoxyhexoside | C26 H28 O14 | 564.1478 | 12.32 | -0.24 |
| Kaempferol di-deoxyhexoside acetyl hexoside | C35 H42 O20 | 782.2275 | 12.40 | 0.72 |
| Kaempferol deoxyhexoside hexoside | C27 H30 O15 | 594.1582 | 12.50 | -0.51 |
| Kaempferol deoxyhexoside hexoside | C27 H30 O15 | 594.1582 | 12.70 | -0.49 |
| Kaempferol deoxyhexoside hexoside | C27 H30 O15 | 594.1581 | 13.07 | -0.58 |
| Kaempferol di-deoxyhexoside | C27 H30 O14 | 578.1636 | 13.24 | 0.01 |
| Kaempferol deoxyhexoside malonyl hexoside | C30 H32 O18 | 680.1602 | 13.35 | 2.02 |
| Kaempferol deoxyhexoside acetyl hexoside | C29 H32 O16 | 636.1694 | 13.45 | 0.64 |
| Kaempferol deoxyhexoside hexoside | C27 H30 O15 | 594.1582 | 13.53 | -0.52 |
| ***Kaempferol hexoside*** | C21 H20 O11 | 448.1005 | 13.58 | -0.07 |
| Kaempferol deoxyhexoside acetyl hexoside | C29 H32 O16 | 636.1694 | 13.60 | 0.61 |
| ***Kaempferol hexoside*** | C21 H20 O11 | 448.1006 | 13.95 | 0.1 |
| ***Kaempferol pentoside deoxyhexoside*** | C26 H28 O14 | 564.1483 | 13.96 | 0.75 |
| ***Kaempferol pentoside*** | C20 H18 O10 | 418.0905 | 14.43 | 1.2 |
| ***Kaempferol deoxyhexoside*** | C21 H20 O10 | 432.1055 | 15.37 | -0.33 |
| Kaempferol deoxyhexoside | C21 H20 O10 | 432.106 | 17.85 | 0.72 |
| **Flavonols (Quercetin derivatives)** |  |  |  |  |
| Quercetin di-hexoside deoxyhexoside | C33 H40 O21 | 772.2071 | 8.93 | 1.14 |
| Quercetin hexoside tri-deoxyhexoside | C39 H50 O24 | 902.2689 | 9.72 | -0.39 |
| ***Quercetin hexoside di-deoxyhexoside*** | C33 H40 O20 | 756.211 | 9.91 | -0.37 |
| Quercetin hexoside di-deoxyhexoside | C33 H40 O20 | 756.2114 | 10.02 | 0.19 |
| Quercetin acetyl hexoside | C24 H26 O12 | 506.1425 | 10.47 | 0.16 |
| Quercetin hexoside di-deoxyhexoside | C33 H40 O20 | 756.2108 | 11.15 | -0.61 |
| ***Quercetin deoxyhexoside hexoside*** | C27 H30 O16 | 610.1532 | 11.16 | -0.34 |
| Quercetin hexoside di-deoxyhexoside | C33 H40 O20 | 756.211 | 11.26 | -0.39 |
| Quercetin pentoside deoxyhexoside hexoside | C32 H38 O19 | 726.2004 | 11.27 | -0.4 |
| Quercetin deoxyhexoside hexoside | C27 H30 O16 | 610.1532 | 11.32 | -0.38 |
| ***Quercetin pentoside deoxyhexoside*** | C26 H28 O15 | 580.1428 | 11.78 | -0.02 |
| ***Quercetin deoxyhexoside hexoside*** | C27 H30 O16 | 610.1529 | 11.81 | -0.88 |
| ***Quercetin deoxyhexoside hexoside*** | C27 H30 O16 | 610.153 | 11.95 | -0.65 |
| ***Quercetin di-dexyhexoside*** | C27 H30 O15 | 594.1582 | 12.29 | -0.43 |
| Quercetin deoxyhexoside hexoside | C27 H30 O16 | 610.1531 | 12.58 | -0.45 |
| Quercetin deoxyhexoside acetyl hexoside | C29 H32 O17 | 652.1653 | 12.78 | 2.03 |
| Quercetin hexoside | C21 H20 O12 | 464.0957 | 12.87 | 0.58 |
| Quercetin hexoside | C21 H20 O12 | 464.0957 | 12.99 | 0.56 |
| Quercetin pentoside deoxyhexoside | C26 H28 O15 | 580.1429 | 13.16 | 0.13 |
| Quercetin pentoside | C20 H18 O11 | 434.085 | 13.71 | 0.19 |
| ***Quercetin deoxyhexoside*** | C21 H20 O11 | 448.1005 | 14.15 | -0.21 |
| Quercetin deoxyhexoside | C21 H20 O11 | 448.1009 | 16.22 | 0.71 |
| **Other Flavonols** | | | | |
| ***Isorhamnetin di-hexoside pentoside*** | C33 H40 O21 | 772.2068 | 10.63 | 0.71 |
| Isorhamnetin hexoside acetyl hexoside | C31 H38 O17 | 682.2117 | 10.75 | 1.21 |
| Isorhamnetin di-hexoside | C28 H32 O17 | 640.1637 | 11.30 | -0.48 |
| ***Myricetin deoxyhexoside hexoside*** | C27 H30 O17 | 626.1489 | 11.57 | 0.87 |
| Isorhamnetin di-dexoyhexoside hexoside | C34 H42 O20 | 770.2273 | 12.12 | 0.43 |
| Isorhamnetin di-deoxyhexoside hexoside | C34 H42 O20 | 770.2272 | 12.35 | 0.34 |
| ***Myricetin deoxyhexoside*** | C21 H20 O12 | 464.0957 | 12.74 | 0.37 |
| ***Mearnsetin hexoside deoxyhexoside*** | C28 H32 O17 | 640.1644 | 12.94 | 0.73 |
| Isorhamnetin deoxyhexoside hexoside | C28 H32 O16 | 624.1695 | 13.88 | 0.67 |
| Syringetin hexoside deoxyhexoside | C29 H34 O17 | 654.1808 | 14.08 | 1.84 |

**Summary S1.** Parameters used in generating the analysis of colored faba bean flower samples using the Compound Discoverer software.

## Workflow

Search name: UntarMetomics_Statistics Detect Unknowns with ID_24Jun2022_CD3.2_QC-RSD50_MaxCand50_ShiftRefFalse_16DB_MinPeak6M_35eV_NoPartents

Search description: Untargeted Metabolomics workflow: Find and identify the differences between samples.

- Performs retention time alignment, unknown compound detection, and compound grouping across all samples. Predicts elemental compositions for all compounds, fills gaps across all samples, and hides chemical background (using Blank samples). Identifies compounds using mzCloud (ddMS2) and ChemSpider (formula or exact mass). Also performs similarity search for all compounds with ddMS2 data using mzCloud. Applies mzLogic algorithm to rank order ChemSpider results. Maps compounds to biological pathways using Metabolika. Applies QC-based batch normalization if QC samples are available. Calculates differential analysis (t-test or ANOVA), determines p-values, adjusted p-values, ratios, fold change, CV, etc.).

Search date: 6/24/2022 11:09:25 PM

Created with Discoverer version: 3.2.0.421

[Input Files (6)]

-->Select Spectra (33)

[Select Spectra (33)]

-->Align Retention Times (26)

[Align Retention Times (26)]

-->Detect Compounds (9)

[Detect Compounds (9)]

-->Group Compounds (31)

[Group Compounds (31)]

-->Fill Gaps (32)

-->Search ChemSpider (23)

-->Map to Metabolika Pathways (34)

-->Search Mass Lists (36)

-->Predict Compositions (29)

-->Assign Compound Annotations (25)

-->Search mzCloud (22)

-->Search mzVault (37)

[Fill Gaps (32)]

-->Apply SERRF QC Correction [beta] (39)

[Apply SERRF QC Correction [beta] (39)]

-->Mark Background Compounds (40)

[Search ChemSpider (23)]

-->Apply mzLogic (35)

[Map to Metabolika Pathways (34)]

-->Apply mzLogic (35)

[Search Mass Lists (36)]

-->Apply mzLogic (35)

[Mark Background Compounds (40)]

[Apply mzLogic (35)]

[Predict Compositions (29)]

[Assign Compound Annotations (25)]

[Search mzCloud (22)]

[Search mzVault (37)]

[Differential Analysis (17)]

[Descriptive Statistics (38)]

------------------------------------------------------------------

Processing node 6: Input Files

------------------------------------------------------------------

Input Data:

- File Name(s) (Hidden):

E:\QE Data\2019MAR12_Jessa\190312QE_001.raw

E:\QE Data\2019MAR12_Jessa\190312QE_002.raw

E:\QE Data\2019MAR12_Jessa\190312QE_005.raw

E:\QE Data\2019MAR12_Jessa\190312QE_006.raw

E:\QE Data\2019MAR12_Jessa\190312QE_007.raw

E:\QE Data\2019MAR12_Jessa\190312QE_008.raw

E:\QE Data\2019MAR12_Jessa\190312QE_009.raw

E:\QE Data\2019MAR12_Jessa\190312QE_010.raw

E:\QE Data\2019MAR12_Jessa\190312QE_011.raw

E:\QE Data\2019MAR12_Jessa\190312QE_012.raw

E:\QE Data\2019MAR12_Jessa\190312QE_013.raw

E:\QE Data\2019MAR12_Jessa\190312QE_014.raw

E:\QE Data\2019MAR12_Jessa\190312QE_016.raw

E:\QE Data\2019MAR12_Jessa\190312QE_017.raw

E:\QE Data\2019MAR12_Jessa\190312QE_020.raw

E:\QE Data\2019MAR12_Jessa\190312QE_021.raw

E:\QE Data\2019MAR12_Jessa\190312QE_022.raw

E:\QE Data\2019MAR12_Jessa\190312QE_023.raw

E:\QE Data\2019MAR12_Jessa\190312QE_024.raw

E:\QE Data\2019MAR12_Jessa\190312QE_025.raw

E:\QE Data\2019MAR12_Jessa\190312QE_026.raw

E:\QE Data\2019MAR12_Jessa\190312QE_028.raw

E:\QE Data\2019MAR12_Jessa\190312QE_029.raw

E:\QE Data\2019MAR12_Jessa\190312QE_033.raw

E:\QE Data\2019MAR12_Jessa\190312QE_034.raw

E:\QE Data\2019MAR12_Jessa\190312QE_035.raw

E:\QE Data\2019MAR12_Jessa\190312QE_036.raw

E:\QE Data\2019MAR12_Jessa\190312QE_037.raw

E:\QE Data\2019MAR12_Jessa\190312QE_038.raw

E:\QE Data\2019MAR12_Jessa\190312QE_039.raw

E:\QE Data\2019MAR12_Jessa\190312QE_040.raw

E:\QE Data\2019MAR12_Jessa\190312QE_041.raw

E:\QE Data\2019MAR12_Jessa\190312QE_042.raw

E:\QE Data\2019MAR12_Jessa\190312QE_043.raw

E:\QE Data\2019MAR12_Jessa\190312QE_044.raw

E:\QE Data\2019MAR12_Jessa\190312QE_045.raw

E:\QE Data\2019MAR12_Jessa\190312QE_046.raw

E:\QE Data\2019MAR12_Jessa\190312QE_049.raw

E:\QE Data\2019MAR12_Jessa\190312QE_050.raw

E:\QE Data\2019MAR12_Jessa\190312QE_051.raw

E:\QE Data\2019MAR12_Jessa\190312QE_052.raw

E:\QE Data\2019MAR12_Jessa\190312QE_053.raw

E:\QE Data\2019MAR12_Jessa\190312QE_054.raw

E:\QE Data\2019MAR12_Jessa\190312QE_055.raw

E:\QE Data\2019MAR12_Jessa\190312QE_057.raw

E:\QE Data\2019MAR12_Jessa\190312QE_058.raw

E:\QE Data\2019MAR12_Jessa\190312QE_059.raw

E:\QE Data\2019MAR12_Jessa\190312QE_060.raw

E:\QE Data\2019MAR12_Jessa\190312QE_061.raw

E:\QE Data\2019MAR12_Jessa\190312QE_066.raw

E:\QE Data\2019MAR12_Jessa\190312QE_067.raw

E:\QE Data\2019MAR12_Jessa\190312QE_068.raw

E:\QE Data\2019MAR12_Jessa\190312QE_069.raw

E:\QE Data\2019MAR12_Jessa\190312QE_070.raw

E:\QE Data\2019MAR12_Jessa\190312QE_071.raw

E:\QE Data\2019MAR12_Jessa\190312QE_072.raw

E:\QE Data\2019MAR12_Jessa\190312QE_075.raw

E:\QE Data\2019MAR12_Jessa\190312QE_076.raw

E:\QE Data\2019MAR12_Jessa\190312QE_077.raw

E:\QE Data\2019MAR12_Jessa\190312QE_078.raw

E:\QE Data\2019MAR12_Jessa\190312QE_080.raw

E:\QE Data\2019MAR12_Jessa\190312QE_081.raw

E:\QE Data\2019MAR12_Jessa\190312QE_082.raw

E:\QE Data\2019MAR12_Jessa\190312QE_083.raw

E:\QE Data\2019MAR12_Jessa\190312QE_084.raw

E:\QE Data\2019MAR12_Jessa\190312QE_085.raw

E:\QE Data\2019MAR12_Jessa\190312QE_086.raw

E:\QE Data\2019MAR12_Jessa\190312QE_087.raw

E:\QE Data\2019MAR12_Jessa\190312QE_088.raw

E:\QE Data\2019MAR12_Jessa\190312QE_089.raw

E:\QE Data\2019MAR12_Jessa\190312QE_090.raw

E:\QE Data\2019MAR12_Jessa\190312QE_091.raw

E:\QE Data\2019MAR12_Jessa\190312QE_092.raw

E:\QE Data\2019MAR12_Jessa\190312QE_093.raw

E:\QE Data\2019MAR12_Jessa\190312QE_094.raw

E:\QE Data\2019MAR12_Jessa\190312QE_095.raw

E:\QE Data\2019MAR12_Jessa\190312QE_096.raw

E:\QE Data\2019MAR12_Jessa\190312QE_097.raw

E:\QE Data\2019MAR12_Jessa\190312QE_098.raw

E:\QE Data\2019MAR12_Jessa\190312QE_099.raw

E:\QE Data\2019MAR12_Jessa\190312QE_100.raw

E:\QE Data\2019MAR12_Jessa\190312QE_101.raw

E:\QE Data\2019MAR12_Jessa\190312QE_102.raw

E:\QE Data\2019MAR12_Jessa\190312QE_103.raw

E:\QE Data\2019MAR12_Jessa\190312QE_104.raw

E:\QE Data\2019MAR12_Jessa\190312QE_105.raw

E:\QE Data\2019MAR12_Jessa\190312QE_107.raw

E:\QE Data\2019MAR12_Jessa\190312QE_108.raw

E:\QE Data\2019MAR12_Jessa\190312QE_109.raw

E:\QE Data\2019MAR12_Jessa\190312QE_110.raw

E:\QE Data\2019MAR12_Jessa\190312QE_111.raw

E:\QE Data\2019MAR12_Jessa\190312QE_112.raw

E:\QE Data\2019MAR12_Jessa\190312QE_113.raw

E:\QE Data\2019MAR12_Jessa\190312QE_114.raw

E:\QE Data\2019MAR12_Jessa\190312QE_115.raw

E:\QE Data\2019MAR12_Jessa\190312QE_116.raw

E:\QE Data\2019MAR12_Jessa\190312QE_118.raw

E:\QE Data\2019MAR12_Jessa\190312QE_119.raw

E:\QE Data\2019MAR12_Jessa\190312QE_120.raw

E:\QE Data\2019MAR12_Jessa\190312QE_121.raw

E:\QE Data\2019MAR12_Jessa\190312QE_122.raw

E:\QE Data\2019MAR12_Jessa\190312QE_123.raw

E:\QE Data\2020Aug2_Jessa'sFlowers_SetB\2020Jul30_Jessa_2\2020Jul30_001.raw

E:\QE Data\2020Aug2_Jessa'sFlowers_SetB\2020Jul30_Jessa_2\2020Jul30_002.raw

E:\QE Data\2020Aug2_Jessa'sFlowers_SetB\2020Jul30_Jessa_2\2020Jul30_003.raw

E:\QE Data\2020Aug2_Jessa'sFlowers_SetB\2020Jul30_Jessa_2\2020Jul30_005.raw

E:\QE Data\2020Aug2_Jessa'sFlowers_SetB\2020Jul30_Jessa_2\2020Jul30_006.raw

E:\QE Data\2020Aug2_Jessa'sFlowers_SetB\2020Jul30_Jessa_2\2020Jul30_007.raw

E:\QE Data\2020Aug2_Jessa'sFlowers_SetB\2020Jul30_Jessa_2\2020Jul30_008.raw

E:\QE Data\2020Aug2_Jessa'sFlowers_SetB\2020Jul30_Jessa_2\2020Jul30_009.raw

E:\QE Data\2020Aug2_Jessa'sFlowers_SetB\2020Jul30_Jessa_2\2020Jul30_010.raw

E:\QE Data\2020Aug2_Jessa'sFlowers_SetB\2020Jul30_Jessa_2\2020Jul30_011.raw

E:\QE Data\2020Aug2_Jessa'sFlowers_SetB\2020Jul30_Jessa_2\2020Jul30_014.raw

E:\QE Data\2020Aug2_Jessa'sFlowers_SetB\2020Jul30_Jessa_2\2020Jul30_015.raw

E:\QE Data\2020Aug2_Jessa'sFlowers_SetB\2020Jul30_Jessa_2\2020Jul30_016.raw

E:\QE Data\2020Aug2_Jessa'sFlowers_SetB\2020Jul30_Jessa_2\2020Jul30_017.raw

E:\QE Data\2020Aug2_Jessa'sFlowers_SetB\2020Jul30_Jessa_2\2020Jul30_018.raw

E:\QE Data\2020Aug2_Jessa'sFlowers_SetB\2020Jul30_Jessa_2\2020Jul30_019.raw

E:\QE Data\2020Aug2_Jessa'sFlowers_SetB\2020Jul30_Jessa_2\2020Jul30_020.raw

E:\QE Data\2020Aug2_Jessa'sFlowers_SetB\2020Jul30_Jessa_2\2020Jul30_023.raw

E:\QE Data\2020Aug2_Jessa'sFlowers_SetB\2020Jul30_Jessa_2\2020Jul30_025.raw

E:\QE Data\2020Aug2_Jessa'sFlowers_SetB\2020Jul30_Jessa_2\2020Jul30_026.raw

E:\QE Data\2020Aug2_Jessa'sFlowers_SetB\2020Jul30_Jessa_2\2020Jul30_027.raw

E:\QE Data\2020Aug2_Jessa'sFlowers_SetB\2020Jul30_Jessa_2\2020Jul30_028.raw

E:\QE Data\2020Aug2_Jessa'sFlowers_SetB\2020Jul30_Jessa_2\2020Jul30_029.raw

E:\QE Data\2020Aug2_Jessa'sFlowers_SetB\2020Jul30_Jessa_2\2020Jul30_030.raw

E:\QE Data\2020Aug2_Jessa'sFlowers_SetB\2020Jul30_Jessa_2\2020Jul30_031.raw

E:\QE Data\2020Aug2_Jessa'sFlowers_SetB\2020Jul30_Jessa_2\2020Jul30_032.raw

E:\QE Data\2020Aug2_Jessa'sFlowers_SetB\2020Jul30_Jessa_2\2020Jul30_033.raw

E:\QE Data\2020Aug2_Jessa'sFlowers_SetB\2020Jul30_Jessa_2\2020Jul30_034.raw

E:\QE Data\2020Aug2_Jessa'sFlowers_SetB\2020Jul30_Jessa_2\2020Jul30_035.raw

E:\QE Data\2020Aug2_Jessa'sFlowers_SetB\2020Jul30_Jessa_2\2020Jul30_036.raw

E:\QE Data\2020Aug2_Jessa'sFlowers_SetB\2020Jul30_Jessa_2\2020Jul30_037.raw

E:\QE Data\2020Aug2_Jessa'sFlowers_SetB\2020Jul30_Jessa_2\2020Jul30_038.raw

E:\QE Data\2020Aug2_Jessa'sFlowers_SetB\2020Jul30_Jessa_2\2020Jul30_039.raw

E:\QE Data\2020Aug2_Jessa'sFlowers_SetB\2020Jul30_Jessa_2\2020Jul30_040.raw

E:\QE Data\2020Aug2_Jessa'sFlowers_SetB\2020Jul30_Jessa_2\2020Jul30_041.raw

E:\QE Data\2020Aug2_Jessa'sFlowers_SetB\2020Jul30_Jessa_2\2020Jul30_042.raw

E:\QE Data\2020Aug2_Jessa'sFlowers_SetB\2020Jul30_Jessa_2\2020Jul30_043.raw

E:\QE Data\2020Aug2_Jessa'sFlowers_SetB\2020Jul30_Jessa_2\2020Jul30_044.raw

E:\QE Data\2020Aug2_Jessa'sFlowers_SetB\2020Jul30_Jessa_2\2020Jul30_045.raw

E:\QE Data\2020Aug2_Jessa'sFlowers_SetB\2020Jul30_Jessa_2\2020Jul30_046.raw

E:\QE Data\2020Aug2_Jessa'sFlowers_SetB\2020Jul30_Jessa_2\2020Jul30_050.raw

E:\QE Data\2020Aug2_Jessa'sFlowers_SetB\2020Jul30_Jessa_2\2020Jul30_051.raw

E:\QE Data\2020Aug2_Jessa'sFlowers_SetB\2020Jul30_Jessa_2\2020Jul30_052.raw

E:\QE Data\2020Aug2_Jessa'sFlowers_SetB\2020Jul30_Jessa_2\2020Jul30_053.raw

E:\QE Data\2020Aug2_Jessa'sFlowers_SetB\2020Jul30_Jessa_2\2020Jul30_054.raw

E:\QE Data\2020Aug2_Jessa'sFlowers_SetB\2020Jul30_Jessa_2\2020Jul30_055.raw

E:\QE Data\2020Aug2_Jessa'sFlowers_SetB\2020Jul30_Jessa_2\2020Jul30_056.raw

E:\QE Data\2020Aug2_Jessa'sFlowers_SetB\2020Jul30_Jessa_2\2020Jul30_059.raw

E:\QE Data\2020Aug2_Jessa'sFlowers_SetB\2020Jul30_Jessa_2\2020Jul30_060.raw

E:\QE Data\2020Aug2_Jessa'sFlowers_SetB\2020Jul30_Jessa_2\2020Jul30_061.raw

E:\QE Data\2020Aug2_Jessa'sFlowers_SetB\2020Jul30_Jessa_2\2020Jul30_062.raw

E:\QE Data\2020Aug2_Jessa'sFlowers_SetB\2020Jul30_Jessa_2\2020Jul30_063.raw

E:\QE Data\2020Aug2_Jessa'sFlowers_SetB\2020Jul30_Jessa_2\2020Jul30_064.raw

E:\QE Data\2020Aug2_Jessa'sFlowers_SetB\2020Jul30_Jessa_2\2020Jul30_065.raw

E:\QE Data\2020Aug2_Jessa'sFlowers_SetB\2020Jul30_Jessa_2\2020Jul30_066.raw

E:\QE Data\2020Aug2_Jessa'sFlowers_SetB\2020Jul30_Jessa_2\2020Jul30_067.raw

E:\QE Data\2020Aug2_Jessa'sFlowers_SetB\2020Jul30_Jessa_2\2020Jul30_068.raw

E:\QE Data\2020Aug2_Jessa'sFlowers_SetB\2020Jul30_Jessa_2\2020Jul30_069.raw

E:\QE Data\2020Aug2_Jessa'sFlowers_SetB\2020Jul30_Jessa_2\2020Jul30_070.raw

E:\QE Data\2020Aug2_Jessa'sFlowers_SetB\2020Jul30_Jessa_2\2020Jul30_071.raw

E:\QE Data\2020Aug2_Jessa'sFlowers_SetB\2020Jul30_Jessa_2\2020Jul30_072.raw

E:\QE Data\2020Aug2_Jessa'sFlowers_SetB\2020Jul30_Jessa_2\2020Jul30_073.raw

E:\QE Data\2020Aug2_Jessa'sFlowers_SetB\2020Jul30_Jessa_2\2020Jul30_074.raw

E:\QE Data\2020Aug2_Jessa'sFlowers_SetB\2020Jul30_Jessa_2\2020Jul30_075.raw

E:\QE Data\2020Aug2_Jessa'sFlowers_SetB\2020Jul30_Jessa_2\2020Jul30_076.raw

E:\QE Data\2020Aug2_Jessa'sFlowers_SetB\2020Jul30_Jessa_2\2020Jul30_078.raw

E:\QE Data\2020Aug2_Jessa'sFlowers_SetB\2020Jul30_Jessa_2\2020Jul30_079.raw

E:\QE Data\2020Aug2_Jessa'sFlowers_SetB\2020Jul30_Jessa_2\2020Jul30_080.raw

E:\QE Data\2020Aug2_Jessa'sFlowers_SetB\2020Jul30_Jessa_2\2020Jul30_081.raw

E:\QE Data\2020Aug2_Jessa'sFlowers_SetB\2020Jul30_Jessa_2\2020Jul30_082.raw

E:\QE Data\2020Aug2_Jessa'sFlowers_SetB\2020Jul30_Jessa_2\2020Jul30_083.raw

E:\QE Data\2020Aug2_Jessa'sFlowers_SetB\2020Jul30_Jessa_2\2020Jul30_084.raw

E:\QE Data\2020Aug2_Jessa'sFlowers_SetB\2020Jul30_Jessa_2\2020Jul30_085.raw

E:\QE Data\2020Aug2_Jessa'sFlowers_SetB\2020Jul30_Jessa_2\2020Jul30_086.raw

E:\QE Data\2020Aug2_Jessa'sFlowers_SetB\2020Jul30_Jessa_2\2020Jul30_088.raw

E:\QE Data\2020Aug2_Jessa'sFlowers_SetB\2020Jul30_Jessa_2\2020Jul30_089.raw

E:\QE Data\2020Aug2_Jessa'sFlowers_SetB\2020Jul30_Jessa_2\2020Jul30_090.raw

E:\QE Data\2020Aug2_Jessa'sFlowers_SetB\2020Jul30_Jessa_2\2020Jul30_091.raw

E:\QE Data\2020Aug2_Jessa'sFlowers_SetB\2020Jul30_Jessa_2\2020Jul30_092.raw

E:\QE Data\2020Aug2_Jessa'sFlowers_SetB\2020Jul30_Jessa_2\2020Jul30_093.raw

E:\QE Data\2020Aug2_Jessa'sFlowers_SetB\2020Jul30_Jessa_2\2020Jul30_094.raw

E:\QE Data\2020Aug2_Jessa'sFlowers_SetB\2020Jul30_Jessa_2\2020Jul30_095.raw

E:\QE Data\2020Aug2_Jessa'sFlowers_SetB\2020Jul30_Jessa_2\2020Jul30_096.raw

E:\QE Data\2020Aug2_Jessa'sFlowers_SetB\2020Jul30_Jessa_2\2020Jul30_097.raw

E:\QE Data\2020Aug5_Jessa'sFlowers_SetC\2020Aug5_Jessa_3_Neg\2020Aug5_098.raw

E:\QE Data\2020Aug5_Jessa'sFlowers_SetC\2020Aug5_Jessa_3_Neg\2020Aug5_099.raw

E:\QE Data\2020Aug5_Jessa'sFlowers_SetC\2020Aug5_Jessa_3_Neg\2020Aug5_100.raw

E:\QE Data\2020Aug5_Jessa'sFlowers_SetC\2020Aug5_Jessa_3_Neg\2020Aug5_103.raw

E:\QE Data\2020Aug5_Jessa'sFlowers_SetC\2020Aug5_Jessa_3_Neg\2020Aug5_106.raw

E:\QE Data\2020Aug5_Jessa'sFlowers_SetC\2020Aug5_Jessa_3_Neg\2020Aug5_109.raw

E:\QE Data\2020Aug5_Jessa'sFlowers_SetC\2020Aug5_Jessa_3_Neg\2020Aug5_113.raw

E:\QE Data\2020Aug5_Jessa'sFlowers_SetC\2020Aug5_Jessa_3_Neg\2020Aug5_116.raw

E:\QE Data\2020Aug5_Jessa'sFlowers_SetC\2020Aug5_Jessa_3_Neg\2020Aug5_119.raw

E:\QE Data\2020Aug5_Jessa'sFlowers_SetC\2020Aug5_Jessa_3_Neg\2020Aug5_122.raw

E:\QE Data\2020Aug5_Jessa'sFlowers_SetC\2020Aug5_Jessa_3_Neg\2020Aug5_001.raw

E:\QE Data\2020Aug5_Jessa'sFlowers_SetC\2020Aug5_Jessa_3_Neg\2020Aug5_002.raw

E:\QE Data\2020Aug5_Jessa'sFlowers_SetC\2020Aug5_Jessa_3_Neg\2020Aug5_003.raw

E:\QE Data\2020Aug5_Jessa'sFlowers_SetC\2020Aug5_Jessa_3_Neg\2020Aug5_005.raw

E:\QE Data\2020Aug5_Jessa'sFlowers_SetC\2020Aug5_Jessa_3_Neg\2020Aug5_006.raw

E:\QE Data\2020Aug5_Jessa'sFlowers_SetC\2020Aug5_Jessa_3_Neg\2020Aug5_007.raw

E:\QE Data\2020Aug5_Jessa'sFlowers_SetC\2020Aug5_Jessa_3_Neg\2020Aug5_008.raw

E:\QE Data\2020Aug5_Jessa'sFlowers_SetC\2020Aug5_Jessa_3_Neg\2020Aug5_009.raw

E:\QE Data\2020Aug5_Jessa'sFlowers_SetC\2020Aug5_Jessa_3_Neg\2020Aug5_010.raw

E:\QE Data\2020Aug5_Jessa'sFlowers_SetC\2020Aug5_Jessa_3_Neg\2020Aug5_011.raw

E:\QE Data\2020Aug5_Jessa'sFlowers_SetC\2020Aug5_Jessa_3_Neg\2020Aug5_014.raw

E:\QE Data\2020Aug5_Jessa'sFlowers_SetC\2020Aug5_Jessa_3_Neg\2020Aug5_015.raw

E:\QE Data\2020Aug5_Jessa'sFlowers_SetC\2020Aug5_Jessa_3_Neg\2020Aug5_016.raw

E:\QE Data\2020Aug5_Jessa'sFlowers_SetC\2020Aug5_Jessa_3_Neg\2020Aug5_017.raw

E:\QE Data\2020Aug5_Jessa'sFlowers_SetC\2020Aug5_Jessa_3_Neg\2020Aug5_018.raw

E:\QE Data\2020Aug5_Jessa'sFlowers_SetC\2020Aug5_Jessa_3_Neg\2020Aug5_019.raw

E:\QE Data\2020Aug5_Jessa'sFlowers_SetC\2020Aug5_Jessa_3_Neg\2020Aug5_020.raw

E:\QE Data\2020Aug5_Jessa'sFlowers_SetC\2020Aug5_Jessa_3_Neg\2020Aug5_023.raw

E:\QE Data\2020Aug5_Jessa'sFlowers_SetC\2020Aug5_Jessa_3_Neg\2020Aug5_025.raw

E:\QE Data\2020Aug5_Jessa'sFlowers_SetC\2020Aug5_Jessa_3_Neg\2020Aug5_026.raw

E:\QE Data\2020Aug5_Jessa'sFlowers_SetC\2020Aug5_Jessa_3_Neg\2020Aug5_027.raw

E:\QE Data\2020Aug5_Jessa'sFlowers_SetC\2020Aug5_Jessa_3_Neg\2020Aug5_028.raw

E:\QE Data\2020Aug5_Jessa'sFlowers_SetC\2020Aug5_Jessa_3_Neg\2020Aug5_029.raw

E:\QE Data\2020Aug5_Jessa'sFlowers_SetC\2020Aug5_Jessa_3_Neg\2020Aug5_030.raw

E:\QE Data\2020Aug5_Jessa'sFlowers_SetC\2020Aug5_Jessa_3_Neg\2020Aug5_031.raw

E:\QE Data\2020Aug5_Jessa'sFlowers_SetC\2020Aug5_Jessa_3_Neg\2020Aug5_032.raw

E:\QE Data\2020Aug5_Jessa'sFlowers_SetC\2020Aug5_Jessa_3_Neg\2020Aug5_033.raw

E:\QE Data\2020Aug5_Jessa'sFlowers_SetC\2020Aug5_Jessa_3_Neg\2020Aug5_034.raw

E:\QE Data\2020Aug5_Jessa'sFlowers_SetC\2020Aug5_Jessa_3_Neg\2020Aug5_035.raw

E:\QE Data\2020Aug5_Jessa'sFlowers_SetC\2020Aug5_Jessa_3_Neg\2020Aug5_036.raw

E:\QE Data\2020Aug5_Jessa'sFlowers_SetC\2020Aug5_Jessa_3_Neg\2020Aug5_037.raw

E:\QE Data\2020Aug5_Jessa'sFlowers_SetC\2020Aug5_Jessa_3_Neg\2020Aug5_038.raw

E:\QE Data\2020Aug5_Jessa'sFlowers_SetC\2020Aug5_Jessa_3_Neg\2020Aug5_039.raw

E:\QE Data\2020Aug5_Jessa'sFlowers_SetC\2020Aug5_Jessa_3_Neg\2020Aug5_040.raw

E:\QE Data\2020Aug5_Jessa'sFlowers_SetC\2020Aug5_Jessa_3_Neg\2020Aug5_041.raw

E:\QE Data\2020Aug5_Jessa'sFlowers_SetC\2020Aug5_Jessa_3_Neg\2020Aug5_042.raw

E:\QE Data\2020Aug5_Jessa'sFlowers_SetC\2020Aug5_Jessa_3_Neg\2020Aug5_043.raw

E:\QE Data\2020Aug5_Jessa'sFlowers_SetC\2020Aug5_Jessa_3_Neg\2020Aug5_044.raw

E:\QE Data\2020Aug5_Jessa'sFlowers_SetC\2020Aug5_Jessa_3_Neg\2020Aug5_045.raw

E:\QE Data\2020Aug5_Jessa'sFlowers_SetC\2020Aug5_Jessa_3_Neg\2020Aug5_046.raw

E:\QE Data\2020Aug5_Jessa'sFlowers_SetC\2020Aug5_Jessa_3_Neg\2020Aug5_050.raw

E:\QE Data\2020Aug5_Jessa'sFlowers_SetC\2020Aug5_Jessa_3_Neg\2020Aug5_051.raw

E:\QE Data\2020Aug5_Jessa'sFlowers_SetC\2020Aug5_Jessa_3_Neg\2020Aug5_052.raw

E:\QE Data\2020Aug5_Jessa'sFlowers_SetC\2020Aug5_Jessa_3_Neg\2020Aug5_053.raw

E:\QE Data\2020Aug5_Jessa'sFlowers_SetC\2020Aug5_Jessa_3_Neg\2020Aug5_054.raw

E:\QE Data\2020Aug5_Jessa'sFlowers_SetC\2020Aug5_Jessa_3_Neg\2020Aug5_055.raw

E:\QE Data\2020Aug5_Jessa'sFlowers_SetC\2020Aug5_Jessa_3_Neg\2020Aug5_056.raw

E:\QE Data\2020Aug5_Jessa'sFlowers_SetC\2020Aug5_Jessa_3_Neg\2020Aug5_059.raw

E:\QE Data\2020Aug5_Jessa'sFlowers_SetC\2020Aug5_Jessa_3_Neg\2020Aug5_060.raw

E:\QE Data\2020Aug5_Jessa'sFlowers_SetC\2020Aug5_Jessa_3_Neg\2020Aug5_061.raw

E:\QE Data\2020Aug5_Jessa'sFlowers_SetC\2020Aug5_Jessa_3_Neg\2020Aug5_062.raw

E:\QE Data\2020Aug5_Jessa'sFlowers_SetC\2020Aug5_Jessa_3_Neg\2020Aug5_063.raw

E:\QE Data\2020Aug5_Jessa'sFlowers_SetC\2020Aug5_Jessa_3_Neg\2020Aug5_064.raw

E:\QE Data\2020Aug5_Jessa'sFlowers_SetC\2020Aug5_Jessa_3_Neg\2020Aug5_065.raw

E:\QE Data\2020Aug5_Jessa'sFlowers_SetC\2020Aug5_Jessa_3_Neg\2020Aug5_066.raw

E:\QE Data\2020Aug5_Jessa'sFlowers_SetC\2020Aug5_Jessa_3_Neg\2020Aug5_067.raw

E:\QE Data\2020Aug5_Jessa'sFlowers_SetC\2020Aug5_Jessa_3_Neg\2020Aug5_068.raw

E:\QE Data\2020Aug5_Jessa'sFlowers_SetC\2020Aug5_Jessa_3_Neg\2020Aug5_069.raw

E:\QE Data\2020Aug5_Jessa'sFlowers_SetC\2020Aug5_Jessa_3_Neg\2020Aug5_070.raw

E:\QE Data\2020Aug5_Jessa'sFlowers_SetC\2020Aug5_Jessa_3_Neg\2020Aug5_071.raw

E:\QE Data\2020Aug5_Jessa'sFlowers_SetC\2020Aug5_Jessa_3_Neg\2020Aug5_072.raw

E:\QE Data\2020Aug5_Jessa'sFlowers_SetC\2020Aug5_Jessa_3_Neg\2020Aug5_073.raw

E:\QE Data\2020Aug5_Jessa'sFlowers_SetC\2020Aug5_Jessa_3_Neg\2020Aug5_074.raw

E:\QE Data\2020Aug5_Jessa'sFlowers_SetC\2020Aug5_Jessa_3_Neg\2020Aug5_075.raw

E:\QE Data\2020Aug5_Jessa'sFlowers_SetC\2020Aug5_Jessa_3_Neg\2020Aug5_076.raw

E:\QE Data\2020Aug5_Jessa'sFlowers_SetC\2020Aug5_Jessa_3_Neg\2020Aug5_078.raw

E:\QE Data\2020Aug5_Jessa'sFlowers_SetC\2020Aug5_Jessa_3_Neg\2020Aug5_079.raw

E:\QE Data\2020Aug5_Jessa'sFlowers_SetC\2020Aug5_Jessa_3_Neg\2020Aug5_080.raw

E:\QE Data\2020Aug5_Jessa'sFlowers_SetC\2020Aug5_Jessa_3_Neg\2020Aug5_081.raw

E:\QE Data\2020Aug5_Jessa'sFlowers_SetC\2020Aug5_Jessa_3_Neg\2020Aug5_082.raw

E:\QE Data\2020Aug5_Jessa'sFlowers_SetC\2020Aug5_Jessa_3_Neg\2020Aug5_083.raw

E:\QE Data\2020Aug5_Jessa'sFlowers_SetC\2020Aug5_Jessa_3_Neg\2020Aug5_084.raw

E:\QE Data\2020Aug5_Jessa'sFlowers_SetC\2020Aug5_Jessa_3_Neg\2020Aug5_085.raw

E:\QE Data\2020Aug5_Jessa'sFlowers_SetC\2020Aug5_Jessa_3_Neg\2020Aug5_086.raw

E:\QE Data\2020Aug5_Jessa'sFlowers_SetC\2020Aug5_Jessa_3_Neg\2020Aug5_088.raw

E:\QE Data\2020Aug5_Jessa'sFlowers_SetC\2020Aug5_Jessa_3_Neg\2020Aug5_089.raw

E:\QE Data\2020Aug5_Jessa'sFlowers_SetC\2020Aug5_Jessa_3_Neg\2020Aug5_090.raw

E:\QE Data\2020Aug5_Jessa'sFlowers_SetC\2020Aug5_Jessa_3_Neg\2020Aug5_091.raw

E:\QE Data\2020Aug5_Jessa'sFlowers_SetC\2020Aug5_Jessa_3_Neg\2020Aug5_092.raw

E:\QE Data\2020Aug5_Jessa'sFlowers_SetC\2020Aug5_Jessa_3_Neg\2020Aug5_093.raw

E:\QE Data\2020Aug5_Jessa'sFlowers_SetC\2020Aug5_Jessa_3_Neg\2020Aug5_094.raw

E:\QE Data\2020Aug5_Jessa'sFlowers_SetC\2020Aug5_Jessa_3_Neg\2020Aug5_095.raw

E:\QE Data\2020Aug5_Jessa'sFlowers_SetC\2020Aug5_Jessa_3_Neg\2020Aug5_096.raw

E:\QE Data\2020Aug5_Jessa'sFlowers_SetC\2020Aug5_Jessa_3_Neg\2020Aug5_097.raw

E:\QE Data\2020Aug2_Jessa'sFlowers_SetB\2020Jul30_Jessa_2\2020Jul30_098.raw

E:\QE Data\2020Aug2_Jessa'sFlowers_SetB\2020Jul30_Jessa_2\2020Jul30_099.raw

E:\QE Data\2020Aug2_Jessa'sFlowers_SetB\2020Jul30_Jessa_2\2020Jul30_100.raw

E:\QE Data\2020Aug2_Jessa'sFlowers_SetB\2020Jul30_Jessa_2\2020Jul30_103.raw

E:\QE Data\2020Aug2_Jessa'sFlowers_SetB\2020Jul30_Jessa_2\2020Jul30_106.raw

E:\QE Data\2020Aug2_Jessa'sFlowers_SetB\2020Jul30_Jessa_2\2020Jul30_109.raw

E:\QE Data\2020Aug5_Jessa'sFlowers_SetC\2020Aug5_Jessa_3_Neg\2020Aug5_112.raw

E:\QE Data\2020Aug2_Jessa'sFlowers_SetB\2020Jul30_Jessa_2\2020Jul30_113.raw

E:\QE Data\2020Aug2_Jessa'sFlowers_SetB\2020Jul30_Jessa_2\2020Jul30_116.raw

E:\QE Data\2020Aug2_Jessa'sFlowers_SetB\2020Jul30_Jessa_2\2020Jul30_119.raw

E:\QE Data\2020Aug2_Jessa'sFlowers_SetB\2020Jul30_Jessa_2\2020Jul30_122.raw

------------------------------------------------------------------

Processing node 33: Select Spectra

------------------------------------------------------------------

1. Spectrum Properties Filter:

- Lower RT Limit: 1.2

- Upper RT Limit: 25

- First Scan: 0

- Last Scan: 0

- Ignore Specified Scans: (not specified)

- Lowest Charge State: 0

- Highest Charge State: 3

- Min. Precursor Mass: 140 Da

- Max. Precursor Mass: 4000 Da

- Total Intensity Threshold: 0

- Minimum Peak Count: 1

2. Scan Event Filters:

- Mass Analyzer: (not specified)

- MS Order: Any

- Activation Type: (not specified)

- Min. Collision Energy: 0

- Max. Collision Energy: 1000

- Scan Type: Any

- Polarity Mode: Any

3. Peak Filters:

- S/N Threshold (FT-only): 1.5

4. Replacements for Unrecognized Properties:

- Unrecognized Charge Replacements: 1

- Unrecognized Mass Analyzer Replacements: ITMS

- Unrecognized MS Order Replacements: MS2

- Unrecognized Activation Type Replacements: CID

- Unrecognized Polarity Replacements: +

- Unrecognized MS Resolution@200 Replacements: 60000

- Unrecognized MSn Resolution@200 Replacements: 30000

5. General Settings:

- Precursor Selection: Use MS1 Precursor

- Use Isotope Pattern in Precursor Reevaluation: True

- Provide Profile Spectra: Automatic

- Store Chromatograms: False

------------------------------------------------------------------

Processing node 26: Align Retention Times

------------------------------------------------------------------

1. General Settings:

- Alignment Model: Adaptive curve

- Alignment Fallback: Use Linear Model

- Maximum Shift [min]: 2

- Shift Reference File: False

- Mass Tolerance: 5 ppm

- Remove Outlier: True

------------------------------------------------------------------

Processing node 9: Detect Compounds

------------------------------------------------------------------

1. General Settings:

- Mass Tolerance [ppm]: 5 ppm

- Intensity Tolerance [%]: 30

- S/N Threshold: 3

- Min. Peak Intensity: 6000000

- Ions:

[2M+FA-H]-1

[2M-H]-1

[M+FA-H]-1

[M-2H]-2

[M-H]-1

[M-H-H2O]-1

- Base Ions: [M-H]-1

- Min. Element Counts: C H O

- Max. Element Counts: C150 H300 N10 Na2 O150 P S2

2. Peak Detection:

- Filter Peaks: True

- Max. Peak Width [min]: 0.5

- Remove Singlets: True

- Min. # Scans per Peak: 10

- Min. # Isotopes: 1

3. Isotope Grouping:

- Min. Spectral Distance Score: 0

- Remove Potentially False Positive Isotopes: True

------------------------------------------------------------------

Processing node 31: Group Compounds

------------------------------------------------------------------

1. Compound Consolidation:

- Mass Tolerance: 5 ppm

- RT Tolerance [min]: 0.2

2. Fragment Data Selection:

- Preferred Ions: [M-2H]-2; [M-H]-1

------------------------------------------------------------------

Processing node 32: Fill Gaps

------------------------------------------------------------------

1. General Settings:

- Mass Tolerance: 5 ppm

- S/N Threshold: 1.5

- Use Real Peak Detection: True

------------------------------------------------------------------

Processing node 39: Apply SERRF QC Correction [beta]

------------------------------------------------------------------

1. General Settings:

- Min. QC Coverage [%]: 75

- Max. QC Area RSD [%]: 30

- Max. Corrected QC Area RSD [%]: 25

- Max. # Files Between QC Files: 12

- Correct Blank Files: True

- # Batches: 3

- Interpolate Gap-filled QC Areas: False

2. Random Forest Settings:

- # Trees: 200

------------------------------------------------------------------

Processing node 40: Mark Background Compounds

------------------------------------------------------------------

1. General Settings:

- Max. Sample/Blank: 5

- Max. Blank/Sample: 0

- Hide Background: True

------------------------------------------------------------------

Processing node 23: Search ChemSpider

------------------------------------------------------------------

1. Search Settings:

- Database(s):

BioCyc

Carotenoids Database

Cayman Chemical

ChEBI

ChEMBL

FooDB

Human Metabolome Database

KEGG

MassBank

NIST

Phenol-Explorer

PlantCyc

Royal Society of Chemistry

Sigma-Aldrich

SMPDB Small Molecule Pathway Database

The Merck Index Online

- Search Mode: By Formula or Mass

- Mass Tolerance: 5 ppm

- Max. # of results per compound: 100

- Max. # of Predicted Compositions to be searched per Compound: 3

- Result Order (for Max. # of results per compound): Order By Reference Count (DESC)

2. Predicted Composition Annotation:

- Check All Predicted Compositions: False

------------------------------------------------------------------

Processing node 35: Apply mzLogic

------------------------------------------------------------------

1. Search Settings:

- FT Fragment Mass Tolerance: 10 ppm

- IT Fragment Mass Tolerance: 0.4 Da

- Max. # Compounds: 10

- Max. # mzCloud Similarity Results to consider per Compound: 10

- Match Factor Threshold: 30

------------------------------------------------------------------

Processing node 34: Map to Metabolika Pathways

------------------------------------------------------------------

1. Search Settings:

- Metabolika Pathways: (3R)-linalool biosynthesis.metabolika|2-nitrobenzoate degradation I.metabolika|2-oxobutanoate degradation I.metabolika|3-phenylpropanoate and 3-(3-hydroxyphenyl)propanoate degradation.metabolika|3-phenylpropanoate degradation.metabolika|Acetyl-CoA fermentation to butanoate II.metabolika|Adenosylcobalamin biosynthesis I (anaerobic).metabolika|Adenosylcobalamin biosynthesis II (aerobic).metabolika|Allantoin degradation IV (anaerobic).metabolika|Allantoin degradation to glyoxylate I.metabolika|Allantoin degradation to glyoxylate II.metabolika|Allantoin degradation to glyoxylate III.metabolika|Ammonia assimilation cycle I.metabolika|Ammonia assimilation cycle III.metabolika|Ammonia oxidation IV (autotrophic ammonia oxidizers).metabolika|Anaerobic aromatic compound degradation (Thauera aromatica).metabolika|Anaerobic energy metabolism (invertebrates, mitochondrial).metabolika|Arachidonate biosynthesis III (6-desaturase, mammals).metabolika|Archaetidylinositol biosynthesis.metabolika|Archaetidylserine and archaetidylethanolamine biosynthesis.metabolika|Arginine, ornithine and proline interconversion.metabolika|Aromatic compounds degradation via ss-ketoadipate.metabolika|Aspartate superpathway.metabolika|B-carotene biosynthesis (engineered).metabolika|Bacillibactin biosynthesis.metabolika|Benzoate biosynthesis I (CoA-dependent, ss-oxidative).metabolika|Benzoate biosynthesis III (CoA-dependent, non-ss-oxidative).metabolika|Benzoate fermentation (to acetate and cyclohexane carboxylate).metabolika|Biotin biosynthesis I.metabolika|Biotin biosynthesis II.metabolika|Bitter acids biosynthesis.metabolika|Caffeine degradation IV (bacteria, via demethylation and oxidation).metabolika|Cardiolipin and phosphatidylethanolamine biosynthesis (Xanthomonas).metabolika|Catechol degradation I (meta-cleavage pathway).metabolika|Catechol degradation II (meta-cleavage pathway).metabolika|Catechol degradation III (ortho-cleavage pathway).metabolika|Cellulose and hemicellulose degradation (cellulolosome).metabolika|Chitin biosynthesis.metabolika|Cholesterol biosynthesis I.metabolika|Cholesterol biosynthesis II (via 24,25-dihydrolanosterol).metabolika|Cholesterol biosynthesis III (via desmosterol).metabolika|Choline degradation IV.metabolika|Choline-O-sulfate degradation.metabolika|Chondroitin sulfate biosynthesis.metabolika|Chorismate biosynthesis I.metabolika|Chorismate biosynthesis II (archaea).metabolika|Colanic acid building blocks biosynthesis.metabolika|Crotonate fermentation (to acetate and cyclohexane carboxylate).metabolika|Curcuminoid biosynthesis.metabolika|D-serine metabolism.metabolika|Dermatan sulfate biosynthesis.metabolika|Enterobacterial common antigen biosynthesis.metabolika|Enterobactin biosynthesis.metabolika|G-butyrobetaine degradation.metabolika|GABA shunt.metabolika|Gamma-glutamyl cycle.metabolika|Gluconeogenesis II (Methanobacterium thermoautotrophicum).metabolika|Glycerol and glycerophosphodiester degradation.metabolika|Glycerol degradation to butanol.metabolika|Glycine biosynthesis II.metabolika|Heparan sulfate biosynthesis.metabolika|Hexitol fermentation to lactate, formate, ethanol and acetate.metabolika|Homolactic fermentation.metabolika|Hyperxanthone E biosynthesis.metabolika|Icosapentaenoate biosynthesis III (fungi).metabolika|Icosapentaenoate biosynthesis IV (bacteria).metabolika|Isoprene biosynthesis I.metabolika|Kanamycin biosynthesis.metabolika|Kauralexin biosynthesis.metabolika|Kdo transfer to lipid IVA III (Chlamydia).metabolika|Ketogluconate metabolism.metabolika|L-alanine fermentation to propanoate and acetate.metabolika|L-arginine biosynthesis I (via L-ornithine).metabolika|L-arginine degradation V (arginine deiminase pathway).metabolika|L-ascorbate biosynthesis V.metabolika|L-cysteine biosynthesis IV (from L-methionine).metabolika|L-cysteine biosynthesis IV (fungi).metabolika|L-glutamate and L-glutamine biosynthesis.metabolika|L-glutamate degradation IX (via 4-aminobutanoate).metabolika|L-glutamate degradation VII (to butanoate).metabolika|L-glutamate degradation VIII (to propanoate).metabolika|L-homoserine and L-methionine biosynthesis.metabolika|L-methionine biosynthesis III.metabolika|L-methionine salvage cycle I (bacteria and plants).metabolika|L-methionine salvage cycle II (plants).metabolika|L-methionine salvage cycle III.metabolika|L-tryptophan degradation III (eukaryotic).metabolika|L-tryptophan degradation IX.metabolika|L-tryptophan degradation XI (mammalian, via kynurenine).metabolika|L-tryptophan degradation XII (Geobacillus).metabolika|L-tyrosine degradation IV (to 4-methylphenol).metabolika|Mandelate degradation to acetyl-CoA.metabolika|Meta cleavage pathway of aromatic compounds.metabolika|Methanobacterium thermoautotrophicum biosynthetic metabolism.metabolika|Methanol and methylamine oxidation to formaldehyde.metabolika|Methanol oxidation to carbon dioxide.metabolika|Methylglyoxal degradation IV.metabolika|MRNA capping II.metabolika|Myo-, chiro- and scillo-inositol degradation.metabolika|N-acetylglucosamine degradation II.metabolika|NAD biosynthesis II (from tryptophan).metabolika|NAD salvage pathway III.metabolika|Naphthalene degradation to acetyl-CoA.metabolika|Nitrifier denitrification.metabolika|Novobiocin biosynthesis.metabolika|O-antigen building blocks biosynthesis (E. coli).metabolika|Oxygenic photosynthesis.metabolika|P-cumate degradation.metabolika|P-cymene degradation.metabolika|Pentose phosphate pathway.metabolika|Peptidoglycan biosynthesis I (meso-diaminopimelate containing).metabolika|Peptidoglycan biosynthesis II (staphylococci).metabolika|Peptidoglycan biosynthesis III (mycobacteria).metabolika|Peptidoglycan biosynthesis IV (Enterococcus faecium).metabolika|Peptidoglycan biosynthesis V (ss-lactam resistance).metabolika|Phosphatidylglycerol biosynthesis I (plastidic).metabolika|Phosphatidylglycerol biosynthesis II (non-plastidic).metabolika|Plant sterol biosynthesis.metabolika|Polyisoprenoid biosynthesis (E. coli).metabolika|Purine nucleotides degradation I (plants).metabolika|Purine nucleotides degradation II (aerobic).metabolika|Pyrimidine nucleobases salvage II.metabolika|Pyruvate fermentation to acetate and alanine.metabolika|Pyruvate fermentation to acetate and lactate I.metabolika|Pyruvate fermentation to acetate and lactate II.metabolika|Pyruvate fermentation to acetate I.metabolika|Pyruvate fermentation to acetate III.metabolika|Pyruvate fermentation to acetate IV.metabolika|Pyruvate fermentation to acetate V.metabolika|Pyruvate fermentation to acetate VI.metabolika|Pyruvate fermentation to acetate VII.metabolika|Reactive oxygen species degradation.metabolika|S-adenosyl-L-methionine cycle I.metabolika|Salicylate glucosides biosynthesis I.metabolika|Sphingolipid biosynthesis (mammals).metabolika|Sucrose biosynthesis I (from photosynthesis).metabolika|Sulfate reduction I (assimilatory).metabolika|Superpathway avenacin A biosynthesis.metabolika|Superpathway NADNADP - NADHNADPH interconversion (yeast).metabolika|Superpathway of (Kdo)2-lipid A biosynthesis.metabolika|Superpathway of (R,R)-butanediol biosynthesis.metabolika|Superpathway of 1D-myo-inositol hexakisphosphate biosynthesis (plants).metabolika|Superpathway of 2,3-butanediol biosynthesis.metabolika|Superpathway of 4-aminobutanoate degradation.metabolika|Superpathway of 4-hydroxybenzoate biosynthesis (yeast).metabolika|Superpathway of 5-aminoimidazole ribonucleotide biosynthesis.metabolika|Superpathway of acetate utilization and formation.metabolika|Superpathway of acetyl-CoA biosynthesis.metabolika|Superpathway of acrylonitrile degradation.metabolika|Superpathway of adenosine nucleotides de novo biosynthesis I.metabolika|Superpathway of adenosine nucleotides de novo biosynthesis II.metabolika|Superpathway of aerobic toluene degradation.metabolika|Superpathway of aflatoxin biosynthesis.metabolika|Superpathway of allantoin degradation in plants.metabolika|Superpathway of allantoin degradation in yeast.metabolika|Superpathway of Allium flavor precursors.metabolika|Superpathway of ammonia assimilation (plants).metabolika|Superpathway of anaerobic energy metabolism (invertebrates).metabolika|Superpathway of anaerobic sucrose degradation.metabolika|Superpathway of anthocyanin biosynthesis (from cyanidin and cyanidin 3-O-glucoside).metabolika|Superpathway of anthocyanin biosynthesis (from delphinidin 3-O-glucoside).metabolika|Superpathway of anthocyanin biosynthesis (from pelargonidin 3-O-glucoside).metabolika|Superpathway of arginine and polyamine biosynthesis.metabolika|Superpathway of aromatic amino acid biosynthesis.metabolika|Superpathway of aromatic compound degradation via 2-oxopent-4-enoate.metabolika|Superpathway of aromatic compound degradation via 3-oxoadipate.metabolika|Superpathway of atrazine degradation.metabolika|Superpathway of bacteriochlorophyll a biosynthesis.metabolika|Superpathway of benzoxazinoid glucosides biosynthesis.metabolika|Superpathway of betalain biosynthesis.metabolika|Superpathway of branched chain amino acid biosynthesis.metabolika|Superpathway of butirocin biosynthesis.metabolika|Superpathway of C1 compounds oxidation to CO2.metabolika|Superpathway of C28 brassinosteroid biosynthesis.metabolika|Superpathway of candicidin biosynthesis.metabolika|Superpathway of carotenoid biosynthesis.metabolika|Superpathway of CDP-glucose-derived O-antigen building blocks biosynthesis.metabolika|Superpathway of cholesterol biosynthesis.metabolika|Superpathway of cholesterol degradation I (cholesterol oxidase).metabolika|Superpathway of cholesterol degradation II (cholesterol dehydrogenase).metabolika|Superpathway of choline biosynthesis.metabolika|Superpathway of chorismate metabolism.metabolika|Superpathway of CMP-sialic acids biosynthesis.metabolika|Superpathway of coenzyme A biosynthesis I.metabolika|Superpathway of coenzyme A biosynthesis II (plants).metabolika|Superpathway of coenzyme A biosynthesis III (mammals).metabolika|Superpathway of cytosolic glycolysis (plants), pyruvate dehydrogenase and TCA cycle.metabolika|Superpathway of D-glucarate and D-galactarate degradation.metabolika|Superpathway of D-myo-inositol (1,4,5)-trisphosphate metabolism.metabolika|Superpathway of demethylmenaquinol-6 biosynthesis I.metabolika|Superpathway of demethylmenaquinol-6 biosynthesis II.metabolika|Superpathway of demethylmenaquinol-8 biosynthesis.metabolika|Superpathway of demethylmenaquinol-9 biosynthesis.metabolika|Superpathway of dimethylsulfone degradation.metabolika|Superpathway of dimethylsulfoniopropanoate degradation.metabolika|Superpathway of diterpene resin acids biosynthesis.metabolika|Superpathway of dTDP-glucose-derived antibiotic building blocks biosynthesis.metabolika|Superpathway of dTDP-glucose-derived O-antigen building blocks biosynthesis.metabolika|Superpathway of ergosterol biosynthesis I.metabolika|Superpathway of ergosterol biosynthesis II.metabolika|Superpathway of ergotamine biosynthesis.metabolika|Superpathway of erythromycin biosynthesis (without sugar biosynthesis).metabolika|Superpathway of erythromycin biosynthesis.metabolika|Superpathway of fatty acid biosynthesis I (E. coli).metabolika|Superpathway of fatty acid biosynthesis II (plant).metabolika|Superpathway of fatty acid biosynthesis initiation (E. coli).metabolika|Superpathway of fatty acids biosynthesis (E. coli).metabolika|Superpathway of fermentation (Chlamydomonas reinhardtii).metabolika|Superpathway of flavones and derivatives biosynthesis .metabolika|Superpathway of formononetin derivative biosynthesis.metabolika|Superpathway of fucose and rhamnose degradation.metabolika|Superpathway of fumitremorgin biosynthesis.metabolika|Superpathway of GDP-mannose-derived O-antigen building blocks biosynthesis.metabolika|Superpathway of geranylgeranyl diphosphate biosynthesis II (via MEP).metabolika|Superpathway of geranylgeranyldiphosphate biosynthesis I (via mevalonate).metabolika|Superpathway of gibberellin biosynthesis.metabolika|Superpathway of gibberellin GA12 biosynthesis.metabolika|Superpathway of glucose and xylose degradation.metabolika|Superpathway of glycerol degradation to 1,3-propanediol.metabolika|Superpathway of glycol metabolism and degradation.metabolika|Superpathway of glycolysis and Entner-Doudoroff.metabolika|Superpathway of glycolysis, pyruvate dehydrogenase, TCA, and glyoxylate bypass.metabolika|Superpathway of glyoxylate bypass and TCA.metabolika|Superpathway of glyoxylate cycle and fatty acid degradation.metabolika|Superpathway of guanine and guanosine salvage.metabolika|Superpathway of guanosine nucleotides degradation (plants).metabolika|Superpathway of guanosine nucleotides de novo biosynthesis I.metabolika|Superpathway of guanosine nucleotides de novo biosynthesis II.metabolika|Superpathway of heme biosynthesis from glutamate.metabolika|Superpathway of heme biosynthesis from glycine.metabolika|Superpathway of heme biosynthesis from uroporphyrinogen-III.metabolika|Superpathway of hexitol degradation (bacteria).metabolika|Superpathway of hexuronide and hexuronate degradation.metabolika|Superpathway of histidine, purine, and pyrimidine biosynthesis.metabolika|Superpathway of hydrogen production.metabolika|Superpathway of hydrolyzable tannin biosynthesis.metabolika|Superpathway of hyoscyamine and scopolamine biosynthesis.metabolika|Superpathway of indole-3-acetate conjugate biosynthesis.metabolika|Superpathway of inositol phosphate compounds.metabolika|Superpathway of isoflavonoids (via naringenin).metabolika|Superpathway of jasmonoyl-amino acid conjugates biosynthesis.metabolika|Superpathway of L-alanine biosynthesis.metabolika|Superpathway of L-arginine and L-ornithine degradation.metabolika|Superpathway of L-arginine, putrescine, and 4-aminobutanoate degradation.metabolika|Superpathway of L-asparagine biosynthesis.metabolika|Superpathway of L-aspartate and L-asparagine biosynthesis.metabolika|Superpathway of L-citrulline metabolism.metabolika|Superpathway of L-cysteine biosynthesis (mammalian).metabolika|Superpathway of L-isoleucine biosynthesis I.metabolika|Superpathway of L-lysine degradation.metabolika|Superpathway of L-lysine, L-threonine and L-methionine biosynthesis I.metabolika|Superpathway of L-lysine, L-threonine and L-methionine biosynthesis II.metabolika|Superpathway of L-methionine biosynthesis (by sulfhydrylation).metabolika|Superpathway of L-methionine biosynthesis (transsulfuration).metabolika|Superpathway of L-methionine salvage and degradation.metabolika|Superpathway of L-phenylalanine and L-tyrosine biosynthesis.metabolika|Superpathway of L-phenylalanine biosynthesis.metabolika|Superpathway of L-serine and glycine biosynthesis I.metabolika|Superpathway of L-threonine biosynthesis.metabolika|Superpathway of L-threonine metabolism.metabolika|Superpathway of L-tryptophan biosynthesis.metabolika|Superpathway of L-tyrosine biosynthesis.metabolika|Superpathway of linalool biosynthesis.metabolika|Superpathway of linamarin and lotaustralin biosynthesis.metabolika|Superpathway of lipopolysaccharide biosynthesis.metabolika|Superpathway of lipoxygenase.metabolika|Superpathway of megalomicin A biosynthesis.metabolika|Superpathway of melatonin degradation.metabolika|Superpathway of menaquinol-10 biosynthesis.metabolika|Superpathway of menaquinol-11 biosynthesis.metabolika|Superpathway of menaquinol-12 biosynthesis.metabolika|Superpathway of menaquinol-13 biosynthesis.metabolika|Superpathway of menaquinol-6 biosynthesis I.metabolika|Superpathway of menaquinol-7 biosynthesis.metabolika|Superpathway of menaquinol-8 biosynthesis I.metabolika|Superpathway of menaquinol-8 biosynthesis II.metabolika|Superpathway of menaquinol-9 biosynthesis.metabolika|Superpathway of methanogenesis.metabolika|Superpathway of methylglyoxal degradation.metabolika|Superpathway of microbial D-galacturonate and D-glucuronate degradation.metabolika|Superpathway of mycolyl-arabinogalactan-peptidoglycan complex biosynthesis.metabolika|Superpathway of NAD biosynthesis in eukaryotes.metabolika|Superpathway of neomycin biosynthesis.metabolika|Superpathway of nicotinate degradation.metabolika|Superpathway of nicotine biosynthesis.metabolika|Superpathway of oleoresin turpentine biosynthesis.metabolika|Superpathway of ornithine degradation.metabolika|Superpathway of penicillin, cephalosporin and cephamycin biosynthesis.metabolika|Superpathway of pentose and pentitol degradation.metabolika|Superpathway of phenylethylamine degradation.metabolika|Superpathway of phosphatidylcholine biosynthesis.metabolika|Superpathway of phospholipid biosynthesis I (bacteria).metabolika|Superpathway of phospholipid biosynthesis II (plants).metabolika|Superpathway of photosynthetic hydrogen production.metabolika|Superpathway of phylloquinol biosynthesis.metabolika|Superpathway of plastoquinol biosynthesis.metabolika|Superpathway of polyamine biosynthesis I.metabolika|Superpathway of polyamine biosynthesis II.metabolika|Superpathway of polyamine biosynthesis III.metabolika|Superpathway of pterocarpan biosynthesis (via daidzein).metabolika|Superpathway of pterocarpan biosynthesis (via formononetin).metabolika|Superpathway of purine deoxyribonucleosides degradation.metabolika|Superpathway of purine nucleotide salvage.metabolika|Superpathway of purine nucleotides de novo biosynthesis I.metabolika|Superpathway of purine nucleotides de novo biosynthesis II.metabolika|Superpathway of purines degradation in plants.metabolika|Superpathway of pyridoxal 5'-phosphate biosynthesis and salvage.metabolika|Superpathway of pyrimidine deoxyribonucleoside salvage.metabolika|Superpathway of pyrimidine deoxyribonucleosides degradation.metabolika|Superpathway of pyrimidine deoxyribonucleotides de novo biosynthesis (E. coli).metabolika|Superpathway of pyrimidine deoxyribonucleotides de novo biosynthesis.metabolika|Superpathway of pyrimidine nucleobases salvage.metabolika|Superpathway of pyrimidine ribonucleosides degradation.metabolika|Superpathway of pyrimidine ribonucleosides salvage.metabolika|Superpathway of pyrimidine ribonucleotides de novo biosynthesis.metabolika|Superpathway of quinolone and alkylquinolone biosynthesis.metabolika|Superpathway of rifamycin B biosynthesis.metabolika|Superpathway of roquefortine, meleagrin and neoxaline biosynthesis.metabolika|Superpathway of rosmarinic acid biosynthesis.metabolika|Superpathway of salicylate degradation.metabolika|Superpathway of scopolin and esculin biosynthesis.metabolika|Superpathway of seleno-compound metabolism.metabolika|Superpathway of ss-D-glucuronide and D-glucuronate degradation.metabolika|Superpathway of stearidonate biosynthesis (cyanobacteria).metabolika|Superpathway of steroid hormone biosynthesis.metabolika|Superpathway of sulfate assimilation and cysteine biosynthesis.metabolika|Superpathway of sulfide oxidation (Acidithiobacillus ferrooxidans).metabolika|Superpathway of sulfide oxidation (phototrophic sulfur bacteria).metabolika|Superpathway of sulfide oxidation (Starkeya novella).metabolika|Superpathway of sulfolactate degradation.metabolika|Superpathway of sulfur amino acid biosynthesis (Saccharomyces cerevisiae).metabolika|Superpathway of sulfur metabolism (Desulfocapsa sulfoexigens).metabolika|Superpathway of sulfur oxidation (Acidianus ambivalens).metabolika|Superpathway of taurine degradation.metabolika|Superpathway of testosterone and androsterone degradation.metabolika|Superpathway of tetracycline and oxytetracycline biosynthesis.metabolika|Superpathway of tetrahydrofolate biosynthesis and salvage.metabolika|Superpathway of tetrahydrofolate biosynthesis.metabolika|Superpathway of tetrahydroxyxanthone biosynthesis.metabolika|Superpathway of tetrathionate reduction (Salmonella typhimurium).metabolika|Superpathway of the 3-hydroxypropanoate cycle.metabolika|Superpathway of thiamine diphosphate biosynthesis I.metabolika|Superpathway of thiamine diphosphate biosynthesis II.metabolika|Superpathway of thiamine diphosphate biosynthesis III (eukaryotes).metabolika|Superpathway of thiosulfate metabolism (Desulfovibrio sulfodismutans).metabolika|Superpathway of trichothecene biosynthesis.metabolika|Superpathway of trimethylamine degradation.metabolika|Superpathway of ubiquinol-6 biosynthesis (eukaryotic).metabolika|Superpathway of ubiquinol-8 biosynthesis (prokaryotic).metabolika|Superpathway of UDP-glucose-derived O-antigen building blocks biosynthesis.metabolika|Superpathway of UDP-N-acetylglucosamine-derived O-antigen building blocks biosynthesis.metabolika|Superpathway of unsaturated fatty acids biosynthesis (E. coli).metabolika|Superpathway of vanillin and vanillate degradation.metabolika|Superpathway of Clostridium acetobutylicum acidogenic and solventogenic fermentation.metabolika|Superpathway of Clostridium acetobutylicum acidogenic fermentation.metabolika|Superpathway of Clostridium acetobutylicum solventogenic fermentation.metabolika|Superpathway of N-acetylglucosamine, N-acetylmannosamine and N-acetylneuraminate degradation.metabolika|Superpathway of N-acetylneuraminate degradation.metabolika|Superpathway of S-adenosyl-L-methionine biosynthesis.metabolika|Superpathway polymethylated quercetinquercetagetin glucoside biosynthesis (Chrysosplenium).metabolika|Superpathways of coenzyme A biosynthesis I.metabolika|Superpathways of coenzyme A biosynthesis III (mammals).metabolika|Syringate degradation.metabolika|Taxadiene biosynthesis (engineered).metabolika|Thiamine salvage II.metabolika|Toluene degradation I (aerobic) (via o-cresol).metabolika|Toluene degradation II (aerobic) (via 4-methylcatechol).metabolika|Toluene degradation III (aerobic) (via p-cresol).metabolika|Toluene degradation IV (aerobic) (via catechol).metabolika|Toluene degradation V (aerobic) (via toluene-cis-diol).metabolika|Toluene degradation VI (anaerobic).metabolika|Trans-lycopene biosynthesis I (bacteria).metabolika|UDP-D-xylose biosynthesis.metabolika|UDP-galactofuranose biosynthesis.metabolika|UDP-sugars interconversion.metabolika|Ureide biosynthesis.metabolika|Vibriobactin biosynthesis.metabolika|Wybutosine biosynthesis.metabolika

- Search Mode: By Formula or Mass

2. By Mass Search Settings:

- Mass Tolerance: 5 ppm

3. By Formula Search Settings:

- Max. # of Predicted Compositions to be searched per Compound: 3

4. Display Settings:

- Max. # Pathways in 'Pathways' column: 20

------------------------------------------------------------------

Processing node 36: Search Mass Lists

------------------------------------------------------------------

1. Search Settings:

- Mass Lists: Arita Lab 6549 Flavonoid Structure Database.masslist|Extractables and Leachables HRAM Compound Database.massList|Natural Products Atlas 2020_06.massList|Polyphenol Compound Database_RT_1FA_2020Jun1_JessaSamples_injected16Mar2019.massList|Prodelphinidin Compound Database_RT_2018Apr25.massList

- Mass Tolerance: 5 ppm

- Use Retention Time: True

- RT Tolerance [min]: 0.2

------------------------------------------------------------------

Processing node 29: Predict Compositions

------------------------------------------------------------------

1. Prediction Settings:

- Mass Tolerance: 5 ppm

- Min. Element Counts: C H

- Max. Element Counts: C120 H240 N4 O150 P S

- Min. RDBE: 0

- Max. RDBE: 40

- Min. H/C: 0.1

- Max. H/C: 4

- Max. # Candidates: 50

- Max. # Internal Candidates: 200

2. Pattern Matching:

- Intensity Tolerance [%]: 30

- Intensity Threshold [%]: 0.1

- S/N Threshold: 3

- Min. Spectral Fit [%]: 30

- Min. Pattern Cov. [%]: 90

- Use Dynamic Recalibration: True

3. Fragments Matching:

- Use Fragments Matching: False

- Mass Tolerance: 5 ppm

- S/N Threshold: 3

------------------------------------------------------------------

Processing node 25: Assign Compound Annotations

------------------------------------------------------------------

1. General Settings:

- Mass Tolerance: 5 ppm

2. Data Sources:

- Data Source #1: MassList Search

- Data Source #2: mzVault Search

- Data Source #3: mzCloud Search

- Data Source #4: Metabolika Search

- Data Source #5: Predicted Compositions

- Data Source #6: (not specified)

- Data Source #7: (not specified)

3. Scoring Rules:

- Use mzLogic: True

- Use Spectral Distance: True

- SFit Threshold: 20

- SFit Range: 20

------------------------------------------------------------------

Processing node 22: Search mzCloud

------------------------------------------------------------------

1. General Settings:

- Compound Classes: All

- Precursor Mass Tolerance: 10 ppm

- FT Fragment Mass Tolerance: 10 ppm

- IT Fragment Mass Tolerance: 0.4 Da

- Library: Autoprocessed; Reference

- Post Processing: Recalibrated

- Max. # Results: 10

- Annotate Matching Fragments: False

2. DDA Search:

- Identity Search: HighChem HighRes

- Match Activation Type: True

- Match Activation Energy: Match with Tolerance

- Activation Energy Tolerance: 40

- Apply Intensity Threshold: True

- Similarity Search: Confidence Reverse

- Match Factor Threshold: 50

3. DIA Search:

- Use DIA Scans for Search: False

- Max. Isolation Width [Da]: 500

- Match Activation Type: False

- Match Activation Energy: Any

- Activation Energy Tolerance: 100

- Apply Intensity Threshold: False

- Match Factor Threshold: 20

------------------------------------------------------------------

Processing node 37: Search mzVault

------------------------------------------------------------------

1. Search Settings:

- mzVault Library: Bamba lab 34 lipid mediators library stepped NCE 10 30 45.db|Bamba lab 598 polar metabolites stepped NCE 10 30 45.db|Polyphenol_CG_oligomers_2018May4.db|PP_2018May9.db|Negative ion mode_Jan2021.db

- Max. # Results: 10

- Match Factor Threshold: 50

- Search Algorithm: HighChem HighRes

- Match Analyzer Type: True

- IT Fragment Mass Tolerance: 0.4 Da

- FT Fragment Mass Tolerance: 10 ppm

- Use Retention Time: False

- Precursor Mass Tolerance: 10 ppm

- Apply Intensity Threshold: True

- Match Ionization Method: True

- Ion Activation Energy Tolerance: 50

- Match Ion Activation Energy: Match with Tolerance

- Match Ion Activation Type: True

- Compound Classes: All

- Remove Precursor Ion: True

- RT Tolerance [min]: 0.2

------------------------------------------------------------------

Processing node 17: Differential Analysis

------------------------------------------------------------------

1. General Settings:

- Log10 Transform Values: True

------------------------------------------------------------------

Processing node 38: Descriptive Statistics

------------------------------------------------------------------

No parameters

This file contains the following filters:

Row Filter for Compounds:

------------------------------------

AND

|

+--BackgroundStatus is false

|

+--NormArea in any category

|

+--in category

| |

| +--Tags

| |

| +--1

|

+--in category

|

+--Tags

|

+--6

## Study

=== Study: FababeanFlowers_Jessa'sSamples_Sep2021_CD3.2, 5/30/2020 8:07:57 AM ===

Directory: C:\Users\Public\Documents\Thermo\Compound Discoverer 3.2\Elessawy\FabaBean Flowers_Sep2021_CD3.2

------------------------------------------------------------------

Factors:

------------------------------------------------------------------

Sample number

Options: 1, 2, 3, 4, 5, 6, 7, 8, 9, 10, 11, 12, 13, 14, 15, 16, 17, 18, 19, 20, 21, 22, 23, 24, 25, 26, 27, 28, 29, 30, 31, 32, 33, 34, 35, 36, 37, 38, 39, 40, 41, 42, 45, 46, 47, 48, 49, 50, 51, 52, 53, 54, 55, 56, 57, 58, 59, 60, 61, 62, 63, 64, 65, 66, 67, 68, 69, 70, 71, 72, 73, 74, 75, 76, 77, 78

Population

Options: F2, 71A, 71B, P, 2263

Female P

Options: Gelber, P47-1, Snowdrop, Aurora, Rinrei, Disco/2

Male P

Options: P47-1, Gelber, Disco/2, NV153, Snowdrop, Rinrei, Aurora

Color group

Options: Brown, Brown mixed, Red, White, White with patterns

Floral organ

Options: W, ST

Secondary color

Options: red, pink, brown, yellow, purple, white

General main color

Options: brown, red, white

Main color

Options: white, brown, pink, red

General secondary color

Options: white, brown, red, purple

ColorCategory

Options: Brown, White, Red, RedBrown, WhiteBrown, RedPurple, WhiteYellow, BrownPurple

Red

Options: Yes, No

Brown

Options: Yes, No

Yellow

Options: Yes, No

Purple

Options: Yes, No

White

Options: Yes, No

Pink

Options: Yes, No

ColorCategories_Updated

Options: White, WhiteBrown, WhiteYellow, Red, Brown, BrownRed, BrownPink, RedPurple

FlowerCategories_W_ST

Options: White, WhiteYellow, WhiteBrown, Brown, BrownPink, BrownRed, RedPurple

MainPetalColor

Options: White, Brown, Pink, Red, Purplish red

MinorPetalColor_Absent

Options: Brown, Red, Pink, Yellow, Absent

MinorPetalColor

Options: White, Yellow, Red, Brown, Pink, Purplish red

PetalColorGroup1

Options: White, WhiteYellow, WhiteBrown, Brown, BrownPink, PinkBrown, Pink, Red, RedBrown, BrownRed, Purplish red, WhitePink

PetalColorGroup2

Options: White, WhiteYellow, WhiteBrown, Brown, Red, Purplish red, BrownRed-RedBrown, BrownPink-PinkBrown, WhitePink

------------------------------------------------------------------

Samples and Files:

------------------------------------------------------------------

[S1] 190312QE_001 [Sample number=n/a, Population=n/a, Female P=n/a, Male P=n/a, Color group=n/a, Floral organ=n/a, Secondary color=n/a, General main color=n/a, Main color=n/a, General secondary color=n/a, ColorCategory=n/a, Red=n/a, Brown=n/a, Yellow=n/a, Purple=n/a, White=n/a, Pink=n/a, ColorCategories_Updated=n/a, FlowerCategories_W_ST=n/a, MainPetalColor=n/a, MinorPetalColor_Absent=n/a, MinorPetalColor=n/a, PetalColorGroup1=n/a, PetalColorGroup2=n/a]

E:\QE Data\2019MAR12_Jessa\190312QE_001.raw

[S2] 190312QE_002 [Sample number=n/a, Population=n/a, Female P=n/a, Male P=n/a, Color group=n/a, Floral organ=n/a, Secondary color=n/a, General main color=n/a, Main color=n/a, General secondary color=n/a, ColorCategory=n/a, Red=n/a, Brown=n/a, Yellow=n/a, Purple=n/a, White=n/a, Pink=n/a, ColorCategories_Updated=n/a, FlowerCategories_W_ST=n/a, MainPetalColor=n/a, MinorPetalColor_Absent=n/a, MinorPetalColor=n/a, PetalColorGroup1=n/a, PetalColorGroup2=n/a]

E:\QE Data\2019MAR12_Jessa\190312QE_002.raw

[S5] 190312QE_005 [Sample number=n/a, Population=n/a, Female P=n/a, Male P=n/a, Color group=n/a, Floral organ=n/a, Secondary color=n/a, General main color=n/a, Main color=n/a, General secondary color=n/a, ColorCategory=n/a, Red=n/a, Brown=n/a, Yellow=n/a, Purple=n/a, White=n/a, Pink=n/a, ColorCategories_Updated=n/a, FlowerCategories_W_ST=n/a, MainPetalColor=n/a, MinorPetalColor_Absent=n/a, MinorPetalColor=n/a, PetalColorGroup1=n/a, PetalColorGroup2=n/a]

E:\QE Data\2019MAR12_Jessa\190312QE_005.raw

[S6] 190312QE_006 [Sample number=n/a, Population=n/a, Female P=n/a, Male P=n/a, Color group=n/a, Floral organ=n/a, Secondary color=n/a, General main color=n/a, Main color=n/a, General secondary color=n/a, ColorCategory=n/a, Red=n/a, Brown=n/a, Yellow=n/a, Purple=n/a, White=n/a, Pink=n/a, ColorCategories_Updated=n/a, FlowerCategories_W_ST=n/a, MainPetalColor=n/a, MinorPetalColor_Absent=n/a, MinorPetalColor=n/a, PetalColorGroup1=n/a, PetalColorGroup2=n/a]

E:\QE Data\2019MAR12_Jessa\190312QE_006.raw

[S7] 190312QE_007 [Sample number=n/a, Population=n/a, Female P=n/a, Male P=n/a, Color group=n/a, Floral organ=n/a, Secondary color=n/a, General main color=n/a, Main color=n/a, General secondary color=n/a, ColorCategory=n/a, Red=n/a, Brown=n/a, Yellow=n/a, Purple=n/a, White=n/a, Pink=n/a, ColorCategories_Updated=n/a, FlowerCategories_W_ST=n/a, MainPetalColor=n/a, MinorPetalColor_Absent=n/a, MinorPetalColor=n/a, PetalColorGroup1=n/a, PetalColorGroup2=n/a]

E:\QE Data\2019MAR12_Jessa\190312QE_007.raw

[S8] 190312QE_008 [Sample number=n/a, Population=n/a, Female P=n/a, Male P=n/a, Color group=n/a, Floral organ=n/a, Secondary color=n/a, General main color=n/a, Main color=n/a, General secondary color=n/a, ColorCategory=n/a, Red=n/a, Brown=n/a, Yellow=n/a, Purple=n/a, White=n/a, Pink=n/a, ColorCategories_Updated=n/a, FlowerCategories_W_ST=n/a, MainPetalColor=n/a, MinorPetalColor_Absent=n/a, MinorPetalColor=n/a, PetalColorGroup1=n/a, PetalColorGroup2=n/a]

E:\QE Data\2019MAR12_Jessa\190312QE_008.raw

[S9] 190312QE_009 [Sample number=n/a, Population=n/a, Female P=n/a, Male P=n/a, Color group=n/a, Floral organ=n/a, Secondary color=n/a, General main color=n/a, Main color=n/a, General secondary color=n/a, ColorCategory=n/a, Red=n/a, Brown=n/a, Yellow=n/a, Purple=n/a, White=n/a, Pink=n/a, ColorCategories_Updated=n/a, FlowerCategories_W_ST=n/a, MainPetalColor=n/a, MinorPetalColor_Absent=n/a, MinorPetalColor=n/a, PetalColorGroup1=n/a, PetalColorGroup2=n/a]

E:\QE Data\2019MAR12_Jessa\190312QE_009.raw

[S10] 190312QE_010 [Sample number=n/a, Population=n/a, Female P=n/a, Male P=n/a, Color group=n/a, Floral organ=n/a, Secondary color=n/a, General main color=n/a, Main color=n/a, General secondary color=n/a, ColorCategory=n/a, Red=n/a, Brown=n/a, Yellow=n/a, Purple=n/a, White=n/a, Pink=n/a, ColorCategories_Updated=n/a, FlowerCategories_W_ST=n/a, MainPetalColor=n/a, MinorPetalColor_Absent=n/a, MinorPetalColor=n/a, PetalColorGroup1=n/a, PetalColorGroup2=n/a]

E:\QE Data\2019MAR12_Jessa\190312QE_010.raw

[S11] 190312QE_011 [Sample number=n/a, Population=n/a, Female P=n/a, Male P=n/a, Color group=n/a, Floral organ=n/a, Secondary color=n/a, General main color=n/a, Main color=n/a, General secondary color=n/a, ColorCategory=n/a, Red=n/a, Brown=n/a, Yellow=n/a, Purple=n/a, White=n/a, Pink=n/a, ColorCategories_Updated=n/a, FlowerCategories_W_ST=n/a, MainPetalColor=n/a, MinorPetalColor_Absent=n/a, MinorPetalColor=n/a, PetalColorGroup1=n/a, PetalColorGroup2=n/a]

E:\QE Data\2019MAR12_Jessa\190312QE_011.raw

[S12] 190312QE_012 [Sample number=n/a, Population=n/a, Female P=n/a, Male P=n/a, Color group=n/a, Floral organ=n/a, Secondary color=n/a, General main color=n/a, Main color=n/a, General secondary color=n/a, ColorCategory=n/a, Red=n/a, Brown=n/a, Yellow=n/a, Purple=n/a, White=n/a, Pink=n/a, ColorCategories_Updated=n/a, FlowerCategories_W_ST=n/a, MainPetalColor=n/a, MinorPetalColor_Absent=n/a, MinorPetalColor=n/a, PetalColorGroup1=n/a, PetalColorGroup2=n/a]

E:\QE Data\2019MAR12_Jessa\190312QE_012.raw

[S13] 190312QE_013 [Sample number=n/a, Population=n/a, Female P=n/a, Male P=n/a, Color group=n/a, Floral organ=n/a, Secondary color=n/a, General main color=n/a, Main color=n/a, General secondary color=n/a, ColorCategory=n/a, Red=n/a, Brown=n/a, Yellow=n/a, Purple=n/a, White=n/a, Pink=n/a, ColorCategories_Updated=n/a, FlowerCategories_W_ST=n/a, MainPetalColor=n/a, MinorPetalColor_Absent=n/a, MinorPetalColor=n/a, PetalColorGroup1=n/a, PetalColorGroup2=n/a]

E:\QE Data\2019MAR12_Jessa\190312QE_013.raw

[S14] 190312QE_014 [Sample number=26, Population=F2, Female P=Gelber, Male P=P47-1, Color group=White, Floral organ=ST, Secondary color=pink, General main color=white, Main color=white, General secondary color=white, ColorCategory=White, Red=No, Brown=No, Yellow=No, Purple=No, White=Yes, Pink=Yes, ColorCategories_Updated=White, FlowerCategories_W_ST=WhiteYellow, MainPetalColor=White, MinorPetalColor_Absent=Pink, MinorPetalColor=Pink, PetalColorGroup1=WhitePink, PetalColorGroup2=WhitePink]

E:\QE Data\2019MAR12_Jessa\190312QE_014.raw

[S16] 190312QE_016 [Sample number=28, Population=F2, Female P=P47-1, Male P=Snowdrop, Color group=White, Floral organ=ST, Secondary color=white, General main color=white, Main color=white, General secondary color=white, ColorCategory=White, Red=No, Brown=No, Yellow=No, Purple=No, White=Yes, Pink=No, ColorCategories_Updated=White, FlowerCategories_W_ST=White, MainPetalColor=White, MinorPetalColor_Absent=Absent, MinorPetalColor=White, PetalColorGroup1=White, PetalColorGroup2=White]

E:\QE Data\2019MAR12_Jessa\190312QE_016.raw

[S17] 190312QE_017 [Sample number=30, Population=F2, Female P=P47-1, Male P=Snowdrop, Color group=White, Floral organ=ST, Secondary color=pink, General main color=white, Main color=white, General secondary color=white, ColorCategory=White, Red=No, Brown=No, Yellow=No, Purple=No, White=Yes, Pink=Yes, ColorCategories_Updated=White, FlowerCategories_W_ST=WhiteBrown, MainPetalColor=White, MinorPetalColor_Absent=Pink, MinorPetalColor=Pink, PetalColorGroup1=WhitePink, PetalColorGroup2=WhitePink]

E:\QE Data\2019MAR12_Jessa\190312QE_017.raw

[S20] 190312QE_020 [Sample number=n/a, Population=n/a, Female P=n/a, Male P=n/a, Color group=n/a, Floral organ=n/a, Secondary color=n/a, General main color=n/a, Main color=n/a, General secondary color=n/a, ColorCategory=n/a, Red=n/a, Brown=n/a, Yellow=n/a, Purple=n/a, White=n/a, Pink=n/a, ColorCategories_Updated=n/a, FlowerCategories_W_ST=n/a, MainPetalColor=n/a, MinorPetalColor_Absent=n/a, MinorPetalColor=n/a, PetalColorGroup1=n/a, PetalColorGroup2=n/a]

E:\QE Data\2019MAR12_Jessa\190312QE_020.raw

[S21] 190312QE_021 [Sample number=n/a, Population=n/a, Female P=n/a, Male P=n/a, Color group=n/a, Floral organ=n/a, Secondary color=n/a, General main color=n/a, Main color=n/a, General secondary color=n/a, ColorCategory=n/a, Red=n/a, Brown=n/a, Yellow=n/a, Purple=n/a, White=n/a, Pink=n/a, ColorCategories_Updated=n/a, FlowerCategories_W_ST=n/a, MainPetalColor=n/a, MinorPetalColor_Absent=n/a, MinorPetalColor=n/a, PetalColorGroup1=n/a, PetalColorGroup2=n/a]

E:\QE Data\2019MAR12_Jessa\190312QE_021.raw

[S22] 190312QE_022 [Sample number=45, Population=F2, Female P=Snowdrop, Male P=Gelber, Color group=White, Floral organ=W, Secondary color=white, General main color=white, Main color=white, General secondary color=white, ColorCategory=White, Red=No, Brown=No, Yellow=No, Purple=No, White=Yes, Pink=No, ColorCategories_Updated=White, FlowerCategories_W_ST=White, MainPetalColor=White, MinorPetalColor_Absent=Absent, MinorPetalColor=White, PetalColorGroup1=White, PetalColorGroup2=White]

E:\QE Data\2019MAR12_Jessa\190312QE_022.raw

[S23] 190312QE_023 [Sample number=46, Population=F2, Female P=Snowdrop, Male P=Gelber, Color group=White, Floral organ=ST, Secondary color=white, General main color=white, Main color=white, General secondary color=white, ColorCategory=White, Red=No, Brown=No, Yellow=No, Purple=No, White=Yes, Pink=No, ColorCategories_Updated=White, FlowerCategories_W_ST=White, MainPetalColor=White, MinorPetalColor_Absent=Absent, MinorPetalColor=White, PetalColorGroup1=White, PetalColorGroup2=White]

E:\QE Data\2019MAR12_Jessa\190312QE_023.raw

[S24] 190312QE_024 [Sample number=52, Population=F2, Female P=Disco/2, Male P=Gelber, Color group=White, Floral organ=ST, Secondary color=pink, General main color=white, Main color=white, General secondary color=white, ColorCategory=White, Red=No, Brown=No, Yellow=No, Purple=No, White=Yes, Pink=Yes, ColorCategories_Updated=White, FlowerCategories_W_ST=WhiteYellow, MainPetalColor=White, MinorPetalColor_Absent=Pink, MinorPetalColor=Pink, PetalColorGroup1=WhitePink, PetalColorGroup2=WhitePink]

E:\QE Data\2019MAR12_Jessa\190312QE_024.raw

[S25] 190312QE_025 [Sample number=53, Population=F2, Female P=Disco/2, Male P=Gelber, Color group=White, Floral organ=W, Secondary color=white, General main color=white, Main color=white, General secondary color=white, ColorCategory=White, Red=No, Brown=No, Yellow=No, Purple=No, White=Yes, Pink=No, ColorCategories_Updated=White, FlowerCategories_W_ST=White, MainPetalColor=White, MinorPetalColor_Absent=Absent, MinorPetalColor=White, PetalColorGroup1=White, PetalColorGroup2=White]

E:\QE Data\2019MAR12_Jessa\190312QE_025.raw

[S26] 190312QE_026 [Sample number=54, Population=F2, Female P=Disco/2, Male P=Gelber, Color group=White, Floral organ=ST, Secondary color=white, General main color=white, Main color=white, General secondary color=white, ColorCategory=White, Red=No, Brown=No, Yellow=No, Purple=No, White=Yes, Pink=No, ColorCategories_Updated=White, FlowerCategories_W_ST=White, MainPetalColor=White, MinorPetalColor_Absent=Absent, MinorPetalColor=White, PetalColorGroup1=White, PetalColorGroup2=White]

E:\QE Data\2019MAR12_Jessa\190312QE_026.raw

[S28] 190312QE_028 [Sample number=n/a, Population=n/a, Female P=n/a, Male P=n/a, Color group=n/a, Floral organ=n/a, Secondary color=n/a, General main color=n/a, Main color=n/a, General secondary color=n/a, ColorCategory=n/a, Red=n/a, Brown=n/a, Yellow=n/a, Purple=n/a, White=n/a, Pink=n/a, ColorCategories_Updated=n/a, FlowerCategories_W_ST=n/a, MainPetalColor=n/a, MinorPetalColor_Absent=n/a, MinorPetalColor=n/a, PetalColorGroup1=n/a, PetalColorGroup2=n/a]

E:\QE Data\2019MAR12_Jessa\190312QE_028.raw

[S29] 190312QE_029 [Sample number=n/a, Population=n/a, Female P=n/a, Male P=n/a, Color group=n/a, Floral organ=n/a, Secondary color=n/a, General main color=n/a, Main color=n/a, General secondary color=n/a, ColorCategory=n/a, Red=n/a, Brown=n/a, Yellow=n/a, Purple=n/a, White=n/a, Pink=n/a, ColorCategories_Updated=n/a, FlowerCategories_W_ST=n/a, MainPetalColor=n/a, MinorPetalColor_Absent=n/a, MinorPetalColor=n/a, PetalColorGroup1=n/a, PetalColorGroup2=n/a]

E:\QE Data\2019MAR12_Jessa\190312QE_029.raw

[S33] 190312QE_033 [Sample number=23, Population=F2, Female P=Gelber, Male P=P47-1, Color group=White with patterns, Floral organ=W, Secondary color=brown, General main color=white, Main color=white, General secondary color=brown, ColorCategory=WhiteBrown, Red=No, Brown=Yes, Yellow=No, Purple=No, White=Yes, Pink=No, ColorCategories_Updated=WhiteBrown, FlowerCategories_W_ST=WhiteBrown, MainPetalColor=White, MinorPetalColor_Absent=Brown, MinorPetalColor=Brown, PetalColorGroup1=WhiteBrown, PetalColorGroup2=WhiteBrown]

E:\QE Data\2019MAR12_Jessa\190312QE_033.raw

[S34] 190312QE_034 [Sample number=24, Population=F2, Female P=Gelber, Male P=P47-1, Color group=White with patterns, Floral organ=ST, Secondary color=brown, General main color=white, Main color=white, General secondary color=brown, ColorCategory=WhiteBrown, Red=No, Brown=Yes, Yellow=No, Purple=No, White=Yes, Pink=No, ColorCategories_Updated=WhiteBrown, FlowerCategories_W_ST=WhiteBrown, MainPetalColor=White, MinorPetalColor_Absent=Brown, MinorPetalColor=Brown, PetalColorGroup1=WhiteBrown, PetalColorGroup2=WhiteBrown]

E:\QE Data\2019MAR12_Jessa\190312QE_034.raw

[S35] 190312QE_035 [Sample number=25, Population=F2, Female P=Gelber, Male P=P47-1, Color group=White with patterns, Floral organ=W, Secondary color=yellow, General main color=white, Main color=white, General secondary color=brown, ColorCategory=WhiteYellow, Red=No, Brown=No, Yellow=Yes, Purple=No, White=Yes, Pink=No, ColorCategories_Updated=WhiteYellow, FlowerCategories_W_ST=WhiteYellow, MainPetalColor=White, MinorPetalColor_Absent=Yellow, MinorPetalColor=Yellow, PetalColorGroup1=WhiteYellow, PetalColorGroup2=WhiteYellow]

E:\QE Data\2019MAR12_Jessa\190312QE_035.raw

[S36] 190312QE_036 [Sample number=n/a, Population=n/a, Female P=n/a, Male P=n/a, Color group=n/a, Floral organ=n/a, Secondary color=n/a, General main color=n/a, Main color=n/a, General secondary color=n/a, ColorCategory=n/a, Red=n/a, Brown=n/a, Yellow=n/a, Purple=n/a, White=n/a, Pink=n/a, ColorCategories_Updated=n/a, FlowerCategories_W_ST=n/a, MainPetalColor=n/a, MinorPetalColor_Absent=n/a, MinorPetalColor=n/a, PetalColorGroup1=n/a, PetalColorGroup2=n/a]

E:\QE Data\2019MAR12_Jessa\190312QE_036.raw

[S37] 190312QE_037 [Sample number=n/a, Population=n/a, Female P=n/a, Male P=n/a, Color group=n/a, Floral organ=n/a, Secondary color=n/a, General main color=n/a, Main color=n/a, General secondary color=n/a, ColorCategory=n/a, Red=n/a, Brown=n/a, Yellow=n/a, Purple=n/a, White=n/a, Pink=n/a, ColorCategories_Updated=n/a, FlowerCategories_W_ST=n/a, MainPetalColor=n/a, MinorPetalColor_Absent=n/a, MinorPetalColor=n/a, PetalColorGroup1=n/a, PetalColorGroup2=n/a]

E:\QE Data\2019MAR12_Jessa\190312QE_037.raw

[S38] 190312QE_038 [Sample number=29, Population=F2, Female P=P47-1, Male P=Snowdrop, Color group=White with patterns, Floral organ=W, Secondary color=brown, General main color=white, Main color=white, General secondary color=brown, ColorCategory=WhiteBrown, Red=No, Brown=Yes, Yellow=No, Purple=No, White=Yes, Pink=No, ColorCategories_Updated=WhiteBrown, FlowerCategories_W_ST=WhiteBrown, MainPetalColor=White, MinorPetalColor_Absent=Brown, MinorPetalColor=Brown, PetalColorGroup1=WhiteBrown, PetalColorGroup2=WhiteBrown]

E:\QE Data\2019MAR12_Jessa\190312QE_038.raw

[S39] 190312QE_039 [Sample number=37, Population=F2, Female P=P47-1, Male P=Disco/2, Color group=White with patterns, Floral organ=W, Secondary color=brown, General main color=white, Main color=white, General secondary color=brown, ColorCategory=WhiteBrown, Red=No, Brown=Yes, Yellow=No, Purple=No, White=Yes, Pink=No, ColorCategories_Updated=WhiteBrown, FlowerCategories_W_ST=WhiteBrown, MainPetalColor=White, MinorPetalColor_Absent=Brown, MinorPetalColor=Brown, PetalColorGroup1=WhiteBrown, PetalColorGroup2=WhiteBrown]

E:\QE Data\2019MAR12_Jessa\190312QE_039.raw

[S40] 190312QE_040 [Sample number=38, Population=F2, Female P=P47-1, Male P=Disco/2, Color group=White with patterns, Floral organ=ST, Secondary color=brown, General main color=white, Main color=white, General secondary color=brown, ColorCategory=WhiteBrown, Red=No, Brown=Yes, Yellow=No, Purple=No, White=Yes, Pink=No, ColorCategories_Updated=WhiteBrown, FlowerCategories_W_ST=WhiteBrown, MainPetalColor=White, MinorPetalColor_Absent=Brown, MinorPetalColor=Brown, PetalColorGroup1=WhiteBrown, PetalColorGroup2=WhiteBrown]

E:\QE Data\2019MAR12_Jessa\190312QE_040.raw

[S41] 190312QE_041 [Sample number=39, Population=F2, Female P=P47-1, Male P=Disco/2, Color group=White with patterns, Floral organ=W, Secondary color=brown, General main color=white, Main color=white, General secondary color=brown, ColorCategory=WhiteBrown, Red=No, Brown=Yes, Yellow=No, Purple=No, White=Yes, Pink=No, ColorCategories_Updated=WhiteBrown, FlowerCategories_W_ST=WhiteBrown, MainPetalColor=White, MinorPetalColor_Absent=Brown, MinorPetalColor=Brown, PetalColorGroup1=WhiteBrown, PetalColorGroup2=WhiteBrown]

E:\QE Data\2019MAR12_Jessa\190312QE_041.raw

[S42] 190312QE_042 [Sample number=40, Population=F2, Female P=P47-1, Male P=Disco/2, Color group=White with patterns, Floral organ=ST, Secondary color=brown, General main color=white, Main color=white, General secondary color=brown, ColorCategory=WhiteBrown, Red=No, Brown=Yes, Yellow=No, Purple=No, White=Yes, Pink=No, ColorCategories_Updated=WhiteBrown, FlowerCategories_W_ST=WhiteBrown, MainPetalColor=White, MinorPetalColor_Absent=Brown, MinorPetalColor=Brown, PetalColorGroup1=WhiteBrown, PetalColorGroup2=WhiteBrown]

E:\QE Data\2019MAR12_Jessa\190312QE_042.raw

[S43] 190312QE_043 [Sample number=47, Population=F2, Female P=Snowdrop, Male P=Gelber, Color group=White with patterns, Floral organ=W, Secondary color=brown, General main color=white, Main color=white, General secondary color=brown, ColorCategory=WhiteBrown, Red=No, Brown=Yes, Yellow=No, Purple=No, White=Yes, Pink=No, ColorCategories_Updated=WhiteBrown, FlowerCategories_W_ST=WhiteBrown, MainPetalColor=White, MinorPetalColor_Absent=Brown, MinorPetalColor=Brown, PetalColorGroup1=WhiteBrown, PetalColorGroup2=WhiteBrown]

E:\QE Data\2019MAR12_Jessa\190312QE_043.raw

[S44] 190312QE_044 [Sample number=n/a, Population=n/a, Female P=n/a, Male P=n/a, Color group=n/a, Floral organ=n/a, Secondary color=n/a, General main color=n/a, Main color=n/a, General secondary color=n/a, ColorCategory=n/a, Red=n/a, Brown=n/a, Yellow=n/a, Purple=n/a, White=n/a, Pink=n/a, ColorCategories_Updated=n/a, FlowerCategories_W_ST=n/a, MainPetalColor=n/a, MinorPetalColor_Absent=n/a, MinorPetalColor=n/a, PetalColorGroup1=n/a, PetalColorGroup2=n/a]

E:\QE Data\2019MAR12_Jessa\190312QE_044.raw

[S45] 190312QE_045 [Sample number=n/a, Population=n/a, Female P=n/a, Male P=n/a, Color group=n/a, Floral organ=n/a, Secondary color=n/a, General main color=n/a, Main color=n/a, General secondary color=n/a, ColorCategory=n/a, Red=n/a, Brown=n/a, Yellow=n/a, Purple=n/a, White=n/a, Pink=n/a, ColorCategories_Updated=n/a, FlowerCategories_W_ST=n/a, MainPetalColor=n/a, MinorPetalColor_Absent=n/a, MinorPetalColor=n/a, PetalColorGroup1=n/a, PetalColorGroup2=n/a]

E:\QE Data\2019MAR12_Jessa\190312QE_045.raw

[S46] 190312QE_046 [Sample number=48, Population=F2, Female P=Snowdrop, Male P=Gelber, Color group=White with patterns, Floral organ=ST, Secondary color=brown, General main color=white, Main color=white, General secondary color=brown, ColorCategory=WhiteBrown, Red=No, Brown=Yes, Yellow=No, Purple=No, White=Yes, Pink=No, ColorCategories_Updated=WhiteBrown, FlowerCategories_W_ST=WhiteBrown, MainPetalColor=White, MinorPetalColor_Absent=Brown, MinorPetalColor=Brown, PetalColorGroup1=WhiteBrown, PetalColorGroup2=WhiteBrown]

E:\QE Data\2019MAR12_Jessa\190312QE_046.raw

[S49] 190312QE_049 [Sample number=51, Population=F2, Female P=Disco/2, Male P=Gelber, Color group=White with patterns, Floral organ=W, Secondary color=yellow, General main color=white, Main color=white, General secondary color=brown, ColorCategory=WhiteYellow, Red=No, Brown=No, Yellow=Yes, Purple=No, White=Yes, Pink=No, ColorCategories_Updated=WhiteYellow, FlowerCategories_W_ST=WhiteYellow, MainPetalColor=White, MinorPetalColor_Absent=Yellow, MinorPetalColor=Yellow, PetalColorGroup1=WhiteYellow, PetalColorGroup2=WhiteYellow]

E:\QE Data\2019MAR12_Jessa\190312QE_049.raw

[S50] 190312QE_050 [Sample number=55, Population=F2, Female P=Disco/2, Male P=Gelber, Color group=White with patterns, Floral organ=W, Secondary color=brown, General main color=white, Main color=white, General secondary color=brown, ColorCategory=WhiteBrown, Red=No, Brown=Yes, Yellow=No, Purple=No, White=Yes, Pink=No, ColorCategories_Updated=WhiteBrown, FlowerCategories_W_ST=WhiteBrown, MainPetalColor=White, MinorPetalColor_Absent=Brown, MinorPetalColor=Brown, PetalColorGroup1=WhiteBrown, PetalColorGroup2=WhiteBrown]

E:\QE Data\2019MAR12_Jessa\190312QE_050.raw

[S51] 190312QE_051 [Sample number=56, Population=F2, Female P=Disco/2, Male P=Gelber, Color group=White with patterns, Floral organ=ST, Secondary color=brown, General main color=white, Main color=white, General secondary color=brown, ColorCategory=WhiteBrown, Red=No, Brown=Yes, Yellow=No, Purple=No, White=Yes, Pink=No, ColorCategories_Updated=WhiteBrown, FlowerCategories_W_ST=WhiteBrown, MainPetalColor=White, MinorPetalColor_Absent=Brown, MinorPetalColor=Brown, PetalColorGroup1=WhiteBrown, PetalColorGroup2=WhiteBrown]

E:\QE Data\2019MAR12_Jessa\190312QE_051.raw

[S52] 190312QE_052 [Sample number=n/a, Population=n/a, Female P=n/a, Male P=n/a, Color group=n/a, Floral organ=n/a, Secondary color=n/a, General main color=n/a, Main color=n/a, General secondary color=n/a, ColorCategory=n/a, Red=n/a, Brown=n/a, Yellow=n/a, Purple=n/a, White=n/a, Pink=n/a, ColorCategories_Updated=n/a, FlowerCategories_W_ST=n/a, MainPetalColor=n/a, MinorPetalColor_Absent=n/a, MinorPetalColor=n/a, PetalColorGroup1=n/a, PetalColorGroup2=n/a]

E:\QE Data\2019MAR12_Jessa\190312QE_052.raw

[S53] 190312QE_053 [Sample number=n/a, Population=n/a, Female P=n/a, Male P=n/a, Color group=n/a, Floral organ=n/a, Secondary color=n/a, General main color=n/a, Main color=n/a, General secondary color=n/a, ColorCategory=n/a, Red=n/a, Brown=n/a, Yellow=n/a, Purple=n/a, White=n/a, Pink=n/a, ColorCategories_Updated=n/a, FlowerCategories_W_ST=n/a, MainPetalColor=n/a, MinorPetalColor_Absent=n/a, MinorPetalColor=n/a, PetalColorGroup1=n/a, PetalColorGroup2=n/a]

E:\QE Data\2019MAR12_Jessa\190312QE_053.raw

[S54] 190312QE_054 [Sample number=57, Population=F2, Female P=Aurora, Male P=Gelber, Color group=White with patterns, Floral organ=W, Secondary color=brown, General main color=white, Main color=white, General secondary color=brown, ColorCategory=WhiteBrown, Red=No, Brown=Yes, Yellow=No, Purple=No, White=Yes, Pink=No, ColorCategories_Updated=WhiteBrown, FlowerCategories_W_ST=WhiteBrown, MainPetalColor=White, MinorPetalColor_Absent=Brown, MinorPetalColor=Brown, PetalColorGroup1=WhiteBrown, PetalColorGroup2=WhiteBrown]

E:\QE Data\2019MAR12_Jessa\190312QE_054.raw

[S55] 190312QE_055 [Sample number=58, Population=F2, Female P=Aurora, Male P=Gelber, Color group=White with patterns, Floral organ=ST, Secondary color=brown, General main color=white, Main color=white, General secondary color=brown, ColorCategory=WhiteBrown, Red=No, Brown=Yes, Yellow=No, Purple=No, White=Yes, Pink=No, ColorCategories_Updated=WhiteBrown, FlowerCategories_W_ST=WhiteBrown, MainPetalColor=White, MinorPetalColor_Absent=Brown, MinorPetalColor=Brown, PetalColorGroup1=WhiteBrown, PetalColorGroup2=WhiteBrown]

E:\QE Data\2019MAR12_Jessa\190312QE_055.raw

[S57] 190312QE_057 [Sample number=60, Population=F2, Female P=Aurora, Male P=Gelber, Color group=White with patterns, Floral organ=ST, Secondary color=yellow, General main color=white, Main color=white, General secondary color=brown, ColorCategory=WhiteYellow, Red=No, Brown=No, Yellow=Yes, Purple=No, White=Yes, Pink=No, ColorCategories_Updated=WhiteYellow, FlowerCategories_W_ST=WhiteYellow, MainPetalColor=White, MinorPetalColor_Absent=Yellow, MinorPetalColor=Yellow, PetalColorGroup1=WhiteYellow, PetalColorGroup2=WhiteYellow]

E:\QE Data\2019MAR12_Jessa\190312QE_057.raw

[S58] 190312QE_058 [Sample number=65, Population=F2, Female P=Aurora, Male P=P47-1, Color group=White with patterns, Floral organ=W, Secondary color=brown, General main color=white, Main color=white, General secondary color=brown, ColorCategory=WhiteBrown, Red=No, Brown=Yes, Yellow=No, Purple=No, White=Yes, Pink=No, ColorCategories_Updated=WhiteBrown, FlowerCategories_W_ST=WhiteBrown, MainPetalColor=White, MinorPetalColor_Absent=Brown, MinorPetalColor=Brown, PetalColorGroup1=WhiteBrown, PetalColorGroup2=WhiteBrown]

E:\QE Data\2019MAR12_Jessa\190312QE_058.raw

[S59] 190312QE_059 [Sample number=66, Population=F2, Female P=Aurora, Male P=P47-1, Color group=White with patterns, Floral organ=ST, Secondary color=brown, General main color=white, Main color=white, General secondary color=brown, ColorCategory=WhiteBrown, Red=No, Brown=Yes, Yellow=No, Purple=No, White=Yes, Pink=No, ColorCategories_Updated=WhiteBrown, FlowerCategories_W_ST=WhiteBrown, MainPetalColor=White, MinorPetalColor_Absent=Brown, MinorPetalColor=Brown, PetalColorGroup1=WhiteBrown, PetalColorGroup2=WhiteBrown]

E:\QE Data\2019MAR12_Jessa\190312QE_059.raw

[S60] 190312QE_060 [Sample number=n/a, Population=n/a, Female P=n/a, Male P=n/a, Color group=n/a, Floral organ=n/a, Secondary color=n/a, General main color=n/a, Main color=n/a, General secondary color=n/a, ColorCategory=n/a, Red=n/a, Brown=n/a, Yellow=n/a, Purple=n/a, White=n/a, Pink=n/a, ColorCategories_Updated=n/a, FlowerCategories_W_ST=n/a, MainPetalColor=n/a, MinorPetalColor_Absent=n/a, MinorPetalColor=n/a, PetalColorGroup1=n/a, PetalColorGroup2=n/a]

E:\QE Data\2019MAR12_Jessa\190312QE_060.raw

[S61] 190312QE_061 [Sample number=n/a, Population=n/a, Female P=n/a, Male P=n/a, Color group=n/a, Floral organ=n/a, Secondary color=n/a, General main color=n/a, Main color=n/a, General secondary color=n/a, ColorCategory=n/a, Red=n/a, Brown=n/a, Yellow=n/a, Purple=n/a, White=n/a, Pink=n/a, ColorCategories_Updated=n/a, FlowerCategories_W_ST=n/a, MainPetalColor=n/a, MinorPetalColor_Absent=n/a, MinorPetalColor=n/a, PetalColorGroup1=n/a, PetalColorGroup2=n/a]

E:\QE Data\2019MAR12_Jessa\190312QE_061.raw

[S66] 190312QE_066 [Sample number=n/a, Population=n/a, Female P=n/a, Male P=n/a, Color group=n/a, Floral organ=n/a, Secondary color=n/a, General main color=n/a, Main color=n/a, General secondary color=n/a, ColorCategory=n/a, Red=n/a, Brown=n/a, Yellow=n/a, Purple=n/a, White=n/a, Pink=n/a, ColorCategories_Updated=n/a, FlowerCategories_W_ST=n/a, MainPetalColor=n/a, MinorPetalColor_Absent=n/a, MinorPetalColor=n/a, PetalColorGroup1=n/a, PetalColorGroup2=n/a]

E:\QE Data\2019MAR12_Jessa\190312QE_066.raw

[S67] 190312QE_067 [Sample number=13, Population=F2, Female P=Snowdrop, Male P=P47-1, Color group=Red, Floral organ=ST, Secondary color=red, General main color=red, Main color=red, General secondary color=red, ColorCategory=Red, Red=Yes, Brown=No, Yellow=No, Purple=No, White=No, Pink=No, ColorCategories_Updated=Red, FlowerCategories_W_ST=BrownRed, MainPetalColor=Red, MinorPetalColor_Absent=Absent, MinorPetalColor=Red, PetalColorGroup1=Red, PetalColorGroup2=Red]

E:\QE Data\2019MAR12_Jessa\190312QE_067.raw

[S68] 190312QE_068 [Sample number=n/a, Population=n/a, Female P=n/a, Male P=n/a, Color group=n/a, Floral organ=n/a, Secondary color=n/a, General main color=n/a, Main color=n/a, General secondary color=n/a, ColorCategory=n/a, Red=n/a, Brown=n/a, Yellow=n/a, Purple=n/a, White=n/a, Pink=n/a, ColorCategories_Updated=n/a, FlowerCategories_W_ST=n/a, MainPetalColor=n/a, MinorPetalColor_Absent=n/a, MinorPetalColor=n/a, PetalColorGroup1=n/a, PetalColorGroup2=n/a]

E:\QE Data\2019MAR12_Jessa\190312QE_068.raw

[S69] 190312QE_069 [Sample number=n/a, Population=n/a, Female P=n/a, Male P=n/a, Color group=n/a, Floral organ=n/a, Secondary color=n/a, General main color=n/a, Main color=n/a, General secondary color=n/a, ColorCategory=n/a, Red=n/a, Brown=n/a, Yellow=n/a, Purple=n/a, White=n/a, Pink=n/a, ColorCategories_Updated=n/a, FlowerCategories_W_ST=n/a, MainPetalColor=n/a, MinorPetalColor_Absent=n/a, MinorPetalColor=n/a, PetalColorGroup1=n/a, PetalColorGroup2=n/a]

E:\QE Data\2019MAR12_Jessa\190312QE_069.raw

[S70] 190312QE_070 [Sample number=14, Population=F2, Female P=Snowdrop, Male P=P47-1, Color group=Red, Floral organ=W, Secondary color=red, General main color=brown, Main color=brown, General secondary color=red, ColorCategory=RedBrown, Red=Yes, Brown=Yes, Yellow=No, Purple=No, White=No, Pink=No, ColorCategories_Updated=BrownRed, FlowerCategories_W_ST=BrownRed, MainPetalColor=Brown, MinorPetalColor_Absent=Red, MinorPetalColor=Red, PetalColorGroup1=BrownRed, PetalColorGroup2=BrownRed-RedBrown]

E:\QE Data\2019MAR12_Jessa\190312QE_070.raw

[S71] 190312QE_071 [Sample number=17, Population=F2, Female P=Snowdrop, Male P=P47-1, Color group=Red, Floral organ=W, Secondary color=brown, General main color=red, Main color=red, General secondary color=brown, ColorCategory=RedBrown, Red=Yes, Brown=Yes, Yellow=No, Purple=No, White=No, Pink=No, ColorCategories_Updated=BrownRed, FlowerCategories_W_ST=BrownRed, MainPetalColor=Red, MinorPetalColor_Absent=Brown, MinorPetalColor=Brown, PetalColorGroup1=RedBrown, PetalColorGroup2=BrownRed-RedBrown]

E:\QE Data\2019MAR12_Jessa\190312QE_071.raw

[S72] 190312QE_072 [Sample number=18, Population=F2, Female P=Snowdrop, Male P=P47-1, Color group=Red, Floral organ=ST, Secondary color=brown, General main color=red, Main color=red, General secondary color=brown, ColorCategory=RedBrown, Red=Yes, Brown=Yes, Yellow=No, Purple=No, White=No, Pink=No, ColorCategories_Updated=BrownRed, FlowerCategories_W_ST=BrownRed, MainPetalColor=Red, MinorPetalColor_Absent=Brown, MinorPetalColor=Brown, PetalColorGroup1=RedBrown, PetalColorGroup2=BrownRed-RedBrown]

E:\QE Data\2019MAR12_Jessa\190312QE_072.raw

[S75] 190312QE_075 [Sample number=21, Population=F2, Female P=Snowdrop, Male P=P47-1, Color group=Red, Floral organ=W, Secondary color=brown, General main color=red, Main color=red, General secondary color=brown, ColorCategory=RedBrown, Red=Yes, Brown=Yes, Yellow=No, Purple=No, White=No, Pink=No, ColorCategories_Updated=BrownRed, FlowerCategories_W_ST=BrownRed, MainPetalColor=Red, MinorPetalColor_Absent=Brown, MinorPetalColor=Brown, PetalColorGroup1=RedBrown, PetalColorGroup2=BrownRed-RedBrown]

E:\QE Data\2019MAR12_Jessa\190312QE_075.raw

[S76] 190312QE_076 [Sample number=n/a, Population=n/a, Female P=n/a, Male P=n/a, Color group=n/a, Floral organ=n/a, Secondary color=n/a, General main color=n/a, Main color=n/a, General secondary color=n/a, ColorCategory=n/a, Red=n/a, Brown=n/a, Yellow=n/a, Purple=n/a, White=n/a, Pink=n/a, ColorCategories_Updated=n/a, FlowerCategories_W_ST=n/a, MainPetalColor=n/a, MinorPetalColor_Absent=n/a, MinorPetalColor=n/a, PetalColorGroup1=n/a, PetalColorGroup2=n/a]

E:\QE Data\2019MAR12_Jessa\190312QE_076.raw

[S77] 190312QE_077 [Sample number=n/a, Population=n/a, Female P=n/a, Male P=n/a, Color group=n/a, Floral organ=n/a, Secondary color=n/a, General main color=n/a, Main color=n/a, General secondary color=n/a, ColorCategory=n/a, Red=n/a, Brown=n/a, Yellow=n/a, Purple=n/a, White=n/a, Pink=n/a, ColorCategories_Updated=n/a, FlowerCategories_W_ST=n/a, MainPetalColor=n/a, MinorPetalColor_Absent=n/a, MinorPetalColor=n/a, PetalColorGroup1=n/a, PetalColorGroup2=n/a]

E:\QE Data\2019MAR12_Jessa\190312QE_077.raw

[S78] 190312QE_078 [Sample number=22, Population=F2, Female P=Snowdrop, Male P=P47-1, Color group=Red, Floral organ=ST, Secondary color=red, General main color=red, Main color=red, General secondary color=red, ColorCategory=Red, Red=Yes, Brown=No, Yellow=No, Purple=No, White=No, Pink=No, ColorCategories_Updated=Red, FlowerCategories_W_ST=BrownRed, MainPetalColor=Red, MinorPetalColor_Absent=Absent, MinorPetalColor=Red, PetalColorGroup1=Red, PetalColorGroup2=Red]

E:\QE Data\2019MAR12_Jessa\190312QE_078.raw

[S80] 190312QE_080 [Sample number=61, Population=F2, Female P=Aurora, Male P=P47-1, Color group=Red, Floral organ=W, Secondary color=brown, General main color=red, Main color=red, General secondary color=brown, ColorCategory=RedBrown, Red=Yes, Brown=Yes, Yellow=No, Purple=No, White=No, Pink=No, ColorCategories_Updated=BrownRed, FlowerCategories_W_ST=BrownRed, MainPetalColor=Red, MinorPetalColor_Absent=Brown, MinorPetalColor=Brown, PetalColorGroup1=RedBrown, PetalColorGroup2=BrownRed-RedBrown]

E:\QE Data\2019MAR12_Jessa\190312QE_080.raw

[S81] 190312QE_081 [Sample number=62, Population=F2, Female P=Aurora, Male P=P47-1, Color group=Red, Floral organ=ST, Secondary color=red, General main color=red, Main color=red, General secondary color=red, ColorCategory=Red, Red=Yes, Brown=No, Yellow=No, Purple=No, White=No, Pink=No, ColorCategories_Updated=Red, FlowerCategories_W_ST=BrownRed, MainPetalColor=Red, MinorPetalColor_Absent=Absent, MinorPetalColor=Red, PetalColorGroup1=Red, PetalColorGroup2=Red]

E:\QE Data\2019MAR12_Jessa\190312QE_081.raw

[S82] 190312QE_082 [Sample number=71, Population=2263, Female P=Rinrei, Male P=P47-1, Color group=Red, Floral organ=ST, Secondary color=purple, General main color=red, Main color=red, General secondary color=purple, ColorCategory=RedPurple, Red=Yes, Brown=No, Yellow=No, Purple=Yes, White=No, Pink=No, ColorCategories_Updated=RedPurple, FlowerCategories_W_ST=RedPurple, MainPetalColor=Purplish red, MinorPetalColor_Absent=Absent, MinorPetalColor=Purplish red, PetalColorGroup1=Purplish red, PetalColorGroup2=Purplish red]

E:\QE Data\2019MAR12_Jessa\190312QE_082.raw

[S83] 190312QE_083 [Sample number=72, Population=2263, Female P=Rinrei, Male P=P47-1, Color group=Red, Floral organ=W, Secondary color=purple, General main color=red, Main color=red, General secondary color=purple, ColorCategory=RedPurple, Red=Yes, Brown=No, Yellow=No, Purple=Yes, White=No, Pink=No, ColorCategories_Updated=RedPurple, FlowerCategories_W_ST=RedPurple, MainPetalColor=Purplish red, MinorPetalColor_Absent=Absent, MinorPetalColor=Purplish red, PetalColorGroup1=Purplish red, PetalColorGroup2=Purplish red]

E:\QE Data\2019MAR12_Jessa\190312QE_083.raw

[S84] 190312QE_084 [Sample number=n/a, Population=n/a, Female P=n/a, Male P=n/a, Color group=n/a, Floral organ=n/a, Secondary color=n/a, General main color=n/a, Main color=n/a, General secondary color=n/a, ColorCategory=n/a, Red=n/a, Brown=n/a, Yellow=n/a, Purple=n/a, White=n/a, Pink=n/a, ColorCategories_Updated=n/a, FlowerCategories_W_ST=n/a, MainPetalColor=n/a, MinorPetalColor_Absent=n/a, MinorPetalColor=n/a, PetalColorGroup1=n/a, PetalColorGroup2=n/a]

E:\QE Data\2019MAR12_Jessa\190312QE_084.raw

[S85] 190312QE_085 [Sample number=n/a, Population=n/a, Female P=n/a, Male P=n/a, Color group=n/a, Floral organ=n/a, Secondary color=n/a, General main color=n/a, Main color=n/a, General secondary color=n/a, ColorCategory=n/a, Red=n/a, Brown=n/a, Yellow=n/a, Purple=n/a, White=n/a, Pink=n/a, ColorCategories_Updated=n/a, FlowerCategories_W_ST=n/a, MainPetalColor=n/a, MinorPetalColor_Absent=n/a, MinorPetalColor=n/a, PetalColorGroup1=n/a, PetalColorGroup2=n/a]

E:\QE Data\2019MAR12_Jessa\190312QE_085.raw

[S86] 190312QE_086 [Sample number=75, Population=71B, Female P=P47-1, Male P=NV153, Color group=Red, Floral organ=W, Secondary color=purple, General main color=red, Main color=red, General secondary color=purple, ColorCategory=RedPurple, Red=Yes, Brown=No, Yellow=No, Purple=Yes, White=No, Pink=No, ColorCategories_Updated=RedPurple, FlowerCategories_W_ST=RedPurple, MainPetalColor=Purplish red, MinorPetalColor_Absent=Absent, MinorPetalColor=Purplish red, PetalColorGroup1=Purplish red, PetalColorGroup2=Purplish red]

E:\QE Data\2019MAR12_Jessa\190312QE_086.raw

[S87] 190312QE_087 [Sample number=76, Population=71B, Female P=P47-1, Male P=NV153, Color group=Red, Floral organ=ST, Secondary color=purple, General main color=red, Main color=red, General secondary color=purple, ColorCategory=RedPurple, Red=Yes, Brown=No, Yellow=No, Purple=Yes, White=No, Pink=No, ColorCategories_Updated=RedPurple, FlowerCategories_W_ST=RedPurple, MainPetalColor=Purplish red, MinorPetalColor_Absent=Absent, MinorPetalColor=Purplish red, PetalColorGroup1=Purplish red, PetalColorGroup2=Purplish red]

E:\QE Data\2019MAR12_Jessa\190312QE_087.raw

[S88] 190312QE_088 [Sample number=n/a, Population=n/a, Female P=n/a, Male P=n/a, Color group=n/a, Floral organ=n/a, Secondary color=n/a, General main color=n/a, Main color=n/a, General secondary color=n/a, ColorCategory=n/a, Red=n/a, Brown=n/a, Yellow=n/a, Purple=n/a, White=n/a, Pink=n/a, ColorCategories_Updated=n/a, FlowerCategories_W_ST=n/a, MainPetalColor=n/a, MinorPetalColor_Absent=n/a, MinorPetalColor=n/a, PetalColorGroup1=n/a, PetalColorGroup2=n/a]

E:\QE Data\2019MAR12_Jessa\190312QE_088.raw

[S89] 190312QE_089 [Sample number=5, Population=F2, Female P=Gelber, Male P=P47-1, Color group=Brown mixed, Floral organ=ST, Secondary color=red, General main color=brown, Main color=brown, General secondary color=red, ColorCategory=RedBrown, Red=Yes, Brown=Yes, Yellow=No, Purple=No, White=No, Pink=No, ColorCategories_Updated=BrownRed, FlowerCategories_W_ST=BrownRed, MainPetalColor=Brown, MinorPetalColor_Absent=Red, MinorPetalColor=Red, PetalColorGroup1=BrownRed, PetalColorGroup2=BrownRed-RedBrown]

E:\QE Data\2019MAR12_Jessa\190312QE_089.raw

[S90] 190312QE_090 [Sample number=7, Population=F2, Female P=Gelber, Male P=P47-1, Color group=Brown mixed, Floral organ=W, Secondary color=pink, General main color=brown, Main color=brown, General secondary color=red, ColorCategory=RedBrown, Red=No, Brown=Yes, Yellow=No, Purple=No, White=No, Pink=Yes, ColorCategories_Updated=BrownPink, FlowerCategories_W_ST=BrownPink, MainPetalColor=Brown, MinorPetalColor_Absent=Pink, MinorPetalColor=Pink, PetalColorGroup1=BrownPink, PetalColorGroup2=BrownPink-PinkBrown]

E:\QE Data\2019MAR12_Jessa\190312QE_090.raw

[S91] 190312QE_091 [Sample number=8, Population=F2, Female P=Gelber, Male P=P47-1, Color group=Brown mixed, Floral organ=ST, Secondary color=brown, General main color=red, Main color=pink, General secondary color=brown, ColorCategory=RedBrown, Red=No, Brown=Yes, Yellow=No, Purple=No, White=No, Pink=Yes, ColorCategories_Updated=BrownPink, FlowerCategories_W_ST=BrownPink, MainPetalColor=Pink, MinorPetalColor_Absent=Brown, MinorPetalColor=Brown, PetalColorGroup1=PinkBrown, PetalColorGroup2=BrownPink-PinkBrown]

E:\QE Data\2019MAR12_Jessa\190312QE_091.raw

[S92] 190312QE_092 [Sample number=n/a, Population=n/a, Female P=n/a, Male P=n/a, Color group=n/a, Floral organ=n/a, Secondary color=n/a, General main color=n/a, Main color=n/a, General secondary color=n/a, ColorCategory=n/a, Red=n/a, Brown=n/a, Yellow=n/a, Purple=n/a, White=n/a, Pink=n/a, ColorCategories_Updated=n/a, FlowerCategories_W_ST=n/a, MainPetalColor=n/a, MinorPetalColor_Absent=n/a, MinorPetalColor=n/a, PetalColorGroup1=n/a, PetalColorGroup2=n/a]

E:\QE Data\2019MAR12_Jessa\190312QE_092.raw

[S93] 190312QE_093 [Sample number=n/a, Population=n/a, Female P=n/a, Male P=n/a, Color group=n/a, Floral organ=n/a, Secondary color=n/a, General main color=n/a, Main color=n/a, General secondary color=n/a, ColorCategory=n/a, Red=n/a, Brown=n/a, Yellow=n/a, Purple=n/a, White=n/a, Pink=n/a, ColorCategories_Updated=n/a, FlowerCategories_W_ST=n/a, MainPetalColor=n/a, MinorPetalColor_Absent=n/a, MinorPetalColor=n/a, PetalColorGroup1=n/a, PetalColorGroup2=n/a]

E:\QE Data\2019MAR12_Jessa\190312QE_093.raw

[S94] 190312QE_094 [Sample number=9, Population=F2, Female P=Gelber, Male P=P47-1, Color group=Brown mixed, Floral organ=W, Secondary color=pink, General main color=brown, Main color=brown, General secondary color=red, ColorCategory=RedBrown, Red=No, Brown=Yes, Yellow=No, Purple=No, White=No, Pink=Yes, ColorCategories_Updated=BrownPink, FlowerCategories_W_ST=BrownPink, MainPetalColor=Brown, MinorPetalColor_Absent=Pink, MinorPetalColor=Pink, PetalColorGroup1=BrownPink, PetalColorGroup2=BrownPink-PinkBrown]

E:\QE Data\2019MAR12_Jessa\190312QE_094.raw

[S95] 190312QE_095 [Sample number=10, Population=F2, Female P=Gelber, Male P=P47-1, Color group=Brown mixed, Floral organ=ST, Secondary color=brown, General main color=red, Main color=pink, General secondary color=brown, ColorCategory=RedBrown, Red=No, Brown=Yes, Yellow=No, Purple=No, White=No, Pink=Yes, ColorCategories_Updated=BrownPink, FlowerCategories_W_ST=BrownPink, MainPetalColor=Pink, MinorPetalColor_Absent=Brown, MinorPetalColor=Brown, PetalColorGroup1=PinkBrown, PetalColorGroup2=BrownPink-PinkBrown]

E:\QE Data\2019MAR12_Jessa\190312QE_095.raw

[S96] 190312QE_096 [Sample number=11, Population=F2, Female P=Snowdrop, Male P=P47-1, Color group=Brown mixed, Floral organ=W, Secondary color=pink, General main color=brown, Main color=brown, General secondary color=red, ColorCategory=RedBrown, Red=No, Brown=Yes, Yellow=No, Purple=No, White=No, Pink=Yes, ColorCategories_Updated=BrownPink, FlowerCategories_W_ST=BrownPink, MainPetalColor=Brown, MinorPetalColor_Absent=Pink, MinorPetalColor=Pink, PetalColorGroup1=BrownPink, PetalColorGroup2=BrownPink-PinkBrown]

E:\QE Data\2019MAR12_Jessa\190312QE_096.raw

[S97] 190312QE_097 [Sample number=12, Population=F2, Female P=Snowdrop, Male P=P47-1, Color group=Brown mixed, Floral organ=ST, Secondary color=brown, General main color=red, Main color=pink, General secondary color=brown, ColorCategory=RedBrown, Red=No, Brown=Yes, Yellow=No, Purple=No, White=No, Pink=Yes, ColorCategories_Updated=BrownPink, FlowerCategories_W_ST=BrownPink, MainPetalColor=Pink, MinorPetalColor_Absent=Brown, MinorPetalColor=Brown, PetalColorGroup1=PinkBrown, PetalColorGroup2=BrownPink-PinkBrown]

E:\QE Data\2019MAR12_Jessa\190312QE_097.raw

[S98] 190312QE_098 [Sample number=31, Population=F2, Female P=P47-1, Male P=Disco/2, Color group=Brown mixed, Floral organ=W, Secondary color=brown, General main color=red, Main color=pink, General secondary color=brown, ColorCategory=RedBrown, Red=No, Brown=Yes, Yellow=No, Purple=No, White=No, Pink=Yes, ColorCategories_Updated=BrownPink, FlowerCategories_W_ST=BrownPink, MainPetalColor=Pink, MinorPetalColor_Absent=Brown, MinorPetalColor=Brown, PetalColorGroup1=PinkBrown, PetalColorGroup2=BrownPink-PinkBrown]

E:\QE Data\2019MAR12_Jessa\190312QE_098.raw

[S99] 190312QE_099 [Sample number=32, Population=F2, Female P=P47-1, Male P=Disco/2, Color group=Brown mixed, Floral organ=ST, Secondary color=brown, General main color=red, Main color=pink, General secondary color=brown, ColorCategory=RedBrown, Red=No, Brown=Yes, Yellow=No, Purple=No, White=No, Pink=Yes, ColorCategories_Updated=BrownPink, FlowerCategories_W_ST=BrownPink, MainPetalColor=Pink, MinorPetalColor_Absent=Brown, MinorPetalColor=Brown, PetalColorGroup1=PinkBrown, PetalColorGroup2=BrownPink-PinkBrown]

E:\QE Data\2019MAR12_Jessa\190312QE_099.raw

[S100] 190312QE_100 [Sample number=n/a, Population=n/a, Female P=n/a, Male P=n/a, Color group=n/a, Floral organ=n/a, Secondary color=n/a, General main color=n/a, Main color=n/a, General secondary color=n/a, ColorCategory=n/a, Red=n/a, Brown=n/a, Yellow=n/a, Purple=n/a, White=n/a, Pink=n/a, ColorCategories_Updated=n/a, FlowerCategories_W_ST=n/a, MainPetalColor=n/a, MinorPetalColor_Absent=n/a, MinorPetalColor=n/a, PetalColorGroup1=n/a, PetalColorGroup2=n/a]

E:\QE Data\2019MAR12_Jessa\190312QE_100.raw

[S101] 190312QE_101 [Sample number=n/a, Population=n/a, Female P=n/a, Male P=n/a, Color group=n/a, Floral organ=n/a, Secondary color=n/a, General main color=n/a, Main color=n/a, General secondary color=n/a, ColorCategory=n/a, Red=n/a, Brown=n/a, Yellow=n/a, Purple=n/a, White=n/a, Pink=n/a, ColorCategories_Updated=n/a, FlowerCategories_W_ST=n/a, MainPetalColor=n/a, MinorPetalColor_Absent=n/a, MinorPetalColor=n/a, PetalColorGroup1=n/a, PetalColorGroup2=n/a]

E:\QE Data\2019MAR12_Jessa\190312QE_101.raw

[S102] 190312QE_102 [Sample number=33, Population=F2, Female P=P47-1, Male P=Disco/2, Color group=Brown mixed, Floral organ=W, Secondary color=red, General main color=brown, Main color=brown, General secondary color=red, ColorCategory=RedBrown, Red=Yes, Brown=Yes, Yellow=No, Purple=No, White=No, Pink=No, ColorCategories_Updated=BrownRed, FlowerCategories_W_ST=BrownRed, MainPetalColor=Brown, MinorPetalColor_Absent=Red, MinorPetalColor=Red, PetalColorGroup1=BrownRed, PetalColorGroup2=BrownRed-RedBrown]

E:\QE Data\2019MAR12_Jessa\190312QE_102.raw

[S103] 190312QE_103 [Sample number=35, Population=F2, Female P=P47-1, Male P=Disco/2, Color group=Brown mixed, Floral organ=W, Secondary color=brown, General main color=brown, Main color=brown, General secondary color=red, ColorCategory=BrownPurple, Red=No, Brown=Yes, Yellow=No, Purple=Yes, White=No, Pink=No, ColorCategories_Updated=Brown, FlowerCategories_W_ST=Brown, MainPetalColor=Brown, MinorPetalColor_Absent=Absent, MinorPetalColor=Brown, PetalColorGroup1=Brown, PetalColorGroup2=Brown]

E:\QE Data\2019MAR12_Jessa\190312QE_103.raw

[S104] 190312QE_104 [Sample number=63, Population=F2, Female P=Aurora, Male P=P47-1, Color group=Brown mixed, Floral organ=W, Secondary color=pink, General main color=brown, Main color=brown, General secondary color=red, ColorCategory=RedBrown, Red=No, Brown=Yes, Yellow=No, Purple=No, White=No, Pink=Yes, ColorCategories_Updated=BrownPink, FlowerCategories_W_ST=BrownPink, MainPetalColor=Brown, MinorPetalColor_Absent=Pink, MinorPetalColor=Pink, PetalColorGroup1=BrownPink, PetalColorGroup2=BrownPink-PinkBrown]

E:\QE Data\2019MAR12_Jessa\190312QE_104.raw

[S105] 190312QE_105 [Sample number=64, Population=F2, Female P=Aurora, Male P=P47-1, Color group=Brown mixed, Floral organ=ST, Secondary color=pink, General main color=red, Main color=pink, General secondary color=red, ColorCategory=Red, Red=No, Brown=No, Yellow=No, Purple=No, White=No, Pink=Yes, ColorCategories_Updated=BrownPink, FlowerCategories_W_ST=BrownPink, MainPetalColor=Pink, MinorPetalColor_Absent=Absent, MinorPetalColor=Pink, PetalColorGroup1=Pink, PetalColorGroup2=BrownPink-PinkBrown]

E:\QE Data\2019MAR12_Jessa\190312QE_105.raw

[S107] 190312QE_107 [Sample number=n/a, Population=n/a, Female P=n/a, Male P=n/a, Color group=n/a, Floral organ=n/a, Secondary color=n/a, General main color=n/a, Main color=n/a, General secondary color=n/a, ColorCategory=n/a, Red=n/a, Brown=n/a, Yellow=n/a, Purple=n/a, White=n/a, Pink=n/a, ColorCategories_Updated=n/a, FlowerCategories_W_ST=n/a, MainPetalColor=n/a, MinorPetalColor_Absent=n/a, MinorPetalColor=n/a, PetalColorGroup1=n/a, PetalColorGroup2=n/a]

E:\QE Data\2019MAR12_Jessa\190312QE_107.raw

[S108] 190312QE_108 [Sample number=n/a, Population=n/a, Female P=n/a, Male P=n/a, Color group=n/a, Floral organ=n/a, Secondary color=n/a, General main color=n/a, Main color=n/a, General secondary color=n/a, ColorCategory=n/a, Red=n/a, Brown=n/a, Yellow=n/a, Purple=n/a, White=n/a, Pink=n/a, ColorCategories_Updated=n/a, FlowerCategories_W_ST=n/a, MainPetalColor=n/a, MinorPetalColor_Absent=n/a, MinorPetalColor=n/a, PetalColorGroup1=n/a, PetalColorGroup2=n/a]

E:\QE Data\2019MAR12_Jessa\190312QE_108.raw

[S109] 190312QE_109 [Sample number=1, Population=F2, Female P=Gelber, Male P=P47-1, Color group=Brown, Floral organ=W, Secondary color=brown, General main color=brown, Main color=brown, General secondary color=brown, ColorCategory=Brown, Red=No, Brown=Yes, Yellow=No, Purple=No, White=No, Pink=No, ColorCategories_Updated=Brown, FlowerCategories_W_ST=Brown, MainPetalColor=Brown, MinorPetalColor_Absent=Absent, MinorPetalColor=Brown, PetalColorGroup1=Brown, PetalColorGroup2=Brown]

E:\QE Data\2019MAR12_Jessa\190312QE_109.raw

[S110] 190312QE_110 [Sample number=2, Population=F2, Female P=Gelber, Male P=P47-1, Color group=Brown, Floral organ=ST, Secondary color=brown, General main color=brown, Main color=brown, General secondary color=brown, ColorCategory=Brown, Red=No, Brown=Yes, Yellow=No, Purple=No, White=No, Pink=No, ColorCategories_Updated=Brown, FlowerCategories_W_ST=Brown, MainPetalColor=Brown, MinorPetalColor_Absent=Absent, MinorPetalColor=Brown, PetalColorGroup1=Brown, PetalColorGroup2=Brown]

E:\QE Data\2019MAR12_Jessa\190312QE_110.raw

[S111] 190312QE_111 [Sample number=3, Population=F2, Female P=Gelber, Male P=P47-1, Color group=Brown, Floral organ=W, Secondary color=brown, General main color=brown, Main color=brown, General secondary color=brown, ColorCategory=Brown, Red=No, Brown=Yes, Yellow=No, Purple=No, White=No, Pink=No, ColorCategories_Updated=Brown, FlowerCategories_W_ST=Brown, MainPetalColor=Brown, MinorPetalColor_Absent=Absent, MinorPetalColor=Brown, PetalColorGroup1=Brown, PetalColorGroup2=Brown]

E:\QE Data\2019MAR12_Jessa\190312QE_111.raw

[S112] 190312QE_112 [Sample number=4, Population=F2, Female P=Gelber, Male P=P47-1, Color group=Brown, Floral organ=ST, Secondary color=brown, General main color=brown, Main color=brown, General secondary color=brown, ColorCategory=Brown, Red=No, Brown=Yes, Yellow=No, Purple=No, White=No, Pink=No, ColorCategories_Updated=Brown, FlowerCategories_W_ST=Brown, MainPetalColor=Brown, MinorPetalColor_Absent=Absent, MinorPetalColor=Brown, PetalColorGroup1=Brown, PetalColorGroup2=Brown]

E:\QE Data\2019MAR12_Jessa\190312QE_112.raw

[S113] 190312QE_113 [Sample number=6, Population=F2, Female P=Gelber, Male P=P47-1, Color group=Brown, Floral organ=W, Secondary color=brown, General main color=brown, Main color=brown, General secondary color=brown, ColorCategory=Brown, Red=No, Brown=Yes, Yellow=No, Purple=No, White=No, Pink=No, ColorCategories_Updated=Brown, FlowerCategories_W_ST=BrownRed, MainPetalColor=Brown, MinorPetalColor_Absent=Absent, MinorPetalColor=Brown, PetalColorGroup1=Brown, PetalColorGroup2=Brown]

E:\QE Data\2019MAR12_Jessa\190312QE_113.raw

[S114] 190312QE_114 [Sample number=36, Population=F2, Female P=P47-1, Male P=Disco/2, Color group=Brown, Floral organ=ST, Secondary color=brown, General main color=brown, Main color=brown, General secondary color=brown, ColorCategory=Brown, Red=No, Brown=Yes, Yellow=No, Purple=No, White=No, Pink=No, ColorCategories_Updated=Brown, FlowerCategories_W_ST=Brown, MainPetalColor=Brown, MinorPetalColor_Absent=Absent, MinorPetalColor=Brown, PetalColorGroup1=Brown, PetalColorGroup2=Brown]

E:\QE Data\2019MAR12_Jessa\190312QE_114.raw

[S115] 190312QE_115 [Sample number=n/a, Population=n/a, Female P=n/a, Male P=n/a, Color group=n/a, Floral organ=n/a, Secondary color=n/a, General main color=n/a, Main color=n/a, General secondary color=n/a, ColorCategory=n/a, Red=n/a, Brown=n/a, Yellow=n/a, Purple=n/a, White=n/a, Pink=n/a, ColorCategories_Updated=n/a, FlowerCategories_W_ST=n/a, MainPetalColor=n/a, MinorPetalColor_Absent=n/a, MinorPetalColor=n/a, PetalColorGroup1=n/a, PetalColorGroup2=n/a]

E:\QE Data\2019MAR12_Jessa\190312QE_115.raw

[S116] 190312QE_116 [Sample number=n/a, Population=n/a, Female P=n/a, Male P=n/a, Color group=n/a, Floral organ=n/a, Secondary color=n/a, General main color=n/a, Main color=n/a, General secondary color=n/a, ColorCategory=n/a, Red=n/a, Brown=n/a, Yellow=n/a, Purple=n/a, White=n/a, Pink=n/a, ColorCategories_Updated=n/a, FlowerCategories_W_ST=n/a, MainPetalColor=n/a, MinorPetalColor_Absent=n/a, MinorPetalColor=n/a, PetalColorGroup1=n/a, PetalColorGroup2=n/a]

E:\QE Data\2019MAR12_Jessa\190312QE_116.raw

[S118] 190312QE_118 [Sample number=2, Population=F2, Female P=Gelber, Male P=P47-1, Color group=Brown, Floral organ=ST, Secondary color=brown, General main color=brown, Main color=brown, General secondary color=brown, ColorCategory=Brown, Red=No, Brown=Yes, Yellow=No, Purple=No, White=No, Pink=No, ColorCategories_Updated=Brown, FlowerCategories_W_ST=Brown, MainPetalColor=Brown, MinorPetalColor_Absent=Absent, MinorPetalColor=Brown, PetalColorGroup1=Brown, PetalColorGroup2=Brown]

E:\QE Data\2019MAR12_Jessa\190312QE_118.raw

[S119] 190312QE_119 [Sample number=3, Population=F2, Female P=Gelber, Male P=P47-1, Color group=Brown, Floral organ=W, Secondary color=brown, General main color=brown, Main color=brown, General secondary color=brown, ColorCategory=Brown, Red=No, Brown=Yes, Yellow=No, Purple=No, White=No, Pink=No, ColorCategories_Updated=Brown, FlowerCategories_W_ST=Brown, MainPetalColor=Brown, MinorPetalColor_Absent=Absent, MinorPetalColor=Brown, PetalColorGroup1=Brown, PetalColorGroup2=Brown]

E:\QE Data\2019MAR12_Jessa\190312QE_119.raw

[S120] 190312QE_120 [Sample number=27, Population=F2, Female P=P47-1, Male P=Snowdrop, Color group=White, Floral organ=W, Secondary color=white, General main color=white, Main color=white, General secondary color=white, ColorCategory=White, Red=No, Brown=No, Yellow=No, Purple=No, White=Yes, Pink=No, ColorCategories_Updated=White, FlowerCategories_W_ST=White, MainPetalColor=White, MinorPetalColor_Absent=Absent, MinorPetalColor=White, PetalColorGroup1=White, PetalColorGroup2=White]

E:\QE Data\2019MAR12_Jessa\190312QE_120.raw

[S121] 190312QE_121 [Sample number=59, Population=F2, Female P=Aurora, Male P=Gelber, Color group=White with patterns, Floral organ=W, Secondary color=yellow, General main color=white, Main color=white, General secondary color=brown, ColorCategory=WhiteYellow, Red=No, Brown=No, Yellow=Yes, Purple=No, White=Yes, Pink=No, ColorCategories_Updated=WhiteYellow, FlowerCategories_W_ST=WhiteYellow, MainPetalColor=White, MinorPetalColor_Absent=Yellow, MinorPetalColor=Yellow, PetalColorGroup1=WhiteYellow, PetalColorGroup2=WhiteYellow]

E:\QE Data\2019MAR12_Jessa\190312QE_121.raw

[S122] 190312QE_122 [Sample number=34, Population=F2, Female P=P47-1, Male P=Disco/2, Color group=Red, Floral organ=ST, Secondary color=brown, General main color=red, Main color=red, General secondary color=brown, ColorCategory=RedBrown, Red=Yes, Brown=Yes, Yellow=No, Purple=No, White=No, Pink=No, ColorCategories_Updated=BrownRed, FlowerCategories_W_ST=BrownRed, MainPetalColor=Red, MinorPetalColor_Absent=Brown, MinorPetalColor=Brown, PetalColorGroup1=RedBrown, PetalColorGroup2=BrownRed-RedBrown]

E:\QE Data\2019MAR12_Jessa\190312QE_122.raw

[S123] 190312QE_123 [Sample number=n/a, Population=n/a, Female P=n/a, Male P=n/a, Color group=n/a, Floral organ=n/a, Secondary color=n/a, General main color=n/a, Main color=n/a, General secondary color=n/a, ColorCategory=n/a, Red=n/a, Brown=n/a, Yellow=n/a, Purple=n/a, White=n/a, Pink=n/a, ColorCategories_Updated=n/a, FlowerCategories_W_ST=n/a, MainPetalColor=n/a, MinorPetalColor_Absent=n/a, MinorPetalColor=n/a, PetalColorGroup1=n/a, PetalColorGroup2=n/a]

E:\QE Data\2019MAR12_Jessa\190312QE_123.raw

[S159] 2020Jul30_001 [Sample number=n/a, Population=n/a, Female P=n/a, Male P=n/a, Color group=n/a, Floral organ=n/a, Secondary color=n/a, General main color=n/a, Main color=n/a, General secondary color=n/a, ColorCategory=n/a, Red=n/a, Brown=n/a, Yellow=n/a, Purple=n/a, White=n/a, Pink=n/a, ColorCategories_Updated=n/a, FlowerCategories_W_ST=n/a, MainPetalColor=n/a, MinorPetalColor_Absent=n/a, MinorPetalColor=n/a, PetalColorGroup1=n/a, PetalColorGroup2=n/a]

E:\QE Data\2020Aug2_Jessa'sFlowers_SetB\2020Jul30_Jessa_2\2020Jul30_001.raw

[S160] 2020Jul30_002 [Sample number=n/a, Population=n/a, Female P=n/a, Male P=n/a, Color group=n/a, Floral organ=n/a, Secondary color=n/a, General main color=n/a, Main color=n/a, General secondary color=n/a, ColorCategory=n/a, Red=n/a, Brown=n/a, Yellow=n/a, Purple=n/a, White=n/a, Pink=n/a, ColorCategories_Updated=n/a, FlowerCategories_W_ST=n/a, MainPetalColor=n/a, MinorPetalColor_Absent=n/a, MinorPetalColor=n/a, PetalColorGroup1=n/a, PetalColorGroup2=n/a]

E:\QE Data\2020Aug2_Jessa'sFlowers_SetB\2020Jul30_Jessa_2\2020Jul30_002.raw

[S161] 2020Jul30_003 [Sample number=n/a, Population=n/a, Female P=n/a, Male P=n/a, Color group=n/a, Floral organ=n/a, Secondary color=n/a, General main color=n/a, Main color=n/a, General secondary color=n/a, ColorCategory=n/a, Red=n/a, Brown=n/a, Yellow=n/a, Purple=n/a, White=n/a, Pink=n/a, ColorCategories_Updated=n/a, FlowerCategories_W_ST=n/a, MainPetalColor=n/a, MinorPetalColor_Absent=n/a, MinorPetalColor=n/a, PetalColorGroup1=n/a, PetalColorGroup2=n/a]

E:\QE Data\2020Aug2_Jessa'sFlowers_SetB\2020Jul30_Jessa_2\2020Jul30_003.raw

[S163] 2020Jul30_005 [Sample number=n/a, Population=n/a, Female P=n/a, Male P=n/a, Color group=n/a, Floral organ=n/a, Secondary color=n/a, General main color=n/a, Main color=n/a, General secondary color=n/a, ColorCategory=n/a, Red=n/a, Brown=n/a, Yellow=n/a, Purple=n/a, White=n/a, Pink=n/a, ColorCategories_Updated=n/a, FlowerCategories_W_ST=n/a, MainPetalColor=n/a, MinorPetalColor_Absent=n/a, MinorPetalColor=n/a, PetalColorGroup1=n/a, PetalColorGroup2=n/a]

E:\QE Data\2020Aug2_Jessa'sFlowers_SetB\2020Jul30_Jessa_2\2020Jul30_005.raw

[S164] 2020Jul30_006 [Sample number=n/a, Population=n/a, Female P=n/a, Male P=n/a, Color group=n/a, Floral organ=n/a, Secondary color=n/a, General main color=n/a, Main color=n/a, General secondary color=n/a, ColorCategory=n/a, Red=n/a, Brown=n/a, Yellow=n/a, Purple=n/a, White=n/a, Pink=n/a, ColorCategories_Updated=n/a, FlowerCategories_W_ST=n/a, MainPetalColor=n/a, MinorPetalColor_Absent=n/a, MinorPetalColor=n/a, PetalColorGroup1=n/a, PetalColorGroup2=n/a]

E:\QE Data\2020Aug2_Jessa'sFlowers_SetB\2020Jul30_Jessa_2\2020Jul30_006.raw

[S165] 2020Jul30_007 [Sample number=n/a, Population=n/a, Female P=n/a, Male P=n/a, Color group=n/a, Floral organ=n/a, Secondary color=n/a, General main color=n/a, Main color=n/a, General secondary color=n/a, ColorCategory=n/a, Red=n/a, Brown=n/a, Yellow=n/a, Purple=n/a, White=n/a, Pink=n/a, ColorCategories_Updated=n/a, FlowerCategories_W_ST=n/a, MainPetalColor=n/a, MinorPetalColor_Absent=n/a, MinorPetalColor=n/a, PetalColorGroup1=n/a, PetalColorGroup2=n/a]

E:\QE Data\2020Aug2_Jessa'sFlowers_SetB\2020Jul30_Jessa_2\2020Jul30_007.raw

[S166] 2020Jul30_008 [Sample number=26, Population=F2, Female P=Gelber, Male P=P47-1, Color group=White, Floral organ=ST, Secondary color=pink, General main color=white, Main color=white, General secondary color=white, ColorCategory=White, Red=No, Brown=No, Yellow=No, Purple=No, White=Yes, Pink=Yes, ColorCategories_Updated=White, FlowerCategories_W_ST=WhiteYellow, MainPetalColor=White, MinorPetalColor_Absent=Pink, MinorPetalColor=Pink, PetalColorGroup1=WhitePink, PetalColorGroup2=WhitePink]

E:\QE Data\2020Aug2_Jessa'sFlowers_SetB\2020Jul30_Jessa_2\2020Jul30_008.raw

[S167] 2020Jul30_009 [Sample number=27, Population=F2, Female P=P47-1, Male P=Snowdrop, Color group=White, Floral organ=W, Secondary color=white, General main color=white, Main color=white, General secondary color=white, ColorCategory=White, Red=No, Brown=No, Yellow=No, Purple=No, White=Yes, Pink=No, ColorCategories_Updated=White, FlowerCategories_W_ST=White, MainPetalColor=White, MinorPetalColor_Absent=Absent, MinorPetalColor=White, PetalColorGroup1=White, PetalColorGroup2=White]

E:\QE Data\2020Aug2_Jessa'sFlowers_SetB\2020Jul30_Jessa_2\2020Jul30_009.raw

[S168] 2020Jul30_010 [Sample number=28, Population=F2, Female P=P47-1, Male P=Snowdrop, Color group=White, Floral organ=ST, Secondary color=white, General main color=white, Main color=white, General secondary color=white, ColorCategory=White, Red=No, Brown=No, Yellow=No, Purple=No, White=Yes, Pink=No, ColorCategories_Updated=White, FlowerCategories_W_ST=White, MainPetalColor=White, MinorPetalColor_Absent=Absent, MinorPetalColor=White, PetalColorGroup1=White, PetalColorGroup2=White]

E:\QE Data\2020Aug2_Jessa'sFlowers_SetB\2020Jul30_Jessa_2\2020Jul30_010.raw

[S169] 2020Jul30_011 [Sample number=30, Population=F2, Female P=P47-1, Male P=Snowdrop, Color group=White, Floral organ=ST, Secondary color=pink, General main color=white, Main color=white, General secondary color=white, ColorCategory=White, Red=No, Brown=No, Yellow=No, Purple=No, White=Yes, Pink=Yes, ColorCategories_Updated=White, FlowerCategories_W_ST=WhiteBrown, MainPetalColor=White, MinorPetalColor_Absent=Pink, MinorPetalColor=Pink, PetalColorGroup1=WhitePink, PetalColorGroup2=WhitePink]

E:\QE Data\2020Aug2_Jessa'sFlowers_SetB\2020Jul30_Jessa_2\2020Jul30_011.raw

[S172] 2020Jul30_014 [Sample number=45, Population=F2, Female P=Snowdrop, Male P=Gelber, Color group=White, Floral organ=W, Secondary color=white, General main color=white, Main color=white, General secondary color=white, ColorCategory=White, Red=No, Brown=No, Yellow=No, Purple=No, White=Yes, Pink=No, ColorCategories_Updated=White, FlowerCategories_W_ST=White, MainPetalColor=White, MinorPetalColor_Absent=Absent, MinorPetalColor=White, PetalColorGroup1=White, PetalColorGroup2=White]

E:\QE Data\2020Aug2_Jessa'sFlowers_SetB\2020Jul30_Jessa_2\2020Jul30_014.raw

[S173] 2020Jul30_015 [Sample number=46, Population=F2, Female P=Snowdrop, Male P=Gelber, Color group=White, Floral organ=ST, Secondary color=white, General main color=white, Main color=white, General secondary color=white, ColorCategory=White, Red=No, Brown=No, Yellow=No, Purple=No, White=Yes, Pink=No, ColorCategories_Updated=White, FlowerCategories_W_ST=White, MainPetalColor=White, MinorPetalColor_Absent=Absent, MinorPetalColor=White, PetalColorGroup1=White, PetalColorGroup2=White]

E:\QE Data\2020Aug2_Jessa'sFlowers_SetB\2020Jul30_Jessa_2\2020Jul30_015.raw

[S174] 2020Jul30_016 [Sample number=n/a, Population=n/a, Female P=n/a, Male P=n/a, Color group=n/a, Floral organ=n/a, Secondary color=n/a, General main color=n/a, Main color=n/a, General secondary color=n/a, ColorCategory=n/a, Red=n/a, Brown=n/a, Yellow=n/a, Purple=n/a, White=n/a, Pink=n/a, ColorCategories_Updated=n/a, FlowerCategories_W_ST=n/a, MainPetalColor=n/a, MinorPetalColor_Absent=n/a, MinorPetalColor=n/a, PetalColorGroup1=n/a, PetalColorGroup2=n/a]

E:\QE Data\2020Aug2_Jessa'sFlowers_SetB\2020Jul30_Jessa_2\2020Jul30_016.raw

[S175] 2020Jul30_017 [Sample number=n/a, Population=n/a, Female P=n/a, Male P=n/a, Color group=n/a, Floral organ=n/a, Secondary color=n/a, General main color=n/a, Main color=n/a, General secondary color=n/a, ColorCategory=n/a, Red=n/a, Brown=n/a, Yellow=n/a, Purple=n/a, White=n/a, Pink=n/a, ColorCategories_Updated=n/a, FlowerCategories_W_ST=n/a, MainPetalColor=n/a, MinorPetalColor_Absent=n/a, MinorPetalColor=n/a, PetalColorGroup1=n/a, PetalColorGroup2=n/a]

E:\QE Data\2020Aug2_Jessa'sFlowers_SetB\2020Jul30_Jessa_2\2020Jul30_017.raw

[S176] 2020Jul30_018 [Sample number=52, Population=F2, Female P=Disco/2, Male P=Gelber, Color group=White, Floral organ=ST, Secondary color=pink, General main color=white, Main color=white, General secondary color=white, ColorCategory=White, Red=No, Brown=No, Yellow=No, Purple=No, White=Yes, Pink=Yes, ColorCategories_Updated=White, FlowerCategories_W_ST=WhiteYellow, MainPetalColor=White, MinorPetalColor_Absent=Pink, MinorPetalColor=Pink, PetalColorGroup1=WhitePink, PetalColorGroup2=WhitePink]

E:\QE Data\2020Aug2_Jessa'sFlowers_SetB\2020Jul30_Jessa_2\2020Jul30_018.raw

[S177] 2020Jul30_019 [Sample number=53, Population=F2, Female P=Disco/2, Male P=Gelber, Color group=White, Floral organ=W, Secondary color=white, General main color=white, Main color=white, General secondary color=white, ColorCategory=White, Red=No, Brown=No, Yellow=No, Purple=No, White=Yes, Pink=No, ColorCategories_Updated=White, FlowerCategories_W_ST=White, MainPetalColor=White, MinorPetalColor_Absent=Absent, MinorPetalColor=White, PetalColorGroup1=White, PetalColorGroup2=White]

E:\QE Data\2020Aug2_Jessa'sFlowers_SetB\2020Jul30_Jessa_2\2020Jul30_019.raw

[S178] 2020Jul30_020 [Sample number=54, Population=F2, Female P=Disco/2, Male P=Gelber, Color group=White, Floral organ=ST, Secondary color=white, General main color=white, Main color=white, General secondary color=white, ColorCategory=White, Red=No, Brown=No, Yellow=No, Purple=No, White=Yes, Pink=No, ColorCategories_Updated=White, FlowerCategories_W_ST=White, MainPetalColor=White, MinorPetalColor_Absent=Absent, MinorPetalColor=White, PetalColorGroup1=White, PetalColorGroup2=White]

E:\QE Data\2020Aug2_Jessa'sFlowers_SetB\2020Jul30_Jessa_2\2020Jul30_020.raw

[S181] 2020Jul30_023 [Sample number=25, Population=F2, Female P=Gelber, Male P=P47-1, Color group=White with patterns, Floral organ=W, Secondary color=yellow, General main color=white, Main color=white, General secondary color=brown, ColorCategory=WhiteYellow, Red=No, Brown=No, Yellow=Yes, Purple=No, White=Yes, Pink=No, ColorCategories_Updated=WhiteYellow, FlowerCategories_W_ST=WhiteYellow, MainPetalColor=White, MinorPetalColor_Absent=Yellow, MinorPetalColor=Yellow, PetalColorGroup1=WhiteYellow, PetalColorGroup2=WhiteYellow]

E:\QE Data\2020Aug2_Jessa'sFlowers_SetB\2020Jul30_Jessa_2\2020Jul30_023.raw

[S183] 2020Jul30_025 [Sample number=51, Population=F2, Female P=Disco/2, Male P=Gelber, Color group=White with patterns, Floral organ=W, Secondary color=yellow, General main color=white, Main color=white, General secondary color=brown, ColorCategory=WhiteYellow, Red=No, Brown=No, Yellow=Yes, Purple=No, White=Yes, Pink=No, ColorCategories_Updated=WhiteYellow, FlowerCategories_W_ST=WhiteYellow, MainPetalColor=White, MinorPetalColor_Absent=Yellow, MinorPetalColor=Yellow, PetalColorGroup1=WhiteYellow, PetalColorGroup2=WhiteYellow]

E:\QE Data\2020Aug2_Jessa'sFlowers_SetB\2020Jul30_Jessa_2\2020Jul30_025.raw

[S184] 2020Jul30_026 [Sample number=59, Population=F2, Female P=Aurora, Male P=Gelber, Color group=White with patterns, Floral organ=W, Secondary color=yellow, General main color=white, Main color=white, General secondary color=brown, ColorCategory=WhiteYellow, Red=No, Brown=No, Yellow=Yes, Purple=No, White=Yes, Pink=No, ColorCategories_Updated=WhiteYellow, FlowerCategories_W_ST=WhiteYellow, MainPetalColor=White, MinorPetalColor_Absent=Yellow, MinorPetalColor=Yellow, PetalColorGroup1=WhiteYellow, PetalColorGroup2=WhiteYellow]

E:\QE Data\2020Aug2_Jessa'sFlowers_SetB\2020Jul30_Jessa_2\2020Jul30_026.raw

[S185] 2020Jul30_027 [Sample number=60, Population=F2, Female P=Aurora, Male P=Gelber, Color group=White with patterns, Floral organ=ST, Secondary color=yellow, General main color=white, Main color=white, General secondary color=brown, ColorCategory=WhiteYellow, Red=No, Brown=No, Yellow=Yes, Purple=No, White=Yes, Pink=No, ColorCategories_Updated=WhiteYellow, FlowerCategories_W_ST=WhiteYellow, MainPetalColor=White, MinorPetalColor_Absent=Yellow, MinorPetalColor=Yellow, PetalColorGroup1=WhiteYellow, PetalColorGroup2=WhiteYellow]

E:\QE Data\2020Aug2_Jessa'sFlowers_SetB\2020Jul30_Jessa_2\2020Jul30_027.raw

[S186] 2020Jul30_028 [Sample number=n/a, Population=n/a, Female P=n/a, Male P=n/a, Color group=n/a, Floral organ=n/a, Secondary color=n/a, General main color=n/a, Main color=n/a, General secondary color=n/a, ColorCategory=n/a, Red=n/a, Brown=n/a, Yellow=n/a, Purple=n/a, White=n/a, Pink=n/a, ColorCategories_Updated=n/a, FlowerCategories_W_ST=n/a, MainPetalColor=n/a, MinorPetalColor_Absent=n/a, MinorPetalColor=n/a, PetalColorGroup1=n/a, PetalColorGroup2=n/a]

E:\QE Data\2020Aug2_Jessa'sFlowers_SetB\2020Jul30_Jessa_2\2020Jul30_028.raw

[S187] 2020Jul30_029 [Sample number=n/a, Population=n/a, Female P=n/a, Male P=n/a, Color group=n/a, Floral organ=n/a, Secondary color=n/a, General main color=n/a, Main color=n/a, General secondary color=n/a, ColorCategory=n/a, Red=n/a, Brown=n/a, Yellow=n/a, Purple=n/a, White=n/a, Pink=n/a, ColorCategories_Updated=n/a, FlowerCategories_W_ST=n/a, MainPetalColor=n/a, MinorPetalColor_Absent=n/a, MinorPetalColor=n/a, PetalColorGroup1=n/a, PetalColorGroup2=n/a]

E:\QE Data\2020Aug2_Jessa'sFlowers_SetB\2020Jul30_Jessa_2\2020Jul30_029.raw

[S188] 2020Jul30_030 [Sample number=23, Population=F2, Female P=Gelber, Male P=P47-1, Color group=White with patterns, Floral organ=W, Secondary color=brown, General main color=white, Main color=white, General secondary color=brown, ColorCategory=WhiteBrown, Red=No, Brown=Yes, Yellow=No, Purple=No, White=Yes, Pink=No, ColorCategories_Updated=WhiteBrown, FlowerCategories_W_ST=WhiteBrown, MainPetalColor=White, MinorPetalColor_Absent=Brown, MinorPetalColor=Brown, PetalColorGroup1=WhiteBrown, PetalColorGroup2=WhiteBrown]

E:\QE Data\2020Aug2_Jessa'sFlowers_SetB\2020Jul30_Jessa_2\2020Jul30_030.raw

[S189] 2020Jul30_031 [Sample number=24, Population=F2, Female P=Gelber, Male P=P47-1, Color group=White with patterns, Floral organ=ST, Secondary color=brown, General main color=white, Main color=white, General secondary color=brown, ColorCategory=WhiteBrown, Red=No, Brown=Yes, Yellow=No, Purple=No, White=Yes, Pink=No, ColorCategories_Updated=WhiteBrown, FlowerCategories_W_ST=WhiteBrown, MainPetalColor=White, MinorPetalColor_Absent=Brown, MinorPetalColor=Brown, PetalColorGroup1=WhiteBrown, PetalColorGroup2=WhiteBrown]

E:\QE Data\2020Aug2_Jessa'sFlowers_SetB\2020Jul30_Jessa_2\2020Jul30_031.raw

[S190] 2020Jul30_032 [Sample number=29, Population=F2, Female P=P47-1, Male P=Snowdrop, Color group=White with patterns, Floral organ=W, Secondary color=brown, General main color=white, Main color=white, General secondary color=brown, ColorCategory=WhiteBrown, Red=No, Brown=Yes, Yellow=No, Purple=No, White=Yes, Pink=No, ColorCategories_Updated=WhiteBrown, FlowerCategories_W_ST=WhiteBrown, MainPetalColor=White, MinorPetalColor_Absent=Brown, MinorPetalColor=Brown, PetalColorGroup1=WhiteBrown, PetalColorGroup2=WhiteBrown]

E:\QE Data\2020Aug2_Jessa'sFlowers_SetB\2020Jul30_Jessa_2\2020Jul30_032.raw

[S191] 2020Jul30_033 [Sample number=37, Population=F2, Female P=P47-1, Male P=Disco/2, Color group=White with patterns, Floral organ=W, Secondary color=brown, General main color=white, Main color=white, General secondary color=brown, ColorCategory=WhiteBrown, Red=No, Brown=Yes, Yellow=No, Purple=No, White=Yes, Pink=No, ColorCategories_Updated=WhiteBrown, FlowerCategories_W_ST=WhiteBrown, MainPetalColor=White, MinorPetalColor_Absent=Brown, MinorPetalColor=Brown, PetalColorGroup1=WhiteBrown, PetalColorGroup2=WhiteBrown]

E:\QE Data\2020Aug2_Jessa'sFlowers_SetB\2020Jul30_Jessa_2\2020Jul30_033.raw

[S192] 2020Jul30_034 [Sample number=38, Population=F2, Female P=P47-1, Male P=Disco/2, Color group=White with patterns, Floral organ=ST, Secondary color=brown, General main color=white, Main color=white, General secondary color=brown, ColorCategory=WhiteBrown, Red=No, Brown=Yes, Yellow=No, Purple=No, White=Yes, Pink=No, ColorCategories_Updated=WhiteBrown, FlowerCategories_W_ST=WhiteBrown, MainPetalColor=White, MinorPetalColor_Absent=Brown, MinorPetalColor=Brown, PetalColorGroup1=WhiteBrown, PetalColorGroup2=WhiteBrown]

E:\QE Data\2020Aug2_Jessa'sFlowers_SetB\2020Jul30_Jessa_2\2020Jul30_034.raw

[S193] 2020Jul30_035 [Sample number=39, Population=F2, Female P=P47-1, Male P=Disco/2, Color group=White with patterns, Floral organ=W, Secondary color=brown, General main color=white, Main color=white, General secondary color=brown, ColorCategory=WhiteBrown, Red=No, Brown=Yes, Yellow=No, Purple=No, White=Yes, Pink=No, ColorCategories_Updated=WhiteBrown, FlowerCategories_W_ST=WhiteBrown, MainPetalColor=White, MinorPetalColor_Absent=Brown, MinorPetalColor=Brown, PetalColorGroup1=WhiteBrown, PetalColorGroup2=WhiteBrown]

E:\QE Data\2020Aug2_Jessa'sFlowers_SetB\2020Jul30_Jessa_2\2020Jul30_035.raw

[S194] 2020Jul30_036 [Sample number=40, Population=F2, Female P=P47-1, Male P=Disco/2, Color group=White with patterns, Floral organ=ST, Secondary color=brown, General main color=white, Main color=white, General secondary color=brown, ColorCategory=WhiteBrown, Red=No, Brown=Yes, Yellow=No, Purple=No, White=Yes, Pink=No, ColorCategories_Updated=WhiteBrown, FlowerCategories_W_ST=WhiteBrown, MainPetalColor=White, MinorPetalColor_Absent=Brown, MinorPetalColor=Brown, PetalColorGroup1=WhiteBrown, PetalColorGroup2=WhiteBrown]

E:\QE Data\2020Aug2_Jessa'sFlowers_SetB\2020Jul30_Jessa_2\2020Jul30_036.raw

[S195] 2020Jul30_037 [Sample number=47, Population=F2, Female P=Snowdrop, Male P=Gelber, Color group=White with patterns, Floral organ=W, Secondary color=brown, General main color=white, Main color=white, General secondary color=brown, ColorCategory=WhiteBrown, Red=No, Brown=Yes, Yellow=No, Purple=No, White=Yes, Pink=No, ColorCategories_Updated=WhiteBrown, FlowerCategories_W_ST=WhiteBrown, MainPetalColor=White, MinorPetalColor_Absent=Brown, MinorPetalColor=Brown, PetalColorGroup1=WhiteBrown, PetalColorGroup2=WhiteBrown]

E:\QE Data\2020Aug2_Jessa'sFlowers_SetB\2020Jul30_Jessa_2\2020Jul30_037.raw

[S196] 2020Jul30_038 [Sample number=48, Population=F2, Female P=Snowdrop, Male P=Gelber, Color group=White with patterns, Floral organ=ST, Secondary color=brown, General main color=white, Main color=white, General secondary color=brown, ColorCategory=WhiteBrown, Red=No, Brown=Yes, Yellow=No, Purple=No, White=Yes, Pink=No, ColorCategories_Updated=WhiteBrown, FlowerCategories_W_ST=WhiteBrown, MainPetalColor=White, MinorPetalColor_Absent=Brown, MinorPetalColor=Brown, PetalColorGroup1=WhiteBrown, PetalColorGroup2=WhiteBrown]

E:\QE Data\2020Aug2_Jessa'sFlowers_SetB\2020Jul30_Jessa_2\2020Jul30_038.raw

[S197] 2020Jul30_039 [Sample number=n/a, Population=n/a, Female P=n/a, Male P=n/a, Color group=n/a, Floral organ=n/a, Secondary color=n/a, General main color=n/a, Main color=n/a, General secondary color=n/a, ColorCategory=n/a, Red=n/a, Brown=n/a, Yellow=n/a, Purple=n/a, White=n/a, Pink=n/a, ColorCategories_Updated=n/a, FlowerCategories_W_ST=n/a, MainPetalColor=n/a, MinorPetalColor_Absent=n/a, MinorPetalColor=n/a, PetalColorGroup1=n/a, PetalColorGroup2=n/a]

E:\QE Data\2020Aug2_Jessa'sFlowers_SetB\2020Jul30_Jessa_2\2020Jul30_039.raw

[S198] 2020Jul30_040 [Sample number=n/a, Population=n/a, Female P=n/a, Male P=n/a, Color group=n/a, Floral organ=n/a, Secondary color=n/a, General main color=n/a, Main color=n/a, General secondary color=n/a, ColorCategory=n/a, Red=n/a, Brown=n/a, Yellow=n/a, Purple=n/a, White=n/a, Pink=n/a, ColorCategories_Updated=n/a, FlowerCategories_W_ST=n/a, MainPetalColor=n/a, MinorPetalColor_Absent=n/a, MinorPetalColor=n/a, PetalColorGroup1=n/a, PetalColorGroup2=n/a]

E:\QE Data\2020Aug2_Jessa'sFlowers_SetB\2020Jul30_Jessa_2\2020Jul30_040.raw

[S199] 2020Jul30_041 [Sample number=55, Population=F2, Female P=Disco/2, Male P=Gelber, Color group=White with patterns, Floral organ=W, Secondary color=brown, General main color=white, Main color=white, General secondary color=brown, ColorCategory=WhiteBrown, Red=No, Brown=Yes, Yellow=No, Purple=No, White=Yes, Pink=No, ColorCategories_Updated=WhiteBrown, FlowerCategories_W_ST=WhiteBrown, MainPetalColor=White, MinorPetalColor_Absent=Brown, MinorPetalColor=Brown, PetalColorGroup1=WhiteBrown, PetalColorGroup2=WhiteBrown]

E:\QE Data\2020Aug2_Jessa'sFlowers_SetB\2020Jul30_Jessa_2\2020Jul30_041.raw

[S200] 2020Jul30_042 [Sample number=56, Population=F2, Female P=Disco/2, Male P=Gelber, Color group=White with patterns, Floral organ=ST, Secondary color=brown, General main color=white, Main color=white, General secondary color=brown, ColorCategory=WhiteBrown, Red=No, Brown=Yes, Yellow=No, Purple=No, White=Yes, Pink=No, ColorCategories_Updated=WhiteBrown, FlowerCategories_W_ST=WhiteBrown, MainPetalColor=White, MinorPetalColor_Absent=Brown, MinorPetalColor=Brown, PetalColorGroup1=WhiteBrown, PetalColorGroup2=WhiteBrown]

E:\QE Data\2020Aug2_Jessa'sFlowers_SetB\2020Jul30_Jessa_2\2020Jul30_042.raw

[S201] 2020Jul30_043 [Sample number=57, Population=F2, Female P=Aurora, Male P=Gelber, Color group=White with patterns, Floral organ=W, Secondary color=brown, General main color=white, Main color=white, General secondary color=brown, ColorCategory=WhiteBrown, Red=No, Brown=Yes, Yellow=No, Purple=No, White=Yes, Pink=No, ColorCategories_Updated=WhiteBrown, FlowerCategories_W_ST=WhiteBrown, MainPetalColor=White, MinorPetalColor_Absent=Brown, MinorPetalColor=Brown, PetalColorGroup1=WhiteBrown, PetalColorGroup2=WhiteBrown]

E:\QE Data\2020Aug2_Jessa'sFlowers_SetB\2020Jul30_Jessa_2\2020Jul30_043.raw

[S202] 2020Jul30_044 [Sample number=58, Population=F2, Female P=Aurora, Male P=Gelber, Color group=White with patterns, Floral organ=ST, Secondary color=brown, General main color=white, Main color=white, General secondary color=brown, ColorCategory=WhiteBrown, Red=No, Brown=Yes, Yellow=No, Purple=No, White=Yes, Pink=No, ColorCategories_Updated=WhiteBrown, FlowerCategories_W_ST=WhiteBrown, MainPetalColor=White, MinorPetalColor_Absent=Brown, MinorPetalColor=Brown, PetalColorGroup1=WhiteBrown, PetalColorGroup2=WhiteBrown]

E:\QE Data\2020Aug2_Jessa'sFlowers_SetB\2020Jul30_Jessa_2\2020Jul30_044.raw

[S203] 2020Jul30_045 [Sample number=65, Population=F2, Female P=Aurora, Male P=P47-1, Color group=White with patterns, Floral organ=W, Secondary color=brown, General main color=white, Main color=white, General secondary color=brown, ColorCategory=WhiteBrown, Red=No, Brown=Yes, Yellow=No, Purple=No, White=Yes, Pink=No, ColorCategories_Updated=WhiteBrown, FlowerCategories_W_ST=WhiteBrown, MainPetalColor=White, MinorPetalColor_Absent=Brown, MinorPetalColor=Brown, PetalColorGroup1=WhiteBrown, PetalColorGroup2=WhiteBrown]

E:\QE Data\2020Aug2_Jessa'sFlowers_SetB\2020Jul30_Jessa_2\2020Jul30_045.raw

[S204] 2020Jul30_046 [Sample number=66, Population=F2, Female P=Aurora, Male P=P47-1, Color group=White with patterns, Floral organ=ST, Secondary color=brown, General main color=white, Main color=white, General secondary color=brown, ColorCategory=WhiteBrown, Red=No, Brown=Yes, Yellow=No, Purple=No, White=Yes, Pink=No, ColorCategories_Updated=WhiteBrown, FlowerCategories_W_ST=WhiteBrown, MainPetalColor=White, MinorPetalColor_Absent=Brown, MinorPetalColor=Brown, PetalColorGroup1=WhiteBrown, PetalColorGroup2=WhiteBrown]

E:\QE Data\2020Aug2_Jessa'sFlowers_SetB\2020Jul30_Jessa_2\2020Jul30_046.raw

[S208] 2020Jul30_050 [Sample number=n/a, Population=n/a, Female P=n/a, Male P=n/a, Color group=n/a, Floral organ=n/a, Secondary color=n/a, General main color=n/a, Main color=n/a, General secondary color=n/a, ColorCategory=n/a, Red=n/a, Brown=n/a, Yellow=n/a, Purple=n/a, White=n/a, Pink=n/a, ColorCategories_Updated=n/a, FlowerCategories_W_ST=n/a, MainPetalColor=n/a, MinorPetalColor_Absent=n/a, MinorPetalColor=n/a, PetalColorGroup1=n/a, PetalColorGroup2=n/a]

E:\QE Data\2020Aug2_Jessa'sFlowers_SetB\2020Jul30_Jessa_2\2020Jul30_050.raw

[S209] 2020Jul30_051 [Sample number=n/a, Population=n/a, Female P=n/a, Male P=n/a, Color group=n/a, Floral organ=n/a, Secondary color=n/a, General main color=n/a, Main color=n/a, General secondary color=n/a, ColorCategory=n/a, Red=n/a, Brown=n/a, Yellow=n/a, Purple=n/a, White=n/a, Pink=n/a, ColorCategories_Updated=n/a, FlowerCategories_W_ST=n/a, MainPetalColor=n/a, MinorPetalColor_Absent=n/a, MinorPetalColor=n/a, PetalColorGroup1=n/a, PetalColorGroup2=n/a]

E:\QE Data\2020Aug2_Jessa'sFlowers_SetB\2020Jul30_Jessa_2\2020Jul30_051.raw

[S210] 2020Jul30_052 [Sample number=1, Population=F2, Female P=Gelber, Male P=P47-1, Color group=Brown, Floral organ=W, Secondary color=brown, General main color=brown, Main color=brown, General secondary color=brown, ColorCategory=Brown, Red=No, Brown=Yes, Yellow=No, Purple=No, White=No, Pink=No, ColorCategories_Updated=Brown, FlowerCategories_W_ST=Brown, MainPetalColor=Brown, MinorPetalColor_Absent=Absent, MinorPetalColor=Brown, PetalColorGroup1=Brown, PetalColorGroup2=Brown]

E:\QE Data\2020Aug2_Jessa'sFlowers_SetB\2020Jul30_Jessa_2\2020Jul30_052.raw

[S211] 2020Jul30_053 [Sample number=2, Population=F2, Female P=Gelber, Male P=P47-1, Color group=Brown, Floral organ=ST, Secondary color=brown, General main color=brown, Main color=brown, General secondary color=brown, ColorCategory=Brown, Red=No, Brown=Yes, Yellow=No, Purple=No, White=No, Pink=No, ColorCategories_Updated=Brown, FlowerCategories_W_ST=Brown, MainPetalColor=Brown, MinorPetalColor_Absent=Absent, MinorPetalColor=Brown, PetalColorGroup1=Brown, PetalColorGroup2=Brown]

E:\QE Data\2020Aug2_Jessa'sFlowers_SetB\2020Jul30_Jessa_2\2020Jul30_053.raw

[S212] 2020Jul30_054 [Sample number=3, Population=F2, Female P=Gelber, Male P=P47-1, Color group=Brown, Floral organ=W, Secondary color=brown, General main color=brown, Main color=brown, General secondary color=brown, ColorCategory=Brown, Red=No, Brown=Yes, Yellow=No, Purple=No, White=No, Pink=No, ColorCategories_Updated=Brown, FlowerCategories_W_ST=Brown, MainPetalColor=Brown, MinorPetalColor_Absent=Absent, MinorPetalColor=Brown, PetalColorGroup1=Brown, PetalColorGroup2=Brown]

E:\QE Data\2020Aug2_Jessa'sFlowers_SetB\2020Jul30_Jessa_2\2020Jul30_054.raw

[S213] 2020Jul30_055 [Sample number=4, Population=F2, Female P=Gelber, Male P=P47-1, Color group=Brown, Floral organ=ST, Secondary color=brown, General main color=brown, Main color=brown, General secondary color=brown, ColorCategory=Brown, Red=No, Brown=Yes, Yellow=No, Purple=No, White=No, Pink=No, ColorCategories_Updated=Brown, FlowerCategories_W_ST=Brown, MainPetalColor=Brown, MinorPetalColor_Absent=Absent, MinorPetalColor=Brown, PetalColorGroup1=Brown, PetalColorGroup2=Brown]

E:\QE Data\2020Aug2_Jessa'sFlowers_SetB\2020Jul30_Jessa_2\2020Jul30_055.raw

[S214] 2020Jul30_056 [Sample number=6, Population=F2, Female P=Gelber, Male P=P47-1, Color group=Brown, Floral organ=W, Secondary color=brown, General main color=brown, Main color=brown, General secondary color=brown, ColorCategory=Brown, Red=No, Brown=Yes, Yellow=No, Purple=No, White=No, Pink=No, ColorCategories_Updated=Brown, FlowerCategories_W_ST=BrownRed, MainPetalColor=Brown, MinorPetalColor_Absent=Absent, MinorPetalColor=Brown, PetalColorGroup1=Brown, PetalColorGroup2=Brown]

E:\QE Data\2020Aug2_Jessa'sFlowers_SetB\2020Jul30_Jessa_2\2020Jul30_056.raw

[S217] 2020Jul30_059 [Sample number=36, Population=F2, Female P=P47-1, Male P=Disco/2, Color group=Brown, Floral organ=ST, Secondary color=brown, General main color=brown, Main color=brown, General secondary color=brown, ColorCategory=Brown, Red=No, Brown=Yes, Yellow=No, Purple=No, White=No, Pink=No, ColorCategories_Updated=Brown, FlowerCategories_W_ST=Brown, MainPetalColor=Brown, MinorPetalColor_Absent=Absent, MinorPetalColor=Brown, PetalColorGroup1=Brown, PetalColorGroup2=Brown]

E:\QE Data\2020Aug2_Jessa'sFlowers_SetB\2020Jul30_Jessa_2\2020Jul30_059.raw

[S218] 2020Jul30_060 [Sample number=n/a, Population=n/a, Female P=n/a, Male P=n/a, Color group=n/a, Floral organ=n/a, Secondary color=n/a, General main color=n/a, Main color=n/a, General secondary color=n/a, ColorCategory=n/a, Red=n/a, Brown=n/a, Yellow=n/a, Purple=n/a, White=n/a, Pink=n/a, ColorCategories_Updated=n/a, FlowerCategories_W_ST=n/a, MainPetalColor=n/a, MinorPetalColor_Absent=n/a, MinorPetalColor=n/a, PetalColorGroup1=n/a, PetalColorGroup2=n/a]

E:\QE Data\2020Aug2_Jessa'sFlowers_SetB\2020Jul30_Jessa_2\2020Jul30_060.raw

[S219] 2020Jul30_061 [Sample number=n/a, Population=n/a, Female P=n/a, Male P=n/a, Color group=n/a, Floral organ=n/a, Secondary color=n/a, General main color=n/a, Main color=n/a, General secondary color=n/a, ColorCategory=n/a, Red=n/a, Brown=n/a, Yellow=n/a, Purple=n/a, White=n/a, Pink=n/a, ColorCategories_Updated=n/a, FlowerCategories_W_ST=n/a, MainPetalColor=n/a, MinorPetalColor_Absent=n/a, MinorPetalColor=n/a, PetalColorGroup1=n/a, PetalColorGroup2=n/a]

E:\QE Data\2020Aug2_Jessa'sFlowers_SetB\2020Jul30_Jessa_2\2020Jul30_061.raw

[S220] 2020Jul30_062 [Sample number=5, Population=F2, Female P=Gelber, Male P=P47-1, Color group=Brown mixed, Floral organ=ST, Secondary color=red, General main color=brown, Main color=brown, General secondary color=red, ColorCategory=RedBrown, Red=Yes, Brown=Yes, Yellow=No, Purple=No, White=No, Pink=No, ColorCategories_Updated=BrownRed, FlowerCategories_W_ST=BrownRed, MainPetalColor=Brown, MinorPetalColor_Absent=Red, MinorPetalColor=Red, PetalColorGroup1=BrownRed, PetalColorGroup2=BrownRed-RedBrown]

E:\QE Data\2020Aug2_Jessa'sFlowers_SetB\2020Jul30_Jessa_2\2020Jul30_062.raw

[S221] 2020Jul30_063 [Sample number=7, Population=F2, Female P=Gelber, Male P=P47-1, Color group=Brown mixed, Floral organ=W, Secondary color=pink, General main color=brown, Main color=brown, General secondary color=red, ColorCategory=RedBrown, Red=No, Brown=Yes, Yellow=No, Purple=No, White=No, Pink=Yes, ColorCategories_Updated=BrownPink, FlowerCategories_W_ST=BrownPink, MainPetalColor=Brown, MinorPetalColor_Absent=Pink, MinorPetalColor=Pink, PetalColorGroup1=BrownPink, PetalColorGroup2=BrownPink-PinkBrown]

E:\QE Data\2020Aug2_Jessa'sFlowers_SetB\2020Jul30_Jessa_2\2020Jul30_063.raw

[S222] 2020Jul30_064 [Sample number=9, Population=F2, Female P=Gelber, Male P=P47-1, Color group=Brown mixed, Floral organ=W, Secondary color=pink, General main color=brown, Main color=brown, General secondary color=red, ColorCategory=RedBrown, Red=No, Brown=Yes, Yellow=No, Purple=No, White=No, Pink=Yes, ColorCategories_Updated=BrownPink, FlowerCategories_W_ST=BrownPink, MainPetalColor=Brown, MinorPetalColor_Absent=Pink, MinorPetalColor=Pink, PetalColorGroup1=BrownPink, PetalColorGroup2=BrownPink-PinkBrown]

E:\QE Data\2020Aug2_Jessa'sFlowers_SetB\2020Jul30_Jessa_2\2020Jul30_064.raw

[S223] 2020Jul30_065 [Sample number=11, Population=F2, Female P=Snowdrop, Male P=P47-1, Color group=Brown mixed, Floral organ=W, Secondary color=pink, General main color=brown, Main color=brown, General secondary color=red, ColorCategory=RedBrown, Red=No, Brown=Yes, Yellow=No, Purple=No, White=No, Pink=Yes, ColorCategories_Updated=BrownPink, FlowerCategories_W_ST=BrownPink, MainPetalColor=Brown, MinorPetalColor_Absent=Pink, MinorPetalColor=Pink, PetalColorGroup1=BrownPink, PetalColorGroup2=BrownPink-PinkBrown]

E:\QE Data\2020Aug2_Jessa'sFlowers_SetB\2020Jul30_Jessa_2\2020Jul30_065.raw

[S224] 2020Jul30_066 [Sample number=14, Population=F2, Female P=Snowdrop, Male P=P47-1, Color group=Red, Floral organ=W, Secondary color=red, General main color=brown, Main color=brown, General secondary color=red, ColorCategory=RedBrown, Red=Yes, Brown=Yes, Yellow=No, Purple=No, White=No, Pink=No, ColorCategories_Updated=BrownRed, FlowerCategories_W_ST=BrownRed, MainPetalColor=Brown, MinorPetalColor_Absent=Red, MinorPetalColor=Red, PetalColorGroup1=BrownRed, PetalColorGroup2=BrownRed-RedBrown]

E:\QE Data\2020Aug2_Jessa'sFlowers_SetB\2020Jul30_Jessa_2\2020Jul30_066.raw

[S225] 2020Jul30_067 [Sample number=33, Population=F2, Female P=P47-1, Male P=Disco/2, Color group=Brown mixed, Floral organ=W, Secondary color=red, General main color=brown, Main color=brown, General secondary color=red, ColorCategory=RedBrown, Red=Yes, Brown=Yes, Yellow=No, Purple=No, White=No, Pink=No, ColorCategories_Updated=BrownRed, FlowerCategories_W_ST=BrownRed, MainPetalColor=Brown, MinorPetalColor_Absent=Red, MinorPetalColor=Red, PetalColorGroup1=BrownRed, PetalColorGroup2=BrownRed-RedBrown]

E:\QE Data\2020Aug2_Jessa'sFlowers_SetB\2020Jul30_Jessa_2\2020Jul30_067.raw

[S226] 2020Jul30_068 [Sample number=35, Population=F2, Female P=P47-1, Male P=Disco/2, Color group=Brown mixed, Floral organ=W, Secondary color=brown, General main color=brown, Main color=brown, General secondary color=red, ColorCategory=BrownPurple, Red=No, Brown=Yes, Yellow=No, Purple=Yes, White=No, Pink=No, ColorCategories_Updated=Brown, FlowerCategories_W_ST=Brown, MainPetalColor=Brown, MinorPetalColor_Absent=Absent, MinorPetalColor=Brown, PetalColorGroup1=Brown, PetalColorGroup2=Brown]

E:\QE Data\2020Aug2_Jessa'sFlowers_SetB\2020Jul30_Jessa_2\2020Jul30_068.raw

[S227] 2020Jul30_069 [Sample number=63, Population=F2, Female P=Aurora, Male P=P47-1, Color group=Brown mixed, Floral organ=W, Secondary color=pink, General main color=brown, Main color=brown, General secondary color=red, ColorCategory=RedBrown, Red=No, Brown=Yes, Yellow=No, Purple=No, White=No, Pink=Yes, ColorCategories_Updated=BrownPink, FlowerCategories_W_ST=BrownPink, MainPetalColor=Brown, MinorPetalColor_Absent=Pink, MinorPetalColor=Pink, PetalColorGroup1=BrownPink, PetalColorGroup2=BrownPink-PinkBrown]

E:\QE Data\2020Aug2_Jessa'sFlowers_SetB\2020Jul30_Jessa_2\2020Jul30_069.raw

[S228] 2020Jul30_070 [Sample number=n/a, Population=n/a, Female P=n/a, Male P=n/a, Color group=n/a, Floral organ=n/a, Secondary color=n/a, General main color=n/a, Main color=n/a, General secondary color=n/a, ColorCategory=n/a, Red=n/a, Brown=n/a, Yellow=n/a, Purple=n/a, White=n/a, Pink=n/a, ColorCategories_Updated=n/a, FlowerCategories_W_ST=n/a, MainPetalColor=n/a, MinorPetalColor_Absent=n/a, MinorPetalColor=n/a, PetalColorGroup1=n/a, PetalColorGroup2=n/a]

E:\QE Data\2020Aug2_Jessa'sFlowers_SetB\2020Jul30_Jessa_2\2020Jul30_070.raw

[S229] 2020Jul30_071 [Sample number=n/a, Population=n/a, Female P=n/a, Male P=n/a, Color group=n/a, Floral organ=n/a, Secondary color=n/a, General main color=n/a, Main color=n/a, General secondary color=n/a, ColorCategory=n/a, Red=n/a, Brown=n/a, Yellow=n/a, Purple=n/a, White=n/a, Pink=n/a, ColorCategories_Updated=n/a, FlowerCategories_W_ST=n/a, MainPetalColor=n/a, MinorPetalColor_Absent=n/a, MinorPetalColor=n/a, PetalColorGroup1=n/a, PetalColorGroup2=n/a]

E:\QE Data\2020Aug2_Jessa'sFlowers_SetB\2020Jul30_Jessa_2\2020Jul30_071.raw

[S230] 2020Jul30_072 [Sample number=8, Population=F2, Female P=Gelber, Male P=P47-1, Color group=Brown mixed, Floral organ=ST, Secondary color=brown, General main color=red, Main color=pink, General secondary color=brown, ColorCategory=RedBrown, Red=No, Brown=Yes, Yellow=No, Purple=No, White=No, Pink=Yes, ColorCategories_Updated=BrownPink, FlowerCategories_W_ST=BrownPink, MainPetalColor=Pink, MinorPetalColor_Absent=Brown, MinorPetalColor=Brown, PetalColorGroup1=PinkBrown, PetalColorGroup2=BrownPink-PinkBrown]

E:\QE Data\2020Aug2_Jessa'sFlowers_SetB\2020Jul30_Jessa_2\2020Jul30_072.raw

[S231] 2020Jul30_073 [Sample number=10, Population=F2, Female P=Gelber, Male P=P47-1, Color group=Brown mixed, Floral organ=ST, Secondary color=brown, General main color=red, Main color=pink, General secondary color=brown, ColorCategory=RedBrown, Red=No, Brown=Yes, Yellow=No, Purple=No, White=No, Pink=Yes, ColorCategories_Updated=BrownPink, FlowerCategories_W_ST=BrownPink, MainPetalColor=Pink, MinorPetalColor_Absent=Brown, MinorPetalColor=Brown, PetalColorGroup1=PinkBrown, PetalColorGroup2=BrownPink-PinkBrown]

E:\QE Data\2020Aug2_Jessa'sFlowers_SetB\2020Jul30_Jessa_2\2020Jul30_073.raw

[S232] 2020Jul30_074 [Sample number=12, Population=F2, Female P=Snowdrop, Male P=P47-1, Color group=Brown mixed, Floral organ=ST, Secondary color=brown, General main color=red, Main color=pink, General secondary color=brown, ColorCategory=RedBrown, Red=No, Brown=Yes, Yellow=No, Purple=No, White=No, Pink=Yes, ColorCategories_Updated=BrownPink, FlowerCategories_W_ST=BrownPink, MainPetalColor=Pink, MinorPetalColor_Absent=Brown, MinorPetalColor=Brown, PetalColorGroup1=PinkBrown, PetalColorGroup2=BrownPink-PinkBrown]

E:\QE Data\2020Aug2_Jessa'sFlowers_SetB\2020Jul30_Jessa_2\2020Jul30_074.raw

[S233] 2020Jul30_075 [Sample number=17, Population=F2, Female P=Snowdrop, Male P=P47-1, Color group=Red, Floral organ=W, Secondary color=brown, General main color=red, Main color=red, General secondary color=brown, ColorCategory=RedBrown, Red=Yes, Brown=Yes, Yellow=No, Purple=No, White=No, Pink=No, ColorCategories_Updated=BrownRed, FlowerCategories_W_ST=BrownRed, MainPetalColor=Red, MinorPetalColor_Absent=Brown, MinorPetalColor=Brown, PetalColorGroup1=RedBrown, PetalColorGroup2=BrownRed-RedBrown]

E:\QE Data\2020Aug2_Jessa'sFlowers_SetB\2020Jul30_Jessa_2\2020Jul30_075.raw

[S234] 2020Jul30_076 [Sample number=18, Population=F2, Female P=Snowdrop, Male P=P47-1, Color group=Red, Floral organ=ST, Secondary color=brown, General main color=red, Main color=red, General secondary color=brown, ColorCategory=RedBrown, Red=Yes, Brown=Yes, Yellow=No, Purple=No, White=No, Pink=No, ColorCategories_Updated=BrownRed, FlowerCategories_W_ST=BrownRed, MainPetalColor=Red, MinorPetalColor_Absent=Brown, MinorPetalColor=Brown, PetalColorGroup1=RedBrown, PetalColorGroup2=BrownRed-RedBrown]

E:\QE Data\2020Aug2_Jessa'sFlowers_SetB\2020Jul30_Jessa_2\2020Jul30_076.raw

[S236] 2020Jul30_078 [Sample number=21, Population=F2, Female P=Snowdrop, Male P=P47-1, Color group=Red, Floral organ=W, Secondary color=brown, General main color=red, Main color=red, General secondary color=brown, ColorCategory=RedBrown, Red=Yes, Brown=Yes, Yellow=No, Purple=No, White=No, Pink=No, ColorCategories_Updated=BrownRed, FlowerCategories_W_ST=BrownRed, MainPetalColor=Red, MinorPetalColor_Absent=Brown, MinorPetalColor=Brown, PetalColorGroup1=RedBrown, PetalColorGroup2=BrownRed-RedBrown]

E:\QE Data\2020Aug2_Jessa'sFlowers_SetB\2020Jul30_Jessa_2\2020Jul30_078.raw

[S237] 2020Jul30_079 [Sample number=31, Population=F2, Female P=P47-1, Male P=Disco/2, Color group=Brown mixed, Floral organ=W, Secondary color=brown, General main color=red, Main color=pink, General secondary color=brown, ColorCategory=RedBrown, Red=No, Brown=Yes, Yellow=No, Purple=No, White=No, Pink=Yes, ColorCategories_Updated=BrownPink, FlowerCategories_W_ST=BrownPink, MainPetalColor=Pink, MinorPetalColor_Absent=Brown, MinorPetalColor=Brown, PetalColorGroup1=PinkBrown, PetalColorGroup2=BrownPink-PinkBrown]

E:\QE Data\2020Aug2_Jessa'sFlowers_SetB\2020Jul30_Jessa_2\2020Jul30_079.raw

[S238] 2020Jul30_080 [Sample number=32, Population=F2, Female P=P47-1, Male P=Disco/2, Color group=Brown mixed, Floral organ=ST, Secondary color=brown, General main color=red, Main color=pink, General secondary color=brown, ColorCategory=RedBrown, Red=No, Brown=Yes, Yellow=No, Purple=No, White=No, Pink=Yes, ColorCategories_Updated=BrownPink, FlowerCategories_W_ST=BrownPink, MainPetalColor=Pink, MinorPetalColor_Absent=Brown, MinorPetalColor=Brown, PetalColorGroup1=PinkBrown, PetalColorGroup2=BrownPink-PinkBrown]

E:\QE Data\2020Aug2_Jessa'sFlowers_SetB\2020Jul30_Jessa_2\2020Jul30_080.raw

[S239] 2020Jul30_081 [Sample number=n/a, Population=n/a, Female P=n/a, Male P=n/a, Color group=n/a, Floral organ=n/a, Secondary color=n/a, General main color=n/a, Main color=n/a, General secondary color=n/a, ColorCategory=n/a, Red=n/a, Brown=n/a, Yellow=n/a, Purple=n/a, White=n/a, Pink=n/a, ColorCategories_Updated=n/a, FlowerCategories_W_ST=n/a, MainPetalColor=n/a, MinorPetalColor_Absent=n/a, MinorPetalColor=n/a, PetalColorGroup1=n/a, PetalColorGroup2=n/a]

E:\QE Data\2020Aug2_Jessa'sFlowers_SetB\2020Jul30_Jessa_2\2020Jul30_081.raw

[S240] 2020Jul30_082 [Sample number=n/a, Population=n/a, Female P=n/a, Male P=n/a, Color group=n/a, Floral organ=n/a, Secondary color=n/a, General main color=n/a, Main color=n/a, General secondary color=n/a, ColorCategory=n/a, Red=n/a, Brown=n/a, Yellow=n/a, Purple=n/a, White=n/a, Pink=n/a, ColorCategories_Updated=n/a, FlowerCategories_W_ST=n/a, MainPetalColor=n/a, MinorPetalColor_Absent=n/a, MinorPetalColor=n/a, PetalColorGroup1=n/a, PetalColorGroup2=n/a]

E:\QE Data\2020Aug2_Jessa'sFlowers_SetB\2020Jul30_Jessa_2\2020Jul30_082.raw

[S241] 2020Jul30_083 [Sample number=34, Population=F2, Female P=P47-1, Male P=Disco/2, Color group=Red, Floral organ=ST, Secondary color=brown, General main color=red, Main color=red, General secondary color=brown, ColorCategory=RedBrown, Red=Yes, Brown=Yes, Yellow=No, Purple=No, White=No, Pink=No, ColorCategories_Updated=BrownRed, FlowerCategories_W_ST=BrownRed, MainPetalColor=Red, MinorPetalColor_Absent=Brown, MinorPetalColor=Brown, PetalColorGroup1=RedBrown, PetalColorGroup2=BrownRed-RedBrown]

E:\QE Data\2020Aug2_Jessa'sFlowers_SetB\2020Jul30_Jessa_2\2020Jul30_083.raw

[S242] 2020Jul30_084 [Sample number=61, Population=F2, Female P=Aurora, Male P=P47-1, Color group=Red, Floral organ=W, Secondary color=brown, General main color=red, Main color=red, General secondary color=brown, ColorCategory=RedBrown, Red=Yes, Brown=Yes, Yellow=No, Purple=No, White=No, Pink=No, ColorCategories_Updated=BrownRed, FlowerCategories_W_ST=BrownRed, MainPetalColor=Red, MinorPetalColor_Absent=Brown, MinorPetalColor=Brown, PetalColorGroup1=RedBrown, PetalColorGroup2=BrownRed-RedBrown]

E:\QE Data\2020Aug2_Jessa'sFlowers_SetB\2020Jul30_Jessa_2\2020Jul30_084.raw

[S243] 2020Jul30_085 [Sample number=n/a, Population=n/a, Female P=n/a, Male P=n/a, Color group=n/a, Floral organ=n/a, Secondary color=n/a, General main color=n/a, Main color=n/a, General secondary color=n/a, ColorCategory=n/a, Red=n/a, Brown=n/a, Yellow=n/a, Purple=n/a, White=n/a, Pink=n/a, ColorCategories_Updated=n/a, FlowerCategories_W_ST=n/a, MainPetalColor=n/a, MinorPetalColor_Absent=n/a, MinorPetalColor=n/a, PetalColorGroup1=n/a, PetalColorGroup2=n/a]

E:\QE Data\2020Aug2_Jessa'sFlowers_SetB\2020Jul30_Jessa_2\2020Jul30_085.raw

[S244] 2020Jul30_086 [Sample number=13, Population=F2, Female P=Snowdrop, Male P=P47-1, Color group=Red, Floral organ=ST, Secondary color=red, General main color=red, Main color=red, General secondary color=red, ColorCategory=Red, Red=Yes, Brown=No, Yellow=No, Purple=No, White=No, Pink=No, ColorCategories_Updated=Red, FlowerCategories_W_ST=BrownRed, MainPetalColor=Red, MinorPetalColor_Absent=Absent, MinorPetalColor=Red, PetalColorGroup1=Red, PetalColorGroup2=Red]

E:\QE Data\2020Aug2_Jessa'sFlowers_SetB\2020Jul30_Jessa_2\2020Jul30_086.raw

[S246] 2020Jul30_088 [Sample number=22, Population=F2, Female P=Snowdrop, Male P=P47-1, Color group=Red, Floral organ=ST, Secondary color=red, General main color=red, Main color=red, General secondary color=red, ColorCategory=Red, Red=Yes, Brown=No, Yellow=No, Purple=No, White=No, Pink=No, ColorCategories_Updated=Red, FlowerCategories_W_ST=BrownRed, MainPetalColor=Red, MinorPetalColor_Absent=Absent, MinorPetalColor=Red, PetalColorGroup1=Red, PetalColorGroup2=Red]

E:\QE Data\2020Aug2_Jessa'sFlowers_SetB\2020Jul30_Jessa_2\2020Jul30_088.raw

[S247] 2020Jul30_089 [Sample number=62, Population=F2, Female P=Aurora, Male P=P47-1, Color group=Red, Floral organ=ST, Secondary color=red, General main color=red, Main color=red, General secondary color=red, ColorCategory=Red, Red=Yes, Brown=No, Yellow=No, Purple=No, White=No, Pink=No, ColorCategories_Updated=Red, FlowerCategories_W_ST=BrownRed, MainPetalColor=Red, MinorPetalColor_Absent=Absent, MinorPetalColor=Red, PetalColorGroup1=Red, PetalColorGroup2=Red]

E:\QE Data\2020Aug2_Jessa'sFlowers_SetB\2020Jul30_Jessa_2\2020Jul30_089.raw

[S248] 2020Jul30_090 [Sample number=64, Population=F2, Female P=Aurora, Male P=P47-1, Color group=Brown mixed, Floral organ=ST, Secondary color=pink, General main color=red, Main color=pink, General secondary color=red, ColorCategory=Red, Red=No, Brown=No, Yellow=No, Purple=No, White=No, Pink=Yes, ColorCategories_Updated=BrownPink, FlowerCategories_W_ST=BrownPink, MainPetalColor=Pink, MinorPetalColor_Absent=Absent, MinorPetalColor=Pink, PetalColorGroup1=Pink, PetalColorGroup2=BrownPink-PinkBrown]

E:\QE Data\2020Aug2_Jessa'sFlowers_SetB\2020Jul30_Jessa_2\2020Jul30_090.raw

[S249] 2020Jul30_091 [Sample number=n/a, Population=n/a, Female P=n/a, Male P=n/a, Color group=n/a, Floral organ=n/a, Secondary color=n/a, General main color=n/a, Main color=n/a, General secondary color=n/a, ColorCategory=n/a, Red=n/a, Brown=n/a, Yellow=n/a, Purple=n/a, White=n/a, Pink=n/a, ColorCategories_Updated=n/a, FlowerCategories_W_ST=n/a, MainPetalColor=n/a, MinorPetalColor_Absent=n/a, MinorPetalColor=n/a, PetalColorGroup1=n/a, PetalColorGroup2=n/a]

E:\QE Data\2020Aug2_Jessa'sFlowers_SetB\2020Jul30_Jessa_2\2020Jul30_091.raw

[S250] 2020Jul30_092 [Sample number=n/a, Population=n/a, Female P=n/a, Male P=n/a, Color group=n/a, Floral organ=n/a, Secondary color=n/a, General main color=n/a, Main color=n/a, General secondary color=n/a, ColorCategory=n/a, Red=n/a, Brown=n/a, Yellow=n/a, Purple=n/a, White=n/a, Pink=n/a, ColorCategories_Updated=n/a, FlowerCategories_W_ST=n/a, MainPetalColor=n/a, MinorPetalColor_Absent=n/a, MinorPetalColor=n/a, PetalColorGroup1=n/a, PetalColorGroup2=n/a]

E:\QE Data\2020Aug2_Jessa'sFlowers_SetB\2020Jul30_Jessa_2\2020Jul30_092.raw

[S251] 2020Jul30_093 [Sample number=71, Population=2263, Female P=Rinrei, Male P=P47-1, Color group=Red, Floral organ=ST, Secondary color=purple, General main color=red, Main color=red, General secondary color=purple, ColorCategory=RedPurple, Red=Yes, Brown=No, Yellow=No, Purple=Yes, White=No, Pink=No, ColorCategories_Updated=RedPurple, FlowerCategories_W_ST=RedPurple, MainPetalColor=Purplish red, MinorPetalColor_Absent=Absent, MinorPetalColor=Purplish red, PetalColorGroup1=Purplish red, PetalColorGroup2=Purplish red]

E:\QE Data\2020Aug2_Jessa'sFlowers_SetB\2020Jul30_Jessa_2\2020Jul30_093.raw

[S252] 2020Jul30_094 [Sample number=72, Population=2263, Female P=Rinrei, Male P=P47-1, Color group=Red, Floral organ=W, Secondary color=purple, General main color=red, Main color=red, General secondary color=purple, ColorCategory=RedPurple, Red=Yes, Brown=No, Yellow=No, Purple=Yes, White=No, Pink=No, ColorCategories_Updated=RedPurple, FlowerCategories_W_ST=RedPurple, MainPetalColor=Purplish red, MinorPetalColor_Absent=Absent, MinorPetalColor=Purplish red, PetalColorGroup1=Purplish red, PetalColorGroup2=Purplish red]

E:\QE Data\2020Aug2_Jessa'sFlowers_SetB\2020Jul30_Jessa_2\2020Jul30_094.raw

[S253] 2020Jul30_095 [Sample number=75, Population=71B, Female P=P47-1, Male P=NV153, Color group=Red, Floral organ=W, Secondary color=purple, General main color=red, Main color=red, General secondary color=purple, ColorCategory=RedPurple, Red=Yes, Brown=No, Yellow=No, Purple=Yes, White=No, Pink=No, ColorCategories_Updated=RedPurple, FlowerCategories_W_ST=RedPurple, MainPetalColor=Purplish red, MinorPetalColor_Absent=Absent, MinorPetalColor=Purplish red, PetalColorGroup1=Purplish red, PetalColorGroup2=Purplish red]

E:\QE Data\2020Aug2_Jessa'sFlowers_SetB\2020Jul30_Jessa_2\2020Jul30_095.raw

[S254] 2020Jul30_096 [Sample number=76, Population=71B, Female P=P47-1, Male P=NV153, Color group=Red, Floral organ=ST, Secondary color=purple, General main color=red, Main color=red, General secondary color=purple, ColorCategory=RedPurple, Red=Yes, Brown=No, Yellow=No, Purple=Yes, White=No, Pink=No, ColorCategories_Updated=RedPurple, FlowerCategories_W_ST=RedPurple, MainPetalColor=Purplish red, MinorPetalColor_Absent=Absent, MinorPetalColor=Purplish red, PetalColorGroup1=Purplish red, PetalColorGroup2=Purplish red]

E:\QE Data\2020Aug2_Jessa'sFlowers_SetB\2020Jul30_Jessa_2\2020Jul30_096.raw

[S255] 2020Jul30_097 [Sample number=n/a, Population=n/a, Female P=n/a, Male P=n/a, Color group=n/a, Floral organ=n/a, Secondary color=n/a, General main color=n/a, Main color=n/a, General secondary color=n/a, ColorCategory=n/a, Red=n/a, Brown=n/a, Yellow=n/a, Purple=n/a, White=n/a, Pink=n/a, ColorCategories_Updated=n/a, FlowerCategories_W_ST=n/a, MainPetalColor=n/a, MinorPetalColor_Absent=n/a, MinorPetalColor=n/a, PetalColorGroup1=n/a, PetalColorGroup2=n/a]

E:\QE Data\2020Aug2_Jessa'sFlowers_SetB\2020Jul30_Jessa_2\2020Jul30_097.raw

[S256] 2020Jul30_098 [Sample number=n/a, Population=n/a, Female P=n/a, Male P=n/a, Color group=n/a, Floral organ=n/a, Secondary color=n/a, General main color=n/a, Main color=n/a, General secondary color=n/a, ColorCategory=n/a, Red=n/a, Brown=n/a, Yellow=n/a, Purple=n/a, White=n/a, Pink=n/a, ColorCategories_Updated=n/a, FlowerCategories_W_ST=n/a, MainPetalColor=n/a, MinorPetalColor_Absent=n/a, MinorPetalColor=n/a, PetalColorGroup1=n/a, PetalColorGroup2=n/a]

E:\QE Data\2020Aug2_Jessa'sFlowers_SetB\2020Jul30_Jessa_2\2020Jul30_098.raw

[S257] 2020Jul30_099 [Sample number=n/a, Population=n/a, Female P=n/a, Male P=n/a, Color group=n/a, Floral organ=n/a, Secondary color=n/a, General main color=n/a, Main color=n/a, General secondary color=n/a, ColorCategory=n/a, Red=n/a, Brown=n/a, Yellow=n/a, Purple=n/a, White=n/a, Pink=n/a, ColorCategories_Updated=n/a, FlowerCategories_W_ST=n/a, MainPetalColor=n/a, MinorPetalColor_Absent=n/a, MinorPetalColor=n/a, PetalColorGroup1=n/a, PetalColorGroup2=n/a]

E:\QE Data\2020Aug2_Jessa'sFlowers_SetB\2020Jul30_Jessa_2\2020Jul30_099.raw

[S258] 2020Jul30_100 [Sample number=n/a, Population=n/a, Female P=n/a, Male P=n/a, Color group=n/a, Floral organ=n/a, Secondary color=n/a, General main color=n/a, Main color=n/a, General secondary color=n/a, ColorCategory=n/a, Red=n/a, Brown=n/a, Yellow=n/a, Purple=n/a, White=n/a, Pink=n/a, ColorCategories_Updated=n/a, FlowerCategories_W_ST=n/a, MainPetalColor=n/a, MinorPetalColor_Absent=n/a, MinorPetalColor=n/a, PetalColorGroup1=n/a, PetalColorGroup2=n/a]

E:\QE Data\2020Aug2_Jessa'sFlowers_SetB\2020Jul30_Jessa_2\2020Jul30_100.raw

[S261] 2020Jul30_103 [Sample number=n/a, Population=n/a, Female P=n/a, Male P=n/a, Color group=n/a, Floral organ=n/a, Secondary color=n/a, General main color=n/a, Main color=n/a, General secondary color=n/a, ColorCategory=n/a, Red=n/a, Brown=n/a, Yellow=n/a, Purple=n/a, White=n/a, Pink=n/a, ColorCategories_Updated=n/a, FlowerCategories_W_ST=n/a, MainPetalColor=n/a, MinorPetalColor_Absent=n/a, MinorPetalColor=n/a, PetalColorGroup1=n/a, PetalColorGroup2=n/a]

E:\QE Data\2020Aug2_Jessa'sFlowers_SetB\2020Jul30_Jessa_2\2020Jul30_103.raw

[S264] 2020Jul30_106 [Sample number=n/a, Population=n/a, Female P=n/a, Male P=n/a, Color group=n/a, Floral organ=n/a, Secondary color=n/a, General main color=n/a, Main color=n/a, General secondary color=n/a, ColorCategory=n/a, Red=n/a, Brown=n/a, Yellow=n/a, Purple=n/a, White=n/a, Pink=n/a, ColorCategories_Updated=n/a, FlowerCategories_W_ST=n/a, MainPetalColor=n/a, MinorPetalColor_Absent=n/a, MinorPetalColor=n/a, PetalColorGroup1=n/a, PetalColorGroup2=n/a]

E:\QE Data\2020Aug2_Jessa'sFlowers_SetB\2020Jul30_Jessa_2\2020Jul30_106.raw

[S267] 2020Jul30_109 [Sample number=n/a, Population=n/a, Female P=n/a, Male P=n/a, Color group=n/a, Floral organ=n/a, Secondary color=n/a, General main color=n/a, Main color=n/a, General secondary color=n/a, ColorCategory=n/a, Red=n/a, Brown=n/a, Yellow=n/a, Purple=n/a, White=n/a, Pink=n/a, ColorCategories_Updated=n/a, FlowerCategories_W_ST=n/a, MainPetalColor=n/a, MinorPetalColor_Absent=n/a, MinorPetalColor=n/a, PetalColorGroup1=n/a, PetalColorGroup2=n/a]

E:\QE Data\2020Aug2_Jessa'sFlowers_SetB\2020Jul30_Jessa_2\2020Jul30_109.raw

[S271] 2020Jul30_113 [Sample number=n/a, Population=n/a, Female P=n/a, Male P=n/a, Color group=n/a, Floral organ=n/a, Secondary color=n/a, General main color=n/a, Main color=n/a, General secondary color=n/a, ColorCategory=n/a, Red=n/a, Brown=n/a, Yellow=n/a, Purple=n/a, White=n/a, Pink=n/a, ColorCategories_Updated=n/a, FlowerCategories_W_ST=n/a, MainPetalColor=n/a, MinorPetalColor_Absent=n/a, MinorPetalColor=n/a, PetalColorGroup1=n/a, PetalColorGroup2=n/a]

E:\QE Data\2020Aug2_Jessa'sFlowers_SetB\2020Jul30_Jessa_2\2020Jul30_113.raw

[S274] 2020Jul30_116 [Sample number=n/a, Population=n/a, Female P=n/a, Male P=n/a, Color group=n/a, Floral organ=n/a, Secondary color=n/a, General main color=n/a, Main color=n/a, General secondary color=n/a, ColorCategory=n/a, Red=n/a, Brown=n/a, Yellow=n/a, Purple=n/a, White=n/a, Pink=n/a, ColorCategories_Updated=n/a, FlowerCategories_W_ST=n/a, MainPetalColor=n/a, MinorPetalColor_Absent=n/a, MinorPetalColor=n/a, PetalColorGroup1=n/a, PetalColorGroup2=n/a]

E:\QE Data\2020Aug2_Jessa'sFlowers_SetB\2020Jul30_Jessa_2\2020Jul30_116.raw

[S277] 2020Jul30_119 [Sample number=n/a, Population=n/a, Female P=n/a, Male P=n/a, Color group=n/a, Floral organ=n/a, Secondary color=n/a, General main color=n/a, Main color=n/a, General secondary color=n/a, ColorCategory=n/a, Red=n/a, Brown=n/a, Yellow=n/a, Purple=n/a, White=n/a, Pink=n/a, ColorCategories_Updated=n/a, FlowerCategories_W_ST=n/a, MainPetalColor=n/a, MinorPetalColor_Absent=n/a, MinorPetalColor=n/a, PetalColorGroup1=n/a, PetalColorGroup2=n/a]

E:\QE Data\2020Aug2_Jessa'sFlowers_SetB\2020Jul30_Jessa_2\2020Jul30_119.raw

[S280] 2020Jul30_122 [Sample number=n/a, Population=n/a, Female P=n/a, Male P=n/a, Color group=n/a, Floral organ=n/a, Secondary color=n/a, General main color=n/a, Main color=n/a, General secondary color=n/a, ColorCategory=n/a, Red=n/a, Brown=n/a, Yellow=n/a, Purple=n/a, White=n/a, Pink=n/a, ColorCategories_Updated=n/a, FlowerCategories_W_ST=n/a, MainPetalColor=n/a, MinorPetalColor_Absent=n/a, MinorPetalColor=n/a, PetalColorGroup1=n/a, PetalColorGroup2=n/a]

E:\QE Data\2020Aug2_Jessa'sFlowers_SetB\2020Jul30_Jessa_2\2020Jul30_122.raw

[S284] 2020Aug5_001 [Sample number=n/a, Population=n/a, Female P=n/a, Male P=n/a, Color group=n/a, Floral organ=n/a, Secondary color=n/a, General main color=n/a, Main color=n/a, General secondary color=n/a, ColorCategory=n/a, Red=n/a, Brown=n/a, Yellow=n/a, Purple=n/a, White=n/a, Pink=n/a, ColorCategories_Updated=n/a, FlowerCategories_W_ST=n/a, MainPetalColor=n/a, MinorPetalColor_Absent=n/a, MinorPetalColor=n/a, PetalColorGroup1=n/a, PetalColorGroup2=n/a]

E:\QE Data\2020Aug5_Jessa'sFlowers_SetC\2020Aug5_Jessa_3_Neg\2020Aug5_001.raw

[S285] 2020Aug5_002 [Sample number=n/a, Population=n/a, Female P=n/a, Male P=n/a, Color group=n/a, Floral organ=n/a, Secondary color=n/a, General main color=n/a, Main color=n/a, General secondary color=n/a, ColorCategory=n/a, Red=n/a, Brown=n/a, Yellow=n/a, Purple=n/a, White=n/a, Pink=n/a, ColorCategories_Updated=n/a, FlowerCategories_W_ST=n/a, MainPetalColor=n/a, MinorPetalColor_Absent=n/a, MinorPetalColor=n/a, PetalColorGroup1=n/a, PetalColorGroup2=n/a]

E:\QE Data\2020Aug5_Jessa'sFlowers_SetC\2020Aug5_Jessa_3_Neg\2020Aug5_002.raw

[S286] 2020Aug5_003 [Sample number=n/a, Population=n/a, Female P=n/a, Male P=n/a, Color group=n/a, Floral organ=n/a, Secondary color=n/a, General main color=n/a, Main color=n/a, General secondary color=n/a, ColorCategory=n/a, Red=n/a, Brown=n/a, Yellow=n/a, Purple=n/a, White=n/a, Pink=n/a, ColorCategories_Updated=n/a, FlowerCategories_W_ST=n/a, MainPetalColor=n/a, MinorPetalColor_Absent=n/a, MinorPetalColor=n/a, PetalColorGroup1=n/a, PetalColorGroup2=n/a]

E:\QE Data\2020Aug5_Jessa'sFlowers_SetC\2020Aug5_Jessa_3_Neg\2020Aug5_003.raw

[S288] 2020Aug5_005 [Sample number=n/a, Population=n/a, Female P=n/a, Male P=n/a, Color group=n/a, Floral organ=n/a, Secondary color=n/a, General main color=n/a, Main color=n/a, General secondary color=n/a, ColorCategory=n/a, Red=n/a, Brown=n/a, Yellow=n/a, Purple=n/a, White=n/a, Pink=n/a, ColorCategories_Updated=n/a, FlowerCategories_W_ST=n/a, MainPetalColor=n/a, MinorPetalColor_Absent=n/a, MinorPetalColor=n/a, PetalColorGroup1=n/a, PetalColorGroup2=n/a]

E:\QE Data\2020Aug5_Jessa'sFlowers_SetC\2020Aug5_Jessa_3_Neg\2020Aug5_005.raw

[S289] 2020Aug5_006 [Sample number=n/a, Population=n/a, Female P=n/a, Male P=n/a, Color group=n/a, Floral organ=n/a, Secondary color=n/a, General main color=n/a, Main color=n/a, General secondary color=n/a, ColorCategory=n/a, Red=n/a, Brown=n/a, Yellow=n/a, Purple=n/a, White=n/a, Pink=n/a, ColorCategories_Updated=n/a, FlowerCategories_W_ST=n/a, MainPetalColor=n/a, MinorPetalColor_Absent=n/a, MinorPetalColor=n/a, PetalColorGroup1=n/a, PetalColorGroup2=n/a]

E:\QE Data\2020Aug5_Jessa'sFlowers_SetC\2020Aug5_Jessa_3_Neg\2020Aug5_006.raw

[S290] 2020Aug5_007 [Sample number=n/a, Population=n/a, Female P=n/a, Male P=n/a, Color group=n/a, Floral organ=n/a, Secondary color=n/a, General main color=n/a, Main color=n/a, General secondary color=n/a, ColorCategory=n/a, Red=n/a, Brown=n/a, Yellow=n/a, Purple=n/a, White=n/a, Pink=n/a, ColorCategories_Updated=n/a, FlowerCategories_W_ST=n/a, MainPetalColor=n/a, MinorPetalColor_Absent=n/a, MinorPetalColor=n/a, PetalColorGroup1=n/a, PetalColorGroup2=n/a]

E:\QE Data\2020Aug5_Jessa'sFlowers_SetC\2020Aug5_Jessa_3_Neg\2020Aug5_007.raw

[S291] 2020Aug5_008 [Sample number=26, Population=F2, Female P=Gelber, Male P=P47-1, Color group=White, Floral organ=ST, Secondary color=pink, General main color=white, Main color=white, General secondary color=white, ColorCategory=White, Red=No, Brown=No, Yellow=No, Purple=No, White=Yes, Pink=Yes, ColorCategories_Updated=White, FlowerCategories_W_ST=WhiteYellow, MainPetalColor=White, MinorPetalColor_Absent=Pink, MinorPetalColor=Pink, PetalColorGroup1=WhitePink, PetalColorGroup2=WhitePink]

E:\QE Data\2020Aug5_Jessa'sFlowers_SetC\2020Aug5_Jessa_3_Neg\2020Aug5_008.raw

[S292] 2020Aug5_009 [Sample number=27, Population=F2, Female P=P47-1, Male P=Snowdrop, Color group=White, Floral organ=W, Secondary color=white, General main color=white, Main color=white, General secondary color=white, ColorCategory=White, Red=No, Brown=No, Yellow=No, Purple=No, White=Yes, Pink=No, ColorCategories_Updated=White, FlowerCategories_W_ST=White, MainPetalColor=White, MinorPetalColor_Absent=Absent, MinorPetalColor=White, PetalColorGroup1=White, PetalColorGroup2=White]

E:\QE Data\2020Aug5_Jessa'sFlowers_SetC\2020Aug5_Jessa_3_Neg\2020Aug5_009.raw

[S293] 2020Aug5_010 [Sample number=28, Population=F2, Female P=P47-1, Male P=Snowdrop, Color group=White, Floral organ=ST, Secondary color=white, General main color=white, Main color=white, General secondary color=white, ColorCategory=White, Red=No, Brown=No, Yellow=No, Purple=No, White=Yes, Pink=No, ColorCategories_Updated=White, FlowerCategories_W_ST=White, MainPetalColor=White, MinorPetalColor_Absent=Absent, MinorPetalColor=White, PetalColorGroup1=White, PetalColorGroup2=White]

E:\QE Data\2020Aug5_Jessa'sFlowers_SetC\2020Aug5_Jessa_3_Neg\2020Aug5_010.raw

[S294] 2020Aug5_011 [Sample number=30, Population=F2, Female P=P47-1, Male P=Snowdrop, Color group=White, Floral organ=ST, Secondary color=pink, General main color=white, Main color=white, General secondary color=white, ColorCategory=White, Red=No, Brown=No, Yellow=No, Purple=No, White=Yes, Pink=Yes, ColorCategories_Updated=White, FlowerCategories_W_ST=WhiteBrown, MainPetalColor=White, MinorPetalColor_Absent=Pink, MinorPetalColor=Pink, PetalColorGroup1=WhitePink, PetalColorGroup2=WhitePink]

E:\QE Data\2020Aug5_Jessa'sFlowers_SetC\2020Aug5_Jessa_3_Neg\2020Aug5_011.raw

[S297] 2020Aug5_014 [Sample number=45, Population=F2, Female P=Snowdrop, Male P=Gelber, Color group=White, Floral organ=W, Secondary color=white, General main color=white, Main color=white, General secondary color=white, ColorCategory=White, Red=No, Brown=No, Yellow=No, Purple=No, White=Yes, Pink=No, ColorCategories_Updated=White, FlowerCategories_W_ST=White, MainPetalColor=White, MinorPetalColor_Absent=Absent, MinorPetalColor=White, PetalColorGroup1=White, PetalColorGroup2=White]

E:\QE Data\2020Aug5_Jessa'sFlowers_SetC\2020Aug5_Jessa_3_Neg\2020Aug5_014.raw

[S298] 2020Aug5_015 [Sample number=46, Population=F2, Female P=Snowdrop, Male P=Gelber, Color group=White, Floral organ=ST, Secondary color=white, General main color=white, Main color=white, General secondary color=white, ColorCategory=White, Red=No, Brown=No, Yellow=No, Purple=No, White=Yes, Pink=No, ColorCategories_Updated=White, FlowerCategories_W_ST=White, MainPetalColor=White, MinorPetalColor_Absent=Absent, MinorPetalColor=White, PetalColorGroup1=White, PetalColorGroup2=White]

E:\QE Data\2020Aug5_Jessa'sFlowers_SetC\2020Aug5_Jessa_3_Neg\2020Aug5_015.raw

[S299] 2020Aug5_016 [Sample number=n/a, Population=n/a, Female P=n/a, Male P=n/a, Color group=n/a, Floral organ=n/a, Secondary color=n/a, General main color=n/a, Main color=n/a, General secondary color=n/a, ColorCategory=n/a, Red=n/a, Brown=n/a, Yellow=n/a, Purple=n/a, White=n/a, Pink=n/a, ColorCategories_Updated=n/a, FlowerCategories_W_ST=n/a, MainPetalColor=n/a, MinorPetalColor_Absent=n/a, MinorPetalColor=n/a, PetalColorGroup1=n/a, PetalColorGroup2=n/a]

E:\QE Data\2020Aug5_Jessa'sFlowers_SetC\2020Aug5_Jessa_3_Neg\2020Aug5_016.raw

[S300] 2020Aug5_017 [Sample number=n/a, Population=n/a, Female P=n/a, Male P=n/a, Color group=n/a, Floral organ=n/a, Secondary color=n/a, General main color=n/a, Main color=n/a, General secondary color=n/a, ColorCategory=n/a, Red=n/a, Brown=n/a, Yellow=n/a, Purple=n/a, White=n/a, Pink=n/a, ColorCategories_Updated=n/a, FlowerCategories_W_ST=n/a, MainPetalColor=n/a, MinorPetalColor_Absent=n/a, MinorPetalColor=n/a, PetalColorGroup1=n/a, PetalColorGroup2=n/a]

E:\QE Data\2020Aug5_Jessa'sFlowers_SetC\2020Aug5_Jessa_3_Neg\2020Aug5_017.raw

[S301] 2020Aug5_018 [Sample number=52, Population=F2, Female P=Disco/2, Male P=Gelber, Color group=White, Floral organ=ST, Secondary color=pink, General main color=white, Main color=white, General secondary color=white, ColorCategory=White, Red=No, Brown=No, Yellow=No, Purple=No, White=Yes, Pink=Yes, ColorCategories_Updated=White, FlowerCategories_W_ST=WhiteYellow, MainPetalColor=White, MinorPetalColor_Absent=Pink, MinorPetalColor=Pink, PetalColorGroup1=WhitePink, PetalColorGroup2=WhitePink]

E:\QE Data\2020Aug5_Jessa'sFlowers_SetC\2020Aug5_Jessa_3_Neg\2020Aug5_018.raw

[S302] 2020Aug5_019 [Sample number=53, Population=F2, Female P=Disco/2, Male P=Gelber, Color group=White, Floral organ=W, Secondary color=white, General main color=white, Main color=white, General secondary color=white, ColorCategory=White, Red=No, Brown=No, Yellow=No, Purple=No, White=Yes, Pink=No, ColorCategories_Updated=White, FlowerCategories_W_ST=White, MainPetalColor=White, MinorPetalColor_Absent=Absent, MinorPetalColor=White, PetalColorGroup1=White, PetalColorGroup2=White]

E:\QE Data\2020Aug5_Jessa'sFlowers_SetC\2020Aug5_Jessa_3_Neg\2020Aug5_019.raw

[S303] 2020Aug5_020 [Sample number=54, Population=F2, Female P=Disco/2, Male P=Gelber, Color group=White, Floral organ=ST, Secondary color=white, General main color=white, Main color=white, General secondary color=white, ColorCategory=White, Red=No, Brown=No, Yellow=No, Purple=No, White=Yes, Pink=No, ColorCategories_Updated=White, FlowerCategories_W_ST=White, MainPetalColor=White, MinorPetalColor_Absent=Absent, MinorPetalColor=White, PetalColorGroup1=White, PetalColorGroup2=White]

E:\QE Data\2020Aug5_Jessa'sFlowers_SetC\2020Aug5_Jessa_3_Neg\2020Aug5_020.raw

[S306] 2020Aug5_023 [Sample number=25, Population=F2, Female P=Gelber, Male P=P47-1, Color group=White with patterns, Floral organ=W, Secondary color=yellow, General main color=white, Main color=white, General secondary color=brown, ColorCategory=WhiteYellow, Red=No, Brown=No, Yellow=Yes, Purple=No, White=Yes, Pink=No, ColorCategories_Updated=WhiteYellow, FlowerCategories_W_ST=WhiteYellow, MainPetalColor=White, MinorPetalColor_Absent=Yellow, MinorPetalColor=Yellow, PetalColorGroup1=WhiteYellow, PetalColorGroup2=WhiteYellow]

E:\QE Data\2020Aug5_Jessa'sFlowers_SetC\2020Aug5_Jessa_3_Neg\2020Aug5_023.raw

[S308] 2020Aug5_025 [Sample number=51, Population=F2, Female P=Disco/2, Male P=Gelber, Color group=White with patterns, Floral organ=W, Secondary color=yellow, General main color=white, Main color=white, General secondary color=brown, ColorCategory=WhiteYellow, Red=No, Brown=No, Yellow=Yes, Purple=No, White=Yes, Pink=No, ColorCategories_Updated=WhiteYellow, FlowerCategories_W_ST=WhiteYellow, MainPetalColor=White, MinorPetalColor_Absent=Yellow, MinorPetalColor=Yellow, PetalColorGroup1=WhiteYellow, PetalColorGroup2=WhiteYellow]

E:\QE Data\2020Aug5_Jessa'sFlowers_SetC\2020Aug5_Jessa_3_Neg\2020Aug5_025.raw

[S309] 2020Aug5_026 [Sample number=59, Population=F2, Female P=Aurora, Male P=Gelber, Color group=White with patterns, Floral organ=W, Secondary color=yellow, General main color=white, Main color=white, General secondary color=brown, ColorCategory=WhiteYellow, Red=No, Brown=No, Yellow=Yes, Purple=No, White=Yes, Pink=No, ColorCategories_Updated=WhiteYellow, FlowerCategories_W_ST=WhiteYellow, MainPetalColor=White, MinorPetalColor_Absent=Yellow, MinorPetalColor=Yellow, PetalColorGroup1=WhiteYellow, PetalColorGroup2=WhiteYellow]

E:\QE Data\2020Aug5_Jessa'sFlowers_SetC\2020Aug5_Jessa_3_Neg\2020Aug5_026.raw

[S310] 2020Aug5_027 [Sample number=60, Population=F2, Female P=Aurora, Male P=Gelber, Color group=White with patterns, Floral organ=ST, Secondary color=yellow, General main color=white, Main color=white, General secondary color=brown, ColorCategory=WhiteYellow, Red=No, Brown=No, Yellow=Yes, Purple=No, White=Yes, Pink=No, ColorCategories_Updated=WhiteYellow, FlowerCategories_W_ST=WhiteYellow, MainPetalColor=White, MinorPetalColor_Absent=Yellow, MinorPetalColor=Yellow, PetalColorGroup1=WhiteYellow, PetalColorGroup2=WhiteYellow]

E:\QE Data\2020Aug5_Jessa'sFlowers_SetC\2020Aug5_Jessa_3_Neg\2020Aug5_027.raw

[S311] 2020Aug5_028 [Sample number=n/a, Population=n/a, Female P=n/a, Male P=n/a, Color group=n/a, Floral organ=n/a, Secondary color=n/a, General main color=n/a, Main color=n/a, General secondary color=n/a, ColorCategory=n/a, Red=n/a, Brown=n/a, Yellow=n/a, Purple=n/a, White=n/a, Pink=n/a, ColorCategories_Updated=n/a, FlowerCategories_W_ST=n/a, MainPetalColor=n/a, MinorPetalColor_Absent=n/a, MinorPetalColor=n/a, PetalColorGroup1=n/a, PetalColorGroup2=n/a]

E:\QE Data\2020Aug5_Jessa'sFlowers_SetC\2020Aug5_Jessa_3_Neg\2020Aug5_028.raw

[S312] 2020Aug5_029 [Sample number=n/a, Population=n/a, Female P=n/a, Male P=n/a, Color group=n/a, Floral organ=n/a, Secondary color=n/a, General main color=n/a, Main color=n/a, General secondary color=n/a, ColorCategory=n/a, Red=n/a, Brown=n/a, Yellow=n/a, Purple=n/a, White=n/a, Pink=n/a, ColorCategories_Updated=n/a, FlowerCategories_W_ST=n/a, MainPetalColor=n/a, MinorPetalColor_Absent=n/a, MinorPetalColor=n/a, PetalColorGroup1=n/a, PetalColorGroup2=n/a]

E:\QE Data\2020Aug5_Jessa'sFlowers_SetC\2020Aug5_Jessa_3_Neg\2020Aug5_029.raw

[S313] 2020Aug5_030 [Sample number=23, Population=F2, Female P=Gelber, Male P=P47-1, Color group=White with patterns, Floral organ=W, Secondary color=brown, General main color=white, Main color=white, General secondary color=brown, ColorCategory=WhiteBrown, Red=No, Brown=Yes, Yellow=No, Purple=No, White=Yes, Pink=No, ColorCategories_Updated=WhiteBrown, FlowerCategories_W_ST=WhiteBrown, MainPetalColor=White, MinorPetalColor_Absent=Brown, MinorPetalColor=Brown, PetalColorGroup1=WhiteBrown, PetalColorGroup2=WhiteBrown]

E:\QE Data\2020Aug5_Jessa'sFlowers_SetC\2020Aug5_Jessa_3_Neg\2020Aug5_030.raw

[S314] 2020Aug5_031 [Sample number=24, Population=F2, Female P=Gelber, Male P=P47-1, Color group=White with patterns, Floral organ=ST, Secondary color=brown, General main color=white, Main color=white, General secondary color=brown, ColorCategory=WhiteBrown, Red=No, Brown=Yes, Yellow=No, Purple=No, White=Yes, Pink=No, ColorCategories_Updated=WhiteBrown, FlowerCategories_W_ST=WhiteBrown, MainPetalColor=White, MinorPetalColor_Absent=Brown, MinorPetalColor=Brown, PetalColorGroup1=WhiteBrown, PetalColorGroup2=WhiteBrown]

E:\QE Data\2020Aug5_Jessa'sFlowers_SetC\2020Aug5_Jessa_3_Neg\2020Aug5_031.raw

[S315] 2020Aug5_032 [Sample number=29, Population=F2, Female P=P47-1, Male P=Snowdrop, Color group=White with patterns, Floral organ=W, Secondary color=brown, General main color=white, Main color=white, General secondary color=brown, ColorCategory=WhiteBrown, Red=No, Brown=Yes, Yellow=No, Purple=No, White=Yes, Pink=No, ColorCategories_Updated=WhiteBrown, FlowerCategories_W_ST=WhiteBrown, MainPetalColor=White, MinorPetalColor_Absent=Brown, MinorPetalColor=Brown, PetalColorGroup1=WhiteBrown, PetalColorGroup2=WhiteBrown]

E:\QE Data\2020Aug5_Jessa'sFlowers_SetC\2020Aug5_Jessa_3_Neg\2020Aug5_032.raw

[S316] 2020Aug5_033 [Sample number=37, Population=F2, Female P=P47-1, Male P=Disco/2, Color group=White with patterns, Floral organ=W, Secondary color=brown, General main color=white, Main color=white, General secondary color=brown, ColorCategory=WhiteBrown, Red=No, Brown=Yes, Yellow=No, Purple=No, White=Yes, Pink=No, ColorCategories_Updated=WhiteBrown, FlowerCategories_W_ST=WhiteBrown, MainPetalColor=White, MinorPetalColor_Absent=Brown, MinorPetalColor=Brown, PetalColorGroup1=WhiteBrown, PetalColorGroup2=WhiteBrown]

E:\QE Data\2020Aug5_Jessa'sFlowers_SetC\2020Aug5_Jessa_3_Neg\2020Aug5_033.raw

[S317] 2020Aug5_034 [Sample number=38, Population=F2, Female P=P47-1, Male P=Disco/2, Color group=White with patterns, Floral organ=ST, Secondary color=brown, General main color=white, Main color=white, General secondary color=brown, ColorCategory=WhiteBrown, Red=No, Brown=Yes, Yellow=No, Purple=No, White=Yes, Pink=No, ColorCategories_Updated=WhiteBrown, FlowerCategories_W_ST=WhiteBrown, MainPetalColor=White, MinorPetalColor_Absent=Brown, MinorPetalColor=Brown, PetalColorGroup1=WhiteBrown, PetalColorGroup2=WhiteBrown]

E:\QE Data\2020Aug5_Jessa'sFlowers_SetC\2020Aug5_Jessa_3_Neg\2020Aug5_034.raw

[S318] 2020Aug5_035 [Sample number=39, Population=F2, Female P=P47-1, Male P=Disco/2, Color group=White with patterns, Floral organ=W, Secondary color=brown, General main color=white, Main color=white, General secondary color=brown, ColorCategory=WhiteBrown, Red=No, Brown=Yes, Yellow=No, Purple=No, White=Yes, Pink=No, ColorCategories_Updated=WhiteBrown, FlowerCategories_W_ST=WhiteBrown, MainPetalColor=White, MinorPetalColor_Absent=Brown, MinorPetalColor=Brown, PetalColorGroup1=WhiteBrown, PetalColorGroup2=WhiteBrown]

E:\QE Data\2020Aug5_Jessa'sFlowers_SetC\2020Aug5_Jessa_3_Neg\2020Aug5_035.raw

[S319] 2020Aug5_036 [Sample number=40, Population=F2, Female P=P47-1, Male P=Disco/2, Color group=White with patterns, Floral organ=ST, Secondary color=brown, General main color=white, Main color=white, General secondary color=brown, ColorCategory=WhiteBrown, Red=No, Brown=Yes, Yellow=No, Purple=No, White=Yes, Pink=No, ColorCategories_Updated=WhiteBrown, FlowerCategories_W_ST=WhiteBrown, MainPetalColor=White, MinorPetalColor_Absent=Brown, MinorPetalColor=Brown, PetalColorGroup1=WhiteBrown, PetalColorGroup2=WhiteBrown]

E:\QE Data\2020Aug5_Jessa'sFlowers_SetC\2020Aug5_Jessa_3_Neg\2020Aug5_036.raw

[S320] 2020Aug5_037 [Sample number=47, Population=F2, Female P=Snowdrop, Male P=Gelber, Color group=White with patterns, Floral organ=W, Secondary color=brown, General main color=white, Main color=white, General secondary color=brown, ColorCategory=WhiteBrown, Red=No, Brown=Yes, Yellow=No, Purple=No, White=Yes, Pink=No, ColorCategories_Updated=WhiteBrown, FlowerCategories_W_ST=WhiteBrown, MainPetalColor=White, MinorPetalColor_Absent=Brown, MinorPetalColor=Brown, PetalColorGroup1=WhiteBrown, PetalColorGroup2=WhiteBrown]

E:\QE Data\2020Aug5_Jessa'sFlowers_SetC\2020Aug5_Jessa_3_Neg\2020Aug5_037.raw

[S321] 2020Aug5_038 [Sample number=48, Population=F2, Female P=Snowdrop, Male P=Gelber, Color group=White with patterns, Floral organ=ST, Secondary color=brown, General main color=white, Main color=white, General secondary color=brown, ColorCategory=WhiteBrown, Red=No, Brown=Yes, Yellow=No, Purple=No, White=Yes, Pink=No, ColorCategories_Updated=WhiteBrown, FlowerCategories_W_ST=WhiteBrown, MainPetalColor=White, MinorPetalColor_Absent=Brown, MinorPetalColor=Brown, PetalColorGroup1=WhiteBrown, PetalColorGroup2=WhiteBrown]

E:\QE Data\2020Aug5_Jessa'sFlowers_SetC\2020Aug5_Jessa_3_Neg\2020Aug5_038.raw

[S322] 2020Aug5_039 [Sample number=n/a, Population=n/a, Female P=n/a, Male P=n/a, Color group=n/a, Floral organ=n/a, Secondary color=n/a, General main color=n/a, Main color=n/a, General secondary color=n/a, ColorCategory=n/a, Red=n/a, Brown=n/a, Yellow=n/a, Purple=n/a, White=n/a, Pink=n/a, ColorCategories_Updated=n/a, FlowerCategories_W_ST=n/a, MainPetalColor=n/a, MinorPetalColor_Absent=n/a, MinorPetalColor=n/a, PetalColorGroup1=n/a, PetalColorGroup2=n/a]

E:\QE Data\2020Aug5_Jessa'sFlowers_SetC\2020Aug5_Jessa_3_Neg\2020Aug5_039.raw

[S323] 2020Aug5_040 [Sample number=n/a, Population=n/a, Female P=n/a, Male P=n/a, Color group=n/a, Floral organ=n/a, Secondary color=n/a, General main color=n/a, Main color=n/a, General secondary color=n/a, ColorCategory=n/a, Red=n/a, Brown=n/a, Yellow=n/a, Purple=n/a, White=n/a, Pink=n/a, ColorCategories_Updated=n/a, FlowerCategories_W_ST=n/a, MainPetalColor=n/a, MinorPetalColor_Absent=n/a, MinorPetalColor=n/a, PetalColorGroup1=n/a, PetalColorGroup2=n/a]

E:\QE Data\2020Aug5_Jessa'sFlowers_SetC\2020Aug5_Jessa_3_Neg\2020Aug5_040.raw

[S324] 2020Aug5_041 [Sample number=55, Population=F2, Female P=Disco/2, Male P=Gelber, Color group=White with patterns, Floral organ=W, Secondary color=brown, General main color=white, Main color=white, General secondary color=brown, ColorCategory=WhiteBrown, Red=No, Brown=Yes, Yellow=No, Purple=No, White=Yes, Pink=No, ColorCategories_Updated=WhiteBrown, FlowerCategories_W_ST=WhiteBrown, MainPetalColor=White, MinorPetalColor_Absent=Brown, MinorPetalColor=Brown, PetalColorGroup1=WhiteBrown, PetalColorGroup2=WhiteBrown]

E:\QE Data\2020Aug5_Jessa'sFlowers_SetC\2020Aug5_Jessa_3_Neg\2020Aug5_041.raw

[S325] 2020Aug5_042 [Sample number=56, Population=F2, Female P=Disco/2, Male P=Gelber, Color group=White with patterns, Floral organ=ST, Secondary color=brown, General main color=white, Main color=white, General secondary color=brown, ColorCategory=WhiteBrown, Red=No, Brown=Yes, Yellow=No, Purple=No, White=Yes, Pink=No, ColorCategories_Updated=WhiteBrown, FlowerCategories_W_ST=WhiteBrown, MainPetalColor=White, MinorPetalColor_Absent=Brown, MinorPetalColor=Brown, PetalColorGroup1=WhiteBrown, PetalColorGroup2=WhiteBrown]

E:\QE Data\2020Aug5_Jessa'sFlowers_SetC\2020Aug5_Jessa_3_Neg\2020Aug5_042.raw

[S326] 2020Aug5_043 [Sample number=57, Population=F2, Female P=Aurora, Male P=Gelber, Color group=White with patterns, Floral organ=W, Secondary color=brown, General main color=white, Main color=white, General secondary color=brown, ColorCategory=WhiteBrown, Red=No, Brown=Yes, Yellow=No, Purple=No, White=Yes, Pink=No, ColorCategories_Updated=WhiteBrown, FlowerCategories_W_ST=WhiteBrown, MainPetalColor=White, MinorPetalColor_Absent=Brown, MinorPetalColor=Brown, PetalColorGroup1=WhiteBrown, PetalColorGroup2=WhiteBrown]

E:\QE Data\2020Aug5_Jessa'sFlowers_SetC\2020Aug5_Jessa_3_Neg\2020Aug5_043.raw

[S327] 2020Aug5_044 [Sample number=58, Population=F2, Female P=Aurora, Male P=Gelber, Color group=White with patterns, Floral organ=ST, Secondary color=brown, General main color=white, Main color=white, General secondary color=brown, ColorCategory=WhiteBrown, Red=No, Brown=Yes, Yellow=No, Purple=No, White=Yes, Pink=No, ColorCategories_Updated=WhiteBrown, FlowerCategories_W_ST=WhiteBrown, MainPetalColor=White, MinorPetalColor_Absent=Brown, MinorPetalColor=Brown, PetalColorGroup1=WhiteBrown, PetalColorGroup2=WhiteBrown]

E:\QE Data\2020Aug5_Jessa'sFlowers_SetC\2020Aug5_Jessa_3_Neg\2020Aug5_044.raw

[S328] 2020Aug5_045 [Sample number=65, Population=F2, Female P=Aurora, Male P=P47-1, Color group=White with patterns, Floral organ=W, Secondary color=brown, General main color=white, Main color=white, General secondary color=brown, ColorCategory=WhiteBrown, Red=No, Brown=Yes, Yellow=No, Purple=No, White=Yes, Pink=No, ColorCategories_Updated=WhiteBrown, FlowerCategories_W_ST=WhiteBrown, MainPetalColor=White, MinorPetalColor_Absent=Brown, MinorPetalColor=Brown, PetalColorGroup1=WhiteBrown, PetalColorGroup2=WhiteBrown]

E:\QE Data\2020Aug5_Jessa'sFlowers_SetC\2020Aug5_Jessa_3_Neg\2020Aug5_045.raw

[S329] 2020Aug5_046 [Sample number=66, Population=F2, Female P=Aurora, Male P=P47-1, Color group=White with patterns, Floral organ=ST, Secondary color=brown, General main color=white, Main color=white, General secondary color=brown, ColorCategory=WhiteBrown, Red=No, Brown=Yes, Yellow=No, Purple=No, White=Yes, Pink=No, ColorCategories_Updated=WhiteBrown, FlowerCategories_W_ST=WhiteBrown, MainPetalColor=White, MinorPetalColor_Absent=Brown, MinorPetalColor=Brown, PetalColorGroup1=WhiteBrown, PetalColorGroup2=WhiteBrown]

E:\QE Data\2020Aug5_Jessa'sFlowers_SetC\2020Aug5_Jessa_3_Neg\2020Aug5_046.raw

[S333] 2020Aug5_050 [Sample number=n/a, Population=n/a, Female P=n/a, Male P=n/a, Color group=n/a, Floral organ=n/a, Secondary color=n/a, General main color=n/a, Main color=n/a, General secondary color=n/a, ColorCategory=n/a, Red=n/a, Brown=n/a, Yellow=n/a, Purple=n/a, White=n/a, Pink=n/a, ColorCategories_Updated=n/a, FlowerCategories_W_ST=n/a, MainPetalColor=n/a, MinorPetalColor_Absent=n/a, MinorPetalColor=n/a, PetalColorGroup1=n/a, PetalColorGroup2=n/a]

E:\QE Data\2020Aug5_Jessa'sFlowers_SetC\2020Aug5_Jessa_3_Neg\2020Aug5_050.raw

[S334] 2020Aug5_051 [Sample number=n/a, Population=n/a, Female P=n/a, Male P=n/a, Color group=n/a, Floral organ=n/a, Secondary color=n/a, General main color=n/a, Main color=n/a, General secondary color=n/a, ColorCategory=n/a, Red=n/a, Brown=n/a, Yellow=n/a, Purple=n/a, White=n/a, Pink=n/a, ColorCategories_Updated=n/a, FlowerCategories_W_ST=n/a, MainPetalColor=n/a, MinorPetalColor_Absent=n/a, MinorPetalColor=n/a, PetalColorGroup1=n/a, PetalColorGroup2=n/a]

E:\QE Data\2020Aug5_Jessa'sFlowers_SetC\2020Aug5_Jessa_3_Neg\2020Aug5_051.raw

[S335] 2020Aug5_052 [Sample number=1, Population=F2, Female P=Gelber, Male P=P47-1, Color group=Brown, Floral organ=W, Secondary color=brown, General main color=brown, Main color=brown, General secondary color=brown, ColorCategory=Brown, Red=No, Brown=Yes, Yellow=No, Purple=No, White=No, Pink=No, ColorCategories_Updated=Brown, FlowerCategories_W_ST=Brown, MainPetalColor=Brown, MinorPetalColor_Absent=Absent, MinorPetalColor=Brown, PetalColorGroup1=Brown, PetalColorGroup2=Brown]

E:\QE Data\2020Aug5_Jessa'sFlowers_SetC\2020Aug5_Jessa_3_Neg\2020Aug5_052.raw

[S336] 2020Aug5_053 [Sample number=2, Population=F2, Female P=Gelber, Male P=P47-1, Color group=Brown, Floral organ=ST, Secondary color=brown, General main color=brown, Main color=brown, General secondary color=brown, ColorCategory=Brown, Red=No, Brown=Yes, Yellow=No, Purple=No, White=No, Pink=No, ColorCategories_Updated=Brown, FlowerCategories_W_ST=Brown, MainPetalColor=Brown, MinorPetalColor_Absent=Absent, MinorPetalColor=Brown, PetalColorGroup1=Brown, PetalColorGroup2=Brown]

E:\QE Data\2020Aug5_Jessa'sFlowers_SetC\2020Aug5_Jessa_3_Neg\2020Aug5_053.raw

[S337] 2020Aug5_054 [Sample number=3, Population=F2, Female P=Gelber, Male P=P47-1, Color group=Brown, Floral organ=W, Secondary color=brown, General main color=brown, Main color=brown, General secondary color=brown, ColorCategory=Brown, Red=No, Brown=Yes, Yellow=No, Purple=No, White=No, Pink=No, ColorCategories_Updated=Brown, FlowerCategories_W_ST=Brown, MainPetalColor=Brown, MinorPetalColor_Absent=Absent, MinorPetalColor=Brown, PetalColorGroup1=Brown, PetalColorGroup2=Brown]

E:\QE Data\2020Aug5_Jessa'sFlowers_SetC\2020Aug5_Jessa_3_Neg\2020Aug5_054.raw

[S338] 2020Aug5_055 [Sample number=4, Population=F2, Female P=Gelber, Male P=P47-1, Color group=Brown, Floral organ=ST, Secondary color=brown, General main color=brown, Main color=brown, General secondary color=brown, ColorCategory=Brown, Red=No, Brown=Yes, Yellow=No, Purple=No, White=No, Pink=No, ColorCategories_Updated=Brown, FlowerCategories_W_ST=Brown, MainPetalColor=Brown, MinorPetalColor_Absent=Absent, MinorPetalColor=Brown, PetalColorGroup1=Brown, PetalColorGroup2=Brown]

E:\QE Data\2020Aug5_Jessa'sFlowers_SetC\2020Aug5_Jessa_3_Neg\2020Aug5_055.raw

[S339] 2020Aug5_056 [Sample number=6, Population=F2, Female P=Gelber, Male P=P47-1, Color group=Brown, Floral organ=W, Secondary color=brown, General main color=brown, Main color=brown, General secondary color=brown, ColorCategory=Brown, Red=No, Brown=Yes, Yellow=No, Purple=No, White=No, Pink=No, ColorCategories_Updated=Brown, FlowerCategories_W_ST=BrownRed, MainPetalColor=Brown, MinorPetalColor_Absent=Absent, MinorPetalColor=Brown, PetalColorGroup1=Brown, PetalColorGroup2=Brown]

E:\QE Data\2020Aug5_Jessa'sFlowers_SetC\2020Aug5_Jessa_3_Neg\2020Aug5_056.raw

[S342] 2020Aug5_059 [Sample number=36, Population=F2, Female P=P47-1, Male P=Disco/2, Color group=Brown, Floral organ=ST, Secondary color=brown, General main color=brown, Main color=brown, General secondary color=brown, ColorCategory=Brown, Red=No, Brown=Yes, Yellow=No, Purple=No, White=No, Pink=No, ColorCategories_Updated=Brown, FlowerCategories_W_ST=Brown, MainPetalColor=Brown, MinorPetalColor_Absent=Absent, MinorPetalColor=Brown, PetalColorGroup1=Brown, PetalColorGroup2=Brown]

E:\QE Data\2020Aug5_Jessa'sFlowers_SetC\2020Aug5_Jessa_3_Neg\2020Aug5_059.raw

[S343] 2020Aug5_060 [Sample number=n/a, Population=n/a, Female P=n/a, Male P=n/a, Color group=n/a, Floral organ=n/a, Secondary color=n/a, General main color=n/a, Main color=n/a, General secondary color=n/a, ColorCategory=n/a, Red=n/a, Brown=n/a, Yellow=n/a, Purple=n/a, White=n/a, Pink=n/a, ColorCategories_Updated=n/a, FlowerCategories_W_ST=n/a, MainPetalColor=n/a, MinorPetalColor_Absent=n/a, MinorPetalColor=n/a, PetalColorGroup1=n/a, PetalColorGroup2=n/a]

E:\QE Data\2020Aug5_Jessa'sFlowers_SetC\2020Aug5_Jessa_3_Neg\2020Aug5_060.raw

[S344] 2020Aug5_061 [Sample number=n/a, Population=n/a, Female P=n/a, Male P=n/a, Color group=n/a, Floral organ=n/a, Secondary color=n/a, General main color=n/a, Main color=n/a, General secondary color=n/a, ColorCategory=n/a, Red=n/a, Brown=n/a, Yellow=n/a, Purple=n/a, White=n/a, Pink=n/a, ColorCategories_Updated=n/a, FlowerCategories_W_ST=n/a, MainPetalColor=n/a, MinorPetalColor_Absent=n/a, MinorPetalColor=n/a, PetalColorGroup1=n/a, PetalColorGroup2=n/a]

E:\QE Data\2020Aug5_Jessa'sFlowers_SetC\2020Aug5_Jessa_3_Neg\2020Aug5_061.raw

[S345] 2020Aug5_062 [Sample number=5, Population=F2, Female P=Gelber, Male P=P47-1, Color group=Brown mixed, Floral organ=ST, Secondary color=red, General main color=brown, Main color=brown, General secondary color=red, ColorCategory=RedBrown, Red=Yes, Brown=Yes, Yellow=No, Purple=No, White=No, Pink=No, ColorCategories_Updated=BrownRed, FlowerCategories_W_ST=BrownRed, MainPetalColor=Brown, MinorPetalColor_Absent=Red, MinorPetalColor=Red, PetalColorGroup1=BrownRed, PetalColorGroup2=BrownRed-RedBrown]

E:\QE Data\2020Aug5_Jessa'sFlowers_SetC\2020Aug5_Jessa_3_Neg\2020Aug5_062.raw

[S346] 2020Aug5_063 [Sample number=7, Population=F2, Female P=Gelber, Male P=P47-1, Color group=Brown mixed, Floral organ=W, Secondary color=pink, General main color=brown, Main color=brown, General secondary color=red, ColorCategory=RedBrown, Red=No, Brown=Yes, Yellow=No, Purple=No, White=No, Pink=Yes, ColorCategories_Updated=BrownPink, FlowerCategories_W_ST=BrownPink, MainPetalColor=Brown, MinorPetalColor_Absent=Pink, MinorPetalColor=Pink, PetalColorGroup1=BrownPink, PetalColorGroup2=BrownPink-PinkBrown]

E:\QE Data\2020Aug5_Jessa'sFlowers_SetC\2020Aug5_Jessa_3_Neg\2020Aug5_063.raw

[S347] 2020Aug5_064 [Sample number=9, Population=F2, Female P=Gelber, Male P=P47-1, Color group=Brown mixed, Floral organ=W, Secondary color=pink, General main color=brown, Main color=brown, General secondary color=red, ColorCategory=RedBrown, Red=No, Brown=Yes, Yellow=No, Purple=No, White=No, Pink=Yes, ColorCategories_Updated=BrownPink, FlowerCategories_W_ST=BrownPink, MainPetalColor=Brown, MinorPetalColor_Absent=Pink, MinorPetalColor=Pink, PetalColorGroup1=BrownPink, PetalColorGroup2=BrownPink-PinkBrown]

E:\QE Data\2020Aug5_Jessa'sFlowers_SetC\2020Aug5_Jessa_3_Neg\2020Aug5_064.raw

[S348] 2020Aug5_065 [Sample number=11, Population=F2, Female P=Snowdrop, Male P=P47-1, Color group=Brown mixed, Floral organ=W, Secondary color=pink, General main color=brown, Main color=brown, General secondary color=red, ColorCategory=RedBrown, Red=No, Brown=Yes, Yellow=No, Purple=No, White=No, Pink=Yes, ColorCategories_Updated=BrownPink, FlowerCategories_W_ST=BrownPink, MainPetalColor=Brown, MinorPetalColor_Absent=Pink, MinorPetalColor=Pink, PetalColorGroup1=BrownPink, PetalColorGroup2=BrownPink-PinkBrown]

E:\QE Data\2020Aug5_Jessa'sFlowers_SetC\2020Aug5_Jessa_3_Neg\2020Aug5_065.raw

[S349] 2020Aug5_066 [Sample number=14, Population=F2, Female P=Snowdrop, Male P=P47-1, Color group=Red, Floral organ=W, Secondary color=red, General main color=brown, Main color=brown, General secondary color=red, ColorCategory=RedBrown, Red=Yes, Brown=Yes, Yellow=No, Purple=No, White=No, Pink=No, ColorCategories_Updated=BrownRed, FlowerCategories_W_ST=BrownRed, MainPetalColor=Brown, MinorPetalColor_Absent=Red, MinorPetalColor=Red, PetalColorGroup1=BrownRed, PetalColorGroup2=BrownRed-RedBrown]

E:\QE Data\2020Aug5_Jessa'sFlowers_SetC\2020Aug5_Jessa_3_Neg\2020Aug5_066.raw

[S350] 2020Aug5_067 [Sample number=33, Population=F2, Female P=P47-1, Male P=Disco/2, Color group=Brown mixed, Floral organ=W, Secondary color=red, General main color=brown, Main color=brown, General secondary color=red, ColorCategory=RedBrown, Red=Yes, Brown=Yes, Yellow=No, Purple=No, White=No, Pink=No, ColorCategories_Updated=BrownRed, FlowerCategories_W_ST=BrownRed, MainPetalColor=Brown, MinorPetalColor_Absent=Red, MinorPetalColor=Red, PetalColorGroup1=BrownRed, PetalColorGroup2=BrownRed-RedBrown]

E:\QE Data\2020Aug5_Jessa'sFlowers_SetC\2020Aug5_Jessa_3_Neg\2020Aug5_067.raw

[S351] 2020Aug5_068 [Sample number=35, Population=F2, Female P=P47-1, Male P=Disco/2, Color group=Brown mixed, Floral organ=W, Secondary color=brown, General main color=brown, Main color=brown, General secondary color=red, ColorCategory=BrownPurple, Red=No, Brown=Yes, Yellow=No, Purple=Yes, White=No, Pink=No, ColorCategories_Updated=Brown, FlowerCategories_W_ST=Brown, MainPetalColor=Brown, MinorPetalColor_Absent=Absent, MinorPetalColor=Brown, PetalColorGroup1=Brown, PetalColorGroup2=Brown]

E:\QE Data\2020Aug5_Jessa'sFlowers_SetC\2020Aug5_Jessa_3_Neg\2020Aug5_068.raw

[S352] 2020Aug5_069 [Sample number=63, Population=F2, Female P=Aurora, Male P=P47-1, Color group=Brown mixed, Floral organ=W, Secondary color=pink, General main color=brown, Main color=brown, General secondary color=red, ColorCategory=RedBrown, Red=No, Brown=Yes, Yellow=No, Purple=No, White=No, Pink=Yes, ColorCategories_Updated=BrownPink, FlowerCategories_W_ST=BrownPink, MainPetalColor=Brown, MinorPetalColor_Absent=Pink, MinorPetalColor=Pink, PetalColorGroup1=BrownPink, PetalColorGroup2=BrownPink-PinkBrown]

E:\QE Data\2020Aug5_Jessa'sFlowers_SetC\2020Aug5_Jessa_3_Neg\2020Aug5_069.raw

[S353] 2020Aug5_070 [Sample number=n/a, Population=n/a, Female P=n/a, Male P=n/a, Color group=n/a, Floral organ=n/a, Secondary color=n/a, General main color=n/a, Main color=n/a, General secondary color=n/a, ColorCategory=n/a, Red=n/a, Brown=n/a, Yellow=n/a, Purple=n/a, White=n/a, Pink=n/a, ColorCategories_Updated=n/a, FlowerCategories_W_ST=n/a, MainPetalColor=n/a, MinorPetalColor_Absent=n/a, MinorPetalColor=n/a, PetalColorGroup1=n/a, PetalColorGroup2=n/a]

E:\QE Data\2020Aug5_Jessa'sFlowers_SetC\2020Aug5_Jessa_3_Neg\2020Aug5_070.raw

[S354] 2020Aug5_071 [Sample number=n/a, Population=n/a, Female P=n/a, Male P=n/a, Color group=n/a, Floral organ=n/a, Secondary color=n/a, General main color=n/a, Main color=n/a, General secondary color=n/a, ColorCategory=n/a, Red=n/a, Brown=n/a, Yellow=n/a, Purple=n/a, White=n/a, Pink=n/a, ColorCategories_Updated=n/a, FlowerCategories_W_ST=n/a, MainPetalColor=n/a, MinorPetalColor_Absent=n/a, MinorPetalColor=n/a, PetalColorGroup1=n/a, PetalColorGroup2=n/a]

E:\QE Data\2020Aug5_Jessa'sFlowers_SetC\2020Aug5_Jessa_3_Neg\2020Aug5_071.raw

[S355] 2020Aug5_072 [Sample number=8, Population=F2, Female P=Gelber, Male P=P47-1, Color group=Brown mixed, Floral organ=ST, Secondary color=brown, General main color=red, Main color=pink, General secondary color=brown, ColorCategory=RedBrown, Red=No, Brown=Yes, Yellow=No, Purple=No, White=No, Pink=Yes, ColorCategories_Updated=BrownPink, FlowerCategories_W_ST=BrownPink, MainPetalColor=Pink, MinorPetalColor_Absent=Brown, MinorPetalColor=Brown, PetalColorGroup1=PinkBrown, PetalColorGroup2=BrownPink-PinkBrown]

E:\QE Data\2020Aug5_Jessa'sFlowers_SetC\2020Aug5_Jessa_3_Neg\2020Aug5_072.raw

[S356] 2020Aug5_073 [Sample number=10, Population=F2, Female P=Gelber, Male P=P47-1, Color group=Brown mixed, Floral organ=ST, Secondary color=brown, General main color=red, Main color=pink, General secondary color=brown, ColorCategory=RedBrown, Red=No, Brown=Yes, Yellow=No, Purple=No, White=No, Pink=Yes, ColorCategories_Updated=BrownPink, FlowerCategories_W_ST=BrownPink, MainPetalColor=Pink, MinorPetalColor_Absent=Brown, MinorPetalColor=Brown, PetalColorGroup1=PinkBrown, PetalColorGroup2=BrownPink-PinkBrown]

E:\QE Data\2020Aug5_Jessa'sFlowers_SetC\2020Aug5_Jessa_3_Neg\2020Aug5_073.raw

[S357] 2020Aug5_074 [Sample number=12, Population=F2, Female P=Snowdrop, Male P=P47-1, Color group=Brown mixed, Floral organ=ST, Secondary color=brown, General main color=red, Main color=pink, General secondary color=brown, ColorCategory=RedBrown, Red=No, Brown=Yes, Yellow=No, Purple=No, White=No, Pink=Yes, ColorCategories_Updated=BrownPink, FlowerCategories_W_ST=BrownPink, MainPetalColor=Pink, MinorPetalColor_Absent=Brown, MinorPetalColor=Brown, PetalColorGroup1=PinkBrown, PetalColorGroup2=BrownPink-PinkBrown]

E:\QE Data\2020Aug5_Jessa'sFlowers_SetC\2020Aug5_Jessa_3_Neg\2020Aug5_074.raw

[S358] 2020Aug5_075 [Sample number=17, Population=F2, Female P=Snowdrop, Male P=P47-1, Color group=Red, Floral organ=W, Secondary color=brown, General main color=red, Main color=red, General secondary color=brown, ColorCategory=RedBrown, Red=Yes, Brown=Yes, Yellow=No, Purple=No, White=No, Pink=No, ColorCategories_Updated=BrownRed, FlowerCategories_W_ST=BrownRed, MainPetalColor=Red, MinorPetalColor_Absent=Brown, MinorPetalColor=Brown, PetalColorGroup1=RedBrown, PetalColorGroup2=BrownRed-RedBrown]

E:\QE Data\2020Aug5_Jessa'sFlowers_SetC\2020Aug5_Jessa_3_Neg\2020Aug5_075.raw

[S359] 2020Aug5_076 [Sample number=18, Population=F2, Female P=Snowdrop, Male P=P47-1, Color group=Red, Floral organ=ST, Secondary color=brown, General main color=red, Main color=red, General secondary color=brown, ColorCategory=RedBrown, Red=Yes, Brown=Yes, Yellow=No, Purple=No, White=No, Pink=No, ColorCategories_Updated=BrownRed, FlowerCategories_W_ST=BrownRed, MainPetalColor=Red, MinorPetalColor_Absent=Brown, MinorPetalColor=Brown, PetalColorGroup1=RedBrown, PetalColorGroup2=BrownRed-RedBrown]

E:\QE Data\2020Aug5_Jessa'sFlowers_SetC\2020Aug5_Jessa_3_Neg\2020Aug5_076.raw

[S361] 2020Aug5_078 [Sample number=21, Population=F2, Female P=Snowdrop, Male P=P47-1, Color group=Red, Floral organ=W, Secondary color=brown, General main color=red, Main color=red, General secondary color=brown, ColorCategory=RedBrown, Red=Yes, Brown=Yes, Yellow=No, Purple=No, White=No, Pink=No, ColorCategories_Updated=BrownRed, FlowerCategories_W_ST=BrownRed, MainPetalColor=Red, MinorPetalColor_Absent=Brown, MinorPetalColor=Brown, PetalColorGroup1=RedBrown, PetalColorGroup2=BrownRed-RedBrown]

E:\QE Data\2020Aug5_Jessa'sFlowers_SetC\2020Aug5_Jessa_3_Neg\2020Aug5_078.raw

[S362] 2020Aug5_079 [Sample number=31, Population=F2, Female P=P47-1, Male P=Disco/2, Color group=Brown mixed, Floral organ=W, Secondary color=brown, General main color=red, Main color=pink, General secondary color=brown, ColorCategory=RedBrown, Red=No, Brown=Yes, Yellow=No, Purple=No, White=No, Pink=Yes, ColorCategories_Updated=BrownPink, FlowerCategories_W_ST=BrownPink, MainPetalColor=Pink, MinorPetalColor_Absent=Brown, MinorPetalColor=Brown, PetalColorGroup1=PinkBrown, PetalColorGroup2=BrownPink-PinkBrown]

E:\QE Data\2020Aug5_Jessa'sFlowers_SetC\2020Aug5_Jessa_3_Neg\2020Aug5_079.raw

[S363] 2020Aug5_080 [Sample number=32, Population=F2, Female P=P47-1, Male P=Disco/2, Color group=Brown mixed, Floral organ=ST, Secondary color=brown, General main color=red, Main color=pink, General secondary color=brown, ColorCategory=RedBrown, Red=No, Brown=Yes, Yellow=No, Purple=No, White=No, Pink=Yes, ColorCategories_Updated=BrownPink, FlowerCategories_W_ST=BrownPink, MainPetalColor=Pink, MinorPetalColor_Absent=Brown, MinorPetalColor=Brown, PetalColorGroup1=PinkBrown, PetalColorGroup2=BrownPink-PinkBrown]

E:\QE Data\2020Aug5_Jessa'sFlowers_SetC\2020Aug5_Jessa_3_Neg\2020Aug5_080.raw

[S364] 2020Aug5_081 [Sample number=n/a, Population=n/a, Female P=n/a, Male P=n/a, Color group=n/a, Floral organ=n/a, Secondary color=n/a, General main color=n/a, Main color=n/a, General secondary color=n/a, ColorCategory=n/a, Red=n/a, Brown=n/a, Yellow=n/a, Purple=n/a, White=n/a, Pink=n/a, ColorCategories_Updated=n/a, FlowerCategories_W_ST=n/a, MainPetalColor=n/a, MinorPetalColor_Absent=n/a, MinorPetalColor=n/a, PetalColorGroup1=n/a, PetalColorGroup2=n/a]

E:\QE Data\2020Aug5_Jessa'sFlowers_SetC\2020Aug5_Jessa_3_Neg\2020Aug5_081.raw

[S365] 2020Aug5_082 [Sample number=n/a, Population=n/a, Female P=n/a, Male P=n/a, Color group=n/a, Floral organ=n/a, Secondary color=n/a, General main color=n/a, Main color=n/a, General secondary color=n/a, ColorCategory=n/a, Red=n/a, Brown=n/a, Yellow=n/a, Purple=n/a, White=n/a, Pink=n/a, ColorCategories_Updated=n/a, FlowerCategories_W_ST=n/a, MainPetalColor=n/a, MinorPetalColor_Absent=n/a, MinorPetalColor=n/a, PetalColorGroup1=n/a, PetalColorGroup2=n/a]

E:\QE Data\2020Aug5_Jessa'sFlowers_SetC\2020Aug5_Jessa_3_Neg\2020Aug5_082.raw

[S366] 2020Aug5_083 [Sample number=34, Population=F2, Female P=P47-1, Male P=Disco/2, Color group=Red, Floral organ=ST, Secondary color=brown, General main color=red, Main color=red, General secondary color=brown, ColorCategory=RedBrown, Red=Yes, Brown=Yes, Yellow=No, Purple=No, White=No, Pink=No, ColorCategories_Updated=BrownRed, FlowerCategories_W_ST=BrownRed, MainPetalColor=Red, MinorPetalColor_Absent=Brown, MinorPetalColor=Brown, PetalColorGroup1=RedBrown, PetalColorGroup2=BrownRed-RedBrown]

E:\QE Data\2020Aug5_Jessa'sFlowers_SetC\2020Aug5_Jessa_3_Neg\2020Aug5_083.raw

[S367] 2020Aug5_084 [Sample number=61, Population=F2, Female P=Aurora, Male P=P47-1, Color group=Red, Floral organ=W, Secondary color=brown, General main color=red, Main color=red, General secondary color=brown, ColorCategory=RedBrown, Red=Yes, Brown=Yes, Yellow=No, Purple=No, White=No, Pink=No, ColorCategories_Updated=BrownRed, FlowerCategories_W_ST=BrownRed, MainPetalColor=Red, MinorPetalColor_Absent=Brown, MinorPetalColor=Brown, PetalColorGroup1=RedBrown, PetalColorGroup2=BrownRed-RedBrown]

E:\QE Data\2020Aug5_Jessa'sFlowers_SetC\2020Aug5_Jessa_3_Neg\2020Aug5_084.raw

[S368] 2020Aug5_085 [Sample number=n/a, Population=n/a, Female P=n/a, Male P=n/a, Color group=n/a, Floral organ=n/a, Secondary color=n/a, General main color=n/a, Main color=n/a, General secondary color=n/a, ColorCategory=n/a, Red=n/a, Brown=n/a, Yellow=n/a, Purple=n/a, White=n/a, Pink=n/a, ColorCategories_Updated=n/a, FlowerCategories_W_ST=n/a, MainPetalColor=n/a, MinorPetalColor_Absent=n/a, MinorPetalColor=n/a, PetalColorGroup1=n/a, PetalColorGroup2=n/a]

E:\QE Data\2020Aug5_Jessa'sFlowers_SetC\2020Aug5_Jessa_3_Neg\2020Aug5_085.raw

[S369] 2020Aug5_086 [Sample number=13, Population=F2, Female P=Snowdrop, Male P=P47-1, Color group=Red, Floral organ=ST, Secondary color=red, General main color=red, Main color=red, General secondary color=red, ColorCategory=Red, Red=Yes, Brown=No, Yellow=No, Purple=No, White=No, Pink=No, ColorCategories_Updated=Red, FlowerCategories_W_ST=BrownRed, MainPetalColor=Red, MinorPetalColor_Absent=Absent, MinorPetalColor=Red, PetalColorGroup1=Red, PetalColorGroup2=Red]

E:\QE Data\2020Aug5_Jessa'sFlowers_SetC\2020Aug5_Jessa_3_Neg\2020Aug5_086.raw

[S371] 2020Aug5_088 [Sample number=22, Population=F2, Female P=Snowdrop, Male P=P47-1, Color group=Red, Floral organ=ST, Secondary color=red, General main color=red, Main color=red, General secondary color=red, ColorCategory=Red, Red=Yes, Brown=No, Yellow=No, Purple=No, White=No, Pink=No, ColorCategories_Updated=Red, FlowerCategories_W_ST=BrownRed, MainPetalColor=Red, MinorPetalColor_Absent=Absent, MinorPetalColor=Red, PetalColorGroup1=Red, PetalColorGroup2=Red]

E:\QE Data\2020Aug5_Jessa'sFlowers_SetC\2020Aug5_Jessa_3_Neg\2020Aug5_088.raw

[S372] 2020Aug5_089 [Sample number=62, Population=F2, Female P=Aurora, Male P=P47-1, Color group=Red, Floral organ=ST, Secondary color=red, General main color=red, Main color=red, General secondary color=red, ColorCategory=Red, Red=Yes, Brown=No, Yellow=No, Purple=No, White=No, Pink=No, ColorCategories_Updated=Red, FlowerCategories_W_ST=BrownRed, MainPetalColor=Red, MinorPetalColor_Absent=Absent, MinorPetalColor=Red, PetalColorGroup1=Red, PetalColorGroup2=Red]

E:\QE Data\2020Aug5_Jessa'sFlowers_SetC\2020Aug5_Jessa_3_Neg\2020Aug5_089.raw

[S373] 2020Aug5_090 [Sample number=64, Population=F2, Female P=Aurora, Male P=P47-1, Color group=Brown mixed, Floral organ=ST, Secondary color=pink, General main color=red, Main color=pink, General secondary color=red, ColorCategory=Red, Red=No, Brown=No, Yellow=No, Purple=No, White=No, Pink=Yes, ColorCategories_Updated=BrownPink, FlowerCategories_W_ST=BrownPink, MainPetalColor=Pink, MinorPetalColor_Absent=Absent, MinorPetalColor=Pink, PetalColorGroup1=Pink, PetalColorGroup2=BrownPink-PinkBrown]

E:\QE Data\2020Aug5_Jessa'sFlowers_SetC\2020Aug5_Jessa_3_Neg\2020Aug5_090.raw

[S374] 2020Aug5_091 [Sample number=n/a, Population=n/a, Female P=n/a, Male P=n/a, Color group=n/a, Floral organ=n/a, Secondary color=n/a, General main color=n/a, Main color=n/a, General secondary color=n/a, ColorCategory=n/a, Red=n/a, Brown=n/a, Yellow=n/a, Purple=n/a, White=n/a, Pink=n/a, ColorCategories_Updated=n/a, FlowerCategories_W_ST=n/a, MainPetalColor=n/a, MinorPetalColor_Absent=n/a, MinorPetalColor=n/a, PetalColorGroup1=n/a, PetalColorGroup2=n/a]

E:\QE Data\2020Aug5_Jessa'sFlowers_SetC\2020Aug5_Jessa_3_Neg\2020Aug5_091.raw

[S375] 2020Aug5_092 [Sample number=n/a, Population=n/a, Female P=n/a, Male P=n/a, Color group=n/a, Floral organ=n/a, Secondary color=n/a, General main color=n/a, Main color=n/a, General secondary color=n/a, ColorCategory=n/a, Red=n/a, Brown=n/a, Yellow=n/a, Purple=n/a, White=n/a, Pink=n/a, ColorCategories_Updated=n/a, FlowerCategories_W_ST=n/a, MainPetalColor=n/a, MinorPetalColor_Absent=n/a, MinorPetalColor=n/a, PetalColorGroup1=n/a, PetalColorGroup2=n/a]

E:\QE Data\2020Aug5_Jessa'sFlowers_SetC\2020Aug5_Jessa_3_Neg\2020Aug5_092.raw

[S376] 2020Aug5_093 [Sample number=71, Population=2263, Female P=Rinrei, Male P=P47-1, Color group=Red, Floral organ=ST, Secondary color=purple, General main color=red, Main color=red, General secondary color=purple, ColorCategory=RedPurple, Red=Yes, Brown=No, Yellow=No, Purple=Yes, White=No, Pink=No, ColorCategories_Updated=RedPurple, FlowerCategories_W_ST=RedPurple, MainPetalColor=Purplish red, MinorPetalColor_Absent=Absent, MinorPetalColor=Purplish red, PetalColorGroup1=Purplish red, PetalColorGroup2=Purplish red]

E:\QE Data\2020Aug5_Jessa'sFlowers_SetC\2020Aug5_Jessa_3_Neg\2020Aug5_093.raw

[S377] 2020Aug5_094 [Sample number=72, Population=2263, Female P=Rinrei, Male P=P47-1, Color group=Red, Floral organ=W, Secondary color=purple, General main color=red, Main color=red, General secondary color=purple, ColorCategory=RedPurple, Red=Yes, Brown=No, Yellow=No, Purple=Yes, White=No, Pink=No, ColorCategories_Updated=RedPurple, FlowerCategories_W_ST=RedPurple, MainPetalColor=Purplish red, MinorPetalColor_Absent=Absent, MinorPetalColor=Purplish red, PetalColorGroup1=Purplish red, PetalColorGroup2=Purplish red]

E:\QE Data\2020Aug5_Jessa'sFlowers_SetC\2020Aug5_Jessa_3_Neg\2020Aug5_094.raw

[S378] 2020Aug5_095 [Sample number=75, Population=71B, Female P=P47-1, Male P=NV153, Color group=Red, Floral organ=W, Secondary color=purple, General main color=red, Main color=red, General secondary color=purple, ColorCategory=RedPurple, Red=Yes, Brown=No, Yellow=No, Purple=Yes, White=No, Pink=No, ColorCategories_Updated=RedPurple, FlowerCategories_W_ST=RedPurple, MainPetalColor=Purplish red, MinorPetalColor_Absent=Absent, MinorPetalColor=Purplish red, PetalColorGroup1=Purplish red, PetalColorGroup2=Purplish red]

E:\QE Data\2020Aug5_Jessa'sFlowers_SetC\2020Aug5_Jessa_3_Neg\2020Aug5_095.raw

[S379] 2020Aug5_096 [Sample number=76, Population=71B, Female P=P47-1, Male P=NV153, Color group=Red, Floral organ=ST, Secondary color=purple, General main color=red, Main color=red, General secondary color=purple, ColorCategory=RedPurple, Red=Yes, Brown=No, Yellow=No, Purple=Yes, White=No, Pink=No, ColorCategories_Updated=RedPurple, FlowerCategories_W_ST=RedPurple, MainPetalColor=Purplish red, MinorPetalColor_Absent=Absent, MinorPetalColor=Purplish red, PetalColorGroup1=Purplish red, PetalColorGroup2=Purplish red]

E:\QE Data\2020Aug5_Jessa'sFlowers_SetC\2020Aug5_Jessa_3_Neg\2020Aug5_096.raw

[S380] 2020Aug5_097 [Sample number=n/a, Population=n/a, Female P=n/a, Male P=n/a, Color group=n/a, Floral organ=n/a, Secondary color=n/a, General main color=n/a, Main color=n/a, General secondary color=n/a, ColorCategory=n/a, Red=n/a, Brown=n/a, Yellow=n/a, Purple=n/a, White=n/a, Pink=n/a, ColorCategories_Updated=n/a, FlowerCategories_W_ST=n/a, MainPetalColor=n/a, MinorPetalColor_Absent=n/a, MinorPetalColor=n/a, PetalColorGroup1=n/a, PetalColorGroup2=n/a]

E:\QE Data\2020Aug5_Jessa'sFlowers_SetC\2020Aug5_Jessa_3_Neg\2020Aug5_097.raw

[S381] 2020Aug5_098 [Sample number=n/a, Population=n/a, Female P=n/a, Male P=n/a, Color group=n/a, Floral organ=n/a, Secondary color=n/a, General main color=n/a, Main color=n/a, General secondary color=n/a, ColorCategory=n/a, Red=n/a, Brown=n/a, Yellow=n/a, Purple=n/a, White=n/a, Pink=n/a, ColorCategories_Updated=n/a, FlowerCategories_W_ST=n/a, MainPetalColor=n/a, MinorPetalColor_Absent=n/a, MinorPetalColor=n/a, PetalColorGroup1=n/a, PetalColorGroup2=n/a]

E:\QE Data\2020Aug5_Jessa'sFlowers_SetC\2020Aug5_Jessa_3_Neg\2020Aug5_098.raw

[S382] 2020Aug5_099 [Sample number=n/a, Population=n/a, Female P=n/a, Male P=n/a, Color group=n/a, Floral organ=n/a, Secondary color=n/a, General main color=n/a, Main color=n/a, General secondary color=n/a, ColorCategory=n/a, Red=n/a, Brown=n/a, Yellow=n/a, Purple=n/a, White=n/a, Pink=n/a, ColorCategories_Updated=n/a, FlowerCategories_W_ST=n/a, MainPetalColor=n/a, MinorPetalColor_Absent=n/a, MinorPetalColor=n/a, PetalColorGroup1=n/a, PetalColorGroup2=n/a]

E:\QE Data\2020Aug5_Jessa'sFlowers_SetC\2020Aug5_Jessa_3_Neg\2020Aug5_099.raw

[S383] 2020Aug5_100 [Sample number=n/a, Population=n/a, Female P=n/a, Male P=n/a, Color group=n/a, Floral organ=n/a, Secondary color=n/a, General main color=n/a, Main color=n/a, General secondary color=n/a, ColorCategory=n/a, Red=n/a, Brown=n/a, Yellow=n/a, Purple=n/a, White=n/a, Pink=n/a, ColorCategories_Updated=n/a, FlowerCategories_W_ST=n/a, MainPetalColor=n/a, MinorPetalColor_Absent=n/a, MinorPetalColor=n/a, PetalColorGroup1=n/a, PetalColorGroup2=n/a]

E:\QE Data\2020Aug5_Jessa'sFlowers_SetC\2020Aug5_Jessa_3_Neg\2020Aug5_100.raw

[S386] 2020Aug5_103 [Sample number=n/a, Population=n/a, Female P=n/a, Male P=n/a, Color group=n/a, Floral organ=n/a, Secondary color=n/a, General main color=n/a, Main color=n/a, General secondary color=n/a, ColorCategory=n/a, Red=n/a, Brown=n/a, Yellow=n/a, Purple=n/a, White=n/a, Pink=n/a, ColorCategories_Updated=n/a, FlowerCategories_W_ST=n/a, MainPetalColor=n/a, MinorPetalColor_Absent=n/a, MinorPetalColor=n/a, PetalColorGroup1=n/a, PetalColorGroup2=n/a]

E:\QE Data\2020Aug5_Jessa'sFlowers_SetC\2020Aug5_Jessa_3_Neg\2020Aug5_103.raw

[S389] 2020Aug5_106 [Sample number=n/a, Population=n/a, Female P=n/a, Male P=n/a, Color group=n/a, Floral organ=n/a, Secondary color=n/a, General main color=n/a, Main color=n/a, General secondary color=n/a, ColorCategory=n/a, Red=n/a, Brown=n/a, Yellow=n/a, Purple=n/a, White=n/a, Pink=n/a, ColorCategories_Updated=n/a, FlowerCategories_W_ST=n/a, MainPetalColor=n/a, MinorPetalColor_Absent=n/a, MinorPetalColor=n/a, PetalColorGroup1=n/a, PetalColorGroup2=n/a]

E:\QE Data\2020Aug5_Jessa'sFlowers_SetC\2020Aug5_Jessa_3_Neg\2020Aug5_106.raw

[S392] 2020Aug5_109 [Sample number=n/a, Population=n/a, Female P=n/a, Male P=n/a, Color group=n/a, Floral organ=n/a, Secondary color=n/a, General main color=n/a, Main color=n/a, General secondary color=n/a, ColorCategory=n/a, Red=n/a, Brown=n/a, Yellow=n/a, Purple=n/a, White=n/a, Pink=n/a, ColorCategories_Updated=n/a, FlowerCategories_W_ST=n/a, MainPetalColor=n/a, MinorPetalColor_Absent=n/a, MinorPetalColor=n/a, PetalColorGroup1=n/a, PetalColorGroup2=n/a]

E:\QE Data\2020Aug5_Jessa'sFlowers_SetC\2020Aug5_Jessa_3_Neg\2020Aug5_109.raw

[S395] 2020Aug5_112 [Sample number=n/a, Population=n/a, Female P=n/a, Male P=n/a, Color group=n/a, Floral organ=n/a, Secondary color=n/a, General main color=n/a, Main color=n/a, General secondary color=n/a, ColorCategory=n/a, Red=n/a, Brown=n/a, Yellow=n/a, Purple=n/a, White=n/a, Pink=n/a, ColorCategories_Updated=n/a, FlowerCategories_W_ST=n/a, MainPetalColor=n/a, MinorPetalColor_Absent=n/a, MinorPetalColor=n/a, PetalColorGroup1=n/a, PetalColorGroup2=n/a]

E:\QE Data\2020Aug5_Jessa'sFlowers_SetC\2020Aug5_Jessa_3_Neg\2020Aug5_112.raw

[S396] 2020Aug5_113 [Sample number=n/a, Population=n/a, Female P=n/a, Male P=n/a, Color group=n/a, Floral organ=n/a, Secondary color=n/a, General main color=n/a, Main color=n/a, General secondary color=n/a, ColorCategory=n/a, Red=n/a, Brown=n/a, Yellow=n/a, Purple=n/a, White=n/a, Pink=n/a, ColorCategories_Updated=n/a, FlowerCategories_W_ST=n/a, MainPetalColor=n/a, MinorPetalColor_Absent=n/a, MinorPetalColor=n/a, PetalColorGroup1=n/a, PetalColorGroup2=n/a]

E:\QE Data\2020Aug5_Jessa'sFlowers_SetC\2020Aug5_Jessa_3_Neg\2020Aug5_113.raw

[S399] 2020Aug5_116 [Sample number=n/a, Population=n/a, Female P=n/a, Male P=n/a, Color group=n/a, Floral organ=n/a, Secondary color=n/a, General main color=n/a, Main color=n/a, General secondary color=n/a, ColorCategory=n/a, Red=n/a, Brown=n/a, Yellow=n/a, Purple=n/a, White=n/a, Pink=n/a, ColorCategories_Updated=n/a, FlowerCategories_W_ST=n/a, MainPetalColor=n/a, MinorPetalColor_Absent=n/a, MinorPetalColor=n/a, PetalColorGroup1=n/a, PetalColorGroup2=n/a]

E:\QE Data\2020Aug5_Jessa'sFlowers_SetC\2020Aug5_Jessa_3_Neg\2020Aug5_116.raw

[S402] 2020Aug5_119 [Sample number=n/a, Population=n/a, Female P=n/a, Male P=n/a, Color group=n/a, Floral organ=n/a, Secondary color=n/a, General main color=n/a, Main color=n/a, General secondary color=n/a, ColorCategory=n/a, Red=n/a, Brown=n/a, Yellow=n/a, Purple=n/a, White=n/a, Pink=n/a, ColorCategories_Updated=n/a, FlowerCategories_W_ST=n/a, MainPetalColor=n/a, MinorPetalColor_Absent=n/a, MinorPetalColor=n/a, PetalColorGroup1=n/a, PetalColorGroup2=n/a]

E:\QE Data\2020Aug5_Jessa'sFlowers_SetC\2020Aug5_Jessa_3_Neg\2020Aug5_119.raw

[S405] 2020Aug5_122 [Sample number=n/a, Population=n/a, Female P=n/a, Male P=n/a, Color group=n/a, Floral organ=n/a, Secondary color=n/a, General main color=n/a, Main color=n/a, General secondary color=n/a, ColorCategory=n/a, Red=n/a, Brown=n/a, Yellow=n/a, Purple=n/a, White=n/a, Pink=n/a, ColorCategories_Updated=n/a, FlowerCategories_W_ST=n/a, MainPetalColor=n/a, MinorPetalColor_Absent=n/a, MinorPetalColor=n/a, PetalColorGroup1=n/a, PetalColorGroup2=n/a]

E:\QE Data\2020Aug5_Jessa'sFlowers_SetC\2020Aug5_Jessa_3_Neg\2020Aug5_122.raw

## Grouping and Ratios

------------------------------------------------------------------

Grouping:

------------------------------------------------------------------

Study variable(s) for grouping:

Floral organ;PetalColorGroup2

------------------------------------------------------------------

Sample Groups:

------------------------------------------------------------------

n/a, n/a

F1: Blank, n/a, n/a, n/a, n/a, n/a, n/a, n/a, n/a, n/a, n/a, n/a, n/a, n/a, n/a, n/a, n/a, n/a, n/a, n/a, n/a, n/a, n/a, n/a, n/a

F2: Blank, n/a, n/a, n/a, n/a, n/a, n/a, n/a, n/a, n/a, n/a, n/a, n/a, n/a, n/a, n/a, n/a, n/a, n/a, n/a, n/a, n/a, n/a, n/a, n/a

F5: Blank, n/a, n/a, n/a, n/a, n/a, n/a, n/a, n/a, n/a, n/a, n/a, n/a, n/a, n/a, n/a, n/a, n/a, n/a, n/a, n/a, n/a, n/a, n/a, n/a

F6: IdentificationOnly, n/a, n/a, n/a, n/a, n/a, n/a, n/a, n/a, n/a, n/a, n/a, n/a, n/a, n/a, n/a, n/a, n/a, n/a, n/a, n/a, n/a, n/a, n/a, n/a

F7: IdentificationOnly, n/a, n/a, n/a, n/a, n/a, n/a, n/a, n/a, n/a, n/a, n/a, n/a, n/a, n/a, n/a, n/a, n/a, n/a, n/a, n/a, n/a, n/a, n/a, n/a

F8: IdentificationOnly, n/a, n/a, n/a, n/a, n/a, n/a, n/a, n/a, n/a, n/a, n/a, n/a, n/a, n/a, n/a, n/a, n/a, n/a, n/a, n/a, n/a, n/a, n/a, n/a

F9: IdentificationOnly, n/a, n/a, n/a, n/a, n/a, n/a, n/a, n/a, n/a, n/a, n/a, n/a, n/a, n/a, n/a, n/a, n/a, n/a, n/a, n/a, n/a, n/a, n/a, n/a

F10: IdentificationOnly, n/a, n/a, n/a, n/a, n/a, n/a, n/a, n/a, n/a, n/a, n/a, n/a, n/a, n/a, n/a, n/a, n/a, n/a, n/a, n/a, n/a, n/a, n/a, n/a

F11: IdentificationOnly, n/a, n/a, n/a, n/a, n/a, n/a, n/a, n/a, n/a, n/a, n/a, n/a, n/a, n/a, n/a, n/a, n/a, n/a, n/a, n/a, n/a, n/a, n/a, n/a

F12: QualityControl, n/a, n/a, n/a, n/a, n/a, n/a, n/a, n/a, n/a, n/a, n/a, n/a, n/a, n/a, n/a, n/a, n/a, n/a, n/a, n/a, n/a, n/a, n/a, n/a

F13: Blank, n/a, n/a, n/a, n/a, n/a, n/a, n/a, n/a, n/a, n/a, n/a, n/a, n/a, n/a, n/a, n/a, n/a, n/a, n/a, n/a, n/a, n/a, n/a, n/a

F20: QualityControl, n/a, n/a, n/a, n/a, n/a, n/a, n/a, n/a, n/a, n/a, n/a, n/a, n/a, n/a, n/a, n/a, n/a, n/a, n/a, n/a, n/a, n/a, n/a, n/a

F21: Blank, n/a, n/a, n/a, n/a, n/a, n/a, n/a, n/a, n/a, n/a, n/a, n/a, n/a, n/a, n/a, n/a, n/a, n/a, n/a, n/a, n/a, n/a, n/a, n/a

F28: QualityControl, n/a, n/a, n/a, n/a, n/a, n/a, n/a, n/a, n/a, n/a, n/a, n/a, n/a, n/a, n/a, n/a, n/a, n/a, n/a, n/a, n/a, n/a, n/a, n/a

F29: Blank, n/a, n/a, n/a, n/a, n/a, n/a, n/a, n/a, n/a, n/a, n/a, n/a, n/a, n/a, n/a, n/a, n/a, n/a, n/a, n/a, n/a, n/a, n/a, n/a

F36: QualityControl, n/a, n/a, n/a, n/a, n/a, n/a, n/a, n/a, n/a, n/a, n/a, n/a, n/a, n/a, n/a, n/a, n/a, n/a, n/a, n/a, n/a, n/a, n/a, n/a

F37: Blank, n/a, n/a, n/a, n/a, n/a, n/a, n/a, n/a, n/a, n/a, n/a, n/a, n/a, n/a, n/a, n/a, n/a, n/a, n/a, n/a, n/a, n/a, n/a, n/a

F44: QualityControl, n/a, n/a, n/a, n/a, n/a, n/a, n/a, n/a, n/a, n/a, n/a, n/a, n/a, n/a, n/a, n/a, n/a, n/a, n/a, n/a, n/a, n/a, n/a, n/a

F45: Blank, n/a, n/a, n/a, n/a, n/a, n/a, n/a, n/a, n/a, n/a, n/a, n/a, n/a, n/a, n/a, n/a, n/a, n/a, n/a, n/a, n/a, n/a, n/a, n/a

F52: QualityControl, n/a, n/a, n/a, n/a, n/a, n/a, n/a, n/a, n/a, n/a, n/a, n/a, n/a, n/a, n/a, n/a, n/a, n/a, n/a, n/a, n/a, n/a, n/a, n/a

F53: Blank, n/a, n/a, n/a, n/a, n/a, n/a, n/a, n/a, n/a, n/a, n/a, n/a, n/a, n/a, n/a, n/a, n/a, n/a, n/a, n/a, n/a, n/a, n/a, n/a

F60: QualityControl, n/a, n/a, n/a, n/a, n/a, n/a, n/a, n/a, n/a, n/a, n/a, n/a, n/a, n/a, n/a, n/a, n/a, n/a, n/a, n/a, n/a, n/a, n/a, n/a

F61: Blank, n/a, n/a, n/a, n/a, n/a, n/a, n/a, n/a, n/a, n/a, n/a, n/a, n/a, n/a, n/a, n/a, n/a, n/a, n/a, n/a, n/a, n/a, n/a, n/a

F66: Blank, n/a, n/a, n/a, n/a, n/a, n/a, n/a, n/a, n/a, n/a, n/a, n/a, n/a, n/a, n/a, n/a, n/a, n/a, n/a, n/a, n/a, n/a, n/a, n/a

F68: QualityControl, n/a, n/a, n/a, n/a, n/a, n/a, n/a, n/a, n/a, n/a, n/a, n/a, n/a, n/a, n/a, n/a, n/a, n/a, n/a, n/a, n/a, n/a, n/a, n/a

F69: Blank, n/a, n/a, n/a, n/a, n/a, n/a, n/a, n/a, n/a, n/a, n/a, n/a, n/a, n/a, n/a, n/a, n/a, n/a, n/a, n/a, n/a, n/a, n/a, n/a

F76: QualityControl, n/a, n/a, n/a, n/a, n/a, n/a, n/a, n/a, n/a, n/a, n/a, n/a, n/a, n/a, n/a, n/a, n/a, n/a, n/a, n/a, n/a, n/a, n/a, n/a

F77: Blank, n/a, n/a, n/a, n/a, n/a, n/a, n/a, n/a, n/a, n/a, n/a, n/a, n/a, n/a, n/a, n/a, n/a, n/a, n/a, n/a, n/a, n/a, n/a, n/a

F84: QualityControl, n/a, n/a, n/a, n/a, n/a, n/a, n/a, n/a, n/a, n/a, n/a, n/a, n/a, n/a, n/a, n/a, n/a, n/a, n/a, n/a, n/a, n/a, n/a, n/a

F85: Blank, n/a, n/a, n/a, n/a, n/a, n/a, n/a, n/a, n/a, n/a, n/a, n/a, n/a, n/a, n/a, n/a, n/a, n/a, n/a, n/a, n/a, n/a, n/a, n/a

F88: Blank, n/a, n/a, n/a, n/a, n/a, n/a, n/a, n/a, n/a, n/a, n/a, n/a, n/a, n/a, n/a, n/a, n/a, n/a, n/a, n/a, n/a, n/a, n/a, n/a

F92: QualityControl, n/a, n/a, n/a, n/a, n/a, n/a, n/a, n/a, n/a, n/a, n/a, n/a, n/a, n/a, n/a, n/a, n/a, n/a, n/a, n/a, n/a, n/a, n/a, n/a

F93: Blank, n/a, n/a, n/a, n/a, n/a, n/a, n/a, n/a, n/a, n/a, n/a, n/a, n/a, n/a, n/a, n/a, n/a, n/a, n/a, n/a, n/a, n/a, n/a, n/a

F100: QualityControl, n/a, n/a, n/a, n/a, n/a, n/a, n/a, n/a, n/a, n/a, n/a, n/a, n/a, n/a, n/a, n/a, n/a, n/a, n/a, n/a, n/a, n/a, n/a, n/a

F101: Blank, n/a, n/a, n/a, n/a, n/a, n/a, n/a, n/a, n/a, n/a, n/a, n/a, n/a, n/a, n/a, n/a, n/a, n/a, n/a, n/a, n/a, n/a, n/a, n/a

F107: QualityControl, n/a, n/a, n/a, n/a, n/a, n/a, n/a, n/a, n/a, n/a, n/a, n/a, n/a, n/a, n/a, n/a, n/a, n/a, n/a, n/a, n/a, n/a, n/a, n/a

F108: Blank, n/a, n/a, n/a, n/a, n/a, n/a, n/a, n/a, n/a, n/a, n/a, n/a, n/a, n/a, n/a, n/a, n/a, n/a, n/a, n/a, n/a, n/a, n/a, n/a

F115: QualityControl, n/a, n/a, n/a, n/a, n/a, n/a, n/a, n/a, n/a, n/a, n/a, n/a, n/a, n/a, n/a, n/a, n/a, n/a, n/a, n/a, n/a, n/a, n/a, n/a

F116: Blank, n/a, n/a, n/a, n/a, n/a, n/a, n/a, n/a, n/a, n/a, n/a, n/a, n/a, n/a, n/a, n/a, n/a, n/a, n/a, n/a, n/a, n/a, n/a, n/a

F123: QualityControl, n/a, n/a, n/a, n/a, n/a, n/a, n/a, n/a, n/a, n/a, n/a, n/a, n/a, n/a, n/a, n/a, n/a, n/a, n/a, n/a, n/a, n/a, n/a, n/a

F159: Blank, n/a, n/a, n/a, n/a, n/a, n/a, n/a, n/a, n/a, n/a, n/a, n/a, n/a, n/a, n/a, n/a, n/a, n/a, n/a, n/a, n/a, n/a, n/a, n/a

F160: Blank, n/a, n/a, n/a, n/a, n/a, n/a, n/a, n/a, n/a, n/a, n/a, n/a, n/a, n/a, n/a, n/a, n/a, n/a, n/a, n/a, n/a, n/a, n/a, n/a

F161: Blank, n/a, n/a, n/a, n/a, n/a, n/a, n/a, n/a, n/a, n/a, n/a, n/a, n/a, n/a, n/a, n/a, n/a, n/a, n/a, n/a, n/a, n/a, n/a, n/a

F163: Blank, n/a, n/a, n/a, n/a, n/a, n/a, n/a, n/a, n/a, n/a, n/a, n/a, n/a, n/a, n/a, n/a, n/a, n/a, n/a, n/a, n/a, n/a, n/a, n/a

F164: QualityControl, n/a, n/a, n/a, n/a, n/a, n/a, n/a, n/a, n/a, n/a, n/a, n/a, n/a, n/a, n/a, n/a, n/a, n/a, n/a, n/a, n/a, n/a, n/a, n/a

F165: Blank, n/a, n/a, n/a, n/a, n/a, n/a, n/a, n/a, n/a, n/a, n/a, n/a, n/a, n/a, n/a, n/a, n/a, n/a, n/a, n/a, n/a, n/a, n/a, n/a

F174: QualityControl, n/a, n/a, n/a, n/a, n/a, n/a, n/a, n/a, n/a, n/a, n/a, n/a, n/a, n/a, n/a, n/a, n/a, n/a, n/a, n/a, n/a, n/a, n/a, n/a

F175: Blank, n/a, n/a, n/a, n/a, n/a, n/a, n/a, n/a, n/a, n/a, n/a, n/a, n/a, n/a, n/a, n/a, n/a, n/a, n/a, n/a, n/a, n/a, n/a, n/a

F186: QualityControl, n/a, n/a, n/a, n/a, n/a, n/a, n/a, n/a, n/a, n/a, n/a, n/a, n/a, n/a, n/a, n/a, n/a, n/a, n/a, n/a, n/a, n/a, n/a, n/a

F187: Blank, n/a, n/a, n/a, n/a, n/a, n/a, n/a, n/a, n/a, n/a, n/a, n/a, n/a, n/a, n/a, n/a, n/a, n/a, n/a, n/a, n/a, n/a, n/a, n/a

F197: QualityControl, n/a, n/a, n/a, n/a, n/a, n/a, n/a, n/a, n/a, n/a, n/a, n/a, n/a, n/a, n/a, n/a, n/a, n/a, n/a, n/a, n/a, n/a, n/a, n/a

F198: Blank, n/a, n/a, n/a, n/a, n/a, n/a, n/a, n/a, n/a, n/a, n/a, n/a, n/a, n/a, n/a, n/a, n/a, n/a, n/a, n/a, n/a, n/a, n/a, n/a

F208: QualityControl, n/a, n/a, n/a, n/a, n/a, n/a, n/a, n/a, n/a, n/a, n/a, n/a, n/a, n/a, n/a, n/a, n/a, n/a, n/a, n/a, n/a, n/a, n/a, n/a

F209: Blank, n/a, n/a, n/a, n/a, n/a, n/a, n/a, n/a, n/a, n/a, n/a, n/a, n/a, n/a, n/a, n/a, n/a, n/a, n/a, n/a, n/a, n/a, n/a, n/a

F218: QualityControl, n/a, n/a, n/a, n/a, n/a, n/a, n/a, n/a, n/a, n/a, n/a, n/a, n/a, n/a, n/a, n/a, n/a, n/a, n/a, n/a, n/a, n/a, n/a, n/a

F219: Blank, n/a, n/a, n/a, n/a, n/a, n/a, n/a, n/a, n/a, n/a, n/a, n/a, n/a, n/a, n/a, n/a, n/a, n/a, n/a, n/a, n/a, n/a, n/a, n/a

F228: QualityControl, n/a, n/a, n/a, n/a, n/a, n/a, n/a, n/a, n/a, n/a, n/a, n/a, n/a, n/a, n/a, n/a, n/a, n/a, n/a, n/a, n/a, n/a, n/a, n/a

F229: Blank, n/a, n/a, n/a, n/a, n/a, n/a, n/a, n/a, n/a, n/a, n/a, n/a, n/a, n/a, n/a, n/a, n/a, n/a, n/a, n/a, n/a, n/a, n/a, n/a

F239: QualityControl, n/a, n/a, n/a, n/a, n/a, n/a, n/a, n/a, n/a, n/a, n/a, n/a, n/a, n/a, n/a, n/a, n/a, n/a, n/a, n/a, n/a, n/a, n/a, n/a

F240: Blank, n/a, n/a, n/a, n/a, n/a, n/a, n/a, n/a, n/a, n/a, n/a, n/a, n/a, n/a, n/a, n/a, n/a, n/a, n/a, n/a, n/a, n/a, n/a, n/a

F243: Blank, n/a, n/a, n/a, n/a, n/a, n/a, n/a, n/a, n/a, n/a, n/a, n/a, n/a, n/a, n/a, n/a, n/a, n/a, n/a, n/a, n/a, n/a, n/a, n/a

F249: QualityControl, n/a, n/a, n/a, n/a, n/a, n/a, n/a, n/a, n/a, n/a, n/a, n/a, n/a, n/a, n/a, n/a, n/a, n/a, n/a, n/a, n/a, n/a, n/a, n/a

F250: Blank, n/a, n/a, n/a, n/a, n/a, n/a, n/a, n/a, n/a, n/a, n/a, n/a, n/a, n/a, n/a, n/a, n/a, n/a, n/a, n/a, n/a, n/a, n/a, n/a

F255: QualityControl, n/a, n/a, n/a, n/a, n/a, n/a, n/a, n/a, n/a, n/a, n/a, n/a, n/a, n/a, n/a, n/a, n/a, n/a, n/a, n/a, n/a, n/a, n/a, n/a

F256: IdentificationOnly, n/a, n/a, n/a, n/a, n/a, n/a, n/a, n/a, n/a, n/a, n/a, n/a, n/a, n/a, n/a, n/a, n/a, n/a, n/a, n/a, n/a, n/a, n/a, n/a

F257: Blank, n/a, n/a, n/a, n/a, n/a, n/a, n/a, n/a, n/a, n/a, n/a, n/a, n/a, n/a, n/a, n/a, n/a, n/a, n/a, n/a, n/a, n/a, n/a, n/a

F258: IdentificationOnly, n/a, n/a, n/a, n/a, n/a, n/a, n/a, n/a, n/a, n/a, n/a, n/a, n/a, n/a, n/a, n/a, n/a, n/a, n/a, n/a, n/a, n/a, n/a, n/a

F261: IdentificationOnly, n/a, n/a, n/a, n/a, n/a, n/a, n/a, n/a, n/a, n/a, n/a, n/a, n/a, n/a, n/a, n/a, n/a, n/a, n/a, n/a, n/a, n/a, n/a, n/a

F264: IdentificationOnly, n/a, n/a, n/a, n/a, n/a, n/a, n/a, n/a, n/a, n/a, n/a, n/a, n/a, n/a, n/a, n/a, n/a, n/a, n/a, n/a, n/a, n/a, n/a, n/a

F267: IdentificationOnly, n/a, n/a, n/a, n/a, n/a, n/a, n/a, n/a, n/a, n/a, n/a, n/a, n/a, n/a, n/a, n/a, n/a, n/a, n/a, n/a, n/a, n/a, n/a, n/a

F271: IdentificationOnly, n/a, n/a, n/a, n/a, n/a, n/a, n/a, n/a, n/a, n/a, n/a, n/a, n/a, n/a, n/a, n/a, n/a, n/a, n/a, n/a, n/a, n/a, n/a, n/a

F274: IdentificationOnly, n/a, n/a, n/a, n/a, n/a, n/a, n/a, n/a, n/a, n/a, n/a, n/a, n/a, n/a, n/a, n/a, n/a, n/a, n/a, n/a, n/a, n/a, n/a, n/a

F277: IdentificationOnly, n/a, n/a, n/a, n/a, n/a, n/a, n/a, n/a, n/a, n/a, n/a, n/a, n/a, n/a, n/a, n/a, n/a, n/a, n/a, n/a, n/a, n/a, n/a, n/a

F280: IdentificationOnly, n/a, n/a, n/a, n/a, n/a, n/a, n/a, n/a, n/a, n/a, n/a, n/a, n/a, n/a, n/a, n/a, n/a, n/a, n/a, n/a, n/a, n/a, n/a, n/a

F284: Blank, n/a, n/a, n/a, n/a, n/a, n/a, n/a, n/a, n/a, n/a, n/a, n/a, n/a, n/a, n/a, n/a, n/a, n/a, n/a, n/a, n/a, n/a, n/a, n/a

F285: Blank, n/a, n/a, n/a, n/a, n/a, n/a, n/a, n/a, n/a, n/a, n/a, n/a, n/a, n/a, n/a, n/a, n/a, n/a, n/a, n/a, n/a, n/a, n/a, n/a

F286: Blank, n/a, n/a, n/a, n/a, n/a, n/a, n/a, n/a, n/a, n/a, n/a, n/a, n/a, n/a, n/a, n/a, n/a, n/a, n/a, n/a, n/a, n/a, n/a, n/a

F288: Blank, n/a, n/a, n/a, n/a, n/a, n/a, n/a, n/a, n/a, n/a, n/a, n/a, n/a, n/a, n/a, n/a, n/a, n/a, n/a, n/a, n/a, n/a, n/a, n/a

F289: QualityControl, n/a, n/a, n/a, n/a, n/a, n/a, n/a, n/a, n/a, n/a, n/a, n/a, n/a, n/a, n/a, n/a, n/a, n/a, n/a, n/a, n/a, n/a, n/a, n/a

F290: Blank, n/a, n/a, n/a, n/a, n/a, n/a, n/a, n/a, n/a, n/a, n/a, n/a, n/a, n/a, n/a, n/a, n/a, n/a, n/a, n/a, n/a, n/a, n/a, n/a

F299: QualityControl, n/a, n/a, n/a, n/a, n/a, n/a, n/a, n/a, n/a, n/a, n/a, n/a, n/a, n/a, n/a, n/a, n/a, n/a, n/a, n/a, n/a, n/a, n/a, n/a

F300: Blank, n/a, n/a, n/a, n/a, n/a, n/a, n/a, n/a, n/a, n/a, n/a, n/a, n/a, n/a, n/a, n/a, n/a, n/a, n/a, n/a, n/a, n/a, n/a, n/a

F311: QualityControl, n/a, n/a, n/a, n/a, n/a, n/a, n/a, n/a, n/a, n/a, n/a, n/a, n/a, n/a, n/a, n/a, n/a, n/a, n/a, n/a, n/a, n/a, n/a, n/a

F312: Blank, n/a, n/a, n/a, n/a, n/a, n/a, n/a, n/a, n/a, n/a, n/a, n/a, n/a, n/a, n/a, n/a, n/a, n/a, n/a, n/a, n/a, n/a, n/a, n/a

F322: QualityControl, n/a, n/a, n/a, n/a, n/a, n/a, n/a, n/a, n/a, n/a, n/a, n/a, n/a, n/a, n/a, n/a, n/a, n/a, n/a, n/a, n/a, n/a, n/a, n/a

F323: Blank, n/a, n/a, n/a, n/a, n/a, n/a, n/a, n/a, n/a, n/a, n/a, n/a, n/a, n/a, n/a, n/a, n/a, n/a, n/a, n/a, n/a, n/a, n/a, n/a

F333: QualityControl, n/a, n/a, n/a, n/a, n/a, n/a, n/a, n/a, n/a, n/a, n/a, n/a, n/a, n/a, n/a, n/a, n/a, n/a, n/a, n/a, n/a, n/a, n/a, n/a

F334: Blank, n/a, n/a, n/a, n/a, n/a, n/a, n/a, n/a, n/a, n/a, n/a, n/a, n/a, n/a, n/a, n/a, n/a, n/a, n/a, n/a, n/a, n/a, n/a, n/a

F343: QualityControl, n/a, n/a, n/a, n/a, n/a, n/a, n/a, n/a, n/a, n/a, n/a, n/a, n/a, n/a, n/a, n/a, n/a, n/a, n/a, n/a, n/a, n/a, n/a, n/a

F344: Blank, n/a, n/a, n/a, n/a, n/a, n/a, n/a, n/a, n/a, n/a, n/a, n/a, n/a, n/a, n/a, n/a, n/a, n/a, n/a, n/a, n/a, n/a, n/a, n/a

F353: QualityControl, n/a, n/a, n/a, n/a, n/a, n/a, n/a, n/a, n/a, n/a, n/a, n/a, n/a, n/a, n/a, n/a, n/a, n/a, n/a, n/a, n/a, n/a, n/a, n/a

F354: Blank, n/a, n/a, n/a, n/a, n/a, n/a, n/a, n/a, n/a, n/a, n/a, n/a, n/a, n/a, n/a, n/a, n/a, n/a, n/a, n/a, n/a, n/a, n/a, n/a

F364: QualityControl, n/a, n/a, n/a, n/a, n/a, n/a, n/a, n/a, n/a, n/a, n/a, n/a, n/a, n/a, n/a, n/a, n/a, n/a, n/a, n/a, n/a, n/a, n/a, n/a

F365: Blank, n/a, n/a, n/a, n/a, n/a, n/a, n/a, n/a, n/a, n/a, n/a, n/a, n/a, n/a, n/a, n/a, n/a, n/a, n/a, n/a, n/a, n/a, n/a, n/a

F368: Blank, n/a, n/a, n/a, n/a, n/a, n/a, n/a, n/a, n/a, n/a, n/a, n/a, n/a, n/a, n/a, n/a, n/a, n/a, n/a, n/a, n/a, n/a, n/a, n/a

F374: QualityControl, n/a, n/a, n/a, n/a, n/a, n/a, n/a, n/a, n/a, n/a, n/a, n/a, n/a, n/a, n/a, n/a, n/a, n/a, n/a, n/a, n/a, n/a, n/a, n/a

F375: Blank, n/a, n/a, n/a, n/a, n/a, n/a, n/a, n/a, n/a, n/a, n/a, n/a, n/a, n/a, n/a, n/a, n/a, n/a, n/a, n/a, n/a, n/a, n/a, n/a

F380: QualityControl, n/a, n/a, n/a, n/a, n/a, n/a, n/a, n/a, n/a, n/a, n/a, n/a, n/a, n/a, n/a, n/a, n/a, n/a, n/a, n/a, n/a, n/a, n/a, n/a

F381: IdentificationOnly, n/a, n/a, n/a, n/a, n/a, n/a, n/a, n/a, n/a, n/a, n/a, n/a, n/a, n/a, n/a, n/a, n/a, n/a, n/a, n/a, n/a, n/a, n/a, n/a

F382: Blank, n/a, n/a, n/a, n/a, n/a, n/a, n/a, n/a, n/a, n/a, n/a, n/a, n/a, n/a, n/a, n/a, n/a, n/a, n/a, n/a, n/a, n/a, n/a, n/a

F383: IdentificationOnly, n/a, n/a, n/a, n/a, n/a, n/a, n/a, n/a, n/a, n/a, n/a, n/a, n/a, n/a, n/a, n/a, n/a, n/a, n/a, n/a, n/a, n/a, n/a, n/a

F386: IdentificationOnly, n/a, n/a, n/a, n/a, n/a, n/a, n/a, n/a, n/a, n/a, n/a, n/a, n/a, n/a, n/a, n/a, n/a, n/a, n/a, n/a, n/a, n/a, n/a, n/a

F389: IdentificationOnly, n/a, n/a, n/a, n/a, n/a, n/a, n/a, n/a, n/a, n/a, n/a, n/a, n/a, n/a, n/a, n/a, n/a, n/a, n/a, n/a, n/a, n/a, n/a, n/a

F392: IdentificationOnly, n/a, n/a, n/a, n/a, n/a, n/a, n/a, n/a, n/a, n/a, n/a, n/a, n/a, n/a, n/a, n/a, n/a, n/a, n/a, n/a, n/a, n/a, n/a, n/a

F395: Blank, n/a, n/a, n/a, n/a, n/a, n/a, n/a, n/a, n/a, n/a, n/a, n/a, n/a, n/a, n/a, n/a, n/a, n/a, n/a, n/a, n/a, n/a, n/a, n/a

F396: IdentificationOnly, n/a, n/a, n/a, n/a, n/a, n/a, n/a, n/a, n/a, n/a, n/a, n/a, n/a, n/a, n/a, n/a, n/a, n/a, n/a, n/a, n/a, n/a, n/a, n/a

F399: IdentificationOnly, n/a, n/a, n/a, n/a, n/a, n/a, n/a, n/a, n/a, n/a, n/a, n/a, n/a, n/a, n/a, n/a, n/a, n/a, n/a, n/a, n/a, n/a, n/a, n/a

F402: IdentificationOnly, n/a, n/a, n/a, n/a, n/a, n/a, n/a, n/a, n/a, n/a, n/a, n/a, n/a, n/a, n/a, n/a, n/a, n/a, n/a, n/a, n/a, n/a, n/a, n/a

F405: IdentificationOnly, n/a, n/a, n/a, n/a, n/a, n/a, n/a, n/a, n/a, n/a, n/a, n/a, n/a, n/a, n/a, n/a, n/a, n/a, n/a, n/a, n/a, n/a, n/a, n/a

ST, Brown

F110: Sample, 2, F2, Gelber, P47-1, Brown, ST, brown, brown, brown, brown, Brown, No, Yes, No, No, No, No, Brown, Brown, Brown, Absent, Brown, Brown, Brown

F112: Sample, 4, F2, Gelber, P47-1, Brown, ST, brown, brown, brown, brown, Brown, No, Yes, No, No, No, No, Brown, Brown, Brown, Absent, Brown, Brown, Brown

F114: Sample, 36, F2, P47-1, Disco/2, Brown, ST, brown, brown, brown, brown, Brown, No, Yes, No, No, No, No, Brown, Brown, Brown, Absent, Brown, Brown, Brown

F118: Sample, 2, F2, Gelber, P47-1, Brown, ST, brown, brown, brown, brown, Brown, No, Yes, No, No, No, No, Brown, Brown, Brown, Absent, Brown, Brown, Brown

F211: Sample, 2, F2, Gelber, P47-1, Brown, ST, brown, brown, brown, brown, Brown, No, Yes, No, No, No, No, Brown, Brown, Brown, Absent, Brown, Brown, Brown

F213: Sample, 4, F2, Gelber, P47-1, Brown, ST, brown, brown, brown, brown, Brown, No, Yes, No, No, No, No, Brown, Brown, Brown, Absent, Brown, Brown, Brown

F217: Sample, 36, F2, P47-1, Disco/2, Brown, ST, brown, brown, brown, brown, Brown, No, Yes, No, No, No, No, Brown, Brown, Brown, Absent, Brown, Brown, Brown

F336: Sample, 2, F2, Gelber, P47-1, Brown, ST, brown, brown, brown, brown, Brown, No, Yes, No, No, No, No, Brown, Brown, Brown, Absent, Brown, Brown, Brown

F338: Sample, 4, F2, Gelber, P47-1, Brown, ST, brown, brown, brown, brown, Brown, No, Yes, No, No, No, No, Brown, Brown, Brown, Absent, Brown, Brown, Brown

F342: Sample, 36, F2, P47-1, Disco/2, Brown, ST, brown, brown, brown, brown, Brown, No, Yes, No, No, No, No, Brown, Brown, Brown, Absent, Brown, Brown, Brown

ST, BrownPink-PinkBrown

F91: Sample, 8, F2, Gelber, P47-1, Brown mixed, ST, brown, red, pink, brown, RedBrown, No, Yes, No, No, No, Yes, BrownPink, BrownPink, Pink, Brown, Brown, PinkBrown, BrownPink-PinkBrown

F95: Sample, 10, F2, Gelber, P47-1, Brown mixed, ST, brown, red, pink, brown, RedBrown, No, Yes, No, No, No, Yes, BrownPink, BrownPink, Pink, Brown, Brown, PinkBrown, BrownPink-PinkBrown

F97: Sample, 12, F2, Snowdrop, P47-1, Brown mixed, ST, brown, red, pink, brown, RedBrown, No, Yes, No, No, No, Yes, BrownPink, BrownPink, Pink, Brown, Brown, PinkBrown, BrownPink-PinkBrown

F99: Sample, 32, F2, P47-1, Disco/2, Brown mixed, ST, brown, red, pink, brown, RedBrown, No, Yes, No, No, No, Yes, BrownPink, BrownPink, Pink, Brown, Brown, PinkBrown, BrownPink-PinkBrown

F105: Sample, 64, F2, Aurora, P47-1, Brown mixed, ST, pink, red, pink, red, Red, No, No, No, No, No, Yes, BrownPink, BrownPink, Pink, Absent, Pink, Pink, BrownPink-PinkBrown

F230: Sample, 8, F2, Gelber, P47-1, Brown mixed, ST, brown, red, pink, brown, RedBrown, No, Yes, No, No, No, Yes, BrownPink, BrownPink, Pink, Brown, Brown, PinkBrown, BrownPink-PinkBrown

F231: Sample, 10, F2, Gelber, P47-1, Brown mixed, ST, brown, red, pink, brown, RedBrown, No, Yes, No, No, No, Yes, BrownPink, BrownPink, Pink, Brown, Brown, PinkBrown, BrownPink-PinkBrown

F232: Sample, 12, F2, Snowdrop, P47-1, Brown mixed, ST, brown, red, pink, brown, RedBrown, No, Yes, No, No, No, Yes, BrownPink, BrownPink, Pink, Brown, Brown, PinkBrown, BrownPink-PinkBrown

F238: Sample, 32, F2, P47-1, Disco/2, Brown mixed, ST, brown, red, pink, brown, RedBrown, No, Yes, No, No, No, Yes, BrownPink, BrownPink, Pink, Brown, Brown, PinkBrown, BrownPink-PinkBrown

F248: Sample, 64, F2, Aurora, P47-1, Brown mixed, ST, pink, red, pink, red, Red, No, No, No, No, No, Yes, BrownPink, BrownPink, Pink, Absent, Pink, Pink, BrownPink-PinkBrown

F355: Sample, 8, F2, Gelber, P47-1, Brown mixed, ST, brown, red, pink, brown, RedBrown, No, Yes, No, No, No, Yes, BrownPink, BrownPink, Pink, Brown, Brown, PinkBrown, BrownPink-PinkBrown

F356: Sample, 10, F2, Gelber, P47-1, Brown mixed, ST, brown, red, pink, brown, RedBrown, No, Yes, No, No, No, Yes, BrownPink, BrownPink, Pink, Brown, Brown, PinkBrown, BrownPink-PinkBrown

F357: Sample, 12, F2, Snowdrop, P47-1, Brown mixed, ST, brown, red, pink, brown, RedBrown, No, Yes, No, No, No, Yes, BrownPink, BrownPink, Pink, Brown, Brown, PinkBrown, BrownPink-PinkBrown

F363: Sample, 32, F2, P47-1, Disco/2, Brown mixed, ST, brown, red, pink, brown, RedBrown, No, Yes, No, No, No, Yes, BrownPink, BrownPink, Pink, Brown, Brown, PinkBrown, BrownPink-PinkBrown

F373: Sample, 64, F2, Aurora, P47-1, Brown mixed, ST, pink, red, pink, red, Red, No, No, No, No, No, Yes, BrownPink, BrownPink, Pink, Absent, Pink, Pink, BrownPink-PinkBrown

ST, BrownRed-RedBrown

F72: Sample, 18, F2, Snowdrop, P47-1, Red, ST, brown, red, red, brown, RedBrown, Yes, Yes, No, No, No, No, BrownRed, BrownRed, Red, Brown, Brown, RedBrown, BrownRed-RedBrown

F89: Sample, 5, F2, Gelber, P47-1, Brown mixed, ST, red, brown, brown, red, RedBrown, Yes, Yes, No, No, No, No, BrownRed, BrownRed, Brown, Red, Red, BrownRed, BrownRed-RedBrown

F122: Sample, 34, F2, P47-1, Disco/2, Red, ST, brown, red, red, brown, RedBrown, Yes, Yes, No, No, No, No, BrownRed, BrownRed, Red, Brown, Brown, RedBrown, BrownRed-RedBrown

F220: Sample, 5, F2, Gelber, P47-1, Brown mixed, ST, red, brown, brown, red, RedBrown, Yes, Yes, No, No, No, No, BrownRed, BrownRed, Brown, Red, Red, BrownRed, BrownRed-RedBrown

F234: Sample, 18, F2, Snowdrop, P47-1, Red, ST, brown, red, red, brown, RedBrown, Yes, Yes, No, No, No, No, BrownRed, BrownRed, Red, Brown, Brown, RedBrown, BrownRed-RedBrown

F241: Sample, 34, F2, P47-1, Disco/2, Red, ST, brown, red, red, brown, RedBrown, Yes, Yes, No, No, No, No, BrownRed, BrownRed, Red, Brown, Brown, RedBrown, BrownRed-RedBrown

F345: Sample, 5, F2, Gelber, P47-1, Brown mixed, ST, red, brown, brown, red, RedBrown, Yes, Yes, No, No, No, No, BrownRed, BrownRed, Brown, Red, Red, BrownRed, BrownRed-RedBrown

F359: Sample, 18, F2, Snowdrop, P47-1, Red, ST, brown, red, red, brown, RedBrown, Yes, Yes, No, No, No, No, BrownRed, BrownRed, Red, Brown, Brown, RedBrown, BrownRed-RedBrown

F366: Sample, 34, F2, P47-1, Disco/2, Red, ST, brown, red, red, brown, RedBrown, Yes, Yes, No, No, No, No, BrownRed, BrownRed, Red, Brown, Brown, RedBrown, BrownRed-RedBrown

ST, Purplish red

F82: Sample, 71, 2263, Rinrei, P47-1, Red, ST, purple, red, red, purple, RedPurple, Yes, No, No, Yes, No, No, RedPurple, RedPurple, Purplish red, Absent, Purplish red, Purplish red, Purplish red

F87: Sample, 76, 71B, P47-1, NV153, Red, ST, purple, red, red, purple, RedPurple, Yes, No, No, Yes, No, No, RedPurple, RedPurple, Purplish red, Absent, Purplish red, Purplish red, Purplish red

F251: Sample, 71, 2263, Rinrei, P47-1, Red, ST, purple, red, red, purple, RedPurple, Yes, No, No, Yes, No, No, RedPurple, RedPurple, Purplish red, Absent, Purplish red, Purplish red, Purplish red

F254: Sample, 76, 71B, P47-1, NV153, Red, ST, purple, red, red, purple, RedPurple, Yes, No, No, Yes, No, No, RedPurple, RedPurple, Purplish red, Absent, Purplish red, Purplish red, Purplish red

F376: Sample, 71, 2263, Rinrei, P47-1, Red, ST, purple, red, red, purple, RedPurple, Yes, No, No, Yes, No, No, RedPurple, RedPurple, Purplish red, Absent, Purplish red, Purplish red, Purplish red

F379: Sample, 76, 71B, P47-1, NV153, Red, ST, purple, red, red, purple, RedPurple, Yes, No, No, Yes, No, No, RedPurple, RedPurple, Purplish red, Absent, Purplish red, Purplish red, Purplish red

ST, Red

F67: Sample, 13, F2, Snowdrop, P47-1, Red, ST, red, red, red, red, Red, Yes, No, No, No, No, No, Red, BrownRed, Red, Absent, Red, Red, Red

F78: Sample, 22, F2, Snowdrop, P47-1, Red, ST, red, red, red, red, Red, Yes, No, No, No, No, No, Red, BrownRed, Red, Absent, Red, Red, Red

F81: Sample, 62, F2, Aurora, P47-1, Red, ST, red, red, red, red, Red, Yes, No, No, No, No, No, Red, BrownRed, Red, Absent, Red, Red, Red

F244: Sample, 13, F2, Snowdrop, P47-1, Red, ST, red, red, red, red, Red, Yes, No, No, No, No, No, Red, BrownRed, Red, Absent, Red, Red, Red

F246: Sample, 22, F2, Snowdrop, P47-1, Red, ST, red, red, red, red, Red, Yes, No, No, No, No, No, Red, BrownRed, Red, Absent, Red, Red, Red

F247: Sample, 62, F2, Aurora, P47-1, Red, ST, red, red, red, red, Red, Yes, No, No, No, No, No, Red, BrownRed, Red, Absent, Red, Red, Red

F369: Sample, 13, F2, Snowdrop, P47-1, Red, ST, red, red, red, red, Red, Yes, No, No, No, No, No, Red, BrownRed, Red, Absent, Red, Red, Red

F371: Sample, 22, F2, Snowdrop, P47-1, Red, ST, red, red, red, red, Red, Yes, No, No, No, No, No, Red, BrownRed, Red, Absent, Red, Red, Red

F372: Sample, 62, F2, Aurora, P47-1, Red, ST, red, red, red, red, Red, Yes, No, No, No, No, No, Red, BrownRed, Red, Absent, Red, Red, Red

ST, White

F16: Sample, 28, F2, P47-1, Snowdrop, White, ST, white, white, white, white, White, No, No, No, No, Yes, No, White, White, White, Absent, White, White, White

F23: Sample, 46, F2, Snowdrop, Gelber, White, ST, white, white, white, white, White, No, No, No, No, Yes, No, White, White, White, Absent, White, White, White

F26: Sample, 54, F2, Disco/2, Gelber, White, ST, white, white, white, white, White, No, No, No, No, Yes, No, White, White, White, Absent, White, White, White

F168: Sample, 28, F2, P47-1, Snowdrop, White, ST, white, white, white, white, White, No, No, No, No, Yes, No, White, White, White, Absent, White, White, White

F173: Sample, 46, F2, Snowdrop, Gelber, White, ST, white, white, white, white, White, No, No, No, No, Yes, No, White, White, White, Absent, White, White, White

F178: Sample, 54, F2, Disco/2, Gelber, White, ST, white, white, white, white, White, No, No, No, No, Yes, No, White, White, White, Absent, White, White, White

F293: Sample, 28, F2, P47-1, Snowdrop, White, ST, white, white, white, white, White, No, No, No, No, Yes, No, White, White, White, Absent, White, White, White

F298: Sample, 46, F2, Snowdrop, Gelber, White, ST, white, white, white, white, White, No, No, No, No, Yes, No, White, White, White, Absent, White, White, White

F303: Sample, 54, F2, Disco/2, Gelber, White, ST, white, white, white, white, White, No, No, No, No, Yes, No, White, White, White, Absent, White, White, White

ST, WhiteBrown

F34: Sample, 24, F2, Gelber, P47-1, White with patterns, ST, brown, white, white, brown, WhiteBrown, No, Yes, No, No, Yes, No, WhiteBrown, WhiteBrown, White, Brown, Brown, WhiteBrown, WhiteBrown

F40: Sample, 38, F2, P47-1, Disco/2, White with patterns, ST, brown, white, white, brown, WhiteBrown, No, Yes, No, No, Yes, No, WhiteBrown, WhiteBrown, White, Brown, Brown, WhiteBrown, WhiteBrown

F42: Sample, 40, F2, P47-1, Disco/2, White with patterns, ST, brown, white, white, brown, WhiteBrown, No, Yes, No, No, Yes, No, WhiteBrown, WhiteBrown, White, Brown, Brown, WhiteBrown, WhiteBrown

F46: Sample, 48, F2, Snowdrop, Gelber, White with patterns, ST, brown, white, white, brown, WhiteBrown, No, Yes, No, No, Yes, No, WhiteBrown, WhiteBrown, White, Brown, Brown, WhiteBrown, WhiteBrown

F51: Sample, 56, F2, Disco/2, Gelber, White with patterns, ST, brown, white, white, brown, WhiteBrown, No, Yes, No, No, Yes, No, WhiteBrown, WhiteBrown, White, Brown, Brown, WhiteBrown, WhiteBrown

F55: Sample, 58, F2, Aurora, Gelber, White with patterns, ST, brown, white, white, brown, WhiteBrown, No, Yes, No, No, Yes, No, WhiteBrown, WhiteBrown, White, Brown, Brown, WhiteBrown, WhiteBrown

F59: Sample, 66, F2, Aurora, P47-1, White with patterns, ST, brown, white, white, brown, WhiteBrown, No, Yes, No, No, Yes, No, WhiteBrown, WhiteBrown, White, Brown, Brown, WhiteBrown, WhiteBrown

F189: Sample, 24, F2, Gelber, P47-1, White with patterns, ST, brown, white, white, brown, WhiteBrown, No, Yes, No, No, Yes, No, WhiteBrown, WhiteBrown, White, Brown, Brown, WhiteBrown, WhiteBrown

F192: Sample, 38, F2, P47-1, Disco/2, White with patterns, ST, brown, white, white, brown, WhiteBrown, No, Yes, No, No, Yes, No, WhiteBrown, WhiteBrown, White, Brown, Brown, WhiteBrown, WhiteBrown

F194: Sample, 40, F2, P47-1, Disco/2, White with patterns, ST, brown, white, white, brown, WhiteBrown, No, Yes, No, No, Yes, No, WhiteBrown, WhiteBrown, White, Brown, Brown, WhiteBrown, WhiteBrown

F196: Sample, 48, F2, Snowdrop, Gelber, White with patterns, ST, brown, white, white, brown, WhiteBrown, No, Yes, No, No, Yes, No, WhiteBrown, WhiteBrown, White, Brown, Brown, WhiteBrown, WhiteBrown

F200: Sample, 56, F2, Disco/2, Gelber, White with patterns, ST, brown, white, white, brown, WhiteBrown, No, Yes, No, No, Yes, No, WhiteBrown, WhiteBrown, White, Brown, Brown, WhiteBrown, WhiteBrown

F202: Sample, 58, F2, Aurora, Gelber, White with patterns, ST, brown, white, white, brown, WhiteBrown, No, Yes, No, No, Yes, No, WhiteBrown, WhiteBrown, White, Brown, Brown, WhiteBrown, WhiteBrown

F204: Sample, 66, F2, Aurora, P47-1, White with patterns, ST, brown, white, white, brown, WhiteBrown, No, Yes, No, No, Yes, No, WhiteBrown, WhiteBrown, White, Brown, Brown, WhiteBrown, WhiteBrown

F314: Sample, 24, F2, Gelber, P47-1, White with patterns, ST, brown, white, white, brown, WhiteBrown, No, Yes, No, No, Yes, No, WhiteBrown, WhiteBrown, White, Brown, Brown, WhiteBrown, WhiteBrown

F317: Sample, 38, F2, P47-1, Disco/2, White with patterns, ST, brown, white, white, brown, WhiteBrown, No, Yes, No, No, Yes, No, WhiteBrown, WhiteBrown, White, Brown, Brown, WhiteBrown, WhiteBrown

F319: Sample, 40, F2, P47-1, Disco/2, White with patterns, ST, brown, white, white, brown, WhiteBrown, No, Yes, No, No, Yes, No, WhiteBrown, WhiteBrown, White, Brown, Brown, WhiteBrown, WhiteBrown

F321: Sample, 48, F2, Snowdrop, Gelber, White with patterns, ST, brown, white, white, brown, WhiteBrown, No, Yes, No, No, Yes, No, WhiteBrown, WhiteBrown, White, Brown, Brown, WhiteBrown, WhiteBrown

F325: Sample, 56, F2, Disco/2, Gelber, White with patterns, ST, brown, white, white, brown, WhiteBrown, No, Yes, No, No, Yes, No, WhiteBrown, WhiteBrown, White, Brown, Brown, WhiteBrown, WhiteBrown

F327: Sample, 58, F2, Aurora, Gelber, White with patterns, ST, brown, white, white, brown, WhiteBrown, No, Yes, No, No, Yes, No, WhiteBrown, WhiteBrown, White, Brown, Brown, WhiteBrown, WhiteBrown

F329: Sample, 66, F2, Aurora, P47-1, White with patterns, ST, brown, white, white, brown, WhiteBrown, No, Yes, No, No, Yes, No, WhiteBrown, WhiteBrown, White, Brown, Brown, WhiteBrown, WhiteBrown

ST, WhitePink

F14: Sample, 26, F2, Gelber, P47-1, White, ST, pink, white, white, white, White, No, No, No, No, Yes, Yes, White, WhiteYellow, White, Pink, Pink, WhitePink, WhitePink

F17: Sample, 30, F2, P47-1, Snowdrop, White, ST, pink, white, white, white, White, No, No, No, No, Yes, Yes, White, WhiteBrown, White, Pink, Pink, WhitePink, WhitePink

F24: Sample, 52, F2, Disco/2, Gelber, White, ST, pink, white, white, white, White, No, No, No, No, Yes, Yes, White, WhiteYellow, White, Pink, Pink, WhitePink, WhitePink

F166: Sample, 26, F2, Gelber, P47-1, White, ST, pink, white, white, white, White, No, No, No, No, Yes, Yes, White, WhiteYellow, White, Pink, Pink, WhitePink, WhitePink

F169: Sample, 30, F2, P47-1, Snowdrop, White, ST, pink, white, white, white, White, No, No, No, No, Yes, Yes, White, WhiteBrown, White, Pink, Pink, WhitePink, WhitePink

F176: Sample, 52, F2, Disco/2, Gelber, White, ST, pink, white, white, white, White, No, No, No, No, Yes, Yes, White, WhiteYellow, White, Pink, Pink, WhitePink, WhitePink

F291: Sample, 26, F2, Gelber, P47-1, White, ST, pink, white, white, white, White, No, No, No, No, Yes, Yes, White, WhiteYellow, White, Pink, Pink, WhitePink, WhitePink

F294: Sample, 30, F2, P47-1, Snowdrop, White, ST, pink, white, white, white, White, No, No, No, No, Yes, Yes, White, WhiteBrown, White, Pink, Pink, WhitePink, WhitePink

F301: Sample, 52, F2, Disco/2, Gelber, White, ST, pink, white, white, white, White, No, No, No, No, Yes, Yes, White, WhiteYellow, White, Pink, Pink, WhitePink, WhitePink

ST, WhiteYellow

F57: Sample, 60, F2, Aurora, Gelber, White with patterns, ST, yellow, white, white, brown, WhiteYellow, No, No, Yes, No, Yes, No, WhiteYellow, WhiteYellow, White, Yellow, Yellow, WhiteYellow, WhiteYellow

F185: Sample, 60, F2, Aurora, Gelber, White with patterns, ST, yellow, white, white, brown, WhiteYellow, No, No, Yes, No, Yes, No, WhiteYellow, WhiteYellow, White, Yellow, Yellow, WhiteYellow, WhiteYellow

F310: Sample, 60, F2, Aurora, Gelber, White with patterns, ST, yellow, white, white, brown, WhiteYellow, No, No, Yes, No, Yes, No, WhiteYellow, WhiteYellow, White, Yellow, Yellow, WhiteYellow, WhiteYellow

W, Brown

F103: Sample, 35, F2, P47-1, Disco/2, Brown mixed, W, brown, brown, brown, red, BrownPurple, No, Yes, No, Yes, No, No, Brown, Brown, Brown, Absent, Brown, Brown, Brown

F109: Sample, 1, F2, Gelber, P47-1, Brown, W, brown, brown, brown, brown, Brown, No, Yes, No, No, No, No, Brown, Brown, Brown, Absent, Brown, Brown, Brown

F111: Sample, 3, F2, Gelber, P47-1, Brown, W, brown, brown, brown, brown, Brown, No, Yes, No, No, No, No, Brown, Brown, Brown, Absent, Brown, Brown, Brown

F113: Sample, 6, F2, Gelber, P47-1, Brown, W, brown, brown, brown, brown, Brown, No, Yes, No, No, No, No, Brown, BrownRed, Brown, Absent, Brown, Brown, Brown

F119: Sample, 3, F2, Gelber, P47-1, Brown, W, brown, brown, brown, brown, Brown, No, Yes, No, No, No, No, Brown, Brown, Brown, Absent, Brown, Brown, Brown

F210: Sample, 1, F2, Gelber, P47-1, Brown, W, brown, brown, brown, brown, Brown, No, Yes, No, No, No, No, Brown, Brown, Brown, Absent, Brown, Brown, Brown

F212: Sample, 3, F2, Gelber, P47-1, Brown, W, brown, brown, brown, brown, Brown, No, Yes, No, No, No, No, Brown, Brown, Brown, Absent, Brown, Brown, Brown

F214: Sample, 6, F2, Gelber, P47-1, Brown, W, brown, brown, brown, brown, Brown, No, Yes, No, No, No, No, Brown, BrownRed, Brown, Absent, Brown, Brown, Brown

F226: Sample, 35, F2, P47-1, Disco/2, Brown mixed, W, brown, brown, brown, red, BrownPurple, No, Yes, No, Yes, No, No, Brown, Brown, Brown, Absent, Brown, Brown, Brown

F335: Sample, 1, F2, Gelber, P47-1, Brown, W, brown, brown, brown, brown, Brown, No, Yes, No, No, No, No, Brown, Brown, Brown, Absent, Brown, Brown, Brown

F337: Sample, 3, F2, Gelber, P47-1, Brown, W, brown, brown, brown, brown, Brown, No, Yes, No, No, No, No, Brown, Brown, Brown, Absent, Brown, Brown, Brown

F339: Sample, 6, F2, Gelber, P47-1, Brown, W, brown, brown, brown, brown, Brown, No, Yes, No, No, No, No, Brown, BrownRed, Brown, Absent, Brown, Brown, Brown

F351: Sample, 35, F2, P47-1, Disco/2, Brown mixed, W, brown, brown, brown, red, BrownPurple, No, Yes, No, Yes, No, No, Brown, Brown, Brown, Absent, Brown, Brown, Brown

W, BrownPink-PinkBrown

F90: Sample, 7, F2, Gelber, P47-1, Brown mixed, W, pink, brown, brown, red, RedBrown, No, Yes, No, No, No, Yes, BrownPink, BrownPink, Brown, Pink, Pink, BrownPink, BrownPink-PinkBrown

F94: Sample, 9, F2, Gelber, P47-1, Brown mixed, W, pink, brown, brown, red, RedBrown, No, Yes, No, No, No, Yes, BrownPink, BrownPink, Brown, Pink, Pink, BrownPink, BrownPink-PinkBrown

F96: Sample, 11, F2, Snowdrop, P47-1, Brown mixed, W, pink, brown, brown, red, RedBrown, No, Yes, No, No, No, Yes, BrownPink, BrownPink, Brown, Pink, Pink, BrownPink, BrownPink-PinkBrown

F98: Sample, 31, F2, P47-1, Disco/2, Brown mixed, W, brown, red, pink, brown, RedBrown, No, Yes, No, No, No, Yes, BrownPink, BrownPink, Pink, Brown, Brown, PinkBrown, BrownPink-PinkBrown

F104: Sample, 63, F2, Aurora, P47-1, Brown mixed, W, pink, brown, brown, red, RedBrown, No, Yes, No, No, No, Yes, BrownPink, BrownPink, Brown, Pink, Pink, BrownPink, BrownPink-PinkBrown

F221: Sample, 7, F2, Gelber, P47-1, Brown mixed, W, pink, brown, brown, red, RedBrown, No, Yes, No, No, No, Yes, BrownPink, BrownPink, Brown, Pink, Pink, BrownPink, BrownPink-PinkBrown

F222: Sample, 9, F2, Gelber, P47-1, Brown mixed, W, pink, brown, brown, red, RedBrown, No, Yes, No, No, No, Yes, BrownPink, BrownPink, Brown, Pink, Pink, BrownPink, BrownPink-PinkBrown

F223: Sample, 11, F2, Snowdrop, P47-1, Brown mixed, W, pink, brown, brown, red, RedBrown, No, Yes, No, No, No, Yes, BrownPink, BrownPink, Brown, Pink, Pink, BrownPink, BrownPink-PinkBrown

F227: Sample, 63, F2, Aurora, P47-1, Brown mixed, W, pink, brown, brown, red, RedBrown, No, Yes, No, No, No, Yes, BrownPink, BrownPink, Brown, Pink, Pink, BrownPink, BrownPink-PinkBrown

F237: Sample, 31, F2, P47-1, Disco/2, Brown mixed, W, brown, red, pink, brown, RedBrown, No, Yes, No, No, No, Yes, BrownPink, BrownPink, Pink, Brown, Brown, PinkBrown, BrownPink-PinkBrown

F346: Sample, 7, F2, Gelber, P47-1, Brown mixed, W, pink, brown, brown, red, RedBrown, No, Yes, No, No, No, Yes, BrownPink, BrownPink, Brown, Pink, Pink, BrownPink, BrownPink-PinkBrown

F347: Sample, 9, F2, Gelber, P47-1, Brown mixed, W, pink, brown, brown, red, RedBrown, No, Yes, No, No, No, Yes, BrownPink, BrownPink, Brown, Pink, Pink, BrownPink, BrownPink-PinkBrown

F348: Sample, 11, F2, Snowdrop, P47-1, Brown mixed, W, pink, brown, brown, red, RedBrown, No, Yes, No, No, No, Yes, BrownPink, BrownPink, Brown, Pink, Pink, BrownPink, BrownPink-PinkBrown

F352: Sample, 63, F2, Aurora, P47-1, Brown mixed, W, pink, brown, brown, red, RedBrown, No, Yes, No, No, No, Yes, BrownPink, BrownPink, Brown, Pink, Pink, BrownPink, BrownPink-PinkBrown

F362: Sample, 31, F2, P47-1, Disco/2, Brown mixed, W, brown, red, pink, brown, RedBrown, No, Yes, No, No, No, Yes, BrownPink, BrownPink, Pink, Brown, Brown, PinkBrown, BrownPink-PinkBrown

W, BrownRed-RedBrown

F70: Sample, 14, F2, Snowdrop, P47-1, Red, W, red, brown, brown, red, RedBrown, Yes, Yes, No, No, No, No, BrownRed, BrownRed, Brown, Red, Red, BrownRed, BrownRed-RedBrown

F71: Sample, 17, F2, Snowdrop, P47-1, Red, W, brown, red, red, brown, RedBrown, Yes, Yes, No, No, No, No, BrownRed, BrownRed, Red, Brown, Brown, RedBrown, BrownRed-RedBrown

F75: Sample, 21, F2, Snowdrop, P47-1, Red, W, brown, red, red, brown, RedBrown, Yes, Yes, No, No, No, No, BrownRed, BrownRed, Red, Brown, Brown, RedBrown, BrownRed-RedBrown

F80: Sample, 61, F2, Aurora, P47-1, Red, W, brown, red, red, brown, RedBrown, Yes, Yes, No, No, No, No, BrownRed, BrownRed, Red, Brown, Brown, RedBrown, BrownRed-RedBrown

F102: Sample, 33, F2, P47-1, Disco/2, Brown mixed, W, red, brown, brown, red, RedBrown, Yes, Yes, No, No, No, No, BrownRed, BrownRed, Brown, Red, Red, BrownRed, BrownRed-RedBrown

F224: Sample, 14, F2, Snowdrop, P47-1, Red, W, red, brown, brown, red, RedBrown, Yes, Yes, No, No, No, No, BrownRed, BrownRed, Brown, Red, Red, BrownRed, BrownRed-RedBrown

F225: Sample, 33, F2, P47-1, Disco/2, Brown mixed, W, red, brown, brown, red, RedBrown, Yes, Yes, No, No, No, No, BrownRed, BrownRed, Brown, Red, Red, BrownRed, BrownRed-RedBrown

F233: Sample, 17, F2, Snowdrop, P47-1, Red, W, brown, red, red, brown, RedBrown, Yes, Yes, No, No, No, No, BrownRed, BrownRed, Red, Brown, Brown, RedBrown, BrownRed-RedBrown

F236: Sample, 21, F2, Snowdrop, P47-1, Red, W, brown, red, red, brown, RedBrown, Yes, Yes, No, No, No, No, BrownRed, BrownRed, Red, Brown, Brown, RedBrown, BrownRed-RedBrown

F242: Sample, 61, F2, Aurora, P47-1, Red, W, brown, red, red, brown, RedBrown, Yes, Yes, No, No, No, No, BrownRed, BrownRed, Red, Brown, Brown, RedBrown, BrownRed-RedBrown

F349: Sample, 14, F2, Snowdrop, P47-1, Red, W, red, brown, brown, red, RedBrown, Yes, Yes, No, No, No, No, BrownRed, BrownRed, Brown, Red, Red, BrownRed, BrownRed-RedBrown

F350: Sample, 33, F2, P47-1, Disco/2, Brown mixed, W, red, brown, brown, red, RedBrown, Yes, Yes, No, No, No, No, BrownRed, BrownRed, Brown, Red, Red, BrownRed, BrownRed-RedBrown

F358: Sample, 17, F2, Snowdrop, P47-1, Red, W, brown, red, red, brown, RedBrown, Yes, Yes, No, No, No, No, BrownRed, BrownRed, Red, Brown, Brown, RedBrown, BrownRed-RedBrown

F361: Sample, 21, F2, Snowdrop, P47-1, Red, W, brown, red, red, brown, RedBrown, Yes, Yes, No, No, No, No, BrownRed, BrownRed, Red, Brown, Brown, RedBrown, BrownRed-RedBrown

F367: Sample, 61, F2, Aurora, P47-1, Red, W, brown, red, red, brown, RedBrown, Yes, Yes, No, No, No, No, BrownRed, BrownRed, Red, Brown, Brown, RedBrown, BrownRed-RedBrown

W, Purplish red

F83: Sample, 72, 2263, Rinrei, P47-1, Red, W, purple, red, red, purple, RedPurple, Yes, No, No, Yes, No, No, RedPurple, RedPurple, Purplish red, Absent, Purplish red, Purplish red, Purplish red

F86: Sample, 75, 71B, P47-1, NV153, Red, W, purple, red, red, purple, RedPurple, Yes, No, No, Yes, No, No, RedPurple, RedPurple, Purplish red, Absent, Purplish red, Purplish red, Purplish red

F252: Sample, 72, 2263, Rinrei, P47-1, Red, W, purple, red, red, purple, RedPurple, Yes, No, No, Yes, No, No, RedPurple, RedPurple, Purplish red, Absent, Purplish red, Purplish red, Purplish red

F253: Sample, 75, 71B, P47-1, NV153, Red, W, purple, red, red, purple, RedPurple, Yes, No, No, Yes, No, No, RedPurple, RedPurple, Purplish red, Absent, Purplish red, Purplish red, Purplish red

F377: Sample, 72, 2263, Rinrei, P47-1, Red, W, purple, red, red, purple, RedPurple, Yes, No, No, Yes, No, No, RedPurple, RedPurple, Purplish red, Absent, Purplish red, Purplish red, Purplish red

F378: Sample, 75, 71B, P47-1, NV153, Red, W, purple, red, red, purple, RedPurple, Yes, No, No, Yes, No, No, RedPurple, RedPurple, Purplish red, Absent, Purplish red, Purplish red, Purplish red

W, White

F22: Sample, 45, F2, Snowdrop, Gelber, White, W, white, white, white, white, White, No, No, No, No, Yes, No, White, White, White, Absent, White, White, White

F25: Sample, 53, F2, Disco/2, Gelber, White, W, white, white, white, white, White, No, No, No, No, Yes, No, White, White, White, Absent, White, White, White

F120: Sample, 27, F2, P47-1, Snowdrop, White, W, white, white, white, white, White, No, No, No, No, Yes, No, White, White, White, Absent, White, White, White

F167: Sample, 27, F2, P47-1, Snowdrop, White, W, white, white, white, white, White, No, No, No, No, Yes, No, White, White, White, Absent, White, White, White

F172: Sample, 45, F2, Snowdrop, Gelber, White, W, white, white, white, white, White, No, No, No, No, Yes, No, White, White, White, Absent, White, White, White

F177: Sample, 53, F2, Disco/2, Gelber, White, W, white, white, white, white, White, No, No, No, No, Yes, No, White, White, White, Absent, White, White, White

F292: Sample, 27, F2, P47-1, Snowdrop, White, W, white, white, white, white, White, No, No, No, No, Yes, No, White, White, White, Absent, White, White, White

F297: Sample, 45, F2, Snowdrop, Gelber, White, W, white, white, white, white, White, No, No, No, No, Yes, No, White, White, White, Absent, White, White, White

F302: Sample, 53, F2, Disco/2, Gelber, White, W, white, white, white, white, White, No, No, No, No, Yes, No, White, White, White, Absent, White, White, White

W, WhiteBrown

F33: Sample, 23, F2, Gelber, P47-1, White with patterns, W, brown, white, white, brown, WhiteBrown, No, Yes, No, No, Yes, No, WhiteBrown, WhiteBrown, White, Brown, Brown, WhiteBrown, WhiteBrown

F38: Sample, 29, F2, P47-1, Snowdrop, White with patterns, W, brown, white, white, brown, WhiteBrown, No, Yes, No, No, Yes, No, WhiteBrown, WhiteBrown, White, Brown, Brown, WhiteBrown, WhiteBrown

F39: Sample, 37, F2, P47-1, Disco/2, White with patterns, W, brown, white, white, brown, WhiteBrown, No, Yes, No, No, Yes, No, WhiteBrown, WhiteBrown, White, Brown, Brown, WhiteBrown, WhiteBrown

F41: Sample, 39, F2, P47-1, Disco/2, White with patterns, W, brown, white, white, brown, WhiteBrown, No, Yes, No, No, Yes, No, WhiteBrown, WhiteBrown, White, Brown, Brown, WhiteBrown, WhiteBrown

F43: Sample, 47, F2, Snowdrop, Gelber, White with patterns, W, brown, white, white, brown, WhiteBrown, No, Yes, No, No, Yes, No, WhiteBrown, WhiteBrown, White, Brown, Brown, WhiteBrown, WhiteBrown

F50: Sample, 55, F2, Disco/2, Gelber, White with patterns, W, brown, white, white, brown, WhiteBrown, No, Yes, No, No, Yes, No, WhiteBrown, WhiteBrown, White, Brown, Brown, WhiteBrown, WhiteBrown

F54: Sample, 57, F2, Aurora, Gelber, White with patterns, W, brown, white, white, brown, WhiteBrown, No, Yes, No, No, Yes, No, WhiteBrown, WhiteBrown, White, Brown, Brown, WhiteBrown, WhiteBrown

F58: Sample, 65, F2, Aurora, P47-1, White with patterns, W, brown, white, white, brown, WhiteBrown, No, Yes, No, No, Yes, No, WhiteBrown, WhiteBrown, White, Brown, Brown, WhiteBrown, WhiteBrown

F188: Sample, 23, F2, Gelber, P47-1, White with patterns, W, brown, white, white, brown, WhiteBrown, No, Yes, No, No, Yes, No, WhiteBrown, WhiteBrown, White, Brown, Brown, WhiteBrown, WhiteBrown

F190: Sample, 29, F2, P47-1, Snowdrop, White with patterns, W, brown, white, white, brown, WhiteBrown, No, Yes, No, No, Yes, No, WhiteBrown, WhiteBrown, White, Brown, Brown, WhiteBrown, WhiteBrown

F191: Sample, 37, F2, P47-1, Disco/2, White with patterns, W, brown, white, white, brown, WhiteBrown, No, Yes, No, No, Yes, No, WhiteBrown, WhiteBrown, White, Brown, Brown, WhiteBrown, WhiteBrown

F193: Sample, 39, F2, P47-1, Disco/2, White with patterns, W, brown, white, white, brown, WhiteBrown, No, Yes, No, No, Yes, No, WhiteBrown, WhiteBrown, White, Brown, Brown, WhiteBrown, WhiteBrown

F195: Sample, 47, F2, Snowdrop, Gelber, White with patterns, W, brown, white, white, brown, WhiteBrown, No, Yes, No, No, Yes, No, WhiteBrown, WhiteBrown, White, Brown, Brown, WhiteBrown, WhiteBrown

F199: Sample, 55, F2, Disco/2, Gelber, White with patterns, W, brown, white, white, brown, WhiteBrown, No, Yes, No, No, Yes, No, WhiteBrown, WhiteBrown, White, Brown, Brown, WhiteBrown, WhiteBrown

F201: Sample, 57, F2, Aurora, Gelber, White with patterns, W, brown, white, white, brown, WhiteBrown, No, Yes, No, No, Yes, No, WhiteBrown, WhiteBrown, White, Brown, Brown, WhiteBrown, WhiteBrown

F203: Sample, 65, F2, Aurora, P47-1, White with patterns, W, brown, white, white, brown, WhiteBrown, No, Yes, No, No, Yes, No, WhiteBrown, WhiteBrown, White, Brown, Brown, WhiteBrown, WhiteBrown

F313: Sample, 23, F2, Gelber, P47-1, White with patterns, W, brown, white, white, brown, WhiteBrown, No, Yes, No, No, Yes, No, WhiteBrown, WhiteBrown, White, Brown, Brown, WhiteBrown, WhiteBrown

F315: Sample, 29, F2, P47-1, Snowdrop, White with patterns, W, brown, white, white, brown, WhiteBrown, No, Yes, No, No, Yes, No, WhiteBrown, WhiteBrown, White, Brown, Brown, WhiteBrown, WhiteBrown

F316: Sample, 37, F2, P47-1, Disco/2, White with patterns, W, brown, white, white, brown, WhiteBrown, No, Yes, No, No, Yes, No, WhiteBrown, WhiteBrown, White, Brown, Brown, WhiteBrown, WhiteBrown

F318: Sample, 39, F2, P47-1, Disco/2, White with patterns, W, brown, white, white, brown, WhiteBrown, No, Yes, No, No, Yes, No, WhiteBrown, WhiteBrown, White, Brown, Brown, WhiteBrown, WhiteBrown

F320: Sample, 47, F2, Snowdrop, Gelber, White with patterns, W, brown, white, white, brown, WhiteBrown, No, Yes, No, No, Yes, No, WhiteBrown, WhiteBrown, White, Brown, Brown, WhiteBrown, WhiteBrown

F324: Sample, 55, F2, Disco/2, Gelber, White with patterns, W, brown, white, white, brown, WhiteBrown, No, Yes, No, No, Yes, No, WhiteBrown, WhiteBrown, White, Brown, Brown, WhiteBrown, WhiteBrown

F326: Sample, 57, F2, Aurora, Gelber, White with patterns, W, brown, white, white, brown, WhiteBrown, No, Yes, No, No, Yes, No, WhiteBrown, WhiteBrown, White, Brown, Brown, WhiteBrown, WhiteBrown

F328: Sample, 65, F2, Aurora, P47-1, White with patterns, W, brown, white, white, brown, WhiteBrown, No, Yes, No, No, Yes, No, WhiteBrown, WhiteBrown, White, Brown, Brown, WhiteBrown, WhiteBrown

W, WhiteYellow

F35: Sample, 25, F2, Gelber, P47-1, White with patterns, W, yellow, white, white, brown, WhiteYellow, No, No, Yes, No, Yes, No, WhiteYellow, WhiteYellow, White, Yellow, Yellow, WhiteYellow, WhiteYellow

F49: Sample, 51, F2, Disco/2, Gelber, White with patterns, W, yellow, white, white, brown, WhiteYellow, No, No, Yes, No, Yes, No, WhiteYellow, WhiteYellow, White, Yellow, Yellow, WhiteYellow, WhiteYellow

F121: Sample, 59, F2, Aurora, Gelber, White with patterns, W, yellow, white, white, brown, WhiteYellow, No, No, Yes, No, Yes, No, WhiteYellow, WhiteYellow, White, Yellow, Yellow, WhiteYellow, WhiteYellow

F181: Sample, 25, F2, Gelber, P47-1, White with patterns, W, yellow, white, white, brown, WhiteYellow, No, No, Yes, No, Yes, No, WhiteYellow, WhiteYellow, White, Yellow, Yellow, WhiteYellow, WhiteYellow

F183: Sample, 51, F2, Disco/2, Gelber, White with patterns, W, yellow, white, white, brown, WhiteYellow, No, No, Yes, No, Yes, No, WhiteYellow, WhiteYellow, White, Yellow, Yellow, WhiteYellow, WhiteYellow

F184: Sample, 59, F2, Aurora, Gelber, White with patterns, W, yellow, white, white, brown, WhiteYellow, No, No, Yes, No, Yes, No, WhiteYellow, WhiteYellow, White, Yellow, Yellow, WhiteYellow, WhiteYellow

F306: Sample, 25, F2, Gelber, P47-1, White with patterns, W, yellow, white, white, brown, WhiteYellow, No, No, Yes, No, Yes, No, WhiteYellow, WhiteYellow, White, Yellow, Yellow, WhiteYellow, WhiteYellow

F308: Sample, 51, F2, Disco/2, Gelber, White with patterns, W, yellow, white, white, brown, WhiteYellow, No, No, Yes, No, Yes, No, WhiteYellow, WhiteYellow, White, Yellow, Yellow, WhiteYellow, WhiteYellow

F309: Sample, 59, F2, Aurora, Gelber, White with patterns, W, yellow, white, white, brown, WhiteYellow, No, No, Yes, No, Yes, No, WhiteYellow, WhiteYellow, White, Yellow, Yellow, WhiteYellow, WhiteYellow

------------------------------------------------------------------

Ratios:

------------------------------------------------------------------

(ST, BrownPink-PinkBrown) / (ST, Brown)

(ST, BrownRed-RedBrown) / (ST, Brown)

(ST, Purplish red) / (ST, Brown)

(ST, Red) / (ST, Brown)

(ST, WhiteBrown) / (ST, Brown)

(ST, WhitePink) / (ST, Brown)

(ST, WhiteYellow) / (ST, Brown)

(ST, BrownPink-PinkBrown) / (ST, Red)

(ST, BrownRed-RedBrown) / (ST, Red)

(ST, Purplish red) / (ST, Red)

(ST, WhiteBrown) / (ST, Red)

(ST, WhitePink) / (ST, Red)

(ST, WhiteYellow) / (ST, Red)

(ST, Brown) / (ST, White)

(ST, BrownPink-PinkBrown) / (ST, White)

(ST, BrownRed-RedBrown) / (ST, White)

(ST, Purplish red) / (ST, White)

(ST, Red) / (ST, White)

(ST, WhiteBrown) / (ST, White)

(ST, WhitePink) / (ST, White)

(ST, WhiteYellow) / (ST, White)

(W, BrownPink-PinkBrown) / (W, Brown)

(W, BrownRed-RedBrown) / (W, Brown)

(W, Purplish red) / (W, Brown)

(W, WhiteBrown) / (W, Brown)

(W, WhiteYellow) / (W, Brown)

(W, Brown) / (W, White)

(W, BrownPink-PinkBrown) / (W, White)

(W, BrownRed-RedBrown) / (W, White)

(W, Purplish red) / (W, White)

(W, WhiteBrown) / (W, White)

(W, WhiteYellow) / (W, White)
